# Asian-White racial disparities in postpartum hemorrhage and severe postpartum hemorrhage in Ontario, Canada: A population-based cohort study

**DOI:** 10.1371/journal.pone.0344365

**Published:** 2026-03-12

**Authors:** Parnian Hossein-Pour, Rohan D’Souza, Anastasia Gayowsky, Irina Oltean, Esther Chin, Azar Mehrabadi, Giulia M. Muraca

**Affiliations:** 1 Department of Obstetrics and Gynecology, Faculty of Health Sciences, McMaster University, Hamilton, Ontario, Canada; 2 Department of Health Research Methods, Evidence, and Impact, McMaster University, Hamilton, Ontario, Canada; 3 ICES McMaster, Hamilton, Ontario, Canada; 4 Departments of Obstetrics & Gynaecology and Pediatrics, Dalhousie University, Halifax, Nova Scotia, Canada; 5 Clinical Epidemiology Unit, Department of Medicine, Solna, Karolinska Institutet, Stockholm, Sweden; Kasr Alainy Medical School, Cairo University, EGYPT

## Abstract

**Background:**

Postpartum hemorrhage (PPH) is the leading preventable cause of maternal morbidity and mortality globally, occurring in 4–6% of Canadian deliveries with evidence suggesting higher rates among Asian individuals. We compared rates of PPH and severe PPH in Ontario, Canada, among Asian and White individuals, focusing on the intersectional relationships between race, language, and immigration status.

**Methods:**

We performed a population-based cohort study in Ontario, Canada (2013–2021). PPH was identified by diagnosis codes used to indicate blood loss of ≥500 mL (vaginal delivery) or ≥1000 mL (cesarean delivery). Severe PPH was defined as PPH with an intervention to control bleeding. Rates were examined by maternal self-reported race, immigration category, duration in Canada, and language at immigration. Modified Poisson regression models were fit to determine the relationships between race, PPH and severe PPH. Models were adjusted for maternal sociodemographic, clinical and obstetric practice factors.

**Results:**

The study included 637,311 deliveries (30.9% Asian, 69.1% White). PPH and severe PPH occurred in 5.5% and 6.8%, respectively, of primipara, and 3.8% and 4.3%, of multipara. Asian race was associated with marginally increased PPH rates among multipara after adjusting for confounding (adjusted rate ratio (aRR) 1.06, 95% CI 1.01–1.10). Asian and White individuals experienced similar rates of severe PPH in an adjusted model (aRR 1.00, 95% CI 0.91–1.09). Overall, immigrants experienced lower rates of PPH than non-immigrants (4.4% vs. 5.5%, p-value <0.01); however, differences were observed after layering primary language, with Southeast Asian language speakers having the highest rates (6.3% vs. 5.5%; aRR of 1.40 (95% CI 1.18–1.54) compared with White individuals.

**Discussion:**

Variation in PPH and severe PPH risk was observed across racial, immigration, and language groups in Ontario, with modest differences overall but meaningful heterogeneity across subgroups. More granular consideration of immigration characteristics may improve understanding of maternal health inequities.

## Introduction

Postpartum hemorrhage (PPH) is the leading preventable cause of maternal morbidity and mortality globally [1]. In Canada, PPH occurs in 4–6% of all deliveries and increasing rates of PPH have been recently reported in Canada and several other countries [2,3]. Severe PPH, defined as PPH requiring blood transfusion, hysterectomy, or interventions to control bleeding, occurs in 4.3 per 1,000 deliveries in Canada and has a case fatality rate of 2.4 per 1,000 cases [4]. Long-term complications of PPH are not well understood, but a recent study using longitudinal data in Québec showed that experiencing severe PPH was associated with a nearly two-fold higher risk of mortality after 10 years compared with those without severe PPH [5]. In Ontario, hemorrhage remains the leading cause of pregnancy-related deaths, accounting for 27.2% of maternal deaths between 2002 and 2022 [6]. Identifying pregnant individuals at heightened risk of PPH is critical, as severe morbidity and mortality resulting from PPH can be avoided with appropriate mobilization of resources and hemorrhage preparedness [7]. Asian individuals represent the largest and fastest-growing minority group in Canada, comprising approximately 19% of the national population and 25% of Ontario’s population [8].

Several previous investigations have demonstrated an association between PPH and Asian maternal race [9–15]. In particular, a systematic review and meta-analysis of risk factors for PPH found that Asian race was a definitive risk factor for PPH with a 40% increased risk in Asian vs White individuals [16]. Notably, the data on racial disparities in PPH included in this review were all from the United States (US). While population-based analyses of Asian-White disparities in pregnancy outcomes in Canada have not assessed PPH, there have been reports of higher rates of pregnancy complications associated with PPH, namely, placental abnormalities, operative delivery, and severe perineal trauma among individuals with Asian race [17].

Due to the multiple forms of individual and structural discrimination that can contribute to an individual’s experience, there is an urgent need to account for intersectionality, a framework that explains how different types of discrimination, such as xenophobia and racism, overlap and intensify one another, when exploring health disparities [18,19]. For example, an individual’s mother tongue can shape how they are perceived in health care settings, regardless of their race or country of origin, due to linguistic/accent discrimination [20–25]. Immigration-related factors are particularly relevant in the Canadian context, as approximately 70% of Asian individuals are immigrants compared to 22% of White individuals in Canada [26]. Failure to account for immigration-related factors, such as immigrant status, primary language, and duration of residence in Canada, in comparisons between race groups can obscure the role of systemic racism and discrimination in shaping health outcomes, potentially masking the true health disparities faced by racial minorities in Canada. To that end, the primary aims of this study were to quantify the variation in PPH and severe PPH between Asian and White individuals and to explore the intersectional relationships between race and immigration-related factors: immigrant status, maternal primary language, and duration of residence in Canada.

## Methods

### Study population and data source

We conducted a population-based, retrospective cohort study of pregnant individuals with a live birth or stillbirth >20 weeks’ gestation in Ontario, Canada between April 1, 2013 and March 31, 2021. This study was facilitated through ICES, an independent, non-profit research institute whose legal status under Ontario’s health information privacy law allows it to collect and analyze health care and demographic data, without consent, for health system evaluation and improvement (https://www.ices.on.ca/). Data sets used for this study are valid and reliable sources for perinatal research and are described in S1 Table [27–35]. These datasets were linked using unique encoded identifiers and analyzed at ICES.

### Exposure and outcome variables

Maternal race information (self-reported and recorded by health care providers as Asian, Black, White, or Other) was obtained from prenatal genetic screening records. Thus, the study population was restricted to the approximately 70% of birthing individuals in Ontario that accessed prenatal genetic screening.

We obtained information on immigration-related characteristics from the Immigration, Refugees and Citizenship Canada (IRCC) Permanent Residents database. Specifically, immigration admission category (economic, family, refugee, other), landing date, and primary language (mother tongue) of immigrants to Canada were collected [36]. Using publicly available sources including The World Factbook, primary language at time of immigration was mapped to a world region based on the country or region where each language is most spoken (S2 Table) [37]. This mapping was then used to report rates of PPH and severe PPH among immigrants who self-identified as Asian by world region of primary language in the following groups: West Asia, Central Asia, East Asia, Southeast Asia, and South Asia (S2 Table). Duration of residence in Canada was calculated as time from landing date to the index birth date among foreign-born individuals and as time from mother’s date of birth to date of index birth for those born in Canada.

PPH was identified by diagnosis codes noted by a health care provider used to indicate blood loss of ≥500 mL following vaginal delivery or ≥1000 mL following cesarean delivery, as defined by Society of Obstetricians and Gynaecologist of Canada guidelines [38]. Severe PPH was defined as PPH requiring blood transfusion or additional interventions to control bleeding (e.g., curettage, compression sutures, uterine or other pelvic artery ligation, or hysterectomy) and was identified using hospital diagnosis and procedure codes using an algorithm adopted by the Canadian Perinatal Surveillance System [5].

### Statistical analysis

We created two models to compare the incidence of PPH and severe PPH among Asian and White individuals. Model 1 used the variable of self-reported maternal race for the exposure (i.e., Asian vs. White) and stratified results by parity (nulliparous and parous). In Model 2, the Asian group was disaggregated to distinguish 1) Asian individuals who were immigrants versus non-immigrants, and 2) among Asian immigrants, maternal primary language/language region (e.g., English, East Asian language, South Asian language). In Model 2, due to limited sample sizes within immigrant language groups, parity was included as a confounder rather than used for stratified analyses. Minimum detectable effect sizes were estimated for each group relative to the reference population (White individuals) based on 80% power and a 0.05 significance level (S3 Table).

The distribution of maternal characteristics was assessed by self-reported race, immigration status, and maternal primary language world region among Asian immigrants. Standardized differences for binary and median variables were computed to compare the White and Asian groups. Crude rates of PPH and severe PPH were reported by self-reported race, immigration category, and maternal primary language world region among Asian immigrants. Multivariable Poisson regression models were used to estimate unadjusted and adjusted total effects, presented as relative rates (aRRs) and 95% confidence intervals (CIs) of PPH and severe PPH. Predicted probabilities of PPH were calculated by duration of residence in Canada and maternal race and language world region.

Models adjusted for duration of residence in Canada (continuous), maternal race*duration of residence in Canada, age at delivery (< 20, 25–29, 30–34, 35–39, 40 + vs. 20–24), parity (primiparous vs multiparous; Model 2 only), plurality, pre-pregnancy body mass index (< 18.5 kg/m^2^, 25.0–29.9 kg/m^2^, ≥ 30 kg/m^2^ vs. 18.5–24.9 kg/m^2^), age and labour force quintile (previously dependency quintile; Q2, Q3, Q4, Q5 vs. Q1); maternal resources quintile (previously deprivation quintile; Q2, Q3, Q4, Q5 vs. Q1), households and dwellings quintile (previously instability quintile; Q2, Q3, Q4, Q5 vs. Q1), maternal immigration admission category (economic, family, resettled refugee and protected person, other vs. not an immigrant), geographic location (rural vs. urban), tobacco use in pregnancy, drug and substance exposure in pregnancy, type of conception (assisted, unknown vs. spontaneous), first trimester prenatal visit, pre-existing diabetes, gestational diabetes, pre-existing hypertension, pregnancy-induced hypertension, previous cesarean delivery, placenta previa, placenta accreta spectrum, placental abruption, labour induction, labour augmentation, episiotomy, fetal presentation (breech, transverse, unknown vs. cephalic), duration of second stage labour (60–119 minutes, 120–179 minutes, 180–239 minutes, 240 + minutes, no second stage vs. < 60 minutes), mode of delivery (forceps delivery, vacuum delivery, forceps and vacuum delivery, operative vaginal delivery (forceps/vacuum unknown), first stage cesarean delivery, second stage cesarean delivery, cesarean delivery without labour, cesarean delivery (missing/unknown stage) or perimortem cesarean delivery vs. spontaneous vaginal delivery), gestational age (preterm, post-term, vs. term), infant birth weight (<3000g, 4000-4499g, ≥ 4500g, missing vs. 3000-3999g),and infant head circumference at birth (<33 cm, 35–36 cm, ≥ 37 cm. vs. 33–34 cm). Confounders were selected based on prior literature and consultation with clinical experts [10].

### Sensitivity analyses

Model 2, which reports rates of PPH by maternal primary language among immigrants, was restricted to individuals who self-identified as Asian so that it aligned with the population used in Model 1. However, many individuals who reported Asian languages as their primary languages, particularly from West and Central Asia, did not self-identify as Asian at prenatal screening and thus were not captured in Model 2. These individuals may have self-identified as White, Black, Other, or had missing information on race. To address this, we conducted a sensitivity analysis using the same Asian language data and categorizations without restricting to those who had self-identified as Asian to determine the effect of this restriction.

Additionally, we assessed the robustness of the results to unmeasured confounding using E-value methodology [39].

This study followed the Strengthening the Reporting of Observational Studies in Epidemiology (STROBE) reporting guideline [40]. Ethics approval was obtained from the Hamilton Integrated Research Ethics Board (Project ID 14419). Data management and analysis were performed using SAS, version 9.4 (SAS Institute Inc). All tests used 2-tailed significance set at p < .05.

## Results

**Fig 1:**
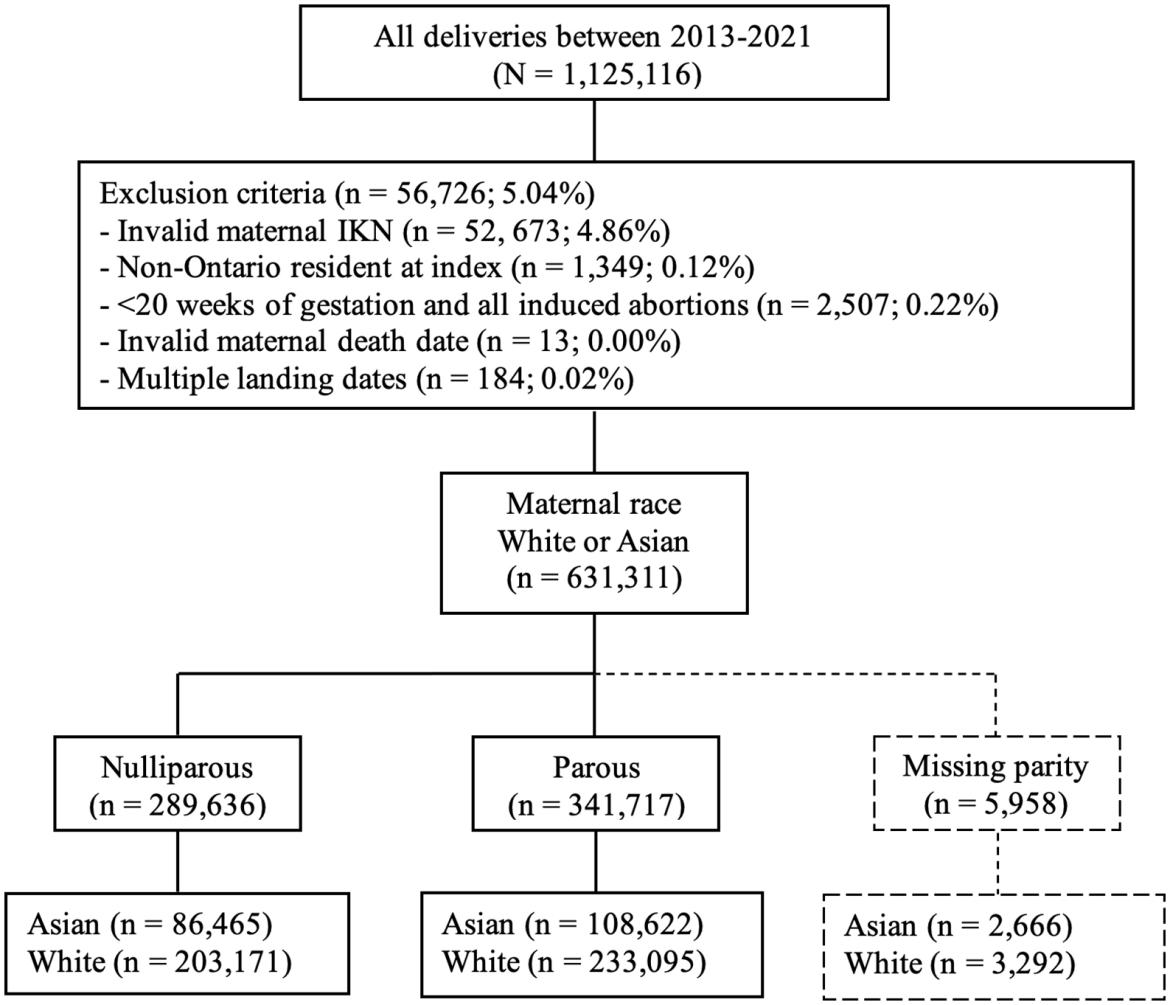
Study population derivation.

A total of 637,311 deliveries from 2013–2021 were included where White individuals represented 69.1% of the population and Asian individuals 30.9% (Fig 1). Approximately 70% of Asian individuals in this study population were immigrants (Table 1). Among Asian immigrants, the language distribution was 52.0% from South Asia, 26.8% from East Asia, 19.0% from Southeast Asia, 1.4% from Central Asia, and 1.0% from West Asia.

**Table 1:** Demographic and clinical characteristics of individuals with births after 20 weeks’ gestation in Ontario, Canada, 2013-2021, (N = 637,311).

	Self-reported race		Primary language/ language world region among Asian immigrants								
	White	Asian	Standardized difference	Not an immigrant	Central Asia	East Asia	South Asia	Southeast Asia	West Asia	English	Other
	N = 439,558	N = 197,753		N = 58,805	N = 1,761	N = 34,008	N = 66,037	N = 24,100	N = 1,205	N = 6,428	N = 5,409
Maternal Characteristics, n (%)											
Maternal age (years)											
< 20	6,156 (1.4%)	363 (0.2%)	0.138	212 (0.4%)	<10	<10	44 (0.1%)	63 (0.3%)	<10	12 (0.2%)	16 (0.3%)
20-24	37,115 (8.4%)	8,046 (4.1%)	0.181	2,292 (3.9%)	230 (13.1%)	892 (2.6%)	3,178 (4.8%)	854 (3.5%)	98 (8.1%)	228 (3.5%)	274 (5.1%)
25-29	112,166 (25.5%)	47,926 (24.2%)	0.030	12,216 (20.8%)	676 (38.4%)	8,369 (24.6%)	19,910 (30.1%)	4,038 (16.8%)	289 (24.0%)	1,387 (21.6%)	1,041 (19.2%)
30-34	176,554 (40.2%)	83,735 (42.3%)	0.044	25,366 (43.1%)	538 (30.6%)	14,460 (42.5%)	29,081 (44.0%)	9,045 (37.5%)	461 (38.3%)	2,820 (43.9%)	1,964 (36.3%)
35-39	92,559 (21.1%)	48,556 (24.6%)	0.083	15,949 (27.1%)	240 (13.6%)	8,513 (25.0%)	12,096 (18.3%)	8,076 (33.5%)	294 (24.4%)	1,676 (26.1%)	1,712 (31.7%)
≥ 40	15,008 (3.4%)	9,127 (4.6%)	0.061	2,770 (4.7%)	72-76	1,765 (5.2%)	1,728 (2.6%)	2,024 (8.4%)	58-62	305 (4.7%)	402 (7.4%)
Parity											
0	203,171 (46.2%)	86,465 (43.7%)	0.050	28,027 (47.7%)	602 (34.2%)	15,572 (45.8%)	26,874 (40.7%)	10,032 (41.6%)	485 (40.2%)	2,906 (45.2%)	1,967 (36.4%)
1	158,665 (36.1%)	74,125 (37.5%)	0.029	21,506 (36.6%)	504 (28.6%)	13,405 (39.4%)	25,001 (37.9%)	9,079 (37.7%)	393 (32.6%)	2,319 (36.1%)	1,918 (35.5%)
2-3	67,999 (15.5%)	32,281 (16.3%)	0.023	7,925 (13.5%)	537 (30.5%)	3,978 (11.7%)	12,862 (19.5%)	4,411 (18.3%)	261 (21.7%)	1,074 (16.7%)	1,233 (22.8%)
≥ 4	6,431 (1.5%)	2,216 (1.1%)	0.030	523 (0.9%)	104 (5.9%)	97 (0.3%)	866 (1.3%)	271 (1.1%)	54 (4.5%)	71 (1.1%)	230 (4.3%)
Unknown	3,292 (0.7%)	2,666 (1.3%)	0.059	824 (1.4%)	14 (0.8%)	956 (2.8%)	434 (0.7%)	307 (1.3%)	12 (1.0%)	58 (0.9%)	61 (1.1%)
Multiple pregnancy	238 (0.1%)	89 (<0.1%)	0.004	22 (<0.1%)	0	9 (<0.1%)	40 (0.1%)	11 (<0.1%)	0	<10	<10
Pre-pregnancy body mass index (kg/m^2^)											
< 18.5	14,980 (3.4%)	13,293 (6.7%)	0.152	3,555 (6.0%)	80 (4.5%)	4,619 (13.6%)	2,964 (4.5%)	1,448 (6.0%)	37 (3.1%)	351 (5.5%)	239 (4.4%)
18.5-24.9	196,478 (44.7%)	94,219 (47.6%)	0.059	28,512 (48.5%)	655 (37.2%)	20,244 (59.5%)	26,218 (39.7%)	12,878 (53.4%)	521 (43.2%)	2,789 (43.4%)	2,402 (44.4%)
25-29.9	95,343 (21.7%)	34,713 (17.6%)	0.104	10,133 (17.2%)	420 (23.9%)	3,149 (9.3%)	14,457 (21.9%)	3,940 (16.3%)	278 (23.1%)	1,247 (19.4%)	1,089 (20.1%)
30-34.9	44,964 (10.2%)	10,904 (5.5%)	0.176	3,513 (6.0%)	141 (8.0%)	510 (1.5%)	4,713 (7.1%)	1,106 (4.6%)	89 (7.4%)	449 (7.0%)	383 (7.1%)
35-39.9	21,016 (4.8%)	2,647 (1.3%)	0.201	950 (1.6%)	38 (2.2%)	102 (0.3%)	1,069 (1.6%)	229 (1.0%)	27 (2.2%)	128 (2.0%)	104 (1.9%)
≥ 40	15,911 (3.6%)	1,687 (0.9%)	0.188	602 (1.0%)	20 (1.1%)	226 (0.7%)	515 (0.8%)	190 (0.8%)	17 (1.4%)	66 (1.0%)	51 (0.9%)
Unknown	50,866 (11.6%)	40,290 (20.4%)	0.242	11,540 (19.6%)	407 (23.1%)	5,158 (15.2%)	16,101 (24.4%)	4,309 (17.9%)	236 (19.6%)	1,398 (21.7%)	1,141 (21.1%)
Age and labour force quintile (previously dependency quintile)											
Q1	138,079 (31.4%)	88,492 (44.7%)	0.277	27,126 (46.1%)	931 (52.9%)	11,307 (33.2%)	33,449 (50.7%)	9,599 (39.8%)	534 (44.3%)	3,082 (47.9%)	2,464 (45.6%)
Q2	89,487 (20.4%)	45,797 (23.2%)	0.068	13,311 (22.6%)	375 (21.3%)	7,741 (22.8%)	15,828 (24.0%)	5,569 (23.1%)	280 (23.2%)	1,490 (23.2%)	1,203 (22.2%)
Q3	77,213 (17.6%)	26,825 (13.6%)	0.111	7,900 (13.4%)	233 (13.2%)	5,545 (16.3%)	7,743 (11.7%)	3,691 (15.3%)	152 (12.6%)	836 (13.0%)	725 (13.4%)
Q4	70,151 (16.0%)	22,287 (11.3%)	0.137	6,242 (10.6%)	119 (6.8%)	5,865 (17.2%)	5,519 (8.4%)	3,141 (13.0%)	145 (12.0%)	635 (9.9%)	621 (11.5%)
Q5	62,891 (14.3%)	13,999 (7.1%)	0.236	4,105 (7.0%)	98-102	3,483 (10.2%)	3,400 (5.1%)	2,059 (8.5%)	94 (7.8%)	373 (5.8%)	383-387
Missing	1,737 (0.4%)	353 (0.2%)	0.041	121 (0.2%)	<10	67 (0.2%)	98 (0.1%)	41 (0.2%)	0	12 (0.2%)	9-13
Material resources quintile (previously deprivation quintile)											
Q1	107,408 (24.4%)	33,216 (16.8%)	0.190	13,440 (22.9%)	174 (9.9%)	7,486 (22.0%)	7,260 (11.0%)	2,649 (11.0%)	222 (18.4%)	1,192 (18.5%)	793 (14.7%)
Q2	99,689 (22.7%)	39,632 (2 < 0.1%)	0.064	13,944 (23.7%)	268 (15.2%)	8,349 (24.6%)	11,092 (16.8%)	3,524 (14.6%)	243 (20.2%)	1,300 (20.2%)	912 (16.9%)
Q3	84,007 (19.1%)	41,377 (20.9%)	0.045	12,738 (21.7%)	338 (19.2%)	5,934 (17.4%)	15,424 (23.4%)	4,405 (18.3%)	213 (17.7%)	1,383 (21.5%)	942 (17.4%)
Q4	73,175 (16.6%)	41,722 (21.1%)	0.114	10,342 (17.6%)	308-312	5,787 (17.0%)	16,754 (25.4%)	5,784 (24.0%)	192 (15.9%)	1,381 (21.5%)	1170-1174
Q5	73,542 (16.7%)	41,453 (21.0%)	0.108	8,220 (14.0%)	668 (37.9%)	6,385 (18.8%)	15,409 (23.3%)	7,697 (31.9%)	335 (27.8%)	1,160 (18.0%)	1,579 (29.2%)
Missing	1,737 (0.4%)	353 (0.2%)	0.041	121 (0.2%)	<10	67 (0.2%)	98 (0.1%)	41 (0.2%)	0	12 (0.2%)	9-13
Households and dwellings quintile (previously instability quintile)											
Q1	81,079 (18.4%)	67,981 (34.4%)	0.367	20,362 (34.6%)	485 (27.5%)	12,033 (35.4%)	27,059 (41.0%)	4,326 (18.0%)	315 (26.1%)	2,194 (34.1%)	1,207 (22.3%)
Q2	88,827 (20.2%)	30,886 (15.6%)	0.120	9,708 (16.5%)	181 (10.3%)	6,254 (18.4%)	9,426 (14.3%)	3,341 (13.9%)	173 (14.4%)	1,036 (16.1%)	767 (14.2%)
Q3	89,066 (20.3%)	25,794 (13.0%)	0.195	8,150 (13.9%)	196 (11.1%)	4,551 (13.4%)	7,803 (11.8%)	3,345 (13.9%)	143 (11.9%)	905 (14.1%)	701 (13.0%)
Q4	87,423 (19.9%)	24,871 (12.6%)	0.199	6,920 (11.8%)	317-321	3,392 (1 < 0.1%)	7,831 (11.9%)	4,575 (19.0%)	167 (13.9%)	801 (12.5%)	864-868
Q5	91,426 (20.8%)	47,868 (24.2%)	0.082	13,544 (23.0%)	577 (32.8%)	7,711 (22.7%)	13,820 (20.9%)	8,472 (35.2%)	407 (33.8%)	1,480 (23.0%)	1,857 (34.3%)
Missing	1,737 (0.4%)	353 (0.2%)	0.041	121 (0.2%)	<10	67 (0.2%)	98 (0.1%)	41 (0.2%)	0	12 (0.2%)	9-13
Maternal immigrant/refugee status											
Not an immigrant	380,590 (86.6%)	58,805 (29.7%)	1.410	58,805 (29.7%)	0	0	0	0	0	0	0
Economic	22,544 (5.1%)	60,696 (30.7%)	0.707	0	35 (2.0%)	15,551 (45.7%)	24,598 (37.2%)	14,398 (59.7%)	253 (21.0%)	3,171 (49.3%)	2,690 (49.7%)
Family	22,961 (5.2%)	65,302 (33.0%)	0.756	0	764 (43.4%)	15,317 (45.0%)	35,339 (53.5%)	8,731 (36.2%)	419 (34.8%)	3,003 (46.7%)	1,729 (32.0%)
Resettled refugee & protected person	12,630 (2.9%)	11,816 (6.0%)	0.151	0	949 (53.9%)	2,718 (8.0%)	5,623 (8.5%)	876 (3.6%)	523 (43.4%)	174 (2.7%)	953 (17.6%)
Other	833 (0.2%)	1,134 (0.6%)	0.062	0	13 (0.7%)	422 (1.2%)	477 (0.7%)	95 (0.4%)	10 (0.8%)	80 (1.2%)	37 (0.7%)
Maternal duration of residence in Canada (years)											
< 1 year	2,674 (0.6%)	8,086 (4.1%)	0.231	0	124 (7.0%)	1,767 (5.2%)	4,407 (6.7%)	1,289 (5.3%)	65 (5.4%)	225 (3.5%)	209 (3.9%)
1–5 years	14,995 (3.4%)	46,536 (23.5%)	0.617	0	531 (30.2%)	10,236 (30.1%)	24,451 (37.0%)	7,869 (32.7%)	374 (31.0%)	1,505 (23.4%)	1,570 (29.0%)
> 5–10 years	11,488 (2.6%)	34,340 (17.4%)	0.508	0	418 (23.7%)	8,924 (26.2%)	16,006 (24.2%)	5,986 (24.8%)	249 (20.7%)	1,287 (2 < 0.1%)	1,470 (27.2%)
> 10 years	27,692 (6.3%)	44,709 (22.6%)	0.477	0	672 (38.2%)	10,997 (32.3%)	19,301 (29.2%)	7,985 (33.1%)	485 (40.2%)	3,227 (50.2%)	2,042 (37.8%)
Median (Q1-Q3)	9 (3-19)	6 (3-12)	0.307	NA	7 (3-13)	6 (3-12)	6 (2-11)	6 (3-13)	7 (3-15)	10 (4-21)	7 (4-15)
Landing date after delivery date	2,119 (0.5%)	5,277 (2.7%)	0.176	0	16 (0.9%)	2,084 (6.1%)	1,872 (2.8%)	971 (4.0%)	32 (2.7%)	184 (2.9%)	118 (2.2%)
Geographic location											
Urban	392,773 (89.4%)	196,411 (99.3%)	0.442	58,331 (99.2%)	1754-1758	33,814 (99.4%)	65,667 (99.4%)	23,891 (99.1%)	1200-1204	6,369 (99.1%)	5,381 (99.5%)
Rural	46,252 (10.5%)	1,036 (0.5%)	0.449	371 (0.6%)	<10	135 (0.4%)	281 (0.4%)	177 (0.7%)	<10	48 (0.7%)	19-23
Pregnancy and Delivery Characteristics											
Tobacco use at first prenatal visit	40,626 (9.2%)	1,630 (0.8%)	0.392	784 (1.3%)	13 (0.7%)	204 (0.6%)	235 (0.4%)	231 (1.0%)	19 (1.6%)	77 (1.2%)	67 (1.2%)
Tobacco use at delivery date	32,245 (7.3%)	1,038 (0.5%)	0.356	466 (0.8%)	8 (0.5%)	120 (0.4%)	170 (0.3%)	158 (0.7%)	18 (1.5%)	49 (0.8%)	49 (0.9%)
Drug and substance exposure in pregnancy	7,300 (1.7%)	413 (0.2%)	0.151	188 (0.3%)	<10	39 (0.1%)	108 (0.2%)	40 (0.2%)	<10	15 (0.2%)	15 (0.3%)
Type of conception											
Spontaneous	398,068 (90.6%)	178,305 (90.2%)	0.013	51,729 (88.0%)	1,658 (94.2%)	31,215 (91.8%)	60,222 (91.2%)	21,892 (90.8%)	1,095 (90.9%)	5,652 (87.9%)	4,842 (89.5%)
Assisted	18,189 (4.1%)	7,165 (3.6%)	0.027	2,463 (4.2%)	52 (3.0%)	1,096 (3.2%)	2,547 (3.9%)	507 (2.1%)	46 (3.8%)	286 (4.4%)	168 (3.1%)
Unknown	23,301 (5.3%)	12,283 (6.2%)	0.039	4,613 (7.8%)	51 (2.9%)	1,697 (5.0%)	3,268 (4.9%)	1,701 (7.1%)	64 (5.3%)	490 (7.6%)	399 (7.4%)
First trimester prenatal visit	384,699 (87.5%)	164,904 (83.4%)	0.117	48,682 (82.8%)	1,456 (82.7%)	28,891 (85.0%)	55,532 (84.1%)	19,732 (81.9%)	1,010 (83.8%)	5,227 (81.3%)	4,374 (80.9%)
Median (Q1-Q3) Number of prenatal visits	11 (9-13)	10 (8-12)	0.209	10 (9-12)	10 (8-12)	11 (9-12)	10 (8-12)	10 (8-12)	10 (8-12)	10 (8-12)	10 (8-12)
Pre-existing diabetes	6,978 (1.6%)	4,808 (2.4%)	0.060	1,353 (2.3%)	42 (2.4%)	362 (1.1%)	2,065 (3.1%)	589 (2.4%)	31 (2.6%)	226 (3.5%)	140 (2.6%)
Gestational diabetes	25,866 (5.9%)	28,827 (14.6%)	0.290	7,435 (12.6%)	268 (15.2%)	4,184 (12.3%)	11,046 (16.7%)	3,978 (16.5%)	151 (12.5%)	1,017 (15.8%)	748 (13.8%)
Pre-existing hypertension	14,325 (3.3%)	5,078 (2.6%)	0.041	1,469 (2.5%)	40 (2.3%)	549 (1.6%)	1,595 (2.4%)	998 (4.1%)	23 (1.9%)	217 (3.4%)	187 (3.5%)
Hypertensive disorders of pregnancy											
Preeclampsia	17,620 (4.0%)	5,973 (3.0%)	0.054	1,762 (3.0%)	41 (2.3%)	428 (1.3%)	2,013 (3.0%)	1,238 (5.1%)	22 (1.8%)	262 (4.1%)	207 (3.8%)
Eclampsia	209 (<0.1%)	107 (0.1%)	0.003	31 (0.1%)	<10	9-13	41 (0.1%)	13 (0.1%)	<10	<10	<10
HELLP Syndrome	308 (0.1%)	57 (<0.1%)	0.019	18 (<0.1%)	0	<10	23 (<0.1%)	7 (<0.1%)	<10	<10	<10
Gestational	9,994 (2.3%)	3,316 (1.7%)	0.043	960 (1.6%)	14-18	282 (0.8%)	1,155 (1.7%)	653 (2.7%)	9-13	113-117	125 (2.3%)
Missing	8,694 (2.0%)	4,144 (2.1%)	0.008	1,432 (2.4%)	22 (1.2%)	596 (1.8%)	1,153 (1.7%)	593 (2.5%)	16 (1.3%)	172 (2.7%)	160 (3.0%)
Previous cesarean delivery	58,535 (13.3%)	31,273 (15.8%)	0.071	8,104 (13.8%)	308 (17.5%)	4,039 (11.9%)	12,748 (19.3%)	3,924 (16.3%)	232 (19.3%)	941 (14.6%)	977 (18.1%)
Placenta previa	2,840 (0.6%)	1,862 (0.9%)	0.033	504 (0.9%)	11 (0.6%)	279 (0.8%)	557 (0.8%)	387 (1.6%)	6 (0.5%)	69 (1.1%)	49 (0.9%)
Placenta accreta spectrum	156 (<0.1%)	74 (<0.1%)	0.001	19 (<0.1%)	<10	18 (0.1%)	22 (<0.1%)	8 (<0.1%)	0	0	<10
Placental abruption	1,085 (0.2%)	402 (0.2%)	0.009	123 (0.2%)	4-8	51 (0.1%)	114 (0.2%)	71 (0.3%)	<10	17 (0.3%)	17 (0.3%)
Labour induction	114,686 (26.1%)	40,695 (20.6%)	0.131	12,445 (21.2%)	372 (21.1%)	6,039 (17.8%)	14,614 (22.1%)	4,447 (18.5%)	247 (20.5%)	1,414 (22.0%)	1,117 (20.7%)
Labour augmentation	129,609 (29.5%)	70,167 (35.5%)	0.128	20,862 (35.5%)	623 (35.4%)	12,789 (37.6%)	23,034 (34.9%)	8,472 (35.2%)	370 (30.7%)	2,229 (34.7%)	1,788 (33.1%)
Episiotomy											
Mediolateral	33,037 (7.5%)	24,008 (12.1%)	0.156	7,035 (12.0%)	152 (8.6%)	5,230 (15.4%)	7,702 (11.7%)	2,617 (10.9%)	104 (8.6%)	657 (10.2%)	511 (9.4%)
Midline	4,206 (1.0%)	3,187 (1.6%)	0.058	841 (1.4%)	18 (1.0%)	1,182 (3.5%)	727 (1.1%)	264 (1.1%)	11 (0.9%)	69 (1.1%)	75 (1.4%)
Unknown	57,511 (13.1%)	24,827 (12.6%)	0.016	7,355 (12.5%)	208 (11.8%)	4,103 (12.1%)	7,865 (11.9%)	3,507 (14.6%)	195 (16.2%)	806 (12.5%)	788 (14.6%)
Fetal presentation											
Cephalic	399,716 (90.9%)	177,151 (89.6%)	0.046	52,353 (89.0%)	1,619 (91.9%)	30,804 (90.6%)	59,462 (90.1%)	21,397 (88.8%)	1,063 (88.2%)	5,639 (87.7%)	4,814 (89.0%)
Breech	16,515 (3.8%)	6,326 (3.2%)	0.030	1,849 (3.1%)	46-50	970 (2.9%)	2,195 (3.3%)	842 (3.5%)	53-57	202 (3.1%)	165 (3.1%)
Transverse	2,537 (0.6%)	1,075 (0.5%)	0.004	248 (0.4%)	10-14	114 (0.3%)	432 (0.7%)	194 (0.8%)	<10	30 (0.5%)	42 (0.8%)
Unknown	20,790 (4.7%)	13,201 (6.7%)	0.084	4,355 (7.4%)	82 (4.7%)	2,120 (6.2%)	3,948 (6.0%)	1,667 (6.9%)	84 (7.0%)	557 (8.7%)	388 (7.2%)
Mode of delivery											
Spontaneous vaginal delivery	278,101 (63.3%)	116,241 (58.8%)	0.092	35,571 (60.5%)	1,047 (59.5%)	22,302 (65.6%)	36,885 (55.9%)	12,826 (53.2%)	663 (55.0%)	3,813 (59.3%)	3,134 (57.9%)
Forceps delivery	10,300 (2.3%)	4,839 (2.4%)	0.007	1,507 (2.6%)	33 (1.9%)	900 (2.6%)	1,415 (2.1%)	679 (2.8%)	25 (2.1%)	149 (2.3%)	131 (2.4%)
Vacuum delivery	26,421 (6.0%)	17,378 (8.8%)	0.106	5,060 (8.6%)	135 (7.7%)	2,928 (8.6%)	6,066 (9.2%)	2,136 (8.9%)	73 (6.1%)	543 (8.4%)	437 (8.1%)
Forceps and vacuum delivery	1,199 (0.3%)	651 (0.3%)	0.010	186 (0.3%)	12 (0.7%)	55 (0.2%)	325 (0.5%)	42 (0.2%)	<10	17-21	<10
Operative vaginal delivery (unknown instrument)	26 (<0.1%)	11 (<0.1%)	0.000	<10	0	<10	<10	<10	0	0	0
CD without labour	66,507 (15.1%)	30,226 (15.3%)	0.004	8,212 (14.0%)	289 (16.4%)	4,399 (12.9%)	11,215 (17.0%)	4,050 (16.8%)	257 (21.3%)	902 (14.0%)	902 (16.7%)
First stage CD	42,460 (9.7%)	21,571 (10.9%)	0.041	6,224 (10.6%)	180 (10.2%)	2,634 (7.7%)	7,794 (11.8%)	3,231 (13.4%)	141 (11.7%)	787 (12.2%)	580 (10.7%)
Second stage CD	10,873 (2.5%)	4,722 (2.4%)	0.006	1,379 (2.3%)	48 (2.7%)	545 (1.6%)	1,615 (2.4%)	815 (3.4%)	33 (2.7%)	149 (2.3%)	138 (2.6%)
CD (missing/unknown stage)	3,619 (0.8%)	2,073 (1.0%)	0.023	656 (1.1%)	17 (1.0%)	238 (0.7%)	700 (1.1%)	311 (1.3%)	8-12	63 (1.0%)	76-80
Perimortem CD	52 (<0.1%)	41 (<0.1%)	0.007	<10	0	<10	17-21	<10	0	<10	<10
Obstetric trauma	16,948 (3.9%)	10,856 (5.5%)	0.077	3,037 (5.2%)	74 (4.2%)	1,725 (5.1%)	4,106 (6.2%)	1,308 (5.4%)	51 (4.2%)	307 (4.8%)	248 (4.6%)
Infant Characteristics											
Gestational age (weeks)											
Preterm (<37 weeks)	27,111 (6.2%)	13,866 (7.0%)	0.034	4,191 (7.1%)	102 (5.8%)	1,668 (4.9%)	4,765 (7.2%)	2,102 (8.7%)	56-60	581 (9.0%)	398-402
Term (37–41 weeks)	410,642 (93.4%)	183,627 (92.9%)	0.022	54,532 (92.7%)	1,653 (93.9%)	32,290 (94.9%)	61,197 (92.7%)	21,966 (91.1%)	1,144 (94.9%)	5,839 (90.8%)	5,006 (92.5%)
Post-term (42 + weeks)	1,805 (0.4%)	260 (0.1%)	0.054	82 (0.1%)	6 (0.3%)	50 (0.1%)	75 (0.1%)	32 (0.1%)	<10	8 (0.1%)	<10
Infant birth weight (g) infant #1											
< 3000	78,866 (17.9%)	60,885 (30.8%)	0.303	18,520 (31.5%)	327 (18.6%)	8,073 (23.7%)	21,787 (33.0%)	7,991 (33.2%)	230 (19.1%)	2,371 (36.9%)	1,586 (29.3%)
3000-3999	305,030 (69.4%)	125,938 (63.7%)	0.121	37,156 (63.2%)	1,221 (69.3%)	23,728 (69.8%)	40,893 (61.9%)	14,844 (61.6%)	859 (71.3%)	3,774 (58.7%)	3,463 (64.0%)
4000-4499	45,001 (10.2%)	8,235 (4.2%)	0.237	2,347 (4.0%)	160 (9.1%)	1,657 (4.9%)	2,596 (3.9%)	897 (3.7%)	86 (7.1%)	208 (3.2%)	284 (5.3%)
≥ 4500	7,531 (1.7%)	1,055 (0.5%)	0.112	272 (0.5%)	38 (2.2%)	181 (0.5%)	332 (0.5%)	150 (0.6%)	19 (1.6%)	24 (0.4%)	39 (0.7%)
Missing	3,130 (0.7%)	1,640 (0.8%)	0.013	510 (0.9%)	15 (0.9%)	369 (1.1%)	429 (0.6%)	218 (0.9%)	11 (0.9%)	51 (0.8%)	37 (0.7%)
Infant head circumference at birth (cm) – infant #1											
< 33	22,128 (5.0%)	14,162 (7.2%)	0.089	4,124 (7.0%)	83 (4.7%)	1,853 (5.4%)	5,162 (7.8%)	1,868 (7.8%)	38 (3.2%)	638 (9.9%)	396 (7.3%)
33-34	90,427 (20.6%)	44,780 (22.6%)	0.050	12,444 (21.2%)	299 (17.0%)	7,907 (23.3%)	15,799 (23.9%)	5,366 (22.3%)	223 (18.5%)	1,521 (23.7%)	1,221 (22.6%)
35-36	88,386 (20.1%)	25,763 (13.0%)	0.191	7,209 (12.3%)	291 (16.5%)	4,639 (13.6%)	9,012 (13.6%)	2,845 (11.8%)	188 (15.6%)	797 (12.4%)	782 (14.5%)
≥ 37	17,411 (4.0%)	3,255 (1.6%)	0.141	898 (1.5%)	63 (3.6%)	537 (1.6%)	1,188 (1.8%)	344 (1.4%)	26 (2.2%)	103 (1.6%)	96 (1.8%)
Missing	221,206 (50.3%)	109,793 (55.5%)	0.104	34,130 (58.0%)	1,025 (58.2%)	19,072 (56.1%)	34,876 (52.8%)	13,677 (56.8%)	730 (60.6%)	3,369 (52.4%)	2,914 (53.9%)

CD, cesarean delivery.

### Demographics

Asian individuals were less likely to be < 24 years old (7.0% compared with 14.3% among White) and more likely to live in urban areas (99.3% vs. 89.7%). Rates of body mass index (BMI) >30 kg/m^2^ were higher in White individuals compared with Asian (17.5% vs. 6.6%). Asian individuals had higher rates of gestational diabetes (13.3% vs. 5.5%), episiotomy (22.0% vs. 13.8%), as well as vacuum delivery (14.0% vs. 9.7%). Rates of obstetric trauma (including 3^rd^ and 4^th^ degree perineal lacerations, high vaginal and cervical lacerations, pelvic hematoma, injuries to the pelvic joints and ligaments, and other pelvic injuries) were higher among Asian individuals (8.9% vs. 6.1%; Table 1).

### Demographics among Asian immigrants

Among Asian immigrants, individuals speaking East Asian languages had the lowest rates of pre-existing hypertension (1.6% vs. 3.3% in White, 2.6% in all Asian) and the highest rates of mediolateral episiotomy (15.4% vs. 7.5% in White and 12.1% in all Asian). Those speaking Southeast Asian languages had the highest rates of preeclampsia (5.1% vs. 4.0% in White and 3.0% in all Asian), placenta previa (1.6% vs. 0.6% in White and 0.9% in all Asian), and preterm birth (8.7% vs. 6.2% in White and 7.0% in all Asian; Table 1).

### Rates of PPH and severe PPH

**Fig 2:**
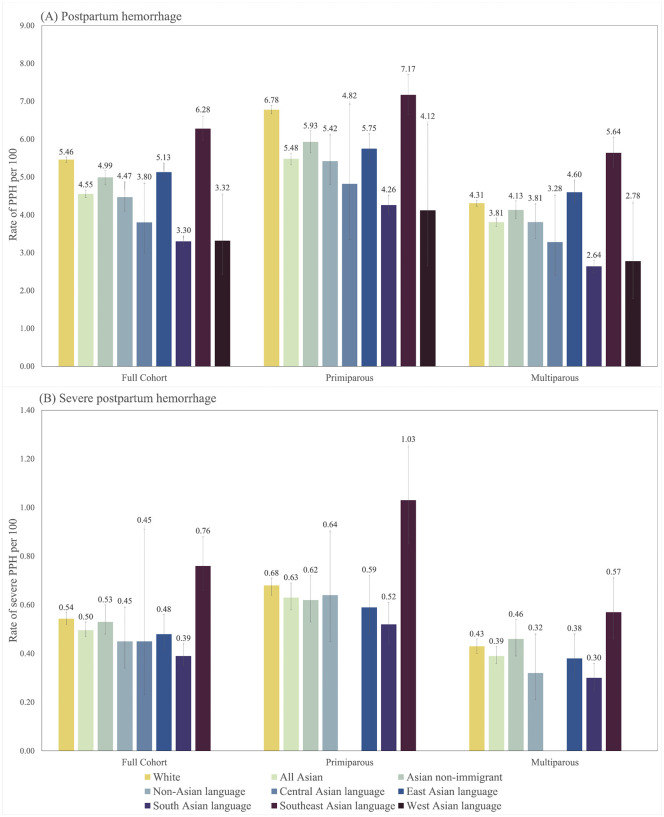
Crude rates and 95% confidence intervals (CIs) of (A) postpartum hemorrhage and (B) severe postpartum hemorrhage by maternal race and Asian immigrant primary language region, Ontario, Canada, 2013-2021.

Among primiparous individuals, PPH occurred in 5.48% of patients who identified as Asian and 6.78% of patients who identified as White (Fig 2). Severe PPH rates were 0.63% in primiparous Asian patients and 0.68% among primiparous White patients. Multiparous and full cohort rates are reported in Fig 2.

Overall, immigrants had lower crude rates of PPH and severe PPH compared with non-immigrants (Supplementary Fig 1). There were no substantial differences observed between immigrant classifications for either PPH or severe PPH. However, it was seen that as duration of residence increased, rates of PPH declined among Asian immigrants speaking languages from South Asia and Southeast Asia (Supplementary Fig 2).

Among primiparous Asian immigrants, those speaking languages from Southeast Asia had the highest crude rates of both PPH and severe PPH (Fig 2); this population also had the highest rates of PPH and severe PPH in the multiparous group.

#### Association between race and PPH (Model 1).

Among primipara, Asian individuals had lower rates of PPH than White individuals in the unadjusted model (RR 0.81, 95% CI 0.78–0.83, p < 0.001) though this association disappeared after adjustment for relevant covariates (aRR 0.99, 95% CI 0.95–1.03). Among multiparous individuals, Asian patients had lower unadjusted rates of PPH compared with White patients (RR 0.88, 95% CI 0.85–0.92, p < 0.001); however, after adjustment, multiparous Asian patients had higher rates of PPH (aRR 1.06, 95% CI 1.01–1.10, p = 0.01).

In the unadjusted analysis, the rate of severe PPH was lower for Asian patients compared with White patients (RR 0.91, 95% CI 0.85–0.98, p = 0.02); after adjustment no association between race and severe PPH was observed (aRR 0.99, 95% CI 0.91–1.09).

#### Association between maternal primary language and PPH (Model 2).

Asian immigrants speaking Southeast Asian languages had higher relative rates of PPH compared with White individuals in both the unadjusted (RR 1.15, 95% CI 1.09–1.21, p < 0.001) and adjusted (aRR 1.41, 95% CI 1.28–1.54, p < 0.001) models. They also experienced the highest rates of severe PPH, with an unadjusted relative rate of 1.40 (95% CI 1.20–1.63, p < 0.001) and an adjusted relative rate of 1.63 (95% CI 1.34–2.00, p < 0.001).

**Fig 3:**
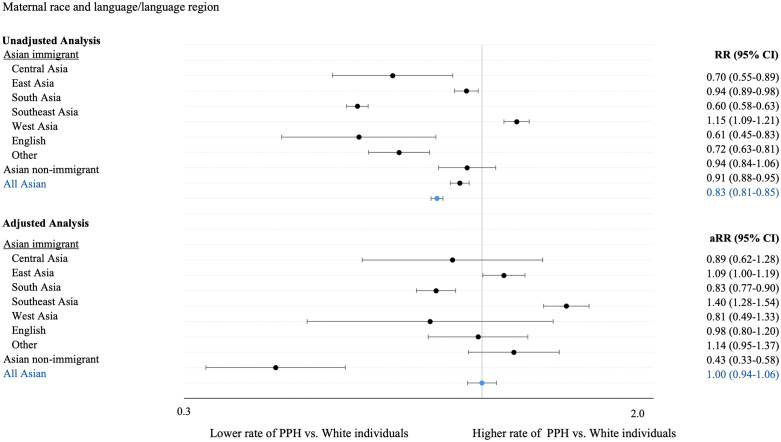
Unadjusted and adjusted relative rates and 95% confidence intervals expressing the relationship between postpartum hemorrhage and Asian immigrant primary language region in Ontario, Canada, 2013-2021. Adjusted models included duration of residence in Canada, age at delivery, multiple gestation, pre-pregnancy body mass index, maternal resources quintile, maternal immigrant status, urban vs. rural residence, tobacco use in pregnancy, drug and substance exposure in pregnancy, type of conception, first trimester prenatal visit, pre-existing diabetes, gestational diabetes, pre-existing hypertension, pregnancy induced hypertension, previous cesarean delivery, labour induction, labour augmentation, fetal presentation, duration of second stage of labour, mode of delivery, and infant birthweight.

Individuals speaking East Asian languages had higher adjusted rates of PPH (aRR 1.09, 95% CI 1.00–1.19, p = 0.04), while those who spoke South Asian languages had a lower adjusted relative rate of PPH compared with White individuals (aRR 0.83, 95% CI 0.77–0.90, p < 0.001; Fig 3).

### Sensitivity analysis

Assessing the distribution of primary language by self-reported race showed that 30.7% of immigrants with Central Asian primary languages identified as White and 59.7% with West Asian primary languages identified as White (S4 Table). In the main analysis limited to individuals who self-identified as Asian, rates of PPH and severe PPH were lower across all categories compared to the unrestricted sensitivity analysis (S5 Table). The most prominent difference was individuals speaking West Asian languages had a 16% higher rate of PPH in the unrestricted analysis compared with the Asian restricted analysis (S5 Table).

The E-values for key associations between maternal race and primary language world region and PPH in all the study strata are provided in S6 Table. E-values for the point estimate and upper 95% confidence bound for PPH among Asian immigrants who spoke Southeast Asian languages were 2.16 and 1.88, respectively, suggesting moderately strong unmeasured confounding would be required to fully explain the observed associations.

## Discussion

This study measured the impact of maternal race and immigration-related factors on PPH and severe PPH among Asian or White individuals in Ontario, Canada and found that, overall, variation exists by race and immigration.

Adjusted analysis showed higher rates of PPH among multiparous Asian individuals compared to their White counterparts, but no significant differences in primiparous individuals or in severe PPH rates. Although White individuals had higher crude PPH and severe PPH rates, this disparity disappeared in adjusted models, possibly accounted for by confounding factors such as obesity, rurality, and infant birthweight. A similar Ontario-based study measuring the impact of maternal race on maternal morbidity found that compared with White race, Asian race was associated with increased risk of gestational diabetes, placental previa, preterm birth, emergency cesarean delivery, and obstetric anal sphincter injury; however, they did not report on PPH or severe PPH [17]. Their findings nonetheless lend support to the presence of racial disparities in obstetric outcomes in Ontario. Furthermore, because our models adjusted for these same maternal and perinatal factors, the higher rates of PPH observed among Asian individuals in our study are unlikely to be driven by the higher prevalence of these conditions.

Immigrants had lower rates of PPH and severe PPH in both Asian and White groups, a pattern sometimes attributed to the “Healthy Immigrant Effect”, whereby newcomers are thought to have better health than non-immigrant populations upon arrival [41]. This has been proposed to result from the self-selection of individuals who are younger and healthier, as well as immigration policies that favour those with fewer health risks [41]. However, immigrants are not a monolith; Southeast Asian language speakers (primarily languages from the Philippines) had the highest crude and adjusted rates of PPH and severe PPH in our study. This is aligned with previous studies showing higher rates of severe maternal morbidity among immigrants to Canada from the Philippines [42]. US studies similarly found that among all Asian groups studied, Filipina patients had highest rates of two contributors to PPH: pregnancy-induced hypertension^,^ and cephalopelvic disproportion [43,44]. Adjustment for maternal age, BMI, and education did not account for the disparities in maternal morbidity in those studies or in ours. These findings highlight the need for further research on disparities among Southeast Asian populations [43,44]. Our finding of a marginally higher adjusted PPH rate among East Asian language–speaking immigrants contrasts with a large U.S. study that found no significant difference in transfusion rates between Chinese, Japanese, and Korean women and White women [15]. This discrepancy may be explained by differences in how East Asian subgroups are defined (language-based vs ethnicity-coded), differences in immigration histories between Canada and the United States, and variation in obstetric practices across health systems. The modest elevation observed in our cohort may reflect system-level factors rather than population differences in risk.

Conversely, South Asian language speakers had the lowest crude and adjusted PPH rates in this study. This contrasts with a United Kingdom (UK) study where South Asian individuals had higher adjusted severe PPH rates than White individuals [45]. This difference is potentially explained by historical differences in immigration practices that cause distributions of the duration of residence and immigrant status to differ in the South Asian populations of Canada and the UK [46,47]. Notably, the UK study included UK-born South Asians, while our study only examined immigrants speaking South Asian languages.

Our findings suggest that the relationship between maternal race, immigration-related factors, and the risk of postpartum hemorrhage is nuanced and cannot be adequately understood through research relying on aggregated racial categories alone. Primary language likely serves as a proxy for several underlying social and structural factors, including cultural background, ethnic identity, and experiences within the healthcare system. Although language and ethnicity may be correlated, they are not interchangeable. In Ontario, the absence of detailed, self-identified race or ethnicity data in administrative health datasets limits our ability to distinguish whether the observed differences reflect ethnic inequities, experiences of systemic or institutional racism, differences in communication and care experiences related to language or accent, or other unmeasured factors. Nevertheless, the variation in PPH rates by maternal primary language among Asian immigrants, particularly the elevated rates observed among those speaking Southeast Asian languages, suggests that health system factors such as differential access to care, communication barriers, or health system bias may contribute to these disparities. A genetic or biological explanation for these differences is not supported by current evidence [48,49]. The concept of distinct genetic risk by racial group has been widely refuted, as genetic variation is far greater within groups than between them, and population categories such as “East Asian” or “Southeast Asian” do not map onto meaningful biological boundaries.

Our findings also highlight that immigrant populations are not homogenous, and that duration of residence may further shape risk. Future research should focus on disaggregating Asian populations more consistently, incorporating immigration history and language and exploring potential mechanisms underlying subgroup disparities.

### Limitations

Our population was limited to the ~ 70% of Ontario pregnancies who accessed prenatal genetic screening [50], a group more likely to be urban, high-income, and under obstetric care [51]. While race was self-reported, our use of primary language has not been validated and should be interpreted cautiously. However, a benefit of this method is that the impact of immigration and primary language could be considered primarily, alongside the more commonly studied race/ethnicity. While mother tongue can be an important proxy, it does not necessarily indicate that an individual has experienced linguistic discrimination. As such, this study was intended as an exploratory examination to begin unpacking these complex dynamics and to inform future, more targeted research. We lacked data on individual income and deprivation measures and approximated these using neighborhood-level indicators. We also lacked information on several variables known to influence rates of PPH including coagulopathy, uterine fibroids, prior PPH, history of APH, anemia, and infection/chorioamnionitis. Additionally, because objective measures of blood loss are not available in the included administrative datasets, some misclassification of PPH is possible. However, our inclusion of severe PPH provides a more objective outcome that is less vulnerable to measurement error than estimated blood loss alone, even acknowledging the limitations of current blood-loss quantification methods [52]. Lastly, statistical power constraints prevented adjusted models for severe PPH by parity or maternal language world regions.

## Conclusion

This study highlights disparities in postpartum hemorrhage and severe postpartum hemorrhage rates across racial, immigration, and language groups in Ontario, Canada. Across most comparisons, differences in risk were limited in magnitude, although heterogeneity was observed across subgroups. The findings underscore the importance of considering nuanced factors such as primary language and immigration patterns in maternal health research. The higher adjusted risk of postpartum hemorrhage among Southeast Asian language speakers specifically warrants further investigation, recognizing that these associations may be influenced by factors not captured in administrative data. Future research addressing limitations in socioeconomic and immigration data, as well as subgroup analyses, are needed to clarify these patterns and inform efforts to improve equity in maternal health.

## Supporting information

S1 Table Descriptions of data sources linked for analysis. (DOCX)

S2 Table Maternal mother tongue language mapping to world region. (DOCX)

S3 Table Calculated minimum detectable effect sizes for each population group compared with White individuals. (DOCX)

S4 Table Distribution of mother tongue world regions and self-reported race. (DOCX)

S5 Table Crude rates (95% confidence intervals) of PPH and severe PPH by maternal mother tongue world region, unrestricted by Asian self-reported race, Ontario, Canada, 2013–2021. (DOCX)

S6 Table E-values expressing the required rate ratio (RR) for any unmeasured confounder to overcome the observed association of maternal race and mother tongue world region and PPH (postpartum hemorrhage) in this study. (DOCX)

S1 Fig Crude rates of (A) PPH and (B) severe PPH by maternal race/ethnicity and maternal immigrant/refugee status. (TIF)

S2 Fig Predicted probabilities of PPH by duration of residence and race. (JPG)

S1 File Model 1 full model results. (DOCX)

S2 File Model 2 full model results. (DOCX)

S3 File Project code. (DOCX)

S4 File Data creation plan. (DOCX)
