# Supplementary material for: Asian-White racial disparities in postpartum hemorrhage and severe postpartum hemorrhage in Ontario, Canada: A population-based cohort study
Source: PLoS One. 2026 Mar 12;21(3):e0344365. doi: 10.1371/journal.pone.0344365 (PMC12981453; doi:10.1371/journal.pone.0344365)
Supplement: S1 Table — (DOCX) [file pone.0344365.s001.docx]

**S1 Table. Descriptions of data sources linked for analysis.**

| **Data Source** | **Description** |
| --- | --- |
| Better Outcomes Registry & Network (BORN) | Ontario birth registry (captures ~100% of maternal-infant records for in-hospital and home deliveries in Ontario), which includes maternal characteristics, diseases, pregnancy, delivery and neonatal complications, and birth characteristics. |
| Prenatal Screening Ontario (PSO) Program | Captures ~70% of deliveries in Ontario. As prenatal screening risk algorithms include adjustment for race, the PSO program, which is administered within the BORN Ontario registry, routinely collects maternal racial/ethnic information. |
| Canadian Institute for Health Information Discharge Abstract Database (CIHI-DAD) | Captures clinical information regarding hospital admissions from all acute care institutions, including diagnoses using ICD-10-CA codes, procedures using CCI codes and discharge disposition. |
| Registered Persons Database (RPDB) Demographic repository | Contains information on all Ontario residents eligible for publicly funded health care in the province to obtain demographic information regarding neighbourhood income quintiles, region of residence and death. |
| Canadian Census and Immigration, Refugees and Citizenship Canada (IRCC) Permanent Residents Data | Contains information on immigration and maternal country of birth. It is considered virtually complete and of high accuracy in most fields because of its administrative and legal use. Less than 1% of records in the database contain missing values. |
| Ontario Marginalization Index (ON-MARG) | Quantifies the level of marginalization occurring in Ontario. This multifaceted index uses data from Statistics Canada’s Census and consists of four dimensions that indicate marginalization: residential instability, material deprivation, dependency and ethnic concentration. Scores corresponding to each of these four dimensions were previously divided into quintiles, where quintile 1 represents areas that are the least marginalized and quintile 5 represents the most marginalized areas. |
| ICES disease cohorts: Ontario Hypertension dataset (Apr 1991-Mar 2020); Ontario Diabetes Dataset (Apr 1994-Mar 2020) | Contains information on presence of pre-existing hypertension and diabetes. |
