# Supplementary material for: Asian-White racial disparities in postpartum hemorrhage and severe postpartum hemorrhage in Ontario, Canada: A population-based cohort study
Source: PLoS One. 2026 Mar 12;21(3):e0344365. doi: 10.1371/journal.pone.0344365 (PMC12981453; doi:10.1371/journal.pone.0344365)
Supplement: S2 Table — (DOCX) [file pone.0344365.s002.docx]

**S2 Table. Maternal mother tongue language mapping to world region**

| **World region** | **Reported primary language** | **Percent of group** |
| --- | --- | --- |
| Central Asia (N=1,761) | Afghan | 1.48 |
|  | Dari | 94.66 |
|  | Kandahari | ≤0.40 |
|  | Kazakh | ≤0.40 |
|  | Tajiki | 0.68 |
|  | Turkmen | ≤0.40 |
|  | Uzbek | 2.50 |
| East Asia (N=34,008) | Cantonese | 19.91 |
|  | Changle | ≤0.01 |
|  | Chaocho | ≤0.01 |
|  | Chinese | 24.44 |
|  | Chiuchow | ≤0.01 |
|  | Chowchau | ≤0.01 |
|  | Enping | ≤0.01 |
|  | Foochow | 0.14 |
|  | Fujian | 0.15 |
|  | Fukien | 0.09 |
|  | Fuqing | 0.07 |
|  | Hakka | 0.19 |
|  | Japanese | 2.90 |
|  | Kaiping | ≤0.01 |
|  | Korean | 8.17 |
|  | Mandarin | 42.26 |
|  | Mongolian | 0.06 |
|  | Ouighour | 0.02 |
|  | Shanghainese | 0.03 |
|  | Sichuan/Szechuan | ≤0.01 |
|  | Taishanese | 0.12 |
|  | Teochew | ≤0.01 |
|  | Tibetan | 1.38 |
|  | Toishan | ≤0.01 |
| South Asia (N=66,037) | Balochi | 0.01 |
|  | Bengali | 5.85 |
|  | Dogri | <0.01 |
|  | Gujarati | 8.66 |
|  | Hindi | 8.71 |
|  | Hindko | 0.04 |
|  | Kacchi | 0.03 |
|  | Kanarese | <0.01 |
|  | Kankani | 0.03 |
|  | Kannada | 0.47 |
|  | Kashmiri | 0.05 |
|  | Konkani | 0.29 |
|  | Malayalam | 3.73 |
|  | Marathi | 0.99 |
|  | Mizo | <0.01 |
|  | Nepali | 1.42 |
|  | Oriya | 0.19 |
|  | Pahari | <0.01 |
|  | Pashto | 1.34 |
|  | Punjabi | 23.70 |
|  | Saraiki | 0.02 |
|  | Sindhi | 0.12 |
|  | Sinhalese | 0.99 |
|  | Tamil | 19.86 |
|  | Telugu | 2.77 |
|  | Urdu | 20.71 |
| Southeast Asia (N=24,100) | Aklanon | 0.08 |
|  | Bicol | 0.24 |
|  | Bisaya | 0.18 |
|  | Bontok | ≤0.02 |
|  | Burmese | 1.19 |
|  | Cambodian | 0.37 |
|  | Capizeno | ≤0.02 |
|  | Cebuano | 1.24 |
|  | Chavacano | ≤0.02 |
|  | Hiligaynon | 0.68 |
|  | Igorot | 0.19 |
|  | Iiongo | 0.11 |
|  | Ilican | 0.16 |
|  | Ilocano | 2.67 |
|  | Indonesian | 1.36 |
|  | Javanese | ≤0.02 |
|  | Khmer | 2.68 |
|  | Kinaraya | 0.03 |
|  | Laotian | 0.49 |
|  | Malay | 0.22 |
|  | Pampango | 0.39 |
|  | Pangasinan | 0.22 |
|  | Shan | ≤0.02 |
|  | Tagalog | 69.20 |
|  | Thai | 0.93 |
|  | Vietnamese | 16.05 |
|  | Visayan | 1.12 |
|  | Waray | 0.15 |
| West Asia (N=1,205) | Arabic Iraq | 1.66 |
|  | Arabic Lebanon | 1.16 |
|  | Arabic Syria | 5.06 |
|  | Arabic Yemen | 0.50 |
|  | Armenian | 1.08 |
|  | Assyrian | 6.31 |
|  | Azeri | 0.75 |
|  | Chaldean | 3.24 |
|  | Farsi | 34.52 |
|  | Georgian | ≤0.41 |
|  | Hebrew | 0.50 |
|  | Kurdish | 7.47 |
|  | Lebanese | ≤0.41 |
|  | Persian | 28.63 |
|  | Turkish | 8.63 |
