# Supplementary material for: Asian-White racial disparities in postpartum hemorrhage and severe postpartum hemorrhage in Ontario, Canada: A population-based cohort study
Source: PLoS One. 2026 Mar 12;21(3):e0344365. doi: 10.1371/journal.pone.0344365 (PMC12981453; doi:10.1371/journal.pone.0344365)
Supplement: S3 Table — (DOCX) [file pone.0344365.s003.docx]

**S3 Table. Calculated minimum detectable effect sizes for each population group compared with White individuals.**

| **Population group** | **N** | **Smallest RR that can be detected with 80% power** |
| --- | --- | --- |
| White | 439,558 | (reference) |
| Asian | 197,753 | 1.04 |
| Not an immigrant | 58,805 | 1.06 |
| Non-Asian language | 11,837 | 1.11 |
| Central Asian language | 1,761 | 1.28 |
| East Asian language | 34,008 | 1.07 |
| South Asian language | 66,037 | 1.05 |
| Southeast Asian language | 24,100 | 1.08 |
| West Asian language | 1,205 | 1.35 |
