# Supplementary material for: Asian-White racial disparities in postpartum hemorrhage and severe postpartum hemorrhage in Ontario, Canada: A population-based cohort study
Source: PLoS One. 2026 Mar 12;21(3):e0344365. doi: 10.1371/journal.pone.0344365 (PMC12981453; doi:10.1371/journal.pone.0344365)
Supplement: S4 Table — (DOCX) [file pone.0344365.s004.docx]

**S4 Table. Distribution of primary language world regions and self-reported race**

|  | **Primary language world region** | | | | |
| --- | --- | --- | --- | --- | --- |
| **Self-reported race** | **Central Asia** | **East Asia** | **South Asia** | **Southeast Asia** | **West Asia** |
|  | **N=5,031** | **N=39,456** | **N=86,527** | **N=30,968** | **N=14,370** |
| Asian | 35.00% | 86.19% | 76.32% | 77.82% | 8.39% |
| Black | <0.12%* | 0.04% | 0.11% | 0.10% | 0.09% |
| White | 30.73% | 0.26% | 1.18% | 0.77% | 59.73% |
| Other | 6.44%-6.52%* | 0.63% | 1.93% | 1.47% | 6.70% |
| Unknown | 27.73% | 12.88% | 20.46% | 19.84% | 25.09% |

*Suppression was required due to small counts in the Black subgroup, which necessitated presenting the “Other” category as a range to prevent back-calculation.
