# Supplementary material for: Asian-White racial disparities in postpartum hemorrhage and severe postpartum hemorrhage in Ontario, Canada: A population-based cohort study
Source: PLoS One. 2026 Mar 12;21(3):e0344365. doi: 10.1371/journal.pone.0344365 (PMC12981453; doi:10.1371/journal.pone.0344365)
Supplement: S5 Table — (DOCX) [file pone.0344365.s005.docx]

**S5 Table. Crude rates (95% confidence intervals) of PPH and severe PPH by world region of maternal primary language, unrestricted by Asian self-reported race, Ontario, Canada, 2013-2021**

| **Immigrant status and world region of maternal primary language** | **Crude Rates of PPH per 100 (95% CI)** | | | **Crude Rates of Severe PPH per 100 (95% CI)** | | |
| --- | --- | --- | --- | --- | --- | --- |
|  | **Full Cohort** | **Nulliparous** | **Parous** | **Full Cohort** | **Nulliparous** | **Parous** |
| Not an immigrant | 5.80 (5.75-5.85) | 7.05 (6.96-7.14) | 4.83 (4.76-4.89) | 0.61 (0.59-0.63) | 0.74 (0.71-0.77) | 0.51 (0.49-0.53) |
| Non-Asian language | 4.54 (4.43-4.66) | 5.56 (5.35-5.77) | 3.97 (3.84-4.10) | 0.55 (0.51-0.59) | 0.68 (0.61-0.75) | 0.48 (0.43-0.53) |
| Central Asia | 4.15 (3.63-4.76) | 4.66 (3.74-5.80) | 3.89 (3.27-4.62) | 0.60 (0.42-0.85) | 0.64 (0.36-1.16) | 0.57 (0.37-0.90) |
| East Asia | 5.30 (5.08-5.53) | 6.02 (5.67-6.38) | 4.69 (4.41-4.99) | 0.50 (0.43-0.57) | 0.61 (0.51-0.74) | 0.40 (0.33-0.50) |
| South Asia | 3.47 (3.35-3.60) | 4.49 (4.27-4.71) | 2.78 (2.64-2.92) | 0.42 (0.38-0.46) | 0.54 (0.47-0.62) | 0.34 (0.29-0.39) |
| Southeast Asia | 6.46 (6.18-6.75) | 7.42 (6.96-7.90) | 5.78 (5.44-6.14) | 0.77 (0.68-0.88) | 1.01 (0.85-1.21) | 0.61 (0.50-0.73) |
| West Asia | 3.97 (3.66-4.31) | 4.91 (4.38-5.51) | 3.30 (2.94-3.72) | 0.40 (0.31-0.51) | 0.47 (0.32-0.68) | 0.35 (0.24-0.50) |

PPH, postpartum hemorrhage; CI, confidence interval
