# Supplementary material for: Asian-White racial disparities in postpartum hemorrhage and severe postpartum hemorrhage in Ontario, Canada: A population-based cohort study
Source: PLoS One. 2026 Mar 12;21(3):e0344365. doi: 10.1371/journal.pone.0344365 (PMC12981453; doi:10.1371/journal.pone.0344365)
Supplement: S6 Table — (DOCX) [file pone.0344365.s006.docx]

**S6 Table. E-values expressing the required rate ratio (RR) for any unmeasured confounder to overcome the observed association of maternal race and mother tongue world region and PPH (postpartum hemorrhage) in this study**

|  | Full Cohort | |  | Primiparous | |  | Parous | |
| --- | --- | --- | --- | --- | --- | --- | --- | --- |
|  | E-value | |  | E-value | |  | E-value | |
|  | Point estimate (RR) | Upper confidence bound |  | Point estimate (RR) | Upper confidence bound |  | Point estimate (RR) | Upper confidence bound |
| Asian | 1.03 | 1.00 |  | 1.11 | 1.00 |  | 1.30 | 1.11 |
| Not an immigrant | 4.03 | 2.87 |  |  |  |  |  |  |
| Non-Asian language | 1.53 | 1.00 |  |  |  |  |  |  |
| Central Asia | 1.51 | 1.00 |  |  |  |  |  |  |
| East Asia | 1.41 | 1.06 |  |  |  |  |  |  |
| South Asia | 1.70 | 1.47 |  |  |  |  |  |  |
| Southeast Asia | 2.16 | 1.88 |  |  |  |  |  |  |
| West Asia | 1.77 | 1.00 |  |  |  |  |  |  |

The E-value of the point estimate is the minimum strength of association on the rate ratio scale that an unmeasured confounder would need to have with both the exposure and the outcome, conditional on the measured covariates, to fully explain away a specific exposure-outcome association.

The E-value of the 95% upper confidence bound expresses the extent of unmeasured confounding that would be required to shift the confidence interval so that it includes a rate ratio of 1.00 (i.e., no association).
