# Supplementary material for: Asian-White racial disparities in postpartum hemorrhage and severe postpartum hemorrhage in Ontario, Canada: A population-based cohort study
Source: PLoS One. 2026 Mar 12;21(3):e0344365. doi: 10.1371/journal.pone.0344365 (PMC12981453; doi:10.1371/journal.pone.0344365)
Supplement: S1 File — (DOCX) [file pone.0344365.s009.docx]

**S1 File. Model 1 full model results.**

| **Variable Label** | **Variable Value** | **Unadjusted (Univariate)** | | | | **Adjusted (Multivariate)** | | | |
| --- | --- | --- | --- | --- | --- | --- | --- | --- | --- |
|  |  | **Risk Ratio** | **Lower** | **Upper** | **P-value** | **Risk Ratio** | **Lower** | **Upper** | **P-value** |
| Maternal race/ethnicity | White (REF) | 1 | - | - | - | 1 | - | - | - |
|  | Asian (all) | 0.8338 | 0.8139 | 0.8541 | <.0001 | 0.9993 | 0.9428 | 1.0591 | 0.9802 |
| Maternal duration of residence in Canada (years) - full cohort (continuous) | | 1.0057 | 1.0047 | 1.0066 | <.0001 | 0.9919 | 0.9892 | 0.9946 | <.0001 |
| Maternal race/ethnicity*maternal duration of residence in Canada (years) - full cohort (continuous) | White (REF) | N/A | N/A | N/A | N/A | 1 | - | - | - |
|  | Asian (all) | N/A | N/A | N/A | N/A | 1.0021 | 0.9999 | 1.0043 | 0.0616 |
| Maternal age | <20 | 1.0980 | 0.9933 | 1.2137 | 0.0675 | 0.9830 | 0.8890 | 1.0869 | 0.7381 |
|  | 20-24 (REF) | 1 | - | - | - | 1 | - | - | - |
|  | 25-29 | 0.9267 | 0.8880 | 0.9671 | 0.0005 | 0.9983 | 0.9545 | 1.0441 | 0.9417 |
|  | 30-34 | 0.8659 | 0.8310 | 0.9023 | <.0001 | 1.0180 | 0.9703 | 1.0680 | 0.4664 |
|  | 35-39 | 0.8395 | 0.8033 | 0.8774 | <.0001 | 1.0756 | 1.0175 | 1.1370 | 0.0101 |
|  | 40+ | 0.9160 | 0.8582 | 0.9777 | 0.0084 | 1.2035 | 1.1140 | 1.3003 | <.0001 |
| Parity (ref=parous) | Nulliparous | 1.5411 | 1.5090 | 1.5739 | <.0001 | 1.2973 | 1.2611 | 1.3346 | <.0001 |
|  | Parous (REF) | 1 | - | - | - | 1 | - | - | - |
|  | Missing/Unknown | 1.3560 | 1.2205 | 1.5066 | <.0001 | 1.1734 | 1.0515 | 1.3094 | 0.0043 |
| Plurality | Yes | 2.0675 | 1.5115 | 2.8280 | <.0001 | 2.3147 | 1.6869 | 3.1763 | <.0001 |
|  | No (REF) | 1 | - | - | - | 1 | - | - | - |
| Pre-pregnancy body mass index | <18.5 | 0.8877 | 0.8398 | 0.9384 | <.0001 | 0.9311 | 0.8808 | 0.9843 | 0.0118 |
|  | 18.5-24.9 (REF) | 1 | - | - | - | 1 | - | - | - |
|  | 25-29.9 | 1.0194 | 0.9913 | 1.0483 | 0.1786 | 1.0065 | 0.9786 | 1.0352 | 0.6523 |
|  | 30-34.9 | 1.0573 | 1.0176 | 1.0985 | 0.0043 | 1.0221 | 0.9832 | 1.0625 | 0.2694 |
|  | 35-39.9 | 1.0509 | 0.9939 | 1.1112 | 0.0808 | 0.9848 | 0.9304 | 1.0423 | 0.5961 |
|  | >=40 | 1.1545 | 1.0856 | 1.2277 | <.0001 | 1.0774 | 1.0124 | 1.1467 | 0.0189 |
|  | Unknown | 0.8919 | 0.8626 | 0.9223 | <.0001 | 0.8833 | 0.8528 | 0.9148 | <.0001 |
| Age and labour force quintile (previously dependency quintile) | Q1 (REF) | 1 | - | - | - | 1 | - | - | - |
|  | Q2 | 1.0455 | 1.0146 | 1.0772 | 0.0036 | 1.0149 | 0.9849 | 1.0459 | 0.3340 |
|  | Q3 | 1.1422 | 1.1067 | 1.1788 | <.0001 | 1.0899 | 1.0553 | 1.1256 | <.0001 |
|  | Q4 | 1.1642 | 1.1268 | 1.2029 | <.0001 | 1.0955 | 1.0593 | 1.1329 | <.0001 |
|  | Q5 | 1.2095 | 1.1686 | 1.2519 | <.0001 | 1.0820 | 1.0434 | 1.1220 | <.0001 |
|  | Missing | 1.4009 | 1.1923 | 1.6459 | <.0001 | 1.3990 | 1.1568 | 1.6919 | 0.0005 |
| Material resources quintile (previously deprivation quintile) | Q1 (REF) | 1 | - | - | - | 1 | - | - | - |
|  | Q2 | 0.9299 | 0.9007 | 0.9600 | <.0001 | 0.9433 | 0.9138 | 0.9739 | 0.0003 |
|  | Q3 | 0.9388 | 0.9086 | 0.9701 | 0.0002 | 0.9463 | 0.9154 | 0.9782 | 0.0011 |
|  | Q4 | 0.9512 | 0.9199 | 0.9835 | 0.0034 | 0.9572 | 0.9245 | 0.9911 | 0.0137 |
|  | Q5 | 0.9616 | 0.9300 | 0.9942 | 0.0215 | 0.9964 | 0.9603 | 1.0339 | 0.8482 |
|  | Missing | 1.2385 | 1.0537 | 1.4556 | 0.0095 | N/A | N/A | N/A | N/A |
| Households and dwellings quintile (previously instability quintile) | Q1 (REF) | 1 | - | - | - | 1 | - | - | - |
|  | Q2 | 1.1544 | 1.1155 | 1.1946 | <.0001 | 1.0566 | 1.0204 | 1.0941 | 0.0020 |
|  | Q3 | 1.2265 | 1.1853 | 1.2690 | <.0001 | 1.0985 | 1.0606 | 1.1377 | <.0001 |
|  | Q4 | 1.2368 | 1.1952 | 1.2800 | <.0001 | 1.1117 | 1.0719 | 1.1530 | <.0001 |
|  | Q5 | 1.2391 | 1.1995 | 1.2799 | <.0001 | 1.1148 | 1.0765 | 1.1544 | <.0001 |
|  | Missing | 1.5072 | 1.2819 | 1.7720 | <.0001 | N/A | N/A | N/A | N/A |
| Maternal immigrant/refugee status | Not an immigrant (REF) | 1 | - | - | - | 1 | - | - | - |
|  | Economic | 0.8515 | 0.8236 | 0.8803 | <.0001 | 0.7932 | 0.7416 | 0.8484 | <.0001 |
|  | Family | 0.7509 | 0.7254 | 0.7773 | <.0001 | 0.7296 | 0.6803 | 0.7826 | <.0001 |
|  | Resettled refugee & protected person | 0.7136 | 0.6691 | 0.7610 | <.0001 | 0.7117 | 0.6567 | 0.7713 | <.0001 |
|  | Other | 0.7796 | 0.6303 | 0.9642 | 0.0217 | 0.7409 | 0.5946 | 0.9233 | 0.0076 |
| Geographic location | Urban (REF) | 1 | - | - | - | 1 | - | - | - |
|  | Rural | 1.4235 | 1.3742 | 1.4746 | <.0001 | 1.3473 | 1.2975 | 1.3991 | <.0001 |
|  | Missing | 0.9017 | 0.6608 | 1.2306 | 0.5144 | 0.6955 | 0.4853 | 0.9967 | 0.0479 |
| Tobacco use in pregnancy | No (REF) | 1 | - | - | - | 1 | - | - | - |
|  | Yes | 1.0171 | 0.9753 | 1.0606 | 0.4294 | 1.0306 | 0.9860 | 1.0772 | 0.1812 |
|  | Missing | 1.2082 | 1.1481 | 1.2714 | <.0001 | 1.0033 | 0.9334 | 1.0784 | 0.9287 |
| Drug and substance exposure in pregnancy | No (REF) | 1 | - | - | - | 1 | - | - | - |
|  | Yes | 1.0728 | 0.9770 | 1.1781 | 0.1409 | 1.0342 | 0.9396 | 1.1383 | 0.4924 |
|  | Missing | 1.2440 | 1.1857 | 1.3052 | <.0001 | 0.9438 | 0.8810 | 1.0112 | 0.1002 |
| Type of conception | Spontaneous (REF) | 1 | - | - | - | 1 | - | - | - |
|  | Assisted | 1.6002 | 1.5313 | 1.6722 | <.0001 | 1.4535 | 1.3895 | 1.5204 | <.0001 |
|  | Unknown | 1.3514 | 1.2973 | 1.4077 | <.0001 | 1.1928 | 1.1308 | 1.2581 | <.0001 |
| First trimester prenatal visit | No | 0.9037 | 0.8600 | 0.9496 | <.0001 | 0.9766 | 0.9294 | 1.0262 | 0.3484 |
|  | Yes (REF) | 1 | - | - | - | 1 | - | - | - |
|  | Missing | 1.3768 | 1.3313 | 1.4238 | <.0001 | 1.4337 | 1.3730 | 1.4972 | <.0001 |
| Pre-existing diabetes | No (REF) | 1 | - | - | - | 1 | - | - | - |
|  | Yes | 0.9608 | 0.8859 | 1.0420 | 0.3340 | 0.9442 | 0.8704 | 1.0242 | 0.1664 |
| Gestational diabetes | No (REF) | 1 | - | - | - | 1 | - | - | - |
|  | Yes | 0.9577 | 0.9213 | 0.9954 | 0.0283 | 0.9801 | 0.9419 | 1.0199 | 0.3222 |
| Pre-existing hypertension | No (REF) | 1 | - | - | - | 1 | - | - | - |
|  | Yes | 1.2909 | 1.2220 | 1.3636 | <.0001 | 1.1719 | 1.1031 | 1.2450 | <.0001 |
| Pregnancy induced hypertension | No (REF) | 1 | - | - | - | 1 | - | - | - |
|  | Yes | 1.4540 | 1.3957 | 1.5147 | <.0001 | 1.2967 | 1.2383 | 1.3578 | <.0001 |
| Previous cesarean delivery | No (REF) | 1 | - | - | - | 1 | - | - | - |
|  | Yes | 0.5413 | 0.5205 | 0.5628 | <.0001 | 1.0479 | 0.9961 | 1.1024 | 0.0706 |
| Placental previa | No (REF) | 1 | - | - | - | 1 | - | - | - |
|  | Yes | 2.1826 | 2.0127 | 2.3669 | <.0001 | 3.4079 | 3.1125 | 3.7314 | <.0001 |
| Placenta accreta spectrum | No (REF) | 1 | - | - | - | 1 | - | - | - |
|  | Yes | 8.1629 | 7.0121 | 9.5026 | <.0001 | 6.5112 | 5.3869 | 7.8703 | <.0001 |
| Placental abruption | No (REF) | 1 | - | - | - | 1 | - | - | - |
|  | Yes | 1.6905 | 1.4340 | 1.9928 | <.0001 | 1.7137 | 1.4483 | 2.0277 | <.0001 |
| Induction | No (REF) | 1 | - | - | - | 1 | - | - | - |
|  | Yes | 1.5208 | 1.4871 | 1.5552 | <.0001 | 1.2629 | 1.2279 | 1.2990 | <.0001 |
| Augmentation | No (REF) | 1 | - | - | - | 1 | - | - | - |
|  | Yes | 1.0212 | 0.9984 | 1.0446 | 0.0687 | 0.9933 | 0.9661 | 1.0213 | 0.6350 |
| Episiotomy | Mediolateral | 1.4798 | 1.4347 | 1.5263 | <.0001 | 1.0144 | 0.9785 | 1.0515 | 0.4377 |
|  | Midline | 1.0482 | 0.9523 | 1.1538 | 0.3361 | 0.8851 | 0.8042 | 0.9741 | 0.0125 |
|  | None (REF) | 1 | - | - | - | 1 | - | - | - |
|  | Unknown | 0.6713 | 0.6462 | 0.6973 | <.0001 | 0.9460 | 0.9017 | 0.9925 | 0.0235 |
| Fetal presentation | Breech | 0.5072 | 0.4686 | 0.5489 | <.0001 | 0.8548 | 0.7858 | 0.9299 | 0.0003 |
|  | Cephalic (REF) | 1 | - | - | - | 1 | - | - | - |
|  | Transverse | 0.9201 | 0.7959 | 1.0636 | 0.2602 | 1.2336 | 1.0664 | 1.4270 | 0.0047 |
|  | Unknown | 1.0342 | 0.9878 | 1.0827 | 0.1508 | 1.0296 | 0.9771 | 1.0850 | 0.2747 |
| Duration of second stage of labour | <60 minutes (REF) | 1 | - | - | - | 1 | - | - | - |
|  | 60-119 minutes | 1.3250 | 1.2853 | 1.3659 | <.0001 | 1.1398 | 1.1033 | 1.1775 | <.0001 |
|  | 120-179 minutes | 1.5745 | 1.5185 | 1.6325 | <.0001 | 1.2587 | 1.2104 | 1.3091 | <.0001 |
|  | 180-239 minutes | 1.8074 | 1.7274 | 1.8911 | <.0001 | 1.3778 | 1.3127 | 1.4463 | <.0001 |
|  | 240+ minutes | 1.7904 | 1.7094 | 1.8753 | <.0001 | 1.3763 | 1.3093 | 1.4468 | <.0001 |
|  | No second stage - n (%) | 0.7250 | 0.7041 | 0.7466 | <.0001 | 1.0800 | 0.9783 | 1.1923 | 0.1272 |
| Mode of delivery | SVD (REF) | 1 | - | - | - | 1 | - | - | - |
|  | Forceps delivery | 2.1211 | 2.0254 | 2.2213 | <.0001 | 1.5543 | 1.4761 | 1.6366 | <.0001 |
|  | Vacuum delivery | 1.3478 | 1.3002 | 1.3971 | <.0001 | 1.1416 | 1.0987 | 1.1861 | <.0001 |
|  | Forceps and vacuum delivery | 1.7866 | 1.5535 | 2.0547 | <.0001 | 1.3587 | 1.1795 | 1.5652 | <.0001 |
|  | Operative vaginal delivery (forceps/vacuum unknown) | 1.4972 | 0.5060 | 4.4300 | 0.4659 | 1.0461 | 0.3474 | 3.1506 | 0.9361 |
|  | First stage cesarean delivery (CD) | 0.8003 | 0.7698 | 0.8319 | <.0001 | 0.4646 | 0.4148 | 0.5202 | <.0001 |
|  | Second stage CD | 1.1509 | 1.0813 | 1.2249 | <.0001 | 0.6552 | 0.5901 | 0.7274 | <.0001 |
|  | CD without labour | 0.4860 | 0.4666 | 0.5062 | <.0001 | 0.8674 | 0.7890 | 0.9537 | 0.0033 |
|  | CD (missing/unknown stage) OR perimortem CD | 0.7820 | 0.6913 | 0.8846 | <.0001 | 0.6126 | 0.5255 | 0.7142 | <.0001 |
| Gestational age | Preterm (<37 weeks) | 1.1344 | 1.0892 | 1.1814 | <.0001 | 1.3843 | 1.3216 | 1.4499 | <.0001 |
|  | Term (37-41 weeks) (REF) | 1 | - | - | - | 1 | - | - | - |
|  | Post-term (42+ weeks) | 1.2836 | 1.0898 | 1.5119 | 0.0028 | 0.9552 | 0.8116 | 1.1241 | 0.5809 |
| Infant birth weight | <3000g | 0.8020 | 0.7795 | 0.8252 | <.0001 | 0.7260 | 0.7029 | 0.7499 | <.0001 |
|  | 3000-3999g (REF) | 1 | - | - | - | 1 | - | - | - |
|  | 4000-4499g | 1.4489 | 1.4022 | 1.4970 | <.0001 | 1.4819 | 1.4325 | 1.5329 | <.0001 |
|  | 4500g+ | 1.8293 | 1.7101 | 1.9567 | <.0001 | 1.9987 | 1.8655 | 2.1415 | <.0001 |
|  | Missing | 1.2185 | 1.0911 | 1.3607 | 0.0005 | 0.9418 | 0.8429 | 1.0523 | 0.2897 |
| Infant head circumference at birth | <33cm | 0.9526 | 0.9026 | 1.0053 | 0.0773 | 0.9811 | 0.9290 | 1.0361 | 0.4932 |
|  | 33-34cm (REF) | 1 | - | - | - | 1 | - | - | - |
|  | 35-36cm | 1.1561 | 1.1168 | 1.1967 | <.0001 | 1.0361 | 1.0004 | 1.0732 | 0.0476 |
|  | 37cm+ | 1.3262 | 1.2502 | 1.4068 | <.0001 | 1.0141 | 0.9536 | 1.0785 | 0.6553 |
|  | Missing | 1.1767 | 1.1440 | 1.2103 | <.0001 | 1.1180 | 1.0865 | 1.1504 | <.0001 |
