# Supplementary material for: Asian-White racial disparities in postpartum hemorrhage and severe postpartum hemorrhage in Ontario, Canada: A population-based cohort study
Source: PLoS One. 2026 Mar 12;21(3):e0344365. doi: 10.1371/journal.pone.0344365 (PMC12981453; doi:10.1371/journal.pone.0344365)
Supplement: S2 File — (DOCX) [file pone.0344365.s010.docx]

**S2 File. Model 2 full model results.**

| **Variable Label** | **Variable Value** | **Unadjusted (Univariate)** | | | | **Adjusted (Multivariate)** | | | |
| --- | --- | --- | --- | --- | --- | --- | --- | --- | --- |
|  |  | **Risk Ratio** | **Lower** | **Upper** | **P-value** | **Risk Ratio** | **Lower** | **Upper** | **P-value** |
| Maternal race/ethnicity | White (REF) | 1 | - | - | - | 1 | - | - | - |
|  | Asian (not an immigrant) | 0.9135 | 0.8794 | 0.9490 | <.0001 | 0.4344 | 0.3278 | 0.5756 | <.0001 |
|  | Asian (English) | 0.7150 | 0.6322 | 0.8086 | <.0001 | 0.9838 | 0.8043 | 1.2035 | 0.8739 |
|  | Asian (other) | 0.9410 | 0.8384 | 1.0563 | 0.3025 | 1.1370 | 0.9462 | 1.3664 | 0.1708 |
|  | Asian (Central Asia) | 0.6966 | 0.5464 | 0.8881 | 0.0035 | 0.8871 | 0.6161 | 1.2773 | 0.5197 |
|  | Asian (East Asia) | 0.9384 | 0.8944 | 0.9846 | 0.0095 | 1.0922 | 1.0029 | 1.1896 | 0.0428 |
|  | Asian (South Asia) | 0.6042 | 0.5784 | 0.6311 | <.0001 | 0.8300 | 0.7668 | 0.8985 | <.0001 |
|  | Asian (Southeast Asia) | 1.1495 | 1.0920 | 1.2099 | <.0001 | 1.4047 | 1.2815 | 1.5397 | <.0001 |
|  | Asian (West Asia) | 0.6078 | 0.4450 | 0.8301 | 0.0017 | 0.8107 | 0.4932 | 1.3326 | 0.4079 |
| Maternal duration of residence in Canada (years) - full cohort (continuous) | | 1.0057 | 1.0047 | 1.0066 | <.0001 | 0.9902 | 0.9870 | 0.9935 | <.0001 |
| Maternal race/ethnicity*maternal duration of residence in Canada (years) - full cohort (continuous) | White (REF) | N/A | N/A | N/A | N/A | 1 | - | - | - |
|  | Asian (not an immigrant) | N/A | N/A | N/A | N/A | 1.0266 | 1.0179 | 1.0352 | <.0001 |
|  | Asian (English) | N/A | N/A | N/A | N/A | 0.9987 | 0.9850 | 1.0126 | 0.8538 |
|  | Asian (other) | N/A | N/A | N/A | N/A | 1.0094 | 0.9961 | 1.0230 | 0.1668 |
|  | Asian (Central Asia) | N/A | N/A | N/A | N/A | 1.0053 | 0.9718 | 1.0400 | 0.7604 |
|  | Asian (East Asia) | N/A | N/A | N/A | N/A | 1.0101 | 1.0035 | 1.0169 | 0.0029 |
|  | Asian (South Asia) | N/A | N/A | N/A | N/A | 1.0008 | 0.9939 | 1.0077 | 0.8152 |
|  | Asian (Southeast Asia) | N/A | N/A | N/A | N/A | 1.0069 | 1.0000 | 1.0140 | 0.0501 |
|  | Asian (West Asia) | N/A | N/A | N/A | N/A | 0.9999 | 0.9576 | 1.0441 | 0.9952 |
| Maternal age | <20 | 1.0980 | 0.9933 | 1.2137 | 0.0675 | 0.9707 | 0.8775 | 1.0739 | 0.5644 |
|  | 20-24 (REF) | 1 | - | - | - | 1 | - | - | - |
|  | 25-29 | 0.9267 | 0.8880 | 0.9671 | 0.0005 | 1.0068 | 0.9618 | 1.0539 | 0.7710 |
|  | 30-34 | 0.8659 | 0.8310 | 0.9023 | <.0001 | 1.0181 | 0.9683 | 1.0705 | 0.4832 |
|  | 35-39 | 0.8395 | 0.8033 | 0.8774 | <.0001 | 1.0535 | 0.9925 | 1.1182 | 0.0867 |
|  | 40+ | 0.9160 | 0.8582 | 0.9777 | 0.0084 | 1.1492 | 1.0587 | 1.2474 | 0.0009 |
| Parity | Nulliparous | 1.5411 | 1.5090 | 1.5739 | <.0001 | 1.2985 | 1.2623 | 1.3357 | <.0001 |
|  | Parous (REF) | 1 | - | - | - | 1 | - | - | - |
|  | Missing/Unknown | 1.3560 | 1.2205 | 1.5066 | <.0001 | 1.1653 | 1.0441 | 1.3005 | 0.0063 |
| Plurality | Yes | 2.0675 | 1.5115 | 2.8280 | <.0001 | 2.3229 | 1.6951 | 3.1832 | <.0001 |
|  | No (REF) | 1 | - | - | - | 1 | - | - | - |
| Pre-pregnancy body mass index (kg/m^2) | <18.5 | 0.8877 | 0.8398 | 0.9384 | <.0001 | 0.9227 | 0.8726 | 0.9755 | 0.0046 |
|  | 18.5-24.9 (REF) | 1 | - | - | - | 1 | - | - | - |
|  | 25-29.9 | 1.0194 | 0.9913 | 1.0483 | 0.1786 | 1.0186 | 0.9903 | 1.0477 | 0.2008 |
|  | 30-34.9 | 1.0573 | 1.0176 | 1.0985 | 0.0043 | 1.0339 | 0.9945 | 1.0748 | 0.0927 |
|  | 35-39.9 | 1.0509 | 0.9939 | 1.1112 | 0.0808 | 0.9933 | 0.9384 | 1.0514 | 0.8164 |
|  | >=40 | 1.1545 | 1.0856 | 1.2277 | <.0001 | 1.0833 | 1.0179 | 1.1529 | 0.0117 |
|  | Unknown | 0.8919 | 0.8626 | 0.9223 | <.0001 | 0.8978 | 0.8668 | 0.9300 | <.0001 |
| Age and labour force quintile (previously dependency quintile) | Q1 (REF) | 1 | - | - | - | 1 | - | - | - |
|  | Q2 | 1.0455 | 1.0146 | 1.0772 | 0.0036 | 1.0083 | 0.9784 | 1.0390 | 0.5908 |
|  | Q3 | 1.1422 | 1.1067 | 1.1788 | <.0001 | 1.0767 | 1.0425 | 1.1120 | <.0001 |
|  | Q4 | 1.1642 | 1.1268 | 1.2029 | <.0001 | 1.0785 | 1.0429 | 1.1153 | <.0001 |
|  | Q5 | 1.2095 | 1.1686 | 1.2519 | <.0001 | 1.0681 | 1.0300 | 1.1076 | 0.0004 |
|  | Missing | 1.4009 | 1.1923 | 1.6459 | <.0001 | 1.3636 | 1.1276 | 1.6489 | 0.0014 |
| Material resources quintile (previously deprivation quintile) | Q1 (REF) | 1 | - | - | - | 1 | - | - | - |
|  | Q2 | 0.9299 | 0.9007 | 0.9600 | <.0001 | 0.9441 | 0.9145 | 0.9747 | 0.0004 |
|  | Q3 | 0.9388 | 0.9086 | 0.9701 | 0.0002 | 0.9529 | 0.9218 | 0.9851 | 0.0044 |
|  | Q4 | 0.9512 | 0.9199 | 0.9835 | 0.0034 | 0.9631 | 0.9301 | 0.9972 | 0.0343 |
|  | Q5 | 0.9616 | 0.9300 | 0.9942 | 0.0215 | 0.9941 | 0.9580 | 1.0316 | 0.7551 |
|  | Missing | 1.2385 | 1.0537 | 1.4556 | 0.0095 | N/A | N/A | N/A | N/A |
| Households and dwellings quintile (previously instability quintile) | Q1 (REF) | 1 | - | - | - | 1 | - | - | - |
|  | Q2 | 1.1544 | 1.1155 | 1.1946 | <.0001 | 1.0415 | 1.0059 | 1.0784 | 0.0221 |
|  | Q3 | 1.2265 | 1.1853 | 1.2690 | <.0001 | 1.0814 | 1.0441 | 1.1200 | <.0001 |
|  | Q4 | 1.2368 | 1.1952 | 1.2800 | <.0001 | 1.0907 | 1.0517 | 1.1312 | <.0001 |
|  | Q5 | 1.2391 | 1.1995 | 1.2799 | <.0001 | 1.0951 | 1.0575 | 1.1341 | <.0001 |
|  | Missing | 1.5072 | 1.2819 | 1.7720 | <.0001 | N/A | N/A | N/A | N/A |
| Maternal immigrant/refugee status | Not an immigrant (REF) | 1 | - | - | - | 1 | - | - | - |
|  | Economic | 0.8515 | 0.8236 | 0.8803 | <.0001 | 0.7109 | 0.6515 | 0.7758 | <.0001 |
|  | Family | 0.7509 | 0.7254 | 0.7773 | <.0001 | 0.6902 | 0.6320 | 0.7537 | <.0001 |
|  | Resettled refugee & protected person | 0.7136 | 0.6691 | 0.7610 | <.0001 | 0.6842 | 0.6209 | 0.7538 | <.0001 |
|  | Other | 0.7796 | 0.6303 | 0.9642 | 0.0217 | 0.6891 | 0.5490 | 0.8649 | 0.0013 |
| Geographic location | Urban (REF) | 1 | - | - | - | 1 | - | - | - |
|  | Rural | 1.4235 | 1.3742 | 1.4746 | <.0001 | 1.3375 | 1.2880 | 1.3888 | <.0001 |
|  | Missing | 0.9017 | 0.6608 | 1.2306 | 0.5144 | 0.7001 | 0.4889 | 1.0026 | 0.0517 |
| Tobaco use in pregnancy | No (REF) | 1 | - | - | - | 1 | - | - | - |
|  | Yes | 1.0171 | 0.9753 | 1.0606 | 0.4294 | 1.0227 | 0.9785 | 1.0690 | 0.3189 |
|  | Missing | 1.2082 | 1.1481 | 1.2714 | <.0001 | 0.9841 | 0.9159 | 1.0574 | 0.6622 |
| Drug and substance exposure in pregnancy | No (REF) | 1 | - | - | - | 1 | - | - | - |
|  | Yes | 1.0728 | 0.9770 | 1.1781 | 0.1409 | 1.0319 | 0.9375 | 1.1359 | 0.5206 |
|  | Missing | 1.2440 | 1.1857 | 1.3052 | <.0001 | 0.9517 | 0.8887 | 1.0192 | 0.1565 |
| Type of conception | Spontaneous (REF) | 1 | - | - | - | 1 | - | - | - |
|  | Assisted | 1.6002 | 1.5313 | 1.6722 | <.0001 | 1.4695 | 1.4048 | 1.5371 | <.0001 |
|  | Unknown | 1.3514 | 1.2973 | 1.4077 | <.0001 | 1.1953 | 1.1333 | 1.2608 | <.0001 |
| First trimester prenatal visit | No | 0.9037 | 0.8600 | 0.9496 | <.0001 | 0.9812 | 0.9338 | 1.0310 | 0.4527 |
|  | Yes (REF) | 1 | - | - | - | 1 | - | - | - |
|  | Missing | 1.3768 | 1.3313 | 1.4238 | <.0001 | 1.4318 | 1.3712 | 1.4951 | <.0001 |
| Pre-existing diabetes | No (REF) | 1 | - | - | - | 1 | - | - | - |
|  | Yes | 0.9608 | 0.8859 | 1.0420 | 0.3340 | 0.9508 | 0.8765 | 1.0313 | 0.2238 |
| Gestational diabetes | No (REF) | 1 | - | - | - | 1 | - | - | - |
|  | Yes | 0.9577 | 0.9213 | 0.9954 | 0.0283 | 0.9806 | 0.9425 | 1.0204 | 0.3347 |
| Pre-existing hypertension | No (REF) | 1 | - | - | - | 1 | - | - | - |
|  | Yes | 1.2909 | 1.2220 | 1.3636 | <.0001 | 1.1682 | 1.0996 | 1.2411 | <.0001 |
| Pregnancy induced hypertension | No (REF) | 1 | - | - | - | 1 | - | - | - |
|  | Yes | 1.4540 | 1.3957 | 1.5147 | <.0001 | 1.2843 | 1.2265 | 1.3449 | <.0001 |
| Previous cesarean delivery | No (REF) | 1 | - | - | - | 1 | - | - | - |
|  | Yes | 0.5413 | 0.5205 | 0.5628 | <.0001 | 1.0575 | 1.0052 | 1.1126 | 0.0308 |
| Placental previa | No (REF) | 1 | - | - | - | 1 | - | - | - |
|  | Yes | 2.1826 | 2.0127 | 2.3669 | <.0001 | 3.3691 | 3.0767 | 3.6893 | <.0001 |
| Placenta accreta spectrum | No (REF) | 1 | - | - | - | 1 | - | - | - |
|  | Yes | 8.1629 | 7.0121 | 9.5026 | <.0001 | 6.5072 | 5.3778 | 7.8738 | <.0001 |
| Placental abruption | No (REF) | 1 | - | - | - | 1 | - | - | - |
|  | Yes | 1.6905 | 1.4340 | 1.9928 | <.0001 | 1.7080 | 1.4440 | 2.0203 | <.0001 |
| Induction | No (REF) | 1 | - | - | - | 1 | - | - | - |
|  | Yes | 1.5208 | 1.4871 | 1.5552 | <.0001 | 1.2701 | 1.2348 | 1.3064 | <.0001 |
| Augmentation | No (REF) | 1 | - | - | - | 1 | - | - | - |
|  | Yes | 1.0212 | 0.9984 | 1.0446 | 0.0687 | 0.9950 | 0.9677 | 1.0230 | 0.7216 |
| Episiotomy | Mediolateral | 1.4798 | 1.4347 | 1.5263 | <.0001 | 1.0141 | 0.9783 | 1.0513 | 0.4450 |
|  | Midline | 1.0482 | 0.9523 | 1.1538 | 0.3361 | 0.8777 | 0.7973 | 0.9661 | 0.0077 |
|  | None (REF) | 1 | - | - | - | 1 | - | - | - |
|  | Unknown | 0.6713 | 0.6462 | 0.6973 | <.0001 | 0.9430 | 0.8988 | 0.9894 | 0.0166 |
| Fetal presentation | Breech | 0.5072 | 0.4686 | 0.5489 | <.0001 | 0.8566 | 0.7875 | 0.9317 | 0.0003 |
|  | Cephalic (REF) | 1 | - | - | - | 1 | - | - | - |
|  | Transverse | 0.9201 | 0.7959 | 1.0636 | 0.2602 | 1.2338 | 1.0668 | 1.4270 | 0.0046 |
|  | Unknown | 1.0342 | 0.9878 | 1.0827 | 0.1508 | 1.0311 | 0.9786 | 1.0865 | 0.2504 |
| Duration of second stage of labour | <60 minutes (REF) | 1 | - | - | - | 1 | - | - | - |
|  | 60-119 minutes | 1.3250 | 1.2853 | 1.3659 | <.0001 | 1.1397 | 1.1032 | 1.1774 | <.0001 |
|  | 120-179 minutes | 1.5745 | 1.5185 | 1.6325 | <.0001 | 1.2585 | 1.2102 | 1.3088 | <.0001 |
|  | 180-239 minutes | 1.8074 | 1.7274 | 1.8911 | <.0001 | 1.3781 | 1.3130 | 1.4465 | <.0001 |
|  | 240+ minutes | 1.7904 | 1.7094 | 1.8753 | <.0001 | 1.3697 | 1.3030 | 1.4398 | <.0001 |
|  | No second stage - n (%) | 0.7250 | 0.7041 | 0.7466 | <.0001 | 1.0920 | 0.9886 | 1.2063 | 0.0830 |
| Mode of delivery | SVD (REF) | 1 | - | - | - | 1 | - | - | - |
|  | Forceps delivery | 2.1211 | 2.0254 | 2.2213 | <.0001 | 1.5501 | 1.4721 | 1.6322 | <.0001 |
|  | Vacuum delivery | 1.3478 | 1.3002 | 1.3971 | <.0001 | 1.1439 | 1.1010 | 1.1885 | <.0001 |
|  | Forceps and vacuum delivery | 1.7866 | 1.5535 | 2.0547 | <.0001 | 1.3855 | 1.2024 | 1.5964 | <.0001 |
|  | Operative vaginal delivery (forceps/vacuum unknown) | 1.4972 | 0.5060 | 4.4300 | 0.4659 | 1.0368 | 0.3449 | 3.1166 | 0.9487 |
|  | First stage cesarean delivery (CD) | 0.8003 | 0.7698 | 0.8319 | <.0001 | 0.4605 | 0.4110 | 0.5160 | <.0001 |
|  | Second stage CD | 1.1509 | 1.0813 | 1.2249 | <.0001 | 0.6471 | 0.5825 | 0.7188 | <.0001 |
|  | CD without labour | 0.4860 | 0.4666 | 0.5062 | <.0001 | 0.8577 | 0.7798 | 0.9435 | 0.0016 |
|  | CD (missing/unknown stage) OR perimortem CD | 0.7820 | 0.6913 | 0.8846 | <.0001 | 0.6051 | 0.5188 | 0.7056 | <.0001 |
| Gestational age | Preterm (<37 weeks) | 1.1344 | 1.0892 | 1.1814 | <.0001 | 1.3788 | 1.3164 | 1.4442 | <.0001 |
|  | Term (37-41 weeks) (REF) | 1 | - | - | - | 1 | - | - | - |
|  | Post-term (42+ weeks) | 1.2836 | 1.0898 | 1.5119 | 0.0028 | 0.9573 | 0.8135 | 1.1264 | 0.5988 |
| Infant birth weight | <3000g | 0.8020 | 0.7795 | 0.8252 | <.0001 | 0.7296 | 0.7063 | 0.7536 | <.0001 |
|  | 3000-3999g (REF) | 1 | - | - | - | 1 | - | - | - |
|  | 4000-4499g | 1.4489 | 1.4022 | 1.4970 | <.0001 | 1.4782 | 1.4290 | 1.5292 | <.0001 |
|  | 4500g+ | 1.8293 | 1.7101 | 1.9567 | <.0001 | 1.9915 | 1.8588 | 2.1337 | <.0001 |
|  | Missing | 1.2185 | 1.0911 | 1.3607 | 0.0005 | 0.9422 | 0.8433 | 1.0527 | 0.2927 |
| Infant head circumference at birth | <33cm | 0.9526 | 0.9026 | 1.0053 | 0.0773 | 0.9824 | 0.9302 | 1.0375 | 0.5241 |
|  | 33-34cm (REF) | 1 | - | - | - | 1 | - | - | - |
|  | 35-36cm | 1.1561 | 1.1168 | 1.1967 | <.0001 | 1.0377 | 1.0019 | 1.0748 | 0.0391 |
|  | 37cm+ | 1.3262 | 1.2502 | 1.4068 | <.0001 | 1.0154 | 0.9547 | 1.0799 | 0.6269 |
|  | Missing | 1.1767 | 1.1440 | 1.2103 | <.0001 | 1.1175 | 1.0861 | 1.1499 | <.0001 |
