# Supplementary material for: Asian-White racial disparities in postpartum hemorrhage and severe postpartum hemorrhage in Ontario, Canada: A population-based cohort study
Source: PLoS One. 2026 Mar 12;21(3):e0344365. doi: 10.1371/journal.pone.0344365 (PMC12981453; doi:10.1371/journal.pone.0344365)
Supplement: S3 File — (DOCX) [file pone.0344365.s011.docx]

**data** pregnancies;

retain pregnancy_id_enc m_ikn b_bdate;

length maternal_bmi_cat $ **9.**;

length presentation_type $ **10.**;

length conception_type $ **11.**;

length preex_dm preg_hyper cs_stage $ **12.**;

length forceps_vacuum $ **18.**;

length cs_type $ **26.**;

length birth_type $ **29.**;

length augmentation $ **34.**;

length induction_method $ **54.**;

set born.agg_pregnancy;

if **'01APR2013'd** <= b_bdate <= **'31MAR2021'd**;

if parity = **.** then parity_cat = 'Unknown';

else if parity = **0** then parity_cat = '0';

else if parity = **1** then parity_cat = '1';

else if **2** <= parity <= **3** then parity_cat = '2-3';

else if parity >= **4** then parity_cat = '4+';

if number_of_fetuses = **.** then plurality = **9**;

else if number_of_fetuses = **1** then plurality = **0**;

else if number_of_fetuses > **1** then plurality = **1**;

if maternal_bmi = **.** then maternal_bmi_cat = 'Unknown';

else if maternal_bmi < **18.5** then maternal_bmi_cat = '<18.5';

else if **18.5** <= maternal_bmi < **25** then maternal_bmi_cat = '18.5-24.9';

else if **25** <= maternal_bmi < **30** then maternal_bmi_cat = '25-29.9';

else if **30** <= maternal_bmi < **35** then maternal_bmi_cat = '30-34.9';

else if **35** <= maternal_bmi < **40** then maternal_bmi_cat = '35-39.9';

else if maternal_bmi >= **40** then maternal_bmi_cat = '>=40';

if matsmokingatfirstprenvisit_id in ('1017440' '1017450' '1017460' '1017470') then smoking_firstvisit = **1**;

else if matsmokingatfirstprenvisit_id = '1017430' then smoking_firstvisit = **0**;

else smoking_firstvisit = **9**;

if mat_smoking_at_adm_for_birth_id in ('1017390' '1017400' '1017410' '1017420') then smoking_atbirth = **1**;

else if mat_smoking_at_adm_for_birth_id = '1017380' then smoking_atbirth = **0**;

else smoking_atbirth = **9**;

if index(expos_drug_and_subst_id, '1020460') > **0** or index(expos_drug_and_subst_id, '1020470') > **0** or index(expos_drug_and_subst_id, '1020480') > **0**

or index(expos_drug_and_subst_id, '1020490') > **0** or index(expos_drug_and_subst_id, '1020500') > **0** or index(expos_drug_and_subst_id, '1020510') > **0**

or index(expos_drug_and_subst_id, '1020520') > **0** or index(expos_drug_and_subst_id, '1020530') > **0** then preg_drug_exposure = **1**;

else if expos_drug_and_subst_id = '1020457' then preg_drug_exposure = **0**;

else preg_drug_exposure = **9**;

if index(conception_type_id, '1013110') > **0** or index(conception_type_id, '1013120') > **0** or index(conception_type_id, '1013130') > **0**

or index(conception_type_id, '1013140') > **0** or index(conception_type_id, '1013150') > **0** or index(conception_type_id, '1013170') > **0**

or index(conception_type_id, '3000006') > **0** then conception_type = 'Assisted';

else if index(conception_type_id, '1013160') > **0** then conception_type = 'Spontaneous';

else conception_type = 'Unknown';

if first_trimester_visit_flag = 'U' then tri1_prenatal_visit = **9**;

else if first_trimester_visit_flag = 'N' then tri1_prenatal_visit = **0**;

else if first_trimester_visit_flag in ('Y' 'y') then tri1_prenatal_visit = **1**;

if diabetes_and_pregnancy_id = '1013500' then preex_dm = 'Type I';

else if diabetes_and_pregnancy_id in ('1013520' '1013550') then preex_dm = 'Type II';

else if diabetes_and_pregnancy_id = '1013390' then preex_dm = 'None';

else if diabetes_and_pregnancy_id in ('1013440' '1013470' '3000001') then preex_dm = 'Gestational';

else if diabetes_and_pregnancy_id = '3000002' then preex_dm = 'Type Unknown';

else preex_dm = 'N/A';

if preg_hypertension_disorder_id = '1020800' then preg_hyper = 'Eclampsia';

else if preg_hypertension_disorder_id = '1020820' then preg_hyper = 'HELLP';

else if preg_hypertension_disorder_id in ('1020840' '1020850' '1020854') then preg_hyper = 'Preeclampsia';

else if preg_hypertension_disorder_id = '1020810' then preg_hyper = 'Gestational';

else if preg_hypertension_disorder_id = '1020830' then preg_hyper = 'None';

else preg_hyper = 'N/A';

if num_of_prev_cs_births > **0** then previous_cd = **1**; else previous_cd = **0**;

if index(complication_id, '1020340') > **0** then placenta_previa = **1**; else placenta_previa = **0**;

if index(complication_id, '1020310') > **0** or index(complication_id, '1020320') > **0** or index(complication_id, '1020330') > **0**

then placenta_accspec = **1**; else placenta_accspec = **0**;

if index(complication_id, '1020300') > **0** then placental_abruption = **1**; else placental_abruption = **0**;

if presentation_type_id in ('1021100' '1021110' '1021120' '1021130' '1021140' '3000089') then presentation_type = 'Breech';

else if presentation_type_id in ('1021150' '1021160' '1021170' '1021180' '1021190' '3000088') then presentation_type = 'Cephalic';

else if presentation_type_id = '1021200' then presentation_type = 'Transverse';

else presentation_type = 'Unknown';

if birth_type_id = '1012880' then birth_type = 'OVD';

else if birth_type_id = '1012890' then birth_type = 'Induced/spontaneous labour CD';

else if birth_type_id = '1012900' then birth_type = 'No labour CD';

else if birth_type_id = '1012910' then birth_type = 'SVD';

else birth_type = 'N/A';

if forceps_vacuum_id = '1013830' then forceps_vacuum = 'Forceps';

else if forceps_vacuum_id = '1013840' then forceps_vacuum = 'None';

else if forceps_vacuum_id = '1013850' then forceps_vacuum = 'Vacuum';

else if forceps_vacuum_id = '1013860' then forceps_vacuum = 'Forceps and Vacuum';

else forceps_vacuum = 'N/A';

if cs_stage_id = '1005730' then cs_stage = 'First stage';

else if cs_stage_id = '1005740' then cs_stage = 'Perimortem';

else if cs_stage_id = '1005750' then cs_stage = 'Second stage';

else if cs_stage_id = '3000021' then cs_stage = 'No labour';

else cs_stage = 'N/A';

if cs_type_id = '1013360' then cs_type = 'Planned (as scheduled)';

else if cs_type_id = '1013370' then cs_type = 'Planned (not as scheduled)';

else if cs_type_id = '1013380' then cs_type = 'Unplanned';

else if cs_type_id = '1048727' then cs_type = 'Planned';

else if cs_type_id = '-1' then cs_type = 'N/A';

if labour_induction_method_id = '1014610' then induction_method = 'Amniotomy';

else if labour_induction_method_id = '1014610;1014620' then induction_method = 'Amniotomy; Oxytocin';

else if labour_induction_method_id = '1014610;1014620;1014625' then induction_method = 'Amniotomy; Oxytocin; Prostaglandin';

else if labour_induction_method_id = '1014610;1014620;1014625;1014627' then induction_method = 'Amniotomy; Oxytocin; Prostaglandin; Sweeping membranes';

else if labour_induction_method_id = '1014610;1014620;1014627' then induction_method = 'Amniotomy; Oxytocin; Sweeping membranes';

else if labour_induction_method_id = '1014610;1014627' then induction_method = 'Amniotomy; Sweeping membranes';

else if labour_induction_method_id = '1014620' then induction_method = 'Oxytocin';

else if labour_induction_method_id = '1014620;1014625' then induction_method = 'Oxytocin; Prostaglandin';

else if labour_induction_method_id = '1014620;1014625;1014627' then induction_method = 'Oxytocin; Prostaglandin; Sweeping membranes';

else if labour_induction_method_id = '1014620;1014627' then induction_method = 'Oxytocin; Sweeping membranes';

else if labour_induction_method_id = '1014625' then induction_method = 'Prostaglandin';

else if labour_induction_method_id = '1014625;1014627' then induction_method = 'Prostaglandin; Sweeping membranes';

else if labour_induction_method_id = '1014627' then induction_method = 'Sweeping membranes';

else if labour_induction_method_id = '1048728' then induction_method = 'None';

else induction_method = 'N/A';

if augmentation_id = '1012680' then augmentation = 'Amniotomy';

else if augmentation_id = '1012690' then augmentation = 'None';

else if augmentation_id = '1012700' then augmentation = 'Oxytocin';

else if augmentation_id = '1012710' then augmentation = 'Prostaglandin';

else if augmentation_id = '1012680;1012700' then augmentation = 'Amniotomy; Oxytocin';

else if augmentation_id = '1012710;1012680' then augmentation = 'Amniotomy; Prostaglandin';

else if augmentation_id = '1012710;1012680;1012700' then augmentation = 'Amniotomy; Oxytocin; Prostaglandin';

else if augmentation_id = '1012710;1012700' then augmentation = 'Oxytocin; Prostaglandin';

else augmentation = 'N/A';

if episiotomy_type_id = '1013620' then episiotomy = 'Mediolateral';

else if episiotomy_type_id = '1013630' then episiotomy = 'Midline';

else if episiotomy_type_id = '1013640' then episiotomy = 'None';

else episiotomy = 'Unknown';

keep pregnancy_id_enc m_ikn b_bdate ga_at_birth_days ga_at_birth_weeks m_valikn agg_pregnancy_outcome_id parity number_of_fetuses maternal_bmi

tri1_prenatal_visit num_of_prev_cs_births lbr_time_sec_stg_total_minutes number_of_induction_attempts parity_cat plurality maternal_bmi_cat

smoking_firstvisit smoking_atbirth preg_drug_exposure conception_type preex_dm preg_hyper previous_cd placenta_previa placenta_accspec

placental_abruption presentation_type birth_type forceps_vacuum cs_stage cs_type induction_method augmentation episiotomy;

**run**;

**data** pregnancies;

length gestage_cat $ **13.**;

set pregnancies;

if ga_at_birth_weeks >= **20**;

if ga_at_birth_weeks < **37** then gestage_cat = '(1) Preterm';

else if **37** <= ga_at_birth_weeks <= **41** then gestage_cat = '(2) Term';

else if ga_at_birth_weeks > **41** then gestage_cat = '(3) Post-term';

rename m_ikn = ikn;

**run**;

**data** pregnancies;

set pregnancies;

if pregnancy_id_enc = '' then delete;

**run**;

**data** pregnancies;

set pregnancies;

if m_valikn = 'V';

drop m_valikn;

**run**;

**proc** **sort** data=pregnancies nodupkey; by pregnancy_id_enc; **run**;

%***getdemo***(

data = pregnancies,

out = pregnancies_demo,

getsex = F,

getdeath = T,

agedate = b_bdate,

geodate = b_bdate);

**data** pregnancies_demo;

set pregnancies_demo;

if substr(prcddablk, **1**, **2**) ^= '35' then delete;

**run**;

**data** pregnancies_demo;

length preg_outcome $ **50.**;

set pregnancies_demo;

if index(agg_pregnancy_outcome_id, '1021035') > **0** or index(agg_pregnancy_outcome_id, '1021040') > **0**

or index(agg_pregnancy_outcome_id, '1021050') > **0** or index(agg_pregnancy_outcome_id, '1021070') > **0** then delete;

if agg_pregnancy_outcome_id in ('1021030; 1021030' '1021030; 1021030; 1021030' '1021030; 1021030; 1021030; 1021030'

'1021030; 1021030; 1021030; 1021030; 1021030') then preg_outcome = '1021030';

else if agg_pregnancy_outcome_id = '1021060; 1021060' then preg_outcome = '1021060';

else if agg_pregnancy_outcome_id in ('1021080; 1021080' '1021080; 1021080; 1021080') then preg_outcome = '1021080';

else if agg_pregnancy_outcome_id in ('1021090; 1021090' '1021090; 1021090; 1021090') then preg_outcome = '1021090';

else if agg_pregnancy_outcome_id in ('1021030; 1021030; 1021080' '1021030; 1021080; 1021080' '1021030; 1021080; 1021080; 1021080; 1021080; 1021080')

then preg_outcome = '1021030; 1021080';

else if agg_pregnancy_outcome_id = '1021030; 1021030; 1021090' then preg_outcome = '1021030; 1021090';

else preg_outcome = agg_pregnancy_outcome_id;

drop agg_pregnancy_outcome_id;

**run**;

**data** pregnancies_demo;

set pregnancies_demo;

if dthdate ^= **.** and dthdate < b_bdate then delete;

**run**;

**data** redpph.preg_cohort;

length age_group $ **5.**;

set pregnancies_demo;

if age < **20** then age_group = '<20';

else if **20** <= age < **25** then age_group = '20-24';

else if **25** <= age < **30** then age_group = '25-29';

else if **30** <= age < **35** then age_group = '30-34';

else if **35** <= age < **40** then age_group = '35-39';

else if age >= **40** then age_group = '40+';

**run**;

/* Pull immigration information: */

**proc** **sql**;

create table preg_immig as

select a.pregnancy_id_enc, a.ikn, a.b_bdate, b.landing_date, b.nat_language, b.immigration_category

from redpph.preg_cohort as a left join cic.cic_ircc as b

on a.ikn = b.ikn;

**quit**;

**proc** **sort** data=preg_immig nodupkey; by pregnancy_id_enc landing_date nat_language immigration_category; **run**;

**proc** **sql**;

create table num_rows as

select pregnancy_id_enc, count(*) as num_rows, count(unique landing_date) as num_dates

from preg_immig

group by pregnancy_id_enc;

create table preg_immig as

select * from preg_immig as a left join num_rows as b

on a.pregnancy_id_enc = b.pregnancy_id_enc;

**quit**;

**data** preg_immig;

set preg_immig;

if num_rows > **1** then delete;

if landing_date ^= **.** then do;

if nat_language in ('-1' '0' '000') then language_cat = 'Unknown';

else if nat_language = '001' then language_cat = 'English';

else if nat_language = '002' then language_cat = 'French';

else language_cat = 'Other';

end;

immig_cat = immigration_category;

if landing_date ^= **.** then immig = **1**; else immig = **0**;

if immig = **1** then do;

immig_birth_datediff = (b_bdate - landing_date)/**365.25**;

end;

drop num_rows num_dates;

format immig_cat CIC_IMMIGCATEG_IRCC_5CAT.;

**run**;

**proc** **sql**;

create table redpph.preg_cohort as

select a.*, b.landing_date, b.nat_language, b.immigration_category, b.language_cat, b.immig_cat, b.immig, b.immig_birth_datediff

from redpph.preg_cohort as a inner join preg_immig as b

on a.pregnancy_id_enc = b.pregnancy_id_enc;

**quit**;

**proc** **sql**;

create table born_merge as

select a.pregnancy_id_enc, a.lbr_time_sec_stg_total_minutes, a.plurality, a.number_of_fetuses, b.consult_reason_id

from redpph.preg_cohort as a left join born.mw_pregnancy_coc_consult as b

on a.pregnancy_id_enc = b.pregnancy_id_enc;

**quit**;

**data** born_merge;

set born_merge;

if index(consult_reason_id, '1025340') > **0** and plurality = **0** then plurality = **1**;

if plurality = **1** then priority = **1**;

else if plurality = **0** then priority = **2**;

else if plurality = **9** then priority = **3**;

drop consult_reason_id;

**run**;

**proc** **sort** data=born_merge; by pregnancy_id_enc priority; **run**;

**proc** **sort** data=born_merge nodupkey out=born_merge(drop=priority); by pregnancy_id_enc; **run**;

**proc** **sql**;

create table born_merge as

select a.*, b.mw_prenatalnumofvisitshome, b.mw_prenatalnumofvisitshospital, b.mw_prenatalnumofvisitsclinic

from born_merge as a left join born.mw_pregnancy_coc as b

on a.pregnancy_id_enc = b.pregnancy_id_enc;

**quit**;

**data** born_merge;

set born_merge;

total_prenatal_visits = (mw_prenatalnumofvisitshome + mw_prenatalnumofvisitshospital + mw_prenatalnumofvisitsclinic);

**run**;

**proc** **sort** data=born_merge; by pregnancy_id_enc descending total_prenatal_visits; **run**;

**proc** **sort** data=born_merge nodupkey; by pregnancy_id_enc; **run**;

**proc** **sql**;

create table born_merge as

select a.*, b.antenatal_consult_reason_all_id

from born_merge as a left join born.ag as b

on a.pregnancy_id_enc = b.pregnancy_id_enc;

**quit**;

**data** born_merge;

set born_merge;

if index(antenatal_consult_reason_all_id, '1025170') > **0** then born_preex_hyper = **1**; else born_preex_hyper = **0**;

drop antenatal_consult_reason_all_id;

**run**;

**proc** **sort** data=born_merge; by pregnancy_id_enc descending born_preex_hyper; **run**;

**proc** **sort** data=born_merge nodupkey; by pregnancy_id_enc; **run**;

**proc** **sql**;

create table born_merge as

select a.*, b.cs_stage_id, b.cs_type_id, b.cs_dilation_cm_id

from born_merge as a left join born.lbm as b

on a.pregnancy_id_enc = b.pregnancy_id_enc;

**quit**;

**data** born_merge;

length duration_stage2 $ **15.**;

set born_merge;

if lbr_time_sec_stg_total_minutes ^= **.** then do;

if lbr_time_sec_stg_total_minutes < **60** then duration_stage2 = '<60 minutes';

else if **60** <= lbr_time_sec_stg_total_minutes < **120** then duration_stage2 = '60-119 minutes';

else if **120** <= lbr_time_sec_stg_total_minutes < **180** then duration_stage2 = '120-179 minutes';

else if **180** <= lbr_time_sec_stg_total_minutes < **240** then duration_stage2 = '180-239 minutes';

else if lbr_time_sec_stg_total_minutes >= **240** then duration_stage2 = '>=240 minutes';

end;

else do;

if cs_stage_id = '3000021' or cs_type_id in ('1013360' '1013370' '1048727') or cs_dilation_cm_id in ('1013230' '1013240'

'1013250' '1013260' '1013270' '1013280' '1013290' '1013300' '1013310' '1013320') then duration_stage2 = 'No second stage';

else duration_stage2 = 'Missing';

end;

if duration_stage2 = 'Missing' then priority = **2**;

else priority = **1**;

drop cs_stage_id cs_type_id cs_dilation_cm_id;

**run**;

**proc** **sort** data=born_merge; by pregnancy_id_enc priority; **run**;

**proc** **sort** data=born_merge nodupkey out=born_merge(drop=priority); by pregnancy_id_enc; **run**;

**proc** **sql**;

create table born_merge as

select a.*, b.b_ikn, b.birth_weight_grams, b.head_circumference_at_birth_cm

from born_merge as a left join born.agg_infant as b

on a.pregnancy_id_enc = b.pregnancy_id_enc;

**quit**;

**proc** **sort** data=born_merge; by pregnancy_id_enc b_ikn; **run**;

**data** fetus1 remaining; set born_merge; by pregnancy_id_enc;

if first.pregnancy_id_enc = **1** then output fetus1; else output remaining;

**run**;

**proc** **sort** data=remaining; by pregnancy_id_enc b_ikn; **run**;

**data** fetus2 remaining; set remaining; by pregnancy_id_enc;

if first.pregnancy_id_enc = **1** then output fetus2; else output remaining;

**run**;

**proc** **sort** data=remaining; by pregnancy_id_enc b_ikn; **run**;

**data** fetus3 remaining; set remaining; by pregnancy_id_enc;

if first.pregnancy_id_enc = **1** then output fetus3; else output remaining;

**run**;

**data** fetus1; set fetus1; rename b_ikn=b_ikn1 birth_weight_grams=b_weight_grams1 head_circumference_at_birth_cm=b_headcirc_cm1; **run**;

**data** fetus2; set fetus2; keep pregnancy_id_enc b_ikn birth_weight_grams head_circumference_at_birth_cm;

rename b_ikn=b_ikn2 birth_weight_grams=b_weight_grams2 head_circumference_at_birth_cm=b_headcirc_cm2;

**run**;

**data** fetus3; set fetus3; keep pregnancy_id_enc b_ikn birth_weight_grams head_circumference_at_birth_cm;

rename b_ikn=b_ikn3 birth_weight_grams=b_weight_grams3 head_circumference_at_birth_cm=b_headcirc_cm3;

**run**;

**data** fetus4; set remaining; keep pregnancy_id_enc b_ikn birth_weight_grams head_circumference_at_birth_cm;

rename b_ikn=b_ikn4 birth_weight_grams=b_weight_grams4 head_circumference_at_birth_cm=b_headcirc_cm4;

**run**;

**data** final_born_merge;

merge fetus1 fetus2 fetus3 fetus4;

by pregnancy_id_enc;

if number_of_fetuses = **.** and b_ikn1 ^= '' then do;

number_of_fetuses = **1**;

plurality = **0**;

end;

**run**;

**data** final_born_merge;

length b_weight_g_cat1 b_weight_g_cat2 b_weight_g_cat3 b_weight_g_cat4 $ **9.**;

length b_headcirc_cm_cat1 b_headcirc_cm_cat2 b_headcirc_cm_cat3 b_headcirc_cm_cat4 $ **7.**;

set final_born_merge;

array weight[**4**] b_weight_grams1-b_weight_grams4;

array wcat[**4**] b_weight_g_cat1 b_weight_g_cat2 b_weight_g_cat3 b_weight_g_cat4;

array circ[**4**] b_headcirc_cm1-b_headcirc_cm4;

array ccat[**4**] b_headcirc_cm_cat1 b_headcirc_cm_cat2 b_headcirc_cm_cat3 b_headcirc_cm_cat4;

do i = **1** to **4**;

if weight[i] ^= **.** then do;

if weight[i] < **3000** then wcat[i] = '<3000';

else if **3000** <= weight[i] < **4000** then wcat[i] = '3000-3999';

else if **4000** <= weight[i] < **4500** then wcat[i] = '4000-4499';

else if weight[i] >= **4500** then wcat[i] = '>=4500';

end;

else wcat[i] = 'Missing';

if circ[i] ^= **.** then do;

if circ[i] < **33** then ccat[i] = '<33';

else if **33** <= circ[i] < **35** then ccat[i] = '33-34';

else if **35** <= circ[i] < **37** then ccat[i] = '35-36';

else if circ[i] >= **37** then ccat[i] = '>=37';

end;

else ccat[i] = 'Missing';

end;

drop lbr_time_sec_stg_total_minutes i;

**run**;

**data** redpph.preg_cohort;

set redpph.preg_cohort;

drop plurality number_of_fetuses;

**run**;

**proc** **sort** data=redpph.preg_cohort;

by pregnancy_id_enc;

**run**;

**data** redpph.preg_cohort;

merge redpph.preg_cohort final_born_merge;

by pregnancy_id_enc;

**run**;

/* Pull ONMARG variables: */

**data** iknlist;

set redpph.preg_cohort;

keep pregnancy_id_enc ikn b_bdate;

**run**;

%***getonmarg***(

data = iknlist,

out = preg_cohort_onmarg,

date = b_bdate);

**proc** **sql**;

create table redpph.preg_cohort as

select a.*, b.age_labourforce_da, b.age_labourforce_q_da, b.households_dwellings_da, b.households_dwellings_q_da,

b.material_resources_da, b.material_resources_q_da, b.racialized_NC_pop_da, b.racialized_NC_pop_q_da

from redpph.preg_cohort as a left join preg_cohort_onmarg as b

on a.pregnancy_id_enc = b.pregnancy_id_enc;

**quit**;

/* Pull rural status from PCCF: */

**data** preg2011 preg2016 preg2021;

set redpph.preg_cohort;

if **2012** <= year(b_bdate) <= **2013** then output preg2011;

else if **2014** <= year(b_bdate) <= **2018** then output preg2016;

else if **2019** <= year(b_bdate) then output preg2021;

keep pregnancy_id_enc ikn b_bdate pstlcode dthdate;

**run**;

%***pccf2011***(

data = preg2011,

id = ikn,

pcode = pstlcode,

fixpstl = T,

residence = T,

geovars = rural,

out = preg2011_pccf);

%***pccf2016***(

data = preg2016,

id = ikn,

pcode = pstlcode,

fixpstl = T,

residence = T,

geovars = rural,

out = preg2016_pccf);

%***pccf2021***(

data = preg2021,

id = ikn,

pcode = pstlcode,

fixpstl = T,

residence = T,

geovars = rural,

out = preg2021_pccf);

**data** preg_pccf;

set preg2011_pccf preg2016_pccf preg2021_pccf;

if rural = '' then rural = 'Z';

**run**;

**data** redpph.preg_cohort;

set redpph.preg_cohort;

drop rural;

**run**;

**proc** **sql**;

create table redpph.preg_cohort as

select a.*, b.rural as rural_pccf

from redpph.preg_cohort as a left join preg_pccf as b

on a.pregnancy_id_enc = b.pregnancy_id_enc;

**quit**;

/* Pull diabetes and hypertension diagnoses from ODD and HYPER: */

**proc** **sql**;

create table redpph.preg_cohort as

select a.*, b.diag_last as odd_dxdate_general

from redpph.preg_cohort as a left join odd.odd2021 as b

on a.ikn = b.ikn;

create table redpph.preg_cohort as

select a.*, b.diag_last as odd_dxdate_specific

from redpph.preg_cohort as a left join odd.odd2021_specific as b

on a.ikn = b.ikn;

create table redpph.preg_cohort as

select a.*, b.diagdate_last as hyper_dxdate

from redpph.preg_cohort as a left join hyper.hypertension2021 as b

on a.ikn = b.ikn;

**quit**;

/* Number of prenatal visits from OHIP: */

**data** preg_cohort;

set redpph.preg_cohort;

preg_startdate = b_bdate - (ga_at_birth_weeks***7**);

keep pregnancy_id_enc ikn b_bdate preg_startdate;

format preg_startdate date9.;

**run**;

%***getohip***(

source = nonlab,

start = **20110601**,

end = **20210331**,

cohort = preg_cohort,

out = prenatal_visits,

where = %str(preg_startdate <= servdate < b_bdate and feecode in ('P002' 'P003' 'P004' 'P005')),

keep = ikn physnum servdate feecode);

**proc** **sort** data=prenatal_visits nodupkey; by pregnancy_id_enc servdate physnum; **run**;

**proc** **sql**;

create table num_prenatal_visits as

select pregnancy_id_enc, count(*) as num_prenatal_visits_ohip

from prenatal_visits

group by pregnancy_id_enc;

**quit**;

**proc** **sort** data=redpph.preg_cohort; by pregnancy_id_enc; **run**;

**data** redpph.preg_cohort;

merge redpph.preg_cohort(in=a) num_prenatal_visits(in=b);

by pregnancy_id_enc;

if a and not b then num_prenatal_visits_ohip = **0**;

**run**;

/* Gestational diabetes: */

%***getdadsds***(

source = inpat,

start = **20110601**,

end = **20210331**,

refdate = admdate,

cohort = preg_cohort,

out = gest_dm,

where = %str(preg_startdate <= admdate < b_bdate),

dxtype = all,

dx10code = ('O248' 'P700'),

keep = ikn admdate ddate,

inclsuspect = F);

**proc** **sort** data=gest_dm; by pregnancy_id_enc admdate; **run**;

**proc** **sort** data=gest_dm nodupkey out=gest_dm(keep=pregnancy_id_enc admdate); by pregnancy_id_enc; **run**;

**proc** **sql**;

create table redpph.preg_cohort as

select a.*, b.admdate as dad_gestdm_dxdate

from redpph.preg_cohort as a left join gest_dm as b

on a.pregnancy_id_enc = b.pregnancy_id_enc;

**quit**;

/* Preeclampsia: */

%***getdadsds***(

source = inpat,

start = **20110601**,

end = **20210331**,

refdate = admdate,

cohort = preg_cohort,

out = gest_preeclampsia,

where = %str(preg_startdate <= admdate < b_bdate),

dxtype = all,

dx10code = ('O11' 'O12' 'O13' 'O14' 'O15'),

keep = ikn admdate ddate,

inclsuspect = F);

**proc** **sort** data=gest_preeclampsia; by pregnancy_id_enc admdate; **run**;

**proc** **sort** data=gest_preeclampsia nodupkey out=gest_preeclampsia(keep=pregnancy_id_enc admdate); by pregnancy_id_enc; **run**;

**proc** **sql**;

create table redpph.preg_cohort as

select a.*, b.admdate as dad_preeclampsia_dxdate

from redpph.preg_cohort as a left join gest_preeclampsia as b

on a.pregnancy_id_enc = b.pregnancy_id_enc;

**quit**;

/* HELLP syndrome: */

%***getdadsds***(

source = inpat,

start = **20110601**,

end = **20210331**,

refdate = admdate,

cohort = preg_cohort,

out = gest_hellp,

where = %str(preg_startdate <= admdate < b_bdate),

dxtype = all,

dx10code = ('O14'),

keep = ikn admdate ddate,

inclsuspect = F);

**proc** **sort** data=gest_hellp; by pregnancy_id_enc admdate; **run**;

**proc** **sort** data=gest_hellp nodupkey out=gest_hellp(keep=pregnancy_id_enc admdate); by pregnancy_id_enc; **run**;

**proc** **sql**;

create table redpph.preg_cohort as

select a.*, b.admdate as dad_hellp_dxdate

from redpph.preg_cohort as a left join gest_hellp as b

on a.pregnancy_id_enc = b.pregnancy_id_enc;

**quit**;

/* Placenta previa: */

%***getdadsds***(

source = inpat,

start = **20110601**,

end = **20210331**,

refdate = admdate,

cohort = preg_cohort,

out = gest_pprevia,

where = %str(preg_startdate <= admdate < b_bdate),

dxtype = all,

dx10code = ('O44'),

keep = ikn admdate ddate,

inclsuspect = F);

**proc** **sort** data=gest_pprevia; by pregnancy_id_enc admdate; **run**;

**proc** **sort** data=gest_pprevia nodupkey out=gest_pprevia(keep=pregnancy_id_enc admdate); by pregnancy_id_enc; **run**;

**proc** **sql**;

create table redpph.preg_cohort as

select a.*, b.admdate as dad_pprevia_dxdate

from redpph.preg_cohort as a left join gest_pprevia as b

on a.pregnancy_id_enc = b.pregnancy_id_enc;

**quit**;

/* Placenta accreta spectrum: */

%***getdadsds***(

source = inpat,

start = **20110601**,

end = **20210331**,

refdate = admdate,

cohort = preg_cohort,

out = gest_paccspec,

where = %str(preg_startdate <= admdate < b_bdate),

dxtype = all,

dx10code = ('O432'),

keep = ikn admdate ddate,

inclsuspect = F);

**proc** **sort** data=gest_paccspec; by pregnancy_id_enc admdate; **run**;

**proc** **sort** data=gest_paccspec nodupkey out=gest_paccspec(keep=pregnancy_id_enc admdate); by pregnancy_id_enc; **run**;

**proc** **sql**;

create table redpph.preg_cohort as

select a.*, b.admdate as dad_paccspec_dxdate

from redpph.preg_cohort as a left join gest_paccspec as b

on a.pregnancy_id_enc = b.pregnancy_id_enc;

**quit**;

/* Placental abruption: */

%***getdadsds***(

source = inpat,

start = **20110601**,

end = **20210331**,

refdate = admdate,

cohort = preg_cohort,

out = gest_pabrupt,

where = %str(preg_startdate <= admdate < b_bdate),

dxtype = all,

dx10code = ('O45'),

keep = ikn admdate ddate,

inclsuspect = F);

**proc** **sort** data=gest_pabrupt; by pregnancy_id_enc admdate; **run**;

**proc** **sort** data=gest_pabrupt nodupkey out=gest_pabrupt(keep=pregnancy_id_enc admdate); by pregnancy_id_enc; **run**;

**proc** **sql**;

create table redpph.preg_cohort as

select a.*, b.admdate as dad_pabrupt_dxdate

from redpph.preg_cohort as a left join gest_pabrupt as b

on a.pregnancy_id_enc = b.pregnancy_id_enc;

**quit**;

/* Induction: */

%***getdadsds***(

source = inpat,

start = **20110601**,

end = **20210331**,

refdate = admdate,

cohort = preg_cohort,

out = delivery_induction,

where = %str(admdate <= b_bdate < ddate),

incode = ('5AC30'),

keep = ikn admdate ddate,

inclsuspect = F);

**proc** **sort** data=delivery_induction nodupkey out=delivery_induction(keep=pregnancy_id_enc); by pregnancy_id_enc; **run**;

**data** redpph.preg_cohort;

merge redpph.preg_cohort(in=a) delivery_induction(in=b);

by pregnancy_id_enc;

if a and b then dad_induction = **1**; else dad_induction = **0**;

**run**;

/* Episiotomy: */

%***getdadsds***(

source = inpat,

start = **20110601**,

end = **20210331**,

refdate = admdate,

cohort = preg_cohort,

out = delivery_episiotomy,

where = %str(admdate <= b_bdate < ddate),

incode = ('5MD53KJ' '5MD53KL' '5MD53KN' '5MD54KJ' '5MD54KL' '5MD54KN' '5MD54NF' '5MD55KN' '5MD55KL' '5MD55KJ' '5MD55KR' '5MD50GH' '5PC80JN'),

keep = ikn admdate ddate,

inclsuspect = F);

**proc** **sort** data=delivery_episiotomy nodupkey out=delivery_episiotomy(keep=pregnancy_id_enc); by pregnancy_id_enc; **run**;

**data** redpph.preg_cohort;

merge redpph.preg_cohort(in=a) delivery_episiotomy(in=b);

by pregnancy_id_enc;

if a and b then dad_episiotomy = **1**; else dad_episiotomy = **0**;

**run**;

**proc** **sql**;

create table mat_language as

select a.pregnancy_id_enc, b.primary_language_id

from redpph.preg_cohort as a left join born.agg_pregnancy as b

on a.pregnancy_id_enc = b.pregnancy_id_enc;

**quit**;

**proc** **freq** data=mat_language;

tables primary_language_id;

**run**;

**data** mat_language;

set mat_language;

if primary_language_id = '' then delete;

**run**;

**proc** **sort** data=mat_language nodupkey; by pregnancy_id_enc primary_language_id; **run**;

**proc** **sort** data=mat_language nodupkey; by pregnancy_id_enc; **run**;

**proc** **sort** data=redpph.preg_cohort nodupkey out=preg_ids(keep=pregnancy_id_enc); by pregnancy_id_enc; **run**;

**data** mat_language;

merge preg_ids(in=a) mat_language(in=b);

by pregnancy_id_enc;

if a and not b then primary_language = 'Unknown/missing';

else do;

if primary_language_id = '1' then primary_language = 'English';

else if primary_language_id = '2' then primary_language = 'French';

else if primary_language_id = '3' then primary_language = 'Other';

end;

drop primary_language_id;

**run**;

**proc** **sql**;

create table redpph.preg_cohort as

select a.*, b.primary_language

from redpph.preg_cohort as a left join mat_language as b

on a.pregnancy_id_enc = b.pregnancy_id_enc;

**quit**;

**proc** **sql**;

create table mat_ancestry_as as

select a.pregnancy_id_enc, b.maternal_ancestry_id

from redpph.preg_cohort as a left join born.as as b

on a.pregnancy_id_enc = b.pregnancy_id_enc;

create table mat_ancestry_psos as

select a.pregnancy_id_enc, b.ancestry

from redpph.preg_cohort as a left join born.psos as b

on a.pregnancy_id_enc = b.pregnancy_id_enc;

**quit**;

**data** mat_ancestry_psos;

set mat_ancestry_psos;

if ancestry = '' then delete;

**run**;

**proc** **sort** data=mat_ancestry_psos nodupkey; by pregnancy_id_enc ancestry; **run**;

**data** mat_ancestry_as;

length ancestry $ **9.**;

set mat_ancestry_as;

if maternal_ancestry_id in ('' '1006460') then delete;

if maternal_ancestry_id in ('1006295' '1006300') then ancestry = 'Asian';

else if maternal_ancestry_id = '1006330' then ancestry = 'Black';

else if maternal_ancestry_id = '1006350' then ancestry = 'Caucasian';

else if maternal_ancestry_id = '1006440' then ancestry = 'Other';

keep pregnancy_id_enc ancestry;

**run**;

**proc** **sort** data=mat_ancestry_as nodupkey; by pregnancy_id_enc ancestry; **run**;

**data** ancestry1(rename=(ancestry=ancestry1)) ancestry2(rename=(ancestry=ancestry2));

set mat_ancestry_as;

by pregnancy_id_enc;

if first.pregnancy_id_enc then output ancestry1;

else output ancestry2;

**run**;

**proc** **sql**;

create table mat_ancestry_as as

select a.pregnancy_id_enc, a.ancestry1, b.ancestry2

from ancestry1 as a left join ancestry2 as b

on a.pregnancy_id_enc = b.pregnancy_id_enc;

**quit**;

**data** mat_ancestry_as;

retain pregnancy_id_enc ancestry_as ancestry2 ancestry_combo;

length ancestry_combo $ **9.**;

set mat_ancestry_as;

if ancestry2 = '' then do;

ancestry_as = ancestry1;

ancestry_combo = ancestry1;

end;

else do;

ancestry_combo = '>1 race';

if ancestry1 = 'Black' then ancestry_as = 'Black';

else if ancestry1 = 'Asian' then ancestry_as = 'Asian';

else if ancestry1 = 'Caucasian' then ancestry_as = 'Caucasian';

end;

keep pregnancy_id_enc ancestry_as ancestry2 ancestry_combo;

rename ancestry2 = second_ancestry;

**run**;

**data** ancestry;

length ancestry_combo_new $ **9.**;

merge mat_ancestry_psos(in=a) mat_ancestry_as(in=b);

by pregnancy_id_enc;

if a then ancestry_source = 'PSOS';

else ancestry_source = 'AS';

if ancestry ^= '' then ancestry_combo_new = ancestry;

else if ancestry = '' then do;

ancestry_combo_new = ancestry_combo;

ancestry = ancestry_as;

end;

if ancestry_source = 'PSOS' then second_ancestry = '';

drop ancestry_as ancestry_combo;

rename ancestry_combo_new = ancestry_combo;

**run**;

**proc** **sort** data=redpph.preg_cohort nodupkey out=preg_ids(keep=pregnancy_id_enc); by pregnancy_id_enc; **run**;

**data** ancestry;

length ancestry ancestry_combo $ **15.**;

merge preg_ids(in=a) ancestry(in=b);

by pregnancy_id_enc;

if a and not b then do;

ancestry = 'Unknown/missing';

ancestry_combo = 'Unknown/missing';

end;

**run**;

**proc** **sql**;

create table redpph.preg_cohort as

select a.*, b.ancestry, b.ancestry_combo, b.second_ancestry, b.ancestry_source

from redpph.preg_cohort as a left join ancestry as b

on a.pregnancy_id_enc = b.pregnancy_id_enc;

**quit**;

**data** redpph.preg_cohort;

set redpph.preg_cohort;

if age_labourforce_q_da = **.** then age_labourforce_q_da = **9**;

if households_dwellings_q_da = **.** then households_dwellings_q_da = **9**;

if material_resources_q_da = **.** then material_resources_q_da = **9**;

if racialized_NC_pop_q_da = **.** then racialized_NC_pop_q_da = **9**;

if immig = **1** then do;

if immig_birth_datediff < **0** then immig_birth_datediff_cat = '(1) Landing date after b_bdate';

else if **0** <= immig_birth_datediff < **1** then immig_birth_datediff_cat = '(2) <1 year';

else if **1** <= immig_birth_datediff <= **5** then immig_birth_datediff_cat = '(3) 1-5 years';

else if **5** < immig_birth_datediff <= **10** then immig_birth_datediff_cat = '(4) >5-10 years';

else if immig_birth_datediff > **10** then immig_birth_datediff_cat = '(5) >10 years';

end;

else immig_birth_datediff_cat = '';

if induction_method in ('N/A' 'None') then induction = **0**; else induction = **1**;

if augmentation in ('N/A' 'None') then augmentation_ind = **0**; else augmentation_ind = **1**;

if lbr_time_sec_stg_total_minutes = **.** then labour_stage2_cat = 'No second stage';

else if lbr_time_sec_stg_total_minutes < **60** then labour_stage2_cat = '<60 minutes';

else if **60** <= lbr_time_sec_stg_total_minutes < **120** then labour_stage2_cat = '60-119 minutes';

else if **120** <= lbr_time_sec_stg_total_minutes < **180** then labour_stage2_cat = '120-179 minutes';

else if **180** <= lbr_time_sec_stg_total_minutes < **240** then labour_stage2_cat = '180-239 minutes';

else if **240** <= lbr_time_sec_stg_total_minutes then labour_stage2_cat = '>=240 minutes';

**run**;

**data** redpph.preg_cohort;

length mode_of_delivery $ **32.**;

set redpph.preg_cohort;

if birth_type = 'SVD' then mode_of_delivery = '(01) SVD';

else if birth_type = 'OVD' then do;

if forceps_vacuum = 'None' then mode_of_delivery = '(05) OVD none/unknown';

else if forceps_vacuum = 'N/A' then mode_of_delivery = '(05) OVD none/unknown';

else if forceps_vacuum = 'Vacuum' then mode_of_delivery = '(03) Vacuum delivery';

else if forceps_vacuum = 'Forceps' then mode_of_delivery = '(02) Forceps delivery';

else if forceps_vacuum = 'Forceps and Vacuum' then mode_of_delivery = '(04) Forceps and vacuum delivery';

end;

else do;

if birth_type = 'No labour CD' then mode_of_delivery = '(08) CD without labour';

else if birth_type = 'Induced/spontaneous labour CD' then do;

if cs_stage = 'First stage' then mode_of_delivery = '(06) First stage CD';

else if cs_stage = 'Second stage' then mode_of_delivery = '(07) Second stage CD';

else if cs_stage = 'N/A' then mode_of_delivery = '(09) Unknown stage CD';

else if cs_stage = 'No labour' then mode_of_delivery = '(08) CD without labour';

else if cs_stage = 'Perimortem' then mode_of_delivery = '(10) Perimortem CD';

end;

end;

if primary_language='Unknown/missing' and nat_language not in ('' '-1') then do;

if nat_language = '001' then primary_language = 'English';

else if nat_language = '002' then primary_language = 'French';

else primary_language = 'Other';

end;

if total_prenatal_visits ^= **.** then prenatal_visits_combo = total_prenatal_visits;

else prenatal_visits_combo = num_prenatal_visits_ohip;

if preex_dm = 'Gestational' or dad_gestdm_dxdate ^= **.** then gest_dm = **1**; else gest_dm = **0**;

if preg_hyper = 'Preeclampsia' or dad_preeclampsia_dxdate ^= **.** then preg_induced_hyper = 'Preeclampsia';

else if preg_hyper = 'HELLP' or dad_hellp_dxdate ^= **.** then preg_induced_hyper = 'HELLP';

else if preg_hyper = 'Eclampsia' then preg_induced_hyper = 'Eclampsia';

else if preg_hyper = 'Gestational' then preg_induced_hyper = 'Gestational';

else if preg_hyper = 'None' then preg_induced_hyper = 'None';

preg_startdate = b_bdate - (ga_at_birth_weeks***7**);

format preg_startdate date9.;

rename preex_dm = preg_dm;

**run**;

/* For pre-existing diabetes and hypertension: */

**proc** **sql**;

create table redpph.preg_cohort as

select a.*, b.preg_hypertension_disorder_id, b.diabetes_and_pregnancy_id

from redpph.preg_cohort as a left join born.agg_pregnancy as b

on a.pregnancy_id_enc = b.pregnancy_id_enc;

create table redpph.preg_cohort as

select a.*, b.antenatal_consult_reason_all_id

from redpph.preg_cohort as a left join born.ag as b

on a.pregnancy_id_enc = b.pregnancy_id_enc;

**quit**;

**data** redpph.preg_cohort;

set redpph.preg_cohort;

if odd_dxdate_general < preg_startdate or diabetes_and_pregnancy_id in ('1013500' '1013520' '1013550') then preex_dm = **1**; else preex_dm = **0**;

if hyper_dxdate < preg_startdate or preg_hypertension_disorder_id = '1020840' or index(antenatal_consult_reason_all_id, '1025170') > **0**

then preex_hyper = **1**; else preex_hyper = **0**;

**run**;

**proc** **sort** data=redpph.preg_cohort; by pregnancy_id_enc descending preex_hyper; **run**;

**proc** **sort** data=redpph.preg_cohort nodupkey out=redpph.preg_cohort(drop=antenatal_consult_reason_all_id); by pregnancy_id_enc; **run**;

**data** redpph.preg_cohort;

length b_weight_g_cat1 b_weight_g_cat2 b_weight_g_cat3 b_weight_g_cat4 $ **13.**;

length b_headcirc_cm_cat1 b_headcirc_cm_cat2 b_headcirc_cm_cat3 b_headcirc_cm_cat4 $ **9.**;

set redpph.preg_cohort;

array weight[**4**] b_weight_g_cat1-b_weight_g_cat4;

array circ[**4**] b_headcirc_cm_cat1-b_headcirc_cm_cat4;

do i = **1** to **4**;

if weight[i] = '<3000' then weight[i] = '(1) <3000';

else if weight[i] = '3000-3999' then weight[i] = '(2) 3000-3999';

else if weight[i] = '4000-4499' then weight[i] = '(3) 4000-4499';

else if weight[i] = '>=4500' then weight[i] = '(4) >=4500';

if circ[i] = '<33' then circ[i] = '(1) <33';

else if circ[i] = '33-34' then circ[i] = '(2) 33-34';

else if circ[i] = '35-36' then circ[i] = '(3) 35-36';

else if circ[i] = '>=37' then circ[i] = '(4) >=37';

end;

drop i;

**run**;

**data** redpph.preg_cohort;

length immig_cat2 $ **20.**;

set redpph.preg_cohort;

if immig = **0** then immig_cat2 = '(1) Not an immigrant';

else do;

if immigration_category in ('-1000001' '-1000002' '040' '043' '044' '045' '046' '050' '056' '060' '061' '062' '065' '067' '079'

'090' '091' '092' '093' '096' '097' '10138544' '10138549' '10138552' '10138579' '10138600' '10138605' '10138608' '10138609'

'10138639' '10138641' '10229825' '154' '155' '156' '157' '41731' '41732' '43705' '43707' '53005' '53007' '53027' '53028'

'7944' '7945' '7947' '7949' '7954' '7955' '7962' '8001' '8008' '8009') then immig_cat2 = '(2) Economic';

else if immigration_category in ('010' '011' '012' '013' '014' '015' '016' '017' '018' '019' '041' '042' '10138501' '10138503'

'10138504' '10138505' '10138507' '10138583' '10138584' '112' '114' '43702' '7910' '7912' '7913' '7914' '7915' '7916' '7917'

'7990' '7992' '8003') then immig_cat2 = '(3) Family';

else if immigration_category in ('030' '035' '036' '057' '098' '099' '10138545' '10138595' '10138597' '150' '151' '152' '8002'

'8004' '8005' '8006') then immig_cat2 = '(5) Other';

else if immigration_category in ('071') then immig_cat2 = '(6) Not stated';

else immig_cat2 = '(4) RR&PP';

end;

**run**;

**data** redpph.preg_cohort;

set redpph.preg_cohort;

if preg_dm in ('None' 'N/A' 'Gestational') and odd_dxdate_general = **.** then preex_dm = **0**;

else if preex_dm = **0** and preg_dm not in ('None' 'N/A' 'Gestational') then preex_dm = **1**;

if preg_hyper in ('None' 'N/A' 'Gestational') and hyper_dxdate = **.** then preex_hyper = **0**;

else if preex_hyper = **0** and preg_hyper not in ('None' 'N/A' 'Gestational') then preex_hyper = **1**;

if immig_cat2 = '(6) Not stated' then immig_cat2 = '(5) Other';

**run**;

/* Pull obstetric trauma information: */

**data** preg_cohort;

set redpph.preg_cohort;

keep pregnancy_id_enc ikn b_bdate;

**run**;

%***getdadsds***(

source = inpat,

start = **20110601**,

end = **20210430**,

refdate = admdate,

cohort = preg_cohort,

out = dad_obtrauma,

where = %str(admdate <= b_bdate <= ddate),

dxtype = all,

dx10code = ('O702' 'O703' 'O713' 'O714' 'O715' 'O716' 'O717' 'O718' 'O719'),

incode = ('5PC80JQ' '5PC80JJ' '5PC80JU' '5PC80JR' '5PC80JH' '5PC80JK' '5PC80JL'),

keep = ikn admdate ddate,

inclsuspect = F);

**proc** **sort** data=dad_obtrauma; by pregnancy_id_enc admdate; **run**;

**proc** **sort** data=dad_obtrauma nodupkey out=dad_obtrauma(keep=pregnancy_id_enc admdate ddate); by pregnancy_id_enc; **run**;

%***getohip***(

source = nonlab,

start = **20110601**,

end = **20210430**,

cohort = preg_cohort,

out = ohip_obtrauma,

where = %str((b_bdate - **3**) <= servdate <= (b_bdate + **3**) and feecode in ('P045' 'P046' 'P039' 'P036')),

keep = ikn physnum servdate feecode);

**proc** **sort** data=ohip_obtrauma; by pregnancy_id_enc servdate; **run**;

**proc** **sort** data=ohip_obtrauma nodupkey out=ohip_obtrauma(keep=pregnancy_id_enc servdate); by pregnancy_id_enc; **run**;

**proc** **sql**;

create table born_obtrauma as

select a.pregnancy_id_enc, b.perineal_laceration_id

from preg_cohort as a left join born.agg_pregnancy as b

on a.pregnancy_id_enc = b.pregnancy_id_enc;

create table born_obtrauma as

select a.pregnancy_id_enc, b.intrapartum_cons_reas_id

from born_obtrauma as a left join born.mw_pregnancy_coc as b

on a.pregnancy_id_enc = b.pregnancy_id_enc;

**quit**;

**data** born_obtrauma;

set born_obtrauma;

if perineal_laceration_id in: ('1019960' '1019970' '1019980') or intrapartum_cons_reas_id in: ('1017902' '1017903' '1017904')

then born_obtrauma = **1**; else born_obtrauma = **0**;

drop perineal_laceration_id intrapartum_cons_reas_id;

**run**;

**proc** **sort** data=born_obtrauma; by pregnancy_id_enc descending born_obtrauma; **run**;

**proc** **sort** data=born_obtrauma nodupkey; by pregnancy_id_enc; **run**;

**data** ob_trauma;

merge born_obtrauma(in=a) dad_obtrauma(in=b) ohip_obtrauma(in=c);

by pregnancy_id_enc;

if a and b then dad_obtrauma = **1**; else dad_obtrauma = **0**;

if a and c then ohip_obtrauma = **1**; else ohip_obtrauma = **0**;

if born_obtrauma = **1** or dad_obtrauma = **1** or ohip_obtrauma = **1** then ob_trauma = **1**; else ob_trauma = **0**;

drop admdate ddate servdate;

**run**;

**data** redpph.preg_cohort;

merge redpph.preg_cohort ob_trauma;

by pregnancy_id_enc;

**run**;

/* Format baseline characteristics table: */

**data** preg_cohort;

set redpph.preg_cohort;

array exist[**15**] plurality smoking_firstvisit smoking_atbirth preg_drug_exposure tri1_prenatal_visit preex_dm gest_dm preex_hyper

previous_cd placenta_previa placenta_accspec placental_abruption induction augmentation_ind ob_trauma;

array new[**15**] $ plural smoking1 smoking2 drugexpose tri1visit dm1 dm2 hyper1 prevcd previa accspec abruption induct augment obtrauma;

do i = **1** to **15**;

if exist[i] = **1** then new[i] = '(1) Y'; else if exist[i] = **0** then new[i] = '(2) N';

end;

drop i;

**run**;

%***bivartab***(

data = preg_cohort,

byvar = ancestry,

numdq = miss_only,

catvar = age_group parity_cat plural primary_language maternal_bmi_cat age_labourforce_q_da material_resources_q_da

households_dwellings_q_da immig_cat2 immig_birth_datediff_cat rural_pccf smoking1 smoking2 drugexpose conception_type

tri1visit dm1 dm2 hyper1 preg_induced_hyper prevcd previa accspec abruption induct augment episiotomy presentation_type

labour_stage2_cat mode_of_delivery obtrauma gestage_cat b_weight_g_cat1 b_weight_g_cat2 b_weight_g_cat3 b_weight_g_cat4

b_headcirc_cm_cat1 b_headcirc_cm_cat2 b_headcirc_cm_cat3 b_headcirc_cm_cat4,

numvar = immig_birth_datediff prenatal_visits_combo lbr_time_sec_stg_total_minutes,

order = age_group parity_cat plural primary_language maternal_bmi_cat age_labourforce_q_da material_resources_q_da

households_dwellings_q_da immig_cat2 immig_birth_datediff immig_birth_datediff_cat rural_pccf smoking1 smoking2

drugexpose conception_type tri1visit prenatal_visits_combo dm1 dm2 hyper1 preg_induced_hyper prevcd previa accspec

abruption induct augment episiotomy presentation_type lbr_time_sec_stg_total_minutes labour_stage2_cat mode_of_delivery

obtrauma gestage_cat b_weight_g_cat1 b_weight_g_cat2 b_weight_g_cat3 b_weight_g_cat4 b_headcirc_cm_cat1 b_headcirc_cm_cat2

b_headcirc_cm_cat3 b_headcirc_cm_cat4,

total = T,

small = T,

stdiff = F,

trend = F,

out = table1);

**data** table1;

retain varlabel varvalue Black Caucasian Asian Other Unknown_missing _Total;

set table1;

if VarValue = '' then delete;

keep varlabel varvalue Black Caucasian Asian Other Unknown_missing _Total;

**run**;

**data** table1;

set table1;

if _n_ = **52** or _n_ = **58** or _n_ = **79** or _n_ = **114** then delete;

**run**;

**data** table1;

set table1;

if varvalue = '<20 - n (%)' then place = _n_ - **5**;

else if varvalue in ('20-24 - n (%)' '25-29 - n (%)' '30-34 - n (%)' '35-39 - n (%)' '40+ - n (%)') then place = _n_ + **1**;

else if varvalue = '<18.5 - n (%)' then place = _n_ - **4**;

else if varvalue in ('18.5-24.9 - n (%)' '25-29.9 - n (%)' '30-34.9 - n (%)' '35-39.9 - n (%)') then place = _n_ + **1**;

else if varvalue = 'Breech - n (%)' then place = _n_ + **1**;

else if varvalue = 'Cephalic - n (%)' then place = _n_ - **1**;

else if varvalue = '<60 minutes - n (%)' then place = _n_ - **3**;

else if varvalue = '60-119 minutes - n (%)' then place = _n_ - **1**;

else if varvalue in ('120-179 minutes - n (%)' '180-239 minutes - n (%)') then place = _n_ + **2**;

else if varvalue = 'Spontaneous - n (%)' then place = _n_ - **1**;

else if varvalue = 'Assisted - n (%)' then place = _n_ + **1**;

else if varlabel = 'preg_induced_hyper' then place = _n_ + **5**;

else if varvalue = 'Preeclampsia - n (%)' then place = _n_ - **5**;

else if varvalue = 'HELLP - n (%)' then place = _n_ - **1**;

else if varvalue = 'Gestational - n (%)' then place = _n_ + **1**;

else place = _n_;

**run**;

**proc** **sort** data=table1 out=tables.table1(drop=place); by place; **run**;

**data** nulli parous;

set preg_cohort;

if parity = **0** then output nulli;

else if parity ^= **.** then output parous;

**run**;

%***bivartab***(

data = nulli,

byvar = ancestry,

numdq = miss_only,

catvar = age_group plural primary_language maternal_bmi_cat age_labourforce_q_da material_resources_q_da

households_dwellings_q_da immig_cat2 immig_birth_datediff_cat rural_pccf smoking1 smoking2 drugexpose conception_type

tri1visit dm1 dm2 hyper1 preg_induced_hyper prevcd previa accspec abruption induct augment episiotomy presentation_type

labour_stage2_cat mode_of_delivery obtrauma gestage_cat b_weight_g_cat1 b_weight_g_cat2 b_weight_g_cat3 b_weight_g_cat4

b_headcirc_cm_cat1 b_headcirc_cm_cat2 b_headcirc_cm_cat3 b_headcirc_cm_cat4,

numvar = immig_birth_datediff prenatal_visits_combo lbr_time_sec_stg_total_minutes,

order = age_group plural primary_language maternal_bmi_cat age_labourforce_q_da material_resources_q_da

households_dwellings_q_da immig_cat2 immig_birth_datediff immig_birth_datediff_cat rural_pccf smoking1 smoking2

drugexpose conception_type tri1visit prenatal_visits_combo dm1 dm2 hyper1 preg_induced_hyper prevcd previa accspec

abruption induct augment episiotomy presentation_type lbr_time_sec_stg_total_minutes labour_stage2_cat mode_of_delivery

obtrauma gestage_cat b_weight_g_cat1 b_weight_g_cat2 b_weight_g_cat3 b_weight_g_cat4 b_headcirc_cm_cat1 b_headcirc_cm_cat2

b_headcirc_cm_cat3 b_headcirc_cm_cat4,

total = T,

small = T,

stdiff = F,

trend = F,

out = table1);

**data** table1;

retain varlabel varvalue Black Caucasian Asian Other Unknown_missing _Total;

set table1;

if VarValue = '' then delete;

keep varlabel varvalue Black Caucasian Asian Other Unknown_missing _Total;

rename Black = Black_nulli Caucasian = Caucasian_nulli Asian = Asian_nulli Other = Other_nulli Unknown_missing = Unknown_missing_nulli _Total = Total_nulli;

**run**;

**data** table1;

set table1;

if _n_ = **47** or _n_ = **53** or _n_ = **74** or _n_ = **109** then delete;

**run**;

**data** table1;

set table1;

if varvalue = '<20 - n (%)' then place = _n_ - **5**;

else if varvalue in ('20-24 - n (%)' '25-29 - n (%)' '30-34 - n (%)' '35-39 - n (%)' '40+ - n (%)') then place = _n_ + **1**;

else if varvalue = '<18.5 - n (%)' then place = _n_ - **4**;

else if varvalue in ('18.5-24.9 - n (%)' '25-29.9 - n (%)' '30-34.9 - n (%)' '35-39.9 - n (%)') then place = _n_ + **1**;

else if varvalue = 'Breech - n (%)' then place = _n_ + **1**;

else if varvalue = 'Cephalic - n (%)' then place = _n_ - **1**;

else if varvalue = '<60 minutes - n (%)' then place = _n_ - **3**;

else if varvalue = '60-119 minutes - n (%)' then place = _n_ - **1**;

else if varvalue in ('120-179 minutes - n (%)' '180-239 minutes - n (%)') then place = _n_ + **2**;

else if varvalue = 'Spontaneous - n (%)' then place = _n_ - **1**;

else if varvalue = 'Assisted - n (%)' then place = _n_ + **1**;

else if varlabel = 'preg_induced_hyper' then place = _n_ + **5**;

else if varvalue = 'Preeclampsia - n (%)' then place = _n_ - **5**;

else if varvalue = 'HELLP - n (%)' then place = _n_ - **1**;

else if varvalue = 'Gestational - n (%)' then place = _n_ + **1**;

else place = _n_;

**run**;

**proc** **sort** data=table1 out=table1_nulli(drop=place); by place; **run**;

**data** table1_nulli;

set table1_nulli;

if _n_ < **135** then place = _n_;

else if **135** <= _n_ < **137** then place = _n_ + **1**;

else if **137** <= _n_ < **139** then place = _n_ + **4**;

else if **139** <= _n_ < **152** then place = _n_ + **7**;

else if **152** <= _n_ < **154** then place = _n_ + **8**;

else if _n_ = **154** then place = _n_ + **12**;

**run**;

%***bivartab***(

data = parous,

byvar = ancestry,

numdq = miss_only,

catvar = age_group plural primary_language maternal_bmi_cat age_labourforce_q_da material_resources_q_da

households_dwellings_q_da immig_cat2 immig_birth_datediff_cat rural_pccf smoking1 smoking2 drugexpose conception_type

tri1visit dm1 dm2 hyper1 preg_induced_hyper prevcd previa accspec abruption induct augment episiotomy presentation_type

labour_stage2_cat mode_of_delivery obtrauma gestage_cat b_weight_g_cat1 b_weight_g_cat2 b_weight_g_cat3 b_weight_g_cat4

b_headcirc_cm_cat1 b_headcirc_cm_cat2 b_headcirc_cm_cat3 b_headcirc_cm_cat4,

numvar = immig_birth_datediff prenatal_visits_combo lbr_time_sec_stg_total_minutes,

order = age_group plural primary_language maternal_bmi_cat age_labourforce_q_da material_resources_q_da

households_dwellings_q_da immig_cat2 immig_birth_datediff immig_birth_datediff_cat rural_pccf smoking1 smoking2

drugexpose conception_type tri1visit prenatal_visits_combo dm1 dm2 hyper1 preg_induced_hyper prevcd previa accspec

abruption induct augment episiotomy presentation_type lbr_time_sec_stg_total_minutes labour_stage2_cat mode_of_delivery

obtrauma gestage_cat b_weight_g_cat1 b_weight_g_cat2 b_weight_g_cat3 b_weight_g_cat4 b_headcirc_cm_cat1 b_headcirc_cm_cat2

b_headcirc_cm_cat3 b_headcirc_cm_cat4,

total = T,

small = T,

stdiff = F,

trend = F,

out = table1);

**data** table1;

retain varlabel varvalue Black Caucasian Asian Other Unknown_missing _Total;

set table1;

if VarValue = '' then delete;

keep varlabel varvalue Black Caucasian Asian Other Unknown_missing _Total;

rename Black = Black_parous Caucasian = Caucasian_parous Asian = Asian_parous Other = Other_parous Unknown_missing = Unknown_missing_parous _Total = Total_parous;

**run**;

**data** table1;

set table1;

if _n_ = **47** or _n_ = **53** or _n_ = **74** or _n_ = **109** then delete;

**run**;

**data** table1;

set table1;

if varvalue = '<20 - n (%)' then place = _n_ - **5**;

else if varvalue in ('20-24 - n (%)' '25-29 - n (%)' '30-34 - n (%)' '35-39 - n (%)' '40+ - n (%)') then place = _n_ + **1**;

else if varvalue = '<18.5 - n (%)' then place = _n_ - **4**;

else if varvalue in ('18.5-24.9 - n (%)' '25-29.9 - n (%)' '30-34.9 - n (%)' '35-39.9 - n (%)') then place = _n_ + **1**;

else if varvalue = 'Breech - n (%)' then place = _n_ + **1**;

else if varvalue = 'Cephalic - n (%)' then place = _n_ - **1**;

else if varvalue = '<60 minutes - n (%)' then place = _n_ - **3**;

else if varvalue = '60-119 minutes - n (%)' then place = _n_ - **1**;

else if varvalue in ('120-179 minutes - n (%)' '180-239 minutes - n (%)') then place = _n_ + **2**;

else if varvalue = 'Spontaneous - n (%)' then place = _n_ - **1**;

else if varvalue = 'Assisted - n (%)' then place = _n_ + **1**;

else if varlabel = 'preg_induced_hyper' then place = _n_ + **5**;

else if varvalue = 'Preeclampsia - n (%)' then place = _n_ - **5**;

else if varvalue = 'HELLP - n (%)' then place = _n_ - **1**;

else if varvalue = 'Gestational - n (%)' then place = _n_ + **1**;

else place = _n_;

**run**;

**proc** **sort** data=table1 out=table1_parous(drop=place); by place; **run**;

**data** table1_parous;

set table1_parous;

if _n_ < **139** then place = _n_;

else if **139** <= _n_ < **141** then place = _n_ + **2**;

else if **141** <= _n_ < **154** then place = _n_ + **5**;

else if **154** = _n_ then place = _n_ + **7**;

else if **155** = _n_ then place = _n_ + **11**;

**run**;

**data** tables.table1_parity;

merge table1_nulli table1_parous;

by place;

drop place;

**run**;

/* BORN variables: */

**proc** **sql**;

create table born_outcomes as

select a.pregnancy_id_enc, b.b_btime, b.lbr_and_birth_complication_id, b.maternal_outcome_id

from redpph.preg_cohort as a left join born.agg_pregnancy as b

on a.pregnancy_id_enc = b.pregnancy_id_enc;

**quit**;

**data** born_outcomes;

set born_outcomes;

if lbr_and_birth_complication_id in: ('1014540') then born_pph = **1**; else born_pph = **0**;

if lbr_and_birth_complication_id in: ('1014475' '1020090') then born_pph_hyst = **1**; else born_pph_hyst = **0**;

if maternal_outcome_id = '1016500' then born_icu_transfer = **1**; else born_icu_transfer = **0**;

if maternal_outcome_id in: ('1016480' '1016470') then born_mat_dth = **1**; else born_mat_dth = **0**;

drop lbr_and_birth_complication_id maternal_outcome_id;

**run**;

**proc** **sql**;

create table born_ppm as

select a.pregnancy_id_enc, b.postpartum_complication_id

from redpph.preg_cohort as a left join born.ppm as b

on a.pregnancy_id_enc = b.pregnancy_id_enc;

create table born_preg_coc as

select a.pregnancy_id_enc, b.postpartum_complication_id

from redpph.preg_cohort as a left join born.mw_pregnancy_coc as b

on a.pregnancy_id_enc = b.pregnancy_id_enc;

**quit**;

**data** ppm_comps;

set born_ppm born_preg_coc;

if postpartum_complication_id in: ('3100563') then born_pph_btrans = **1**; else born_pph_btrans = **0**;

drop postpartum_complication_id;

**run**;

**proc** **sort** data=ppm_comps; by pregnancy_id_enc descending born_pph_btrans; **run**;

**proc** **sort** data=ppm_comps nodupkey; by pregnancy_id_enc; **run**;

**data** born_outcomes;

merge born_outcomes ppm_comps;

by pregnancy_id_enc;

**run**;

/* DAD outcomes: */

**data** iknlist;

set redpph.preg_cohort;

lf6wks = b_bdate + **6*****7**;

b_bdatetime = dhms(b_bdate, hour(b_btime), minute(b_btime), second(b_btime));

keep pregnancy_id_enc ikn b_bdate lf6wks b_bdatetime;

format lf6wks date9.;

format b_bdatetime DATETIME15.;

**run**;

%***getdadsds***(

source = inpat,

start = **20120401**,

end = **20210531**,

refdate = admdate,

cohort = iknlist,

out = dad_outcomes,

where = %str(admdate <= b_bdate <= ddate or b_bdate <= admdate <= lf6wks),

inclsuspect = F);

**data** dad_outcomes2;

retain ikn pregnancy_id_enc b_bdate lf6wks admdate ddate pph: bloodtrans hysterectomy hystcond hystexcl bldctrl btredbc;

set dad_outcomes;

pph = **0**;

bloodtrans = **0**;

hysterectomy = **0**;

hystcond = **0**;

hystexcl = **0**;

bldctrl = **0**;

array icd[**25**] dx10code1-dx10code25;

do i = **1** to **25**;

if substr(icd[i], **1**, **3**) = 'O72' then pph + **1**;

end;

array cci[**20**] incode1-incode20;

do j = **1** to **20**;

if substr(cci[j], **1**, **5**) = '1LZ19' then bloodtrans + **1**;

if substr(cci[j], **1**, **7**) in ('5MD60KE' '5MD60RC' '5MD60CB' '5MD60RD' '1RM89LA') or substr(cci[j], **1**, **9**) = '1RM87LAGX' then hysterectomy + **1**;

if substr(cci[j], **1**, **7**) = '1RM89LA' then hystcond + **1**;

if substr(cci[j], **1**, **5**) in ('1PL74' '1RS80' '1RS74') then hystexcl + **1**;

if substr(cci[j], **1**, **5**) in ('1RM13' '1KT51') or substr(cci[j], **1**, **7**) in ('5PC91LA' '5PC91HT') then bldctrl + **1**;

end;

if pph > **0** then do;

if btredbc = '1' or bloodtrans > **0** then pph_btrans = **1**;

else pph_btrans = **0**;

if hysterectomy > **0** then pph_hyst = **1**;

else if hystcond > **0** and hystexcl = **0** then pph_hyst = **1**;

else pph_hyst = **0**;

if btredbc = '1' and bldctrl > **0** then pph_bldctrl = **1**;

else pph_bldctrl = **0**;

end;

else do;

pph_btrans = **0**;

pph_hyst = **0**;

pph_bldctrl = **0**;

end;

keep ikn pregnancy_id_enc b_bdate lf6wks admdate ddate pph: bloodtrans hysterectomy hystcond hystexcl bldctrl btredbc;

**run**;

**proc** **freq** data=dad_outcomes2;

tables pph pph_bldctrl pph_btrans pph_hyst;

**run**;

**data** pph(drop=pph_btrans pph_hyst pph_bldctrl rename=(admdate=pph_admdate ddate=pph_ddate))

pph_btrans(drop=pph pph_hyst pph_bldctrl rename=(admdate=pph_btrans_admdate ddate=pph_btrans_ddate))

pph_hyst(drop=pph pph_btrans pph_bldctrl rename=(admdate=pph_hyst_admdate ddate=pph_hyst_ddate))

pph_bldctrl(drop=pph pph_btrans pph_hyst rename=(admdate=pph_bldctrl_admdate ddate=pph_bldctrl_ddate));

set dad_outcomes2;

if pph > **0** then output pph;

if pph_btrans > **0** then output pph_btrans;

if pph_hyst > **0** then output pph_hyst;

if pph_bldctrl > **0** then output pph_bldctrl;

drop lf6wks bloodtrans hysterectomy hystexcl hystcond hystexcl bldctrl btredbc;

**run**;

**proc** **sql**;

create table pph_hosp_count as

select pregnancy_id_enc, count(unique pph_admdate) as num_hosp

from pph

group by pregnancy_id_enc;

**quit**;

**proc** **freq** data=pph_hosp_count;

tables num_hosp;

**run**;

**proc** **sort** data=pph; by pregnancy_id_enc pph_admdate descending pph_ddate; **run**;

**proc** **sort** data=pph nodupkey; by pregnancy_id_enc; **run**;

**proc** **sort** data=pph_btrans; by pregnancy_id_enc pph_btrans_admdate descending pph_btrans_ddate; **run**;

**proc** **sort** data=pph_btrans nodupkey; by pregnancy_id_enc; **run**;

**proc** **sort** data=pph_hyst; by pregnancy_id_enc pph_hyst_admdate descending pph_hyst_ddate; **run**;

**proc** **sort** data=pph_hyst nodupkey; by pregnancy_id_enc; **run**;

**proc** **sort** data=pph_bldctrl; by pregnancy_id_enc pph_bldctrl_admdate descending pph_bldctrl_ddate; **run**;

**proc** **sort** data=pph_bldctrl nodupkey; by pregnancy_id_enc; **run**;

**data** pph;

merge pph pph_btrans pph_hyst pph_bldctrl;

by pregnancy_id_enc;

if pph > **1** then pph = **1**;

**run**;

**data** pph;

merge born_outcomes pph;

by pregnancy_id_enc;

if pph = **.** then pph = **0**;

if pph_btrans = **.** then pph_btrans = **0**;

if pph_hyst = **.** then pph_hyst = **0**;

if pph_bldctrl = **.** then pph_bldctrl = **0**;

drop ikn b_bdate;

**run**;

**proc** **sql**;

create table redpph.preg_outcomes as

select a.pregnancy_id_enc, a.ikn, a.b_bdate, a.ancestry, b.*

from redpph.preg_cohort as a left join pph as b

on a.pregnancy_id_enc = b.pregnancy_id_enc;

**quit**;

**data** redpph.preg_outcomes;

set redpph.preg_outcomes;

if born_pph = **1** or pph = **1** then born_dad_pph = **1**; else born_dad_pph = **0**;

if born_pph_hyst = **1** or pph_hyst = **1** then born_dad_pph_hyst = **1**; else born_dad_pph_hyst = **0**;

if born_pph_btrans = **1** or pph_btrans = **1** then born_dad_pph_btrans = **1**; else born_dad_pph_btrans = **0**;

if born_dad_pph_hyst = **1** or born_dad_pph_btrans = **1** or pph_bldctrl = **1** then pph_severe = **1**; else pph_severe = **0**;

**run**;

**data** icu1(keep=pregnancy_id_enc ikn admdate ddate b_bdate b_bdatetime lf6wks scu1 scuadmdate1 scuddate1 scuadmtime1 scudtime1)

icu2(keep=pregnancy_id_enc ikn admdate ddate b_bdate b_bdatetime lf6wks scu2 scuadmdate2 scuddate2 scuadmtime2 scudtime2)

icu3(keep=pregnancy_id_enc ikn admdate ddate b_bdate b_bdatetime lf6wks scu3 scuadmdate3 scuddate3 scuadmtime3 scudtime3)

icu4(keep=pregnancy_id_enc ikn admdate ddate b_bdate b_bdatetime lf6wks scu4 scuadmdate4 scuddate4 scuadmtime4 scudtime4)

icu5(keep=pregnancy_id_enc ikn admdate ddate b_bdate b_bdatetime lf6wks scu5 scuadmdate5 scuddate5 scuadmtime5 scudtime5)

icu6(keep=pregnancy_id_enc ikn admdate ddate b_bdate b_bdatetime lf6wks scu6 scuadmdate6 scuddate6 scuadmtime6 scudtime6);

set dad_outcomes;

if scu1 in ('10' '20' '25' '30' '35' '40' '45' '60' '80') then output icu1;

if scu2 in ('10' '20' '25' '30' '35' '40' '45' '60' '80') then output icu2;

if scu3 in ('10' '20' '25' '30' '35' '40' '45' '60' '80') then output icu3;

if scu4 in ('10' '20' '25' '30' '35' '40' '45' '60' '80') then output icu4;

if scu5 in ('10' '20' '25' '30' '35' '40' '45' '60' '80') then output icu5;

if scu6 in ('10' '20' '25' '30' '35' '40' '45' '60' '80') then output icu6;

**run**;

**proc** **sort** data=icu1; by pregnancy_id_enc admdate ddate; **run**;

**proc** **sort** data=icu2; by pregnancy_id_enc admdate ddate; **run**;

**proc** **sort** data=icu3; by pregnancy_id_enc admdate ddate; **run**;

**proc** **sort** data=icu4; by pregnancy_id_enc admdate ddate; **run**;

**proc** **sort** data=icu5; by pregnancy_id_enc admdate ddate; **run**;

**proc** **sort** data=icu6; by pregnancy_id_enc admdate ddate; **run**;

**data** dad_icu;

retain ikn pregnancy_id_enc admdate ddate b_bdate b_bdatetime lf6wks;

merge icu1 icu2 icu3 icu4 icu5 icu6;

by pregnancy_id_enc admdate ddate;

array admtime[**6**] scuadmtime1-scuadmtime6;

array dtime[**6**] scudtime1-scudtime6;

array icudays[**6**] icudays1 icudays2 icudays3 icudays4 icudays5 icudays6;

array icutime[**6**] icuhrs1 icuhrs2 icuhrs3 icuhrs4 icuhrs5 icuhrs6;

array deldiff[**6**] icudeldiff1 icudeldiff2 icudeldiff3 icudeldiff4 icudeldiff5 icudeldiff6;

array post[**6**] post24hrs1 post24hrs2 post24hrs3 post24hrs4 post24hrs5 post24hrs6;

array prohrs[**6**] proicuhrs1 proicuhrs2 proicuhrs3 proicuhrs4 proicuhrs5 proicuhrs6;

array prodays[**6**] proicudays1 proicudays2 proicudays3 proicudays4 proicudays5 proicudays6;

do i = **1** to **6**;

icutime[i] = (dtime[i] - admtime[i])/(**60*****60**);

icudays[i] = (dtime[i] - admtime[i])/(**60*****60*****24**);

deldiff[i] = (admtime[i] - b_bdatetime)/(**60*****60**);

if deldiff[i] > **24** then post[i] = **1**; else post[i] = **0**;

prohrs[i] = post[i]*icutime[i];

prodays[i] = post[i]*icudays[i];

end;

total_icuhrs = sum(icuhrs1, icuhrs2, icuhrs3, icuhrs4, icuhrs5, icuhrs6);

total_icudays = sum(icudays1, icudays2, icudays3, icudays4, icudays5, icudays6);

total_prohrs = sum(proicuhrs1, proicuhrs2, proicuhrs3, proicuhrs4, proicuhrs5, proicuhrs6);

total_prodays = sum(proicudays1, proicudays2, proicudays3, proicudays4, proicudays5, proicudays6);

if admdate <= b_bdate <= ddate then delivery = **1**; else delivery = **0**;

drop i;

**run**;

**proc** **sql**;

create table icu_total as

select pregnancy_id_enc, delivery, sum(total_icuhrs) as total_icu_hrs,

sum(total_icudays) as total_icu_days,

sum(total_prohrs) as total_pro_icu_hrs,

sum(total_prodays) as total_pro_icu_days

from dad_icu

group by pregnancy_id_enc, delivery;

**quit**;

**data** delivery(rename=(total_icu_hrs=del_icu_hrs total_icu_days=del_icu_days total_pro_icu_hrs=del_pro_icu_hrs total_pro_icu_days=del_pro_icu_days))

post6wks(rename=(total_icu_hrs=lf6wk_icu_hrs total_icu_days=lf6wk_icu_days total_pro_icu_hrs=lf6wk_pro_icu_hrs total_pro_icu_days=lf6wk_pro_icu_days));

set icu_total;

if delivery = **1** then output delivery;

else output post6wks;

drop delivery;

**run**;

**data** icu_total;

merge delivery post6wks;

by pregnancy_id_enc;

total_icu_hrs = sum(del_icu_hrs, lf6wk_icu_hrs);

total_icu_days = sum(del_icu_days, lf6wk_icu_days);

total_pro_icu_hrs = sum(del_pro_icu_hrs, lf6wk_pro_icu_hrs);

total_pro_icu_days = sum(del_pro_icu_days, lf6wk_pro_icu_days);

**run**;

**data** redpph.preg_outcomes;

merge redpph.preg_outcomes icu_total;

by pregnancy_id_enc;

if del_icu_hrs ^= **.** then icu_delivery = **1**; else icu_delivery = **0**;

if lf6wk_icu_hrs ^= **.** then icu_6wkpostdel = **1**; else icu_6wkpostdel = **0**;

if del_pro_icu_hrs ^= **.** then pro_icu_delivery = **1**; else pro_icu_delivery = **0**;

if lf6wk_pro_icu_hrs ^= **.** then pro_icu_6wkpostdel = **1**; else pro_icu_6wkpostdel = **0**;

if icu_delivery = **1** or icu_6wkpostdel = **1** then icu_any = **1**; else icu_any = **0**;

if pro_icu_delivery = **1** or pro_icu_6wkpostdel = **1** then pro_icu_any = **1**; else pro_icu_any = **0**;

**run**;

**data** dad_los;

set dad_outcomes;

if admdate <= b_bdate <= ddate then delivery = **1**; else delivery = **0**;

keep pregnancy_id_enc ikn b_bdate admdate ddate los delivery;

**run**;

**data** dad_pro_los;

set dad_los;

if los >= **7**;

**run**;

**proc** **sort** data=dad_pro_los nodupkey; by pregnancy_id_enc delivery; **run**;

**data** delivery post;

set dad_pro_los;

if delivery = **1** then output delivery;

else output post;

keep pregnancy_id_enc;

**run**;

**data** dad_pro_los;

merge delivery(in=a) post(in=b);

by pregnancy_id_enc;

if a then prolonged_los_del = **1**;

if b then prolonged_los_lf6wk = **1**;

**run**;

**data** redpph.preg_outcomes;

merge redpph.preg_outcomes dad_pro_los;

by pregnancy_id_enc;

if prolonged_los_del = **.** then prolonged_los_del = **0**;

if prolonged_los_lf6wk = **.** then prolonged_los_lf6wk = **0**;

if prolonged_los_del = **1** or prolonged_los_lf6wk = **1** then prolonged_los_any = **1**;

else prolonged_los_any = **0**;

**run**;

**proc** **sort** data=dad_los; by pregnancy_id_enc admdate descending ddate; **run**;

**proc** **sort** data=dad_los nodupkey; by pregnancy_id_enc admdate; **run**;

%***getnacrs***(

source = ed,

start = **20120401**,

end = **20210531**,

cohort = iknlist,

out = nacrs_lf6wks,

where = %str(b_bdate <= regdate <= lf6wks),

inclscheduled = T,

inclfrom_typee = T,

inclto_typeip = T,

inclnotseen = F,

inclsuspect = F);

**proc** **sort** data=nacrs_lf6wks nodupkey out=nacrs_lf6wks(keep=pregnancy_id_enc ikn regdate); by pregnancy_id_enc regdate; **run**;

**data** day1;

set iknlist;

day = b_bdate;

keep pregnancy_id_enc ikn b_bdate lf6wks day;

format day date9.;

**run**;

**%macro** lf6wks();

%do i = **2** %to **43**;

%let j = %eval(&i. - 1);

data day&i.;

set day&j.;

day + **1**;

run;

%end;

data lf6wks; set day1-day43; run;

proc sort data=lf6wks; by pregnancy_id_enc day; run;

proc datasets; delete day1-day43; run;

**%mend**;

%***lf6wks***();

**proc** **sql**;

create table lf6wks as

select a.*, b.admdate, b.ddate

from lf6wks as a left join dad_los as b

on a.pregnancy_id_enc = b.pregnancy_id_enc and b.admdate <= a.day <= b.ddate;

create table lf6wks as

select a.*, b.regdate

from lf6wks as a left join nacrs_lf6wks as b

on a.pregnancy_id_enc = b.pregnancy_id_enc and a.day = b.regdate;

**quit**;

**data** lf6wks;

set lf6wks;

if admdate ^= **.** then hosp = **1**; else hosp = **0**;

if regdate ^= **.** then ed = **1**; else ed = **0**;

if hosp = **1** or ed = **1** then hosp_ed = **1**; else hosp_ed = **0**;

if admdate <= b_bdate <= ddate then delivery = **1**;

else if b_bdate = regdate then delivery = **1**;

else delivery = **0**;

**run**;

**proc** **sort** data=lf6wks; by pregnancy_id_enc day descending delivery; **run**;

**proc** **sort** data=lf6wks nodupkey; by pregnancy_id_enc day; **run**;

**proc** **sql**;

create table num_hosp_ed_days as

select pregnancy_id_enc, delivery, sum(hosp) as num_hosp_days, sum(ed) as num_ed_days, sum(hosp_ed) as num_hosp_ed_days

from lf6wks

group by pregnancy_id_enc, delivery;

**quit**;

**data** delivery(rename=(num_hosp_days=num_hosp_days_del num_ed_days=num_ed_days_del num_hosp_ed_days=num_hosp_ed_days_del))

post6wks(rename=(num_hosp_days=num_hosp_days_lf6wks num_ed_days=num_ed_days_lf6wks num_hosp_ed_days=num_hosp_ed_days_lf6wks));

set num_hosp_ed_days;

if delivery = **1** then output delivery;

else output post6wks;

drop delivery;

**run**;

**data** hosp_ed;

merge delivery(in=a) post6wks(in=b);

by pregnancy_id_enc;

if a and not b then do;

num_hosp_days_lf6wks = **0**;

num_ed_days_lf6wks = **0**;

num_hosp_ed_days_lf6wks = **0**;

end;

if b and not a then do;

num_hosp_days_del = **0**;

num_ed_days_del = **0**;

num_hosp_ed_days_del = **0**;

end;

total_hosp_days = sum(num_hosp_days_del, num_hosp_days_lf6wks);

total_ed_days = sum(num_ed_days_del, num_ed_days_lf6wks);

total_hosp_ed_days = sum(num_hosp_ed_days_del, num_hosp_ed_days_lf6wks);

**run**;

**proc** **sql**;

create table redpph.preg_outcomes as

select * from redpph.preg_outcomes as a left join hosp_ed as b

on a.pregnancy_id_enc = b.pregnancy_id_enc;

**quit**;

/* Maternal mortality within 1 year of delivery: */

**data** mortality;

set redpph.preg_cohort;

lf1yr = intnx("year", b_bdate, **1**, "s");

if dthdate ^= **.** and b_bdate <= dthdate <= lf1yr then dth_1yr = **1**;

else dth_1yr = **0**;

**run**;

**proc** **sql**;

create table redpph.preg_outcomes as

select a.*, b.dthdate, b.dth_1yr

from redpph.preg_outcomes as a left join mortality as b

on a.pregnancy_id_enc = b.pregnancy_id_enc;

**quit**;

**data** redpph.preg_outcomes;

set redpph.preg_outcomes;

if num_hosp_days_del = **0** then hosp_adm_del = **0**; else hosp_adm_del = **1**;

if num_hosp_days_lf6wks = **0** then hosp_adm_lf6wks = **0**; else hosp_adm_lf6wks = **1**;

if hosp_adm_del = **1** or hosp_adm_lf6wks = **1** then hosp_adm_any = **1**; else hosp_adm_any = **0**;

if num_ed_days_del = **0** then ed_visit_del = **0**; else ed_visit_del = **1**;

if num_ed_days_lf6wks = **0** then ed_visit_lf6wks = **0**; else ed_visit_lf6wks = **1**;

if num_hosp_ed_days_del = **0** then hosp_ed_del = **0**; else hosp_ed_del = **1**;

if num_hosp_days_lf6wks = **0** then hosp_ed_lf6wks = **0**; else hosp_ed_lf6wks = **1**;

if hosp_ed_del = **1** or hosp_ed_lf6wks = **1** then hosp_ed_any = **1**; else hosp_ed_any = **0**;

if dthdate ^= **.** and (dthdate - b_bdate) <= **42** then dth_42d = **1**; else dth_42d = **0**;

**run**;

%***bivartab***(

data = redpph.preg_outcomes,

byvar = ancestry,

numdq = miss_only,

catvar = born_dad_pph pph_severe born_dad_pph_btrans born_dad_pph_hyst pph_bldctrl icu_delivery icu_6wkpostdel icu_any pro_icu_delivery

pro_icu_6wkpostdel pro_icu_any dth_42d dth_1yr hosp_adm_del hosp_adm_lf6wks hosp_adm_any prolonged_los_del prolonged_los_lf6wk

prolonged_los_any ed_visit_del ed_visit_lf6wks hosp_ed_del hosp_ed_lf6wks hosp_ed_any,

numvar = del_icu_hrs lf6wk_icu_hrs total_icu_hrs del_pro_icu_hrs lf6wk_pro_icu_hrs total_pro_icu_hrs num_hosp_days_del num_hosp_days_lf6wks

total_hosp_days num_ed_days_lf6wks num_hosp_ed_days_del num_hosp_ed_days_lf6wks total_hosp_ed_days,

order = born_dad_pph pph_severe born_dad_pph_btrans born_dad_pph_hyst pph_bldctrl icu_delivery del_icu_hrs icu_6wkpostdel lf6wk_icu_hrs icu_any

total_icu_hrs pro_icu_delivery del_pro_icu_hrs pro_icu_6wkpostdel lf6wk_pro_icu_hrs pro_icu_any total_pro_icu_hrs dth_42d dth_1yr hosp_adm_del

num_hosp_days_del hosp_adm_lf6wks num_hosp_days_lf6wks hosp_adm_any total_hosp_days prolonged_los_del prolonged_los_lf6wk prolonged_los_any

ed_visit_del ed_visit_lf6wks num_ed_days_lf6wks hosp_ed_del num_hosp_ed_days_del hosp_ed_lf6wks

num_hosp_ed_days_lf6wks hosp_ed_any total_hosp_ed_days,

total = T,

small = T,

stdiff = F,

trend = F,

binary = T,

out = outcomes_tab);

**data** outcomes_tab;

retain VarLabel Black Caucasian Asian other Unknown_missing _Total;

set outcomes_tab;

if VarValue in ('' 'Missing Data (%)') then delete;

keep VarLabel Black Caucasian Asian other Unknown_missing _Total;

**run**;

**proc** **sql**;

create table preg_cohort as

select a.parity, a.primary_language, a.immig_cat2, a.immig_birth_datediff_cat, b.*

from redpph.preg_cohort as a inner join redpph.preg_outcomes as b

on a.pregnancy_id_enc = b.pregnancy_id_enc;

**run**;

**data** preg_cohort;

set preg_cohort;

if immig_birth_datediff_cat = '' then immig_birth_datediff_cat = '(0) Not an immigrant';

**run**;

**data** nulli parous;

set preg_cohort;

if parity = **0** then output nulli;

else if parity ^= **.** then output parous;

**run**;

%***bivartab***(

data = nulli,

byvar = ancestry,

numdq = miss_only,

catvar = born_dad_pph pph_severe born_dad_pph_btrans born_dad_pph_hyst pph_bldctrl icu_delivery icu_6wkpostdel icu_any pro_icu_delivery

pro_icu_6wkpostdel pro_icu_any dth_42d dth_1yr hosp_adm_del hosp_adm_lf6wks hosp_adm_any prolonged_los_del prolonged_los_lf6wk

prolonged_los_any ed_visit_del ed_visit_lf6wks hosp_ed_del hosp_ed_lf6wks hosp_ed_any,

numvar = del_icu_hrs lf6wk_icu_hrs total_icu_hrs del_pro_icu_hrs lf6wk_pro_icu_hrs total_pro_icu_hrs num_hosp_days_del num_hosp_days_lf6wks

total_hosp_days num_ed_days_lf6wks num_hosp_ed_days_del num_hosp_ed_days_lf6wks total_hosp_ed_days,

order = born_dad_pph pph_severe born_dad_pph_btrans born_dad_pph_hyst pph_bldctrl icu_delivery del_icu_hrs icu_6wkpostdel lf6wk_icu_hrs icu_any

total_icu_hrs pro_icu_delivery del_pro_icu_hrs pro_icu_6wkpostdel lf6wk_pro_icu_hrs pro_icu_any total_pro_icu_hrs dth_42d dth_1yr hosp_adm_del

num_hosp_days_del hosp_adm_lf6wks num_hosp_days_lf6wks hosp_adm_any total_hosp_days prolonged_los_del prolonged_los_lf6wk prolonged_los_any

ed_visit_del ed_visit_lf6wks num_ed_days_lf6wks hosp_ed_del num_hosp_ed_days_del hosp_ed_lf6wks

num_hosp_ed_days_lf6wks hosp_ed_any total_hosp_ed_days,

total = T,

small = T,

stdiff = F,

trend = F,

binary = T,

out = outcomes_nulli);

**data** outcomes_nulli;

retain VarLabel Black Caucasian Asian other Unknown_missing _Total;

set outcomes_nulli;

if VarValue in ('' 'Missing Data (%)') then delete;

keep VarLabel Black Caucasian Asian other Unknown_missing _Total;

rename Black = Black_nulli Caucasian = Caucasian_nulli Asian = Asian_nulli other = other_nulli Unknown_missing = Unknown_nulli _Total = Total_nulli;

**run**;

%***bivartab***(

data = parous,

byvar = ancestry,

numdq = miss_only,

catvar = born_dad_pph pph_severe born_dad_pph_btrans born_dad_pph_hyst pph_bldctrl icu_delivery icu_6wkpostdel icu_any pro_icu_delivery

pro_icu_6wkpostdel pro_icu_any dth_42d dth_1yr hosp_adm_del hosp_adm_lf6wks hosp_adm_any prolonged_los_del prolonged_los_lf6wk

prolonged_los_any ed_visit_del ed_visit_lf6wks hosp_ed_del hosp_ed_lf6wks hosp_ed_any,

numvar = del_icu_hrs lf6wk_icu_hrs total_icu_hrs del_pro_icu_hrs lf6wk_pro_icu_hrs total_pro_icu_hrs num_hosp_days_del num_hosp_days_lf6wks

total_hosp_days num_ed_days_lf6wks num_hosp_ed_days_del num_hosp_ed_days_lf6wks total_hosp_ed_days,

order = born_dad_pph pph_severe born_dad_pph_btrans born_dad_pph_hyst pph_bldctrl icu_delivery del_icu_hrs icu_6wkpostdel lf6wk_icu_hrs icu_any

total_icu_hrs pro_icu_delivery del_pro_icu_hrs pro_icu_6wkpostdel lf6wk_pro_icu_hrs pro_icu_any total_pro_icu_hrs dth_42d dth_1yr hosp_adm_del

num_hosp_days_del hosp_adm_lf6wks num_hosp_days_lf6wks hosp_adm_any total_hosp_days prolonged_los_del prolonged_los_lf6wk prolonged_los_any

ed_visit_del ed_visit_lf6wks num_ed_days_lf6wks hosp_ed_del num_hosp_ed_days_del hosp_ed_lf6wks

num_hosp_ed_days_lf6wks hosp_ed_any total_hosp_ed_days,

total = T,

small = T,

stdiff = F,

trend = F,

binary = T,

out = outcomes_parous);

**data** outcomes_parous;

retain VarLabel Black Caucasian Asian other Unknown_missing _Total;

set outcomes_parous;

if VarValue in ('' 'Missing Data (%)') then delete;

keep VarLabel Black Caucasian Asian other Unknown_missing _Total;

rename Black = Black_parous Caucasian = Caucasian_parous Asian = Asian_parous other = other_parous Unknown_missing = Unknown_parous _Total = Total_parous;

**run**;

**data** outcomes_parity;

merge outcomes_nulli outcomes_parous;

**run**;

**proc** **sql**;

create table pph_ns as

select parous, ancestry, primary_language, count(*) as total, sum(born_dad_pph) as num_pph, sum(pph_severe) as num_sev_pph

from parity_cohort

group by parous, ancestry, primary_language;

create table pph_total as

select parous, ancestry, count(*) as total, sum(born_dad_pph) as num_pph, sum(pph_severe) as num_sev_pph

from parity_cohort

group by parous, ancestry;

**quit**;

**data** pph_ns;

set pph_ns pph_total;

if primary_language = '' then primary_language = 'All';

**run**;

**proc** **sort** data=pph_ns; by parous ancestry primary_language; **run**;

%***cirates***(

data = pph_ns,

num = num_pph,

denom = total,

out = pph_rates,

per = **100**,

method = poisson);

**data** pph_rates;

set pph_rates;

rate = catx(' (', round(CrudeRate, **0.01**), cats(round(lower, **0.01**), '-', round(upper, **0.01**), ')'));

keep parous ancestry primary_language rate;

**run**;

%***cirates***(

data = pph_ns,

num = num_sev_pph,

denom = total,

out = sev_pph_rates,

per = **100**,

method = poisson);

**data** sev_pph_rates;

set sev_pph_rates;

rate = catx(' (', round(CrudeRate, **0.01**), cats(round(lower, **0.01**), '-', round(upper, **0.01**), ')'));

keep parous ancestry primary_language rate;

**run**;

**%macro** pphsort(parous=, ancestry=, label=);

data pph;

length value $ **10.**;

set pph_rates;

if parous = &parous. and ancestry = "&ancestry";

value = "PPH";

drop parous ancestry;

rename rate = &label.&parous.;

run;

data severe_pph;

length value $ **10.**;

set sev_pph_rates;

if parous = &parous. and ancestry = "&ancestry";

value = "Severe PPH";

drop parous ancestry;

rename rate = &label.&parous.;

run;

data &label.&parous.; set pph severe_pph; run;

proc datasets; delete pph severe_pph; run;

**%mend**;

%***pphsort***(parous=**0**, ancestry=Asian, label=asian);

%***pphsort***(parous=**0**, ancestry=Black, label=black);

%***pphsort***(parous=**0**, ancestry=Caucasian, label=white);

%***pphsort***(parous=**0**, ancestry=Other, label=other);

%***pphsort***(parous=**0**, ancestry=Unknown/missing, label=unk);

%***pphsort***(parous=**1**, ancestry=Asian, label=asian);

%***pphsort***(parous=**1**, ancestry=Black, label=black);

%***pphsort***(parous=**1**, ancestry=Caucasian, label=white);

%***pphsort***(parous=**1**, ancestry=Other, label=other);

%***pphsort***(parous=**1**, ancestry=Unknown/missing, label=unk);

**data** rate_tab1; merge black0 white0 asian0 other0 unk0 black1 white1 asian1 other1 unk1; **run**;

**proc** **sort** data=rate_tab1; by value primary_language; **run**;

**proc** **sql**;

create table pph_ns as

select parous, ancestry, immig_cat2, count(*) as total, sum(born_dad_pph) as num_pph, sum(pph_severe) as num_sev_pph

from parity_cohort

group by parous, ancestry, immig_cat2;

create table pph_total as

select parous, ancestry, count(*) as total, sum(born_dad_pph) as num_pph, sum(pph_severe) as num_sev_pph

from parity_cohort

group by parous, ancestry;

**quit**;

**data** pph_ns;

set pph_ns pph_total;

if immig_cat2 = '' then immig_cat2 = '(0) All';

**run**;

**proc** **sort** data=pph_ns; by parous ancestry immig_cat2; **run**;

%***cirates***(

data = pph_ns,

num = num_pph,

denom = total,

out = pph_rates,

per = **100**,

method = poisson);

**data** pph_rates;

set pph_rates;

rate = catx(' (', round(CrudeRate, **0.01**), cats(round(lower, **0.01**), '-', round(upper, **0.01**), ')'));

keep parous ancestry immig_cat2 rate;

**run**;

%***cirates***(

data = pph_ns,

num = num_sev_pph,

denom = total,

out = sev_pph_rates,

per = **100**,

method = poisson);

**data** sev_pph_rates;

set sev_pph_rates;

rate = catx(' (', round(CrudeRate, **0.01**), cats(round(lower, **0.01**), '-', round(upper, **0.01**), ')'));

keep parous ancestry immig_cat2 rate;

**run**;

%***pphsort***(parous=**0**, ancestry=Asian, label=asian);

%***pphsort***(parous=**0**, ancestry=Black, label=black);

%***pphsort***(parous=**0**, ancestry=Caucasian, label=white);

%***pphsort***(parous=**0**, ancestry=Other, label=other);

%***pphsort***(parous=**0**, ancestry=Unknown/missing, label=unk);

%***pphsort***(parous=**1**, ancestry=Asian, label=asian);

%***pphsort***(parous=**1**, ancestry=Black, label=black);

%***pphsort***(parous=**1**, ancestry=Caucasian, label=white);

%***pphsort***(parous=**1**, ancestry=Other, label=other);

%***pphsort***(parous=**1**, ancestry=Unknown/missing, label=unk);

**data** rate_tab2; merge black0 white0 asian0 other0 unk0 black1 white1 asian1 other1 unk1; **run**;

**proc** **sort** data=rate_tab2; by value immig_cat2; **run**;

**proc** **sql**;

create table pph_ns as

select parous, ancestry, immig_birth_datediff_cat, count(*) as total, sum(born_dad_pph) as num_pph, sum(pph_severe) as num_sev_pph

from parity_cohort

group by parous, ancestry, immig_birth_datediff_cat;

create table pph_total as

select parous, ancestry, count(*) as total, sum(born_dad_pph) as num_pph, sum(pph_severe) as num_sev_pph

from parity_cohort

group by parous, ancestry;

**quit**;

**data** pph_ns;

set pph_ns pph_total;

if immig_birth_datediff_cat = '' then immig_birth_datediff_cat = '(0) All';

**run**;

**proc** **sort** data=pph_ns; by parous ancestry immig_birth_datediff_cat; **run**;

%***cirates***(

data = pph_ns,

num = num_pph,

denom = total,

out = pph_rates,

per = **100**,

method = poisson);

**data** pph_rates;

set pph_rates;

rate = catx(' (', round(CrudeRate, **0.01**), cats(round(lower, **0.01**), '-', round(upper, **0.01**), ')'));

keep parous ancestry immig_birth_datediff_cat rate;

**run**;

%***cirates***(

data = pph_ns,

num = num_sev_pph,

denom = total,

out = sev_pph_rates,

per = **100**,

method = poisson);

**data** sev_pph_rates;

set sev_pph_rates;

rate = catx(' (', round(CrudeRate, **0.01**), cats(round(lower, **0.01**), '-', round(upper, **0.01**), ')'));

keep parous ancestry immig_birth_datediff_cat rate;

**run**;

%***pphsort***(parous=**0**, ancestry=Asian, label=asian);

%***pphsort***(parous=**0**, ancestry=Black, label=black);

%***pphsort***(parous=**0**, ancestry=Caucasian, label=white);

%***pphsort***(parous=**0**, ancestry=Other, label=other);

%***pphsort***(parous=**0**, ancestry=Unknown/missing, label=unk);

%***pphsort***(parous=**1**, ancestry=Asian, label=asian);

%***pphsort***(parous=**1**, ancestry=Black, label=black);

%***pphsort***(parous=**1**, ancestry=Caucasian, label=white);

%***pphsort***(parous=**1**, ancestry=Other, label=other);

%***pphsort***(parous=**1**, ancestry=Unknown/missing, label=unk);

**data** rate_tab3; merge black0 white0 asian0 other0 unk0 black1 white1 asian1 other1 unk1; **run**;

**proc** **sort** data=rate_tab3; by value immig_birth_datediff_cat; **run**;

**data** rate_tab1; set rate_tab1; rename primary_language = category; **run**;

**data** rate_tab2; set rate_tab2; rename immig_cat2 = category; **run**;

**data** rate_tab3; set rate_tab3; rename immig_birth_datediff_cat = category; **run**;

**data** rate_tab; set rate_tab1-rate_tab3; **run**;

/* Second stage duration variable missingness check: */

**data** durstage2;

set redpph.preg_cohort;

if **'01APR2013'd** <= b_bdate < **'01APR2014'd** then fyear = **2014**;

else if **'01APR2014'd** <= b_bdate < **'01APR2015'd** then fyear = **2015**;

else if **'01APR2015'd** <= b_bdate < **'01APR2016'd** then fyear = **2016**;

else if **'01APR2016'd** <= b_bdate < **'01APR2017'd** then fyear = **2017**;

else if **'01APR2017'd** <= b_bdate < **'01APR2018'd** then fyear = **2018**;

else if **'01APR2018'd** <= b_bdate < **'01APR2019'd** then fyear = **2019**;

else if **'01APR2019'd** <= b_bdate < **'01APR2020'd** then fyear = **2020**;

else if **'01APR2020'd** <= b_bdate < **'01APR2021'd** then fyear = **2021**;

if duration_stage2 = 'Missing' then missing_durstage2 = **1**;

else missing_durstage2 = **0**;

keep ikn duration_stage2 b_bdate fyear missing_durstage2 mode_of_delivery;

**run**;

**proc** **sql**;

create table total_missing as

select mode_of_delivery, fyear, count(*) as total

from durstage2

group by mode_of_delivery, fyear;

create table missing as

select mode_of_delivery, fyear, missing_durstage2, count(*) as missing

from durstage2

group by mode_of_delivery, fyear, missing_durstage2;

**quit**;

**data** missing; set missing; if missing_durstage2 = **1**; drop missing_durstage2; **run**;

**data** missing_tab;

merge missing total_missing;

by mode_of_delivery fyear;

if missing = **.** then missing = **0**;

percent = missing/total;

**run**;

%***bivartab***(

data = durstage2,

byvar = fyear,

catvar = missing_durstage2,

total = T,

small = T,

stdiff = F,

trend = F,

binary = T,

out = table1);

**data** table1;

set table1;

if fyear__Total = '' then delete;

keep Variable fyear:;

**run**;

**proc** **transpose** data=table1 out=table1_tr;

id Variable;

var fyear_2014 fyear_2015 fyear_2016 fyear_2017 fyear_2018 fyear_2019 fyear_2020 fyear_2021 fyear__Total;

**run**;

/* Temporal trend in PPH and severe PPH: */

**data** preg_cohort;

set redpph.preg_outcomes;

if **'01APR2013'd** <= b_bdate < **'01APR2014'd** then fyear = **2014**;

else if **'01APR2014'd** <= b_bdate < **'01APR2015'd** then fyear = **2015**;

else if **'01APR2015'd** <= b_bdate < **'01APR2016'd** then fyear = **2016**;

else if **'01APR2016'd** <= b_bdate < **'01APR2017'd** then fyear = **2017**;

else if **'01APR2017'd** <= b_bdate < **'01APR2018'd** then fyear = **2018**;

else if **'01APR2018'd** <= b_bdate < **'01APR2019'd** then fyear = **2019**;

else if **'01APR2019'd** <= b_bdate < **'01APR2020'd** then fyear = **2020**;

else if **'01APR2020'd** <= b_bdate < **'01APR2021'd** then fyear = **2021**;

keep pregnancy_id_enc b_bdate fyear ancestry ikn born_dad_pph pph_severe;

**run**;

**proc** **sql**;

create table preg_cohort as

select a.*, b.parity_cat, b.mode_of_delivery

from preg_cohort as a inner join redpph.preg_cohort as b

on a.pregnancy_id_enc = b.pregnancy_id_enc;

**quit**;

**data** preg_cohort;

set preg_cohort;

if parity_cat = '0' then parous = **0**; else parous = **1**;

**run**;

**proc** **sort** data=preg_cohort; by ancestry mode_of_delivery; **run**;

ods trace on;

**proc** **freq** data=preg_cohort;

by ancestry mode_of_delivery;

tables born_dad_pph*fyear / nofreq nopercent norow trend;

**run**;

ods trace off;

**%macro** trends(var=);

proc sort data=preg_cohort; by ancestry parous; run;

proc freq data=preg_cohort;

by ancestry;

tables &var.*fyear / nofreq nopercent norow trend;

%do i = **1** %to **5**;

ods output Freq.ByGroup&i.**.T**able1.TrendTest = trend&i.;

%end;

run;

%do j = **1** %to **5**;

data trend&j.; set trend&j.; if _n_ = **3**; keep ancestry nValue1; rename nValue1 = trend_pval; run;

%end;

data full_trend; set trend1-trend5; run;

proc datasets; delete trend1-trend5; run;

proc freq data=preg_cohort;

by ancestry parous;

tables &var.*fyear / nofreq nopercent norow trend;

%do k = **1** %to **10**;

ods output Freq.ByGroup&k.**.T**able1.TrendTest = trend&k.;

%end;

run;

%do l = **1** %to **10**;

data trend&l.; set trend&l.; if _n_ = **3**; keep ancestry parous nValue1; rename nValue1 = trend_pval; run;

%end;

data parity_trend; set trend1-trend10; run;

proc datasets; delete trend1-trend10; run;

data parity_trend; retain parous ancestry; set full_trend parity_trend; run;

proc sort data=parity_trend; by parous ancestry; run;

proc sort data=preg_cohort; by ancestry mode_of_delivery; run;

proc freq data=preg_cohort;

by ancestry mode_of_delivery;

tables &var.*fyear / nofreq nopercent norow trend;

%do m = **1** %to **50**;

ods output Freq.ByGroup&m.**.T**able1.TrendTest = trend&m.;

%end;

run;

%do n = **1** %to **50**;

data trend&n.; set trend&n.; if _n_ = **3**; keep ancestry mode_of_delivery nValue1; rename nValue1 = trend_pval; run;

%end;

data mode_trend; set trend1-trend14 trend16-trend19 trend21-trend34 trend36-trend50; run;

proc datasets; delete trend1-trend50; run;

data mode_trend; retain mode_of_delivery ancestry; set full_trend mode_trend; run;

proc sort data=mode_trend; by mode_of_delivery ancestry; run;

proc datasets; delete full_trend; run;

**%mend**;

%***trends***(var=born_dad_pph);

**data** pph_parity_trend; length outcome $ **10.**; set parity_trend; outcome = 'PPH'; **run**;

**data** pph_mode_trend; length outcome $ **10.**; set mode_trend; outcome = 'PPH'; **run**;

%***trends***(var=pph_severe);

**data** spph_parity_trend; length outcome $ **10.**; set parity_trend; outcome = 'Severe PPH'; **run**;

**data** spph_mode_trend; length outcome $ **10.**; set mode_trend; outcome = 'Severe PPH'; **run**;

**data** parity_trend;

retain parous outcome ancestry;

set pph_parity_trend spph_parity_trend;

**run**;

**proc** **sort** data=parity_trend; by parous outcome ancestry; **run**;

**data** mode_trend;

retain mode_of_delivery outcome ancestry;

set pph_mode_trend spph_mode_trend;

**run**;

**proc** **sort** data=mode_trend; by mode_of_delivery outcome ancestry; **run**;

**proc** **sql**;

create table totals as

select ancestry, count(*) as total

from preg_cohort

group by ancestry;

create table totals_parity as

select ancestry, parous, count(*) as total

from preg_cohort

group by ancestry, parous;

create table pph_rates as

select ancestry, fyear, sum(born_dad_pph)***1000**/(count(*)) as rate

from preg_cohort

group by ancestry, fyear;

create table pph_rates_parity as

select ancestry, parous, fyear, sum(born_dad_pph)***1000**/(count(*)) as rate

from preg_cohort

group by ancestry, parous, fyear;

create table severe_pph_rates as

select ancestry, fyear, sum(pph_severe)***1000**/(count(*)) as rate

from preg_cohort

group by ancestry, fyear;

create table severe_pph_rates_parity as

select ancestry, parous, fyear, sum(pph_severe)***1000**/(count(*)) as rate

from preg_cohort

group by ancestry, parous, fyear;

**quit**;

**data** redpph.pph_rates_parity;

retain severe parous ancestry;

set pph_rates(in=a) pph_rates_parity(in=b) severe_pph_rates(in=c) severe_pph_rates_parity(in=d);

if a or b then severe = **0**;

else severe = **1**;

if parous = **.** then parnum = '1';

else if parous = **0** then parnum = '2';

else parnum = '3';

**run**;

**proc** **sort** data=redpph.pph_rates_parity; by severe parous ancestry fyear; **run**;

**data** totals; set totals totals_parity; **run**;

**proc** **sort** data=totals; by parous ancestry; **run**;

**data** pph;

length outcome $ **10.**;

set pph_rates pph_rates_parity;

outcome = 'PPH';

**run**;

**data** severe_pph;

length outcome $ **10.**;

set severe_pph_rates severe_pph_rates_parity;

outcome = 'Severe PPH';

**run**;

**proc** **sort** data=pph; by parous ancestry; **run**;

**proc** **sort** data=severe_pph; by parous ancestry; **run**;

**data** pph;

merge totals pph;

by parous ancestry;

**run**;

**data** severe_pph;

merge totals severe_pph;

by parous ancestry;

**run**;

**data** pph;

retain outcome parous ancestry fyear total rate;

length outcome $ **10.**;

set pph severe_pph;

**run**;

**proc** **sort** data=pph; by outcome parous ancestry; **run**;

**data** rate2014(rename=(rate=rate2014)) rate2015(drop=total rename=(rate=rate2015)) rate2016(drop=total rename=(rate=rate2016))

rate2017(drop=total rename=(rate=rate2017)) rate2018(drop=total rename=(rate=rate2018)) rate2019(drop=total rename=(rate=rate2019))

rate2020(drop=total rename=(rate=rate2020)) rate2021(drop=total rename=(rate=rate2021));

set pph;

if fyear = **2014** then output rate2014;

else if fyear = **2015** then output rate2015;

else if fyear = **2016** then output rate2016;

else if fyear = **2017** then output rate2017;

else if fyear = **2018** then output rate2018;

else if fyear = **2019** then output rate2019;

else if fyear = **2020** then output rate2020;

else output rate2021;

drop fyear;

**run**;

**data** pph_parity;

retain parous outcome ancestry total;

merge rate2014-rate2021;

by outcome parous ancestry;

**run**;

**proc** **sort** data=pph_parity; by parous outcome ancestry; **run**;

**data** tables.table5_parity;

merge pph_parity parity_trend;

by parous outcome ancestry;

**run**;

**proc** **sql**;

create table totals as

select ancestry, count(*) as total

from preg_cohort

group by ancestry;

create table totals_mode as

select ancestry, mode_of_delivery, count(*) as total

from preg_cohort

group by ancestry, mode_of_delivery;

create table pph_rates as

select ancestry, fyear, sum(born_dad_pph)***1000**/(count(*)) as rate

from preg_cohort

group by ancestry, fyear;

create table pph_rates_mode as

select ancestry, mode_of_delivery, fyear, sum(born_dad_pph)***1000**/(count(*)) as rate

from preg_cohort

group by ancestry, mode_of_delivery, fyear;

create table severe_pph_rates as

select ancestry, fyear, sum(pph_severe)***1000**/(count(*)) as rate

from preg_cohort

group by ancestry, fyear;

create table severe_pph_rates_mode as

select ancestry, mode_of_delivery, fyear, sum(pph_severe)***1000**/(count(*)) as rate

from preg_cohort

group by ancestry, mode_of_delivery, fyear;

**quit**;

**data** redpph.pph_rates_mode;

retain severe delivery_mode ancestry;

set pph_rates(in=a) pph_rates_mode(in=b) severe_pph_rates(in=c) severe_pph_rates_mode(in=d);

if a or b then severe = **0**;

else severe = **1**;

if mode_of_delivery in ('(05) OVD none/unknown' '(09) Unknown stage CD' '(10) Perimortem CD') then delete;

delivery_mode = substr(mode_of_delivery, **6**, **27**);

if delivery_mode = '' then delivery_mode = 'Full';

if delivery_mode = 'Full' then modenum = '1';

else if delivery_mode = 'SVD' then modenum = '2';

else if delivery_mode = 'Forceps delivery' then modenum = '3';

else if delivery_mode = 'Vacuum delivery' then modenum = '4';

else if delivery_mode = 'Forceps and vacuum delivery' then modenum = '5';

else if delivery_mode = 'First stage CD' then modenum = '6';

else if delivery_mode = 'Second stage CD' then modenum = '7';

else modenum = '8';

drop mode_of_delivery;

**run**;

**proc** **sort** data=redpph.pph_rates_mode; by severe delivery_mode ancestry fyear; **run**;

**data** totals;

set totals totals_mode;

if mode_of_delivery in ('(05) OVD none/unknown' '(09) Unknown stage CD' '(10) Perimortem CD') then delete;

if mode_of_delivery = '' then mode_of_delivery = '(00) Full';

**run**;

**proc** **sort** data=totals; by mode_of_delivery ancestry; **run**;

**data** pph;

length outcome $ **10.**;

set pph_rates pph_rates_mode;

outcome = 'PPH';

if mode_of_delivery in ('(05) OVD none/unknown' '(09) Unknown stage CD' '(10) Perimortem CD') then delete;

if mode_of_delivery = '' then mode_of_delivery = '(00) Full';

**run**;

**data** severe_pph;

length outcome $ **10.**;

set severe_pph_rates severe_pph_rates_mode;

outcome = 'Severe PPH';

if mode_of_delivery in ('(05) OVD none/unknown' '(09) Unknown stage CD' '(10) Perimortem CD') then delete;

if mode_of_delivery = '' then mode_of_delivery = '(00) Full';

**run**;

**proc** **sort** data=pph; by mode_of_delivery ancestry; **run**;

**proc** **sort** data=severe_pph; by mode_of_delivery ancestry; **run**;

**data** pph;

merge totals pph;

by mode_of_delivery ancestry;

**run**;

**data** severe_pph;

merge totals severe_pph;

by mode_of_delivery ancestry;

**run**;

**data** pph;

retain outcome mode_of_delivery ancestry fyear total rate;

set pph severe_pph;

**run**;

**proc** **sort** data=pph; by outcome mode_of_delivery ancestry; **run**;

**data** rate2014(rename=(rate=rate2014)) rate2015(drop=total rename=(rate=rate2015)) rate2016(drop=total rename=(rate=rate2016))

rate2017(drop=total rename=(rate=rate2017)) rate2018(drop=total rename=(rate=rate2018)) rate2019(drop=total rename=(rate=rate2019))

rate2020(drop=total rename=(rate=rate2020)) rate2021(drop=total rename=(rate=rate2021));

set pph;

if fyear = **2014** then output rate2014;

else if fyear = **2015** then output rate2015;

else if fyear = **2016** then output rate2016;

else if fyear = **2017** then output rate2017;

else if fyear = **2018** then output rate2018;

else if fyear = **2019** then output rate2019;

else if fyear = **2020** then output rate2020;

else output rate2021;

drop fyear;

**run**;

**data** pph_mode;

retain mode_of_delivery outcome ancestry total;

merge rate2014-rate2021;

by outcome mode_of_delivery ancestry;

**run**;

**proc** **sort** data=pph_mode; by mode_of_delivery outcome ancestry; **run**;

**data** mode_trend;

set mode_trend;

if mode_of_delivery in ('(05) OVD none/unknown' '(09) Unknown stage CD' '(10) Perimortem CD') then delete;

if mode_of_delivery = '' then mode_of_delivery = '(00) Full';

**run**;

**data** tables.table5_mode;

merge pph_mode mode_trend;

by mode_of_delivery outcome ancestry;

**run**;

/* Explore maternal race/ethnicity by year: */

**data** ethnic_year;

set redpph.preg_cohort;

if **'01APR2013'd** <= b_bdate < **'01APR2014'd** then fyear = **2014**;

else if **'01APR2014'd** <= b_bdate < **'01APR2015'd** then fyear = **2015**;

else if **'01APR2015'd** <= b_bdate < **'01APR2016'd** then fyear = **2016**;

else if **'01APR2016'd** <= b_bdate < **'01APR2017'd** then fyear = **2017**;

else if **'01APR2017'd** <= b_bdate < **'01APR2018'd** then fyear = **2018**;

else if **'01APR2018'd** <= b_bdate < **'01APR2019'd** then fyear = **2019**;

else if **'01APR2019'd** <= b_bdate < **'01APR2020'd** then fyear = **2020**;

else if **'01APR2020'd** <= b_bdate < **'01APR2021'd** then fyear = **2021**;

if ancestry = 'Unknown/missing' then ancestry_missing = **1**; else ancestry_missing = **0**;

keep pregnancy_id_enc b_bdate ancestry ikn fyear ancestry_missing;

**run**;

**proc** **freq** data=ethnic_year;

tables fyear / out=year_totals;

tables ancestry_missing*fyear / out=ethnic_missing_year;

**run**;

**data** year_totals; set year_totals; drop percent; rename count = total; **run**;

**data** ethnic_missing_year; set ethnic_missing_year; if ancestry_missing = **1**; drop ancestry_missing percent; rename count=num_missing; **run**;

**data** ethnic_missing_year;

merge ethnic_missing_year year_totals;

by fyear;

percent_missing = num_missing***100**/total;

**run**;

/* Get data for models: */

**data** modeldata;

set redpph.preg_outcomes;

if ancestry = 'Unknown/missing' then delete;

keep pregnancy_id_enc ancestry ikn born_dad_pph pph_severe;

**run**;

**data** modelvars;

set redpph.preg_cohort;

if parity_cat = '0' then parity_bin = **0**;

else if parity_cat = 'Unknown' then parity_bin = **9**;

else parity_bin = **1**;

*if primary_language in ('English' 'French') then primary_language = 'English/French';

if smoking_atbirth = **1** or smoking_firstvisit = **1** then preg_smoking = **1**;

else if smoking_atbirth = **9** and smoking_firstvisit = **9** then preg_smoking = **9**;

else preg_smoking = **0**;

if preg_hyper not in ('N/A' 'None') then gest_hyper = **1**;

else gest_hyper = **0**;

if immig_birth_datediff_cat = '' then immig_birth_datediff_cat = '(6) Not an immigrant';

if duration_stage2 = 'Missing' then duration_stage2 = 'No second stage';

if substr(mode_of_delivery, **1**, **3**) in ('(09' '(10') then mode_of_delivery = '(09) US/PM CD';

keep pregnancy_id_enc age_group parity_bin plurality primary_language maternal_bmi_cat age_labourforce_q_da material_resources_q_da

households_dwellings_q_da immig_cat2 immig_birth_datediff_cat rural_pccf preg_smoking preg_drug_exposure conception_type

tri1_prenatal_visit preex_dm preex_hyper previous_cd gest_dm gest_hyper placenta_previa placenta_accspec placental_abruption

duration_stage2 mode_of_delivery gestage_cat induction augmentation_ind presentation_type episiotomy b_weight_g_cat1 b_headcirc_cm_cat1;

**run**;

**proc** **sql**;

create table modeldata as

select * from modeldata as a left join modelvars as b

on a.pregnancy_id_enc = b.pregnancy_id_enc;

**quit**;

**%macro** univariate(data=, var=, ref=, num=);

proc genmod data=&data.;

class ikn &var.(ref="&ref") / param=ref;

model born_dad_pph = &var. / dist=poisson link=log;

repeated subject=ikn;

ods output Genmod.GEEEmpPEst = &var._uni_rr;

run;

data &var._uni_rr;

retain Variable Level1 est lower upper probz place1;

length Variable $ **24.**;

set &var._uni_rr;

Variable = "&var";

if Parm = 'Intercept' then delete;

est = round(exp(Estimate), **0.0001**);

lower = round(exp(LowerCL), **0.0001**);

upper = round(exp(UpperCL), **0.0001**);

place1 = &num.;

keep Variable Level1 est lower upper probz place1;

rename probz = pval;

run;

data &var._uni_rr;

set &var._uni_rr;

place2 = _n_;

run;

**%mend**;

%***univariate***(data=modeldata, var=ancestry, ref=Caucasian, num=**1**);

%***univariate***(data=modeldata, var=age_group, ref=**20**-**24**, num=**2**);

**data** age_group_uni_rr;

set age_group_uni_rr;

if Level1 = '<20' then place2 = **1**;

else place2 = _n_ + **1**;

**run**;

%***univariate***(data=modeldata, var=parity_bin, ref=**1**, num=**3**);

%***univariate***(data=modeldata, var=plurality, ref=**0**, num=**4**);

%***univariate***(data=modeldata, var=primary_language, ref=English, num=**5**);

%***univariate***(data=modeldata, var=maternal_bmi_cat, ref=**18.5**-**24.9**, num=**6**);

**data** maternal_bmi_cat_uni_rr;

set maternal_bmi_cat_uni_rr;

if Level1 = '<18.5' then place2 = **1**;

else if Level1 not in ('>=40' 'Unknown') then place2 = _n_ + **1**;

else place2 = _n_;

**run**;

%***univariate***(data=modeldata, var=age_labourforce_q_da, ref=**1**, num=**7**);

%***univariate***(data=modeldata, var=material_resources_q_da, ref=**1**, num=**8**);

%***univariate***(data=modeldata, var=households_dwellings_q_da, ref=**1**, num=**9**);

%***univariate***(data=modeldata, var=immig_cat2, ref=(**1**) Not an immigrant, num=**10**);

%***univariate***(data=modeldata, var=immig_birth_datediff_cat, ref=(**5**) >**10** years, num=**11**);

%***univariate***(data=modeldata, var=rural_pccf, ref=N, num=**12**);

%***univariate***(data=modeldata, var=preg_smoking, ref=**0**, num=**13**);

%***univariate***(data=modeldata, var=preg_drug_exposure, ref=**0**, num=**14**);

%***univariate***(data=modeldata, var=conception_type, ref=Spontaneous, num=**15**);

%***univariate***(data=modeldata, var=tri1_prenatal_visit, ref=**1**, num=**16**);

%***univariate***(data=modeldata, var=preex_dm, ref=**0**, num=**17**);

%***univariate***(data=modeldata, var=gest_dm, ref=**0**, num=**18**);

%***univariate***(data=modeldata, var=preex_hyper, ref=**0**, num=**19**);

%***univariate***(data=modeldata, var=gest_hyper, ref=**0**, num=**20**);

%***univariate***(data=modeldata, var=previous_cd, ref=**0**, num=**21**);

%***univariate***(data=modeldata, var=placenta_previa, ref=**0**, num=**22**);

%***univariate***(data=modeldata, var=placenta_accspec, ref=**0**, num=**23**);

%***univariate***(data=modeldata, var=placental_abruption, ref=**0**, num=**24**);

%***univariate***(data=modeldata, var=induction, ref=**0**, num=**25**);

%***univariate***(data=modeldata, var=augmentation_ind, ref=**0**, num=**26**);

%***univariate***(data=modeldata, var=episiotomy, ref=None, num=**27**);

%***univariate***(data=modeldata, var=presentation_type, ref=Cephalic, num=**28**);

%***univariate***(data=modeldata, var=duration_stage2, ref=<**60** minutes, num=**29**);

**data** duration_stage2_uni_rr;

set duration_stage2_uni_rr;

if Level1 = '60-119 minutes' then place2 = **1**;

else if Level1 in ('120-179 minutes' '180-239 minutes') then place2 = _n_ + **1**;

else place2 = _n_;

**run**;

%***univariate***(data=modeldata, var=mode_of_delivery, ref=(**01**) SVD, num=**30**);

%***univariate***(data=modeldata, var=gestage_cat, ref=(**2**) Term, num=**31**);

%***univariate***(data=modeldata, var=b_weight_g_cat1, ref=(**2**) **3000**-**3999**, num=**32**);

%***univariate***(data=modeldata, var=b_headcirc_cm_cat1, ref=(**2**) **33**-**34**, num=**33**);

**data** uni_rr;

retain Variable Level1;

length Level1 $ **30.**;

set ancestry_uni_rr age_group_uni_rr parity_bin_uni_rr plurality_uni_rr primary_language_uni_rr maternal_bmi_cat_uni_rr age_labourforce_q_da_uni_rr

material_resources_q_da_uni_rr households_dwellings_q_da_uni_rr immig_cat2_uni_rr immig_birth_datediff_cat_uni_rr rural_pccf_uni_rr preg_smoking_uni_rr

preg_drug_exposure_uni_rr conception_type_uni_rr tri1_prenatal_visit_uni_rr preex_dm_uni_rr gest_dm_uni_rr preex_hyper_uni_rr gest_hyper_uni_rr

previous_cd_uni_rr placenta_previa_uni_rr placenta_accspec_uni_rr placental_abruption_uni_rr induction_uni_rr augmentation_ind_uni_rr episiotomy_uni_rr

presentation_type_uni_rr duration_stage2_uni_rr mode_of_delivery_uni_rr gestage_cat_uni_rr b_weight_g_cat1_uni_rr b_headcirc_cm_cat1_uni_rr;

rename est=uni_est lower=uni_lower upper=uni_upper pval=uni_pval;

**run**;

**proc** **sort** data=uni_rr out=uni_rr(drop=place:); by place1 place2; **run**;

**data** uni_rr_reduced;

retain Variable Level1;

length Level1 $ **30.**;

set ancestry_uni_rr age_group_uni_rr plurality_uni_rr primary_language_uni_rr maternal_bmi_cat_uni_rr material_resources_q_da_uni_rr immig_cat2_uni_rr

rural_pccf_uni_rr preg_smoking_uni_rr preg_drug_exposure_uni_rr conception_type_uni_rr tri1_prenatal_visit_uni_rr preex_dm_uni_rr gest_dm_uni_rr

preex_hyper_uni_rr gest_hyper_uni_rr previous_cd_uni_rr induction_uni_rr augmentation_ind_uni_rr presentation_type_uni_rr duration_stage2_uni_rr

mode_of_delivery_uni_rr b_weight_g_cat1_uni_rr;

rename est=uni_est lower=uni_lower upper=uni_upper pval=uni_pval;

**run**;

**proc** **sort** data=uni_rr_reduced out=uni_rr_reduced(drop=place:); by place1 place2; **run**;

**proc** **genmod** data=modeldata;

class ikn ancestry(ref="Caucasian") age_group(ref="20-24") parity_bin(ref="1") plurality(ref="0") primary_language(ref="English/French")

maternal_bmi_cat(ref="18.5-24.9") age_labourforce_q_da(ref="1") material_resources_q_da(ref="1") households_dwellings_q_da(ref="1")

immig_cat2(ref="(1) Not an immigrant") immig_birth_datediff_cat(ref="(5) >10 years") rural_pccf(ref="N") preg_smoking(ref="0")

preg_drug_exposure(ref="0") conception_type(ref="Spontaneous") tri1_prenatal_visit(ref="1") preex_dm(ref="0") gest_dm(ref="0")

preex_hyper(ref="0") gest_hyper(ref="0") previous_cd(ref="0") placenta_previa(ref="0") placenta_accspec(ref="0")

placental_abruption(ref="0") induction(ref="0") augmentation_ind(ref="0") episiotomy(ref="None") presentation_type(ref="Cephalic")

duration_stage2(ref="<60 minutes") mode_of_delivery(ref="(01) SVD") gestage_cat(ref="(2) Term") b_weight_g_cat1(ref="(2) 3000-3999")

b_headcirc_cm_cat1(ref="(2) 33-34");

model born_dad_pph = ancestry age_group parity_bin plurality primary_language maternal_bmi_cat age_labourforce_q_da material_resources_q_da

households_dwellings_q_da immig_cat2 immig_birth_datediff_cat rural_pccf preg_smoking preg_drug_exposure conception_type

tri1_prenatal_visit preex_dm gest_dm preex_hyper gest_hyper previous_cd placenta_previa placenta_accspec placental_abruption induction

augmentation_ind episiotomy presentation_type duration_stage2 mode_of_delivery gestage_cat b_weight_g_cat1 b_headcirc_cm_cat1

/ dist=poisson link=log;

repeated subject=ikn;

ods output Genmod.GEEEmpPEst = multi_rr;

**run**;

**data** multi_rr;

retain Parm Level1 adj_est adj_lower adj_upper ProbZ;

set multi_rr;

if _n_ = **1** or _n_ = **5** or _n_ = **11** or _n_ = **14** or _n_ = **16** or _n_ = **19** or _n_ = **26** or _n_ = **32** or _n_ = **38** or _n_ = **44** or _n_ = **49** or _n_ = **55**

or _n_ = **58** or _n_ = **61** or _n_ = **64** or _n_ = **67** or _n_ = **70** or _n_ = **72** or _n_ = **74** or _n_ = **76** or _n_ = **78** or _n_ = **80** or _n_ = **82** or _n_ = **84**

or _n_ = **86** or _n_ = **88** or _n_ = **90** or _n_ = **94** or _n_ = **98** or _n_ = **104** or _n_ = **113** or _n_ = **116** or _n_ = **121** or _n_ = **126** then delete;

adj_est = round(exp(Estimate), **0.0001**);

adj_lower = round(exp(LowerCL), **0.0001**);

adj_upper = round(exp(UpperCL), **0.0001**);

keep Parm Level1 adj_est adj_lower adj_upper ProbZ;

rename ProbZ = adj_pval;

**run**;

**data** multi_rr;

set multi_rr;

if Parm = 'age_group' then do;

if Level1 = '<20' then place = _n_ - **4**;

else place = _n_ + **1**;

end;

else if Parm = 'maternal_bmi_cat' then do;

if Level1 = '<18.5' then place = _n_ - **3**;

else if Level1 in ('>=40' 'Unknown') then place = _n_;

else place = _n_ + **1**;

end;

else if Parm = 'duration_stage2' then do;

if Level1 = '60-119 minutes' then place = _n_ - **2**;

else if Level1 in ('120-179 minutes' '180-239 minutes') then place = _n_ + **1**;

else place = _n_;

end;

else place = _n_;

**run**;

**proc** **sort** data=multi_rr out=multi_rr(drop=place); by place; **run**;

**data** tables.model_results;

merge uni_rr multi_rr;

drop Parm;

**run**;

**proc** **genmod** data=modeldata;

class ikn ancestry(ref="Caucasian") age_group(ref="20-24") plurality(ref="0") primary_language(ref="English/French") maternal_bmi_cat(ref="18.5-24.9")

material_resources_q_da(ref="1") immig_cat2(ref="(1) Not an immigrant") rural_pccf(ref="N") preg_smoking(ref="0") preg_drug_exposure(ref="0")

conception_type(ref="Spontaneous") tri1_prenatal_visit(ref="1") preex_dm(ref="0") gest_dm(ref="0") preex_hyper(ref="0") gest_hyper(ref="0")

previous_cd(ref="0") induction(ref="0") augmentation_ind(ref="0") presentation_type(ref="Cephalic") duration_stage2(ref="<60 minutes")

mode_of_delivery(ref="(01) SVD") b_weight_g_cat1(ref="(2) 3000-3999");

model born_dad_pph = ancestry age_group plurality primary_language maternal_bmi_cat material_resources_q_da immig_cat2 rural_pccf preg_smoking preg_drug_exposure

conception_type tri1_prenatal_visit preex_dm gest_dm preex_hyper gest_hyper previous_cd induction augmentation_ind presentation_type duration_stage2

mode_of_delivery b_weight_g_cat1 / dist=poisson link=log;

repeated subject=ikn;

ods output Genmod.GEEEmpPEst = multi_rr_reduced;

**run**;

**data** multi_rr_reduced;

retain Parm Level1 adj_est adj_lower adj_upper ProbZ;

set multi_rr_reduced;

adj_est = round(exp(Estimate), **0.0001**);

adj_lower = round(exp(LowerCL), **0.0001**);

adj_upper = round(exp(UpperCL), **0.0001**);

keep Parm Level1 adj_est adj_lower adj_upper ProbZ;

rename ProbZ = adj_pval;

**run**;

**data** multi_rr_reduced;

set multi_rr_reduced;

if Parm = 'age_group' then do;

if Level1 = '<20' then place = _n_ - **4**;

else place = _n_ + **1**;

end;

else if Parm = 'maternal_bmi_cat' then do;

if Level1 = '<18.5' then place = _n_ - **3**;

else if Level1 in ('>=40' 'Unknown') then place = _n_;

else place = _n_ + **1**;

end;

else if Parm = 'duration_stage2' then do;

if Level1 = '60-119 minutes' then place = _n_ - **2**;

else if Level1 in ('120-179 minutes' '180-239 minutes') then place = _n_ + **1**;

else place = _n_;

end;

else place = _n_;

**run**;

**proc** **sort** data=multi_rr_reduced out=multi_rr_reduced(drop=place); by place; **run**;

**data** tables.model_results_reduced;

merge uni_rr_reduced multi_rr_reduced;

**run**;

**data** tables.model_results_reduced;

set tables.model_results_reduced;

drop place: Parm;

**run**;

/* Stratify by parity: */

**data** nulli parous;

set modeldata;

if parity_bin = **1** then output parous;

else if parity_bin = **0** then output nulli;

**run**;

%***univariate***(data=nulli, var=ancestry, ref=Caucasian, num=**1**);

**data** ancestry_uni_rr;

retain Variable Level1 est lower upper probz place1;

length Variable $ **24.**;

set ancestry_uni_rr;

Variable = "ancestry";

if Parm = 'Intercept' then delete;

est = round(exp(Estimate), **0.0001**);

lower = round(exp(LowerCL), **0.0001**);

upper = round(exp(UpperCL), **0.0001**);

place1 = &num.;

keep Variable Level1 est lower upper probz place1;

rename probz = pval;

**run**;

**data** &var._uni_rr;

set &var._uni_rr;

place2 = _n_;

**run**;

%***univariate***(data=nulli, var=age_group, ref=**20**-**24**, num=**2**);

**data** age_group_uni_rr;

set age_group_uni_rr;

if Level1 = '<20' then place2 = **1**;

else place2 = _n_ + **1**;

**run**;

%***univariate***(data=nulli, var=plurality, ref=**0**, num=**3**);

%***univariate***(data=nulli, var=primary_language, ref=English, num=**4**);

%***univariate***(data=nulli, var=maternal_bmi_cat, ref=**18.5**-**24.9**, num=**5**);

**data** maternal_bmi_cat_uni_rr;

set maternal_bmi_cat_uni_rr;

if Level1 = '<18.5' then place2 = **1**;

else if Level1 not in ('>=40' 'Unknown') then place2 = _n_ + **1**;

else place2 = _n_;

**run**;

%***univariate***(data=nulli, var=age_labourforce_q_da, ref=**1**, num=**6**);

%***univariate***(data=nulli, var=material_resources_q_da, ref=**1**, num=**7**);

%***univariate***(data=nulli, var=households_dwellings_q_da, ref=**1**, num=**8**);

%***univariate***(data=nulli, var=immig_cat2, ref=(**1**) Not an immigrant, num=**9**);

%***univariate***(data=nulli, var=immig_birth_datediff_cat, ref=(**5**) >**10** years, num=**10**);

%***univariate***(data=nulli, var=rural_pccf, ref=N, num=**11**);

%***univariate***(data=nulli, var=preg_smoking, ref=**0**, num=**12**);

%***univariate***(data=nulli, var=preg_drug_exposure, ref=**0**, num=**13**);

%***univariate***(data=nulli, var=conception_type, ref=Spontaneous, num=**14**);

%***univariate***(data=nulli, var=tri1_prenatal_visit, ref=**1**, num=**15**);

%***univariate***(data=nulli, var=preex_dm, ref=**0**, num=**16**);

%***univariate***(data=nulli, var=gest_dm, ref=**0**, num=**17**);

%***univariate***(data=nulli, var=preex_hyper, ref=**0**, num=**18**);

%***univariate***(data=nulli, var=gest_hyper, ref=**0**, num=**19**);

%***univariate***(data=nulli, var=placenta_previa, ref=**0**, num=**20**);

%***univariate***(data=nulli, var=placenta_accspec, ref=**0**, num=**21**);

%***univariate***(data=nulli, var=placental_abruption, ref=**0**, num=**22**);

%***univariate***(data=nulli, var=induction, ref=**0**, num=**23**);

%***univariate***(data=nulli, var=augmentation_ind, ref=**0**, num=**24**);

%***univariate***(data=nulli, var=episiotomy, ref=None, num=**25**);

%***univariate***(data=nulli, var=presentation_type, ref=Cephalic, num=**26**);

%***univariate***(data=nulli, var=duration_stage2, ref=<**60** minutes, num=**27**);

**data** duration_stage2_uni_rr;

set duration_stage2_uni_rr;

if Level1 = '60-119 minutes' then place2 = **1**;

else if Level1 in ('120-179 minutes' '180-239 minutes') then place2 = _n_ + **1**;

else place2 = _n_;

**run**;

%***univariate***(data=nulli, var=mode_of_delivery, ref=(**01**) SVD, num=**28**);

%***univariate***(data=nulli, var=gestage_cat, ref=(**2**) Term, num=**29**);

%***univariate***(data=nulli, var=b_weight_g_cat1, ref=(**2**) **3000**-**3999**, num=**30**);

%***univariate***(data=nulli, var=b_headcirc_cm_cat1, ref=(**2**) **33**-**34**, num=**31**);

**data** nulli_uni_rr;

retain Variable Level1;

length Level1 $ **30.**;

set ancestry_uni_rr age_group_uni_rr plurality_uni_rr primary_language_uni_rr maternal_bmi_cat_uni_rr age_labourforce_q_da_uni_rr

material_resources_q_da_uni_rr households_dwellings_q_da_uni_rr immig_cat2_uni_rr immig_birth_datediff_cat_uni_rr rural_pccf_uni_rr

preg_smoking_uni_rr preg_drug_exposure_uni_rr conception_type_uni_rr tri1_prenatal_visit_uni_rr preex_dm_uni_rr gest_dm_uni_rr

preex_hyper_uni_rr gest_hyper_uni_rr placenta_previa_uni_rr placenta_accspec_uni_rr placental_abruption_uni_rr induction_uni_rr

augmentation_ind_uni_rr episiotomy_uni_rr presentation_type_uni_rr duration_stage2_uni_rr mode_of_delivery_uni_rr gestage_cat_uni_rr

b_weight_g_cat1_uni_rr b_headcirc_cm_cat1_uni_rr;

rename est=uni_est lower=uni_lower upper=uni_upper pval=uni_pval;

**run**;

**proc** **sort** data=nulli_uni_rr out=nulli_uni_rr(drop=place1 place2); by place1 place2; **run**;

**data** nulli_uni_rr_reduced;

retain Variable Level1;

length Level1 $ **30.**;

set ancestry_uni_rr age_group_uni_rr plurality_uni_rr primary_language_uni_rr maternal_bmi_cat_uni_rr material_resources_q_da_uni_rr immig_cat2_uni_rr

rural_pccf_uni_rr preg_smoking_uni_rr preg_drug_exposure_uni_rr conception_type_uni_rr tri1_prenatal_visit_uni_rr preex_dm_uni_rr gest_dm_uni_rr

preex_hyper_uni_rr gest_hyper_uni_rr induction_uni_rr augmentation_ind_uni_rr presentation_type_uni_rr duration_stage2_uni_rr

mode_of_delivery_uni_rr b_weight_g_cat1_uni_rr;

rename est=uni_est lower=uni_lower upper=uni_upper pval=uni_pval;

**run**;

**proc** **sort** data=nulli_uni_rr_reduced out=nulli_uni_rr_reduced(drop=place1 place2); by place1 place2; **run**;

**proc** **genmod** data=nulli;

class ikn ancestry(ref="Caucasian") age_group(ref="20-24") plurality(ref="0") primary_language(ref="English/French")

maternal_bmi_cat(ref="18.5-24.9") age_labourforce_q_da(ref="1") material_resources_q_da(ref="1") households_dwellings_q_da(ref="1")

immig_cat2(ref="(1) Not an immigrant") immig_birth_datediff_cat(ref="(5) >10 years") rural_pccf(ref="N") preg_smoking(ref="0")

preg_drug_exposure(ref="0") conception_type(ref="Spontaneous") tri1_prenatal_visit(ref="1") preex_dm(ref="0") gest_dm(ref="0")

preex_hyper(ref="0") gest_hyper(ref="0") placenta_previa(ref="0") placenta_accspec(ref="0") placental_abruption(ref="0")

induction(ref="0") augmentation_ind(ref="0") episiotomy(ref="None") presentation_type(ref="Cephalic") duration_stage2(ref="<60 minutes")

mode_of_delivery(ref="(01) SVD") gestage_cat(ref="(2) Term") b_weight_g_cat1(ref="(2) 3000-3999") b_headcirc_cm_cat1(ref="(2) 33-34");

model born_dad_pph = ancestry age_group plurality primary_language maternal_bmi_cat age_labourforce_q_da material_resources_q_da

households_dwellings_q_da immig_cat2 immig_birth_datediff_cat rural_pccf preg_smoking preg_drug_exposure conception_type

tri1_prenatal_visit preex_dm gest_dm preex_hyper gest_hyper placenta_previa placenta_accspec placental_abruption induction

augmentation_ind episiotomy presentation_type duration_stage2 mode_of_delivery gestage_cat b_weight_g_cat1 b_headcirc_cm_cat1

/ dist=poisson link=log;

repeated subject=ikn;

ods output Genmod.GEEEmpPEst = nulli_multi_rr;

**run**;

**data** nulli_multi_rr;

retain Parm Level1 adj_est adj_lower adj_upper ProbZ;

set nulli_multi_rr;

if _n_ = **1** or _n_ = **5** or _n_ = **11** or _n_ = **13** or _n_ = **16** or _n_ = **23** or _n_ = **29** or _n_ = **35** or _n_ = **41** or _n_ = **46** or _n_ = **52** or _n_ = **55**

or _n_ = **58** or _n_ = **61** or _n_ = **64** or _n_ = **67** or _n_ = **69** or _n_ = **71** or _n_ = **73** or _n_ = **75** or _n_ = **77** or _n_ = **79** or _n_ = **81** or _n_ = **83**

or _n_ = **85** or _n_ = **89** or _n_ = **93** or _n_ = **99** or _n_ = **108** or _n_ = **111** or _n_ = **116** or _n_ = **121** then delete;

adj_est = round(exp(Estimate), **0.0001**);

adj_lower = round(exp(LowerCL), **0.0001**);

adj_upper = round(exp(UpperCL), **0.0001**);

keep Parm Level1 adj_est adj_lower adj_upper ProbZ;

rename ProbZ = adj_pval;

**run**;

**data** nulli_multi_rr;

set nulli_multi_rr;

if Parm = 'age_group' then do;

if Level1 = '<20' then place = _n_ - **4**;

else place = _n_ + **1**;

end;

else if Parm = 'maternal_bmi_cat' then do;

if Level1 = '<18.5' then place = _n_ - **3**;

else if Level1 in ('>=40' 'Unknown') then place = _n_;

else place = _n_ + **1**;

end;

else if Parm = 'duration_stage2' then do;

if Level1 = '60-119 minutes' then place = _n_ - **2**;

else if Level1 in ('120-179 minutes' '180-239 minutes') then place = _n_ + **1**;

else place = _n_;

end;

else place = _n_;

**run**;

**proc** **sort** data=nulli_multi_rr out=nulli_multi_rr(drop=place); by place; **run**;

**data** tables.model_results_nulli;

merge nulli_uni_rr nulli_multi_rr;

drop Parm place:;

**run**;

**proc** **genmod** data=nulli;

class ikn ancestry(ref="Caucasian") age_group(ref="20-24") plurality(ref="0") primary_language(ref="English")

maternal_bmi_cat(ref="18.5-24.9") material_resources_q_da(ref="1") immig_cat2(ref="(1) Not an immigrant")

rural_pccf(ref="N") preg_smoking(ref="0") preg_drug_exposure(ref="0") conception_type(ref="Spontaneous") tri1_prenatal_visit(ref="1")

preex_dm(ref="0") gest_dm(ref="0") preex_hyper(ref="0") gest_hyper(ref="0") induction(ref="0") augmentation_ind(ref="0")

presentation_type(ref="Cephalic") duration_stage2(ref="<60 minutes") mode_of_delivery(ref="(01) SVD") b_weight_g_cat1(ref="(2) 3000-3999");

model born_dad_pph = ancestry age_group primary_language plurality maternal_bmi_cat material_resources_q_da immig_cat2

rural_pccf preg_smoking preg_drug_exposure conception_type tri1_prenatal_visit preex_dm gest_dm preex_hyper gest_hyper induction

augmentation_ind presentation_type duration_stage2 mode_of_delivery b_weight_g_cat1 / dist=poisson link=log;

repeated subject=ikn;

ods output Genmod.GEEEmpPEst = nulli_multi_rr_reduced;

**run**;

**data** nulli_multi_rr_reduced;

retain Parm Level1 adj_est adj_lower adj_upper ProbZ;

set nulli_multi_rr_reduced;

if Parm = 'Intercept' then delete;

*if Estimate = 0 and Stderr = 0 and LowerCL = 0 and UpperCL = 0 then delete;

adj_est = round(exp(Estimate), **0.0001**);

adj_lower = round(exp(LowerCL), **0.0001**);

adj_upper = round(exp(UpperCL), **0.0001**);

keep Parm Level1 adj_est adj_lower adj_upper ProbZ;

rename ProbZ = adj_pval;

**run**;

**data** nulli_multi_rr_reduced;

set nulli_multi_rr_reduced;

if Parm = 'ancestry' then do;

if Level1 = 'Caucasian' then place = _n_ - **1**;

else if Level1 = 'Other' then place = _n_ + **1**;

else place = _n_;

end;

else if Parm = 'age_group' then do;

if Level1 in ('<20' '20-24') then place = _n_ - **4**;

else place = _n_ + **2**;

end;

else if Parm = 'primary_language' then do;

if Level1 = 'English' then place = _n_ - **3**;

else place = _n_ + **1**;

end;

else if Parm = 'maternal_bmi_cat' then do;

if Level1 = '<18.5' then place = _n_ - **3**;

else if Level1 = '18.5-24.9' then place = _n_ - **5**;

else if Level1 in ('>=40' 'Unknown') then place = _n_ + **1**;

else place = _n_ + **2**;

end;

else if Parm = 'material_resources_q' then do;

if Level1 = '1' then place = _n_ - **5**;

else place = _n_ + **1**;

end;

else if Parm = 'immig_cat2' then do;

if Level1 = '(1) Not an immigrant' then place = _n_ - **4**;

else place = _n_ + **1**;

end;

else if Parm = 'rural_pccf' then do;

if Level1 = 'N' then place = _n_ - **2**;

else place = _n_ + **1**;

end;

else if Parm in ('preg_smoking' 'preg_drug_exposure') then do;

if Level1 = '0' then place = _n_ - **2**;

else place = _n_ + **1**;

end;

else if Parm = 'tri1_prenatal_visit' then do;

if Level1 = '1' then place = _n_ - **1**;

else if Level1 = '0' then place = _n_;

else place = _n_ + **1**;

end;

else if Parm = 'conception_type' then do;

if Level1 = 'Spontaneous' then place = _n_ - **2**;

else place = _n_ + **1**;

end;

else if Parm in ('preex_dm' 'gest_dm' 'preex_hyper' 'gest_hyper' 'induction' 'augmentation_ind') then do;

if Level1 = '0' then place = _n_ - **1**;

else place = _n_ + **1**;

end;

else if Parm = 'presentation_type' then do;

if Level1 = 'Cephalic' then place = _n_ - **2**;

else if Level1 = 'Breech' then place = _n_;

else place = _n_ + **1**;

end;

else if Parm = 'duration_stage2' then do;

if Level1 = '<60 minutes' then place = _n_ - **5**;

else if Level1 = '60-119 minutes' then place = _n_ - **1**;

else if Level1 in ('120-179 minutes' '180-239 minutes') then place = _n_ + **2**;

else place = _n_ + **1**;

end;

else if Parm = 'mode_of_delivery' then do;

if Level1 = '(01) SVD' then place = _n_ - **8**;

else place = _n_ + **1**;

end;

else if Parm = 'b_weight_g_cat1' then do;

if Level1 = '(1) <3000' then place = _n_;

else if Level1 = '(2) 3000-3999' then place = _n_ - **3**;

else place = _n_ + **1**;

end;

else place = _n_;

**run**;

**proc** **sort** data=nulli_multi_rr_reduced out=nulli_multi_rr_reduced(drop=place); by place; **run**;

**data** nulli_multi_rr_reduced;

set nulli_multi_rr_reduced;

**run**;

**data** tables.model_results_nulli_reduced;

merge nulli_uni_rr_reduced nulli_multi_rr_reduced;

drop Parm place:;

**run**;

**proc** **freq** data=nulli; tables primary_language*born_dad_pph / out=nulli_lang; **run**;

**data** nulli_pph(rename=(count=pph)) nulli_nopph(rename=(count=nopph));

set nulli_lang;

if born_dad_pph = **1** then output nulli_pph;

else output nulli_nopph;

drop born_dad_pph percent;

**run**;

**data** nulli_lang;

merge nulli_pph nulli_nopph;

by primary_language;

percent = pph***100**/(pph+nopph);

pph_pc = catx(' (', pph, cats(round(percent, **0.1**), '%)'));

drop nopph pph percent;

**run**;

**proc** **freq** data=nulli; tables ancestry*primary_language*born_dad_pph / out=nulli_crosstab; **run**;

**data** nulli_pph(rename=(count=pph)) nulli_nopph(rename=(count=nopph));

set nulli_crosstab;

if born_dad_pph = **1** then output nulli_pph;

else output nulli_nopph;

drop born_dad_pph percent;

**run**;

**data** nulli_crosstab;

merge nulli_pph nulli_nopph;

by ancestry primary_language;

percent = pph***100**/(pph+nopph);

pph_pc = catx(' (', pph, cats(round(percent, **0.1**), '%)'));

drop nopph pph percent;

**run**;

**proc** **genmod** data=nulli;

class ikn ancestry(ref="Caucasian") age_group(ref="20-24") plurality(ref="0") primary_language(ref="English")

maternal_bmi_cat(ref="18.5-24.9") material_resources_q_da(ref="1") immig_cat2(ref="(1) Not an immigrant")

rural_pccf(ref="N") preg_smoking(ref="0") preg_drug_exposure(ref="0") conception_type(ref="Spontaneous") tri1_prenatal_visit(ref="1")

preex_dm(ref="0") gest_dm(ref="0") preex_hyper(ref="0") gest_hyper(ref="0") induction(ref="0") augmentation_ind(ref="0")

presentation_type(ref="Cephalic") duration_stage2(ref="<60 minutes") mode_of_delivery(ref="(01) SVD") b_weight_g_cat1(ref="(2) 3000-3999");

model born_dad_pph = ancestry primary_language ancestry*primary_language age_group plurality maternal_bmi_cat material_resources_q_da immig_cat2

rural_pccf preg_smoking preg_drug_exposure conception_type tri1_prenatal_visit preex_dm gest_dm preex_hyper gest_hyper induction

augmentation_ind presentation_type duration_stage2 mode_of_delivery b_weight_g_cat1 / dist=poisson link=log;

repeated subject=ikn;

ods output Genmod.GEEEmpPEst = nulli_multi_rr_int;

**run**;

**data** nulli_multi_rr_int;

retain Parm Level1 Level2 adj_est adj_lower adj_upper ProbZ;

set nulli_multi_rr_int;

if _n_ = **1** then delete;

*if Estimate = 0 and Stderr = 0 and LowerCL = 0 and UpperCL = 0 then delete;

adj_est = round(exp(Estimate), **0.0001**);

adj_lower = round(exp(LowerCL), **0.0001**);

adj_upper = round(exp(UpperCL), **0.0001**);

keep Parm Level1 Level2 adj_est adj_lower adj_upper ProbZ;

rename ProbZ = adj_pval;

**run**;

**data** nulli_multi_rr_int;

set nulli_multi_rr_int;

if Parm = 'ancestry' then do;

if Level1 = 'Caucasian' then place = _n_ - **1**;

else if Level1 = 'Other' then place = _n_ + **1**;

else place = _n_;

end;

else if Parm = 'primary_language' then do;

if Level1 = 'English' then place = _n_ - **3**;

else place = _n_ + **1**;

end;

else if Parm = 'ancestry*primary_lan' then do;

if Level1 in ('Asian' 'Black') then do;

if Level2 = 'English' then place = _n_ - **3**;

else place = _n_ + **1**;

end;

else if Level1 = 'Caucasian' then do;

if Level2 = 'English' then place = _n_ - **7**;

else place = _n_ - **3**;

end;

else do;

if Level2 = 'English' then place = _n_ + **1**;

else place = _n_ + **5**;

end;

end;

else if Parm = 'age_group' then do;

if Level1 = '<20' then place = _n_ - **4**;

else if Level1 = '20-24' then place = _n_ - **4**;

else place = _n_ + **2**;

end;

else if Parm = 'maternal_bmi_cat' then do;

if Level1 = '<18.5' then place = _n_ - **3**;

else if Level1 = '18.5-24.9' then place = _n_ - **5**;

else if Level1 in ('>=40' 'Unknown') then place = _n_ + **1**;

else place = _n_ + **2**;

end;

else if Parm = 'material_resources_q' then do;

if Level1 = '1' then place = _n_ - **5**;

else place = _n_ + **1**;

end;

else if Parm = 'immig_cat2' then do;

if Level1 = '(1) Not an immigrant' then place = _n_ - **4**;

else place = _n_ + **1**;

end;

else if Parm = 'rural_pccf' then do;

if Level1 = 'N' then place = _n_ - **2**;

else place = _n_ + **1**;

end;

else if Parm in ('preg_smoking' 'preg_drug_exposure') then do;

if Level1 = '0' then place = _n_ - **2**;

else place = _n_ + **1**;

end;

else if Parm = 'tri1_prenatal_visit' then do;

if Level1 = '1' then place = _n_ - **1**;

else if Level1 = '0' then place = _n_;

else place = _n_ + **1**;

end;

else if Parm = 'conception_type' then do;

if Level1 = 'Spontaneous' then place = _n_ - **2**;

else place = _n_ + **1**;

end;

else if Parm in ('preex_dm' 'gest_dm' 'preex_hyper' 'gest_hyper' 'induction' 'augmentation_ind') then do;

if Level1 = '0' then place = _n_ - **1**;

else place = _n_ + **1**;

end;

else if Parm = 'presentation_type' then do;

if Level1 = 'Cephalic' then place = _n_ - **2**;

else if Level1 = 'Breech' then place = _n_;

else place = _n_ + **1**;

end;

else if Parm = 'duration_stage2' then do;

if Level1 = '<60 minutes' then place = _n_ - **5**;

else if Level1 = '60-119 minutes' then place = _n_ - **1**;

else if Level1 in ('120-179 minutes' '180-239 minutes') then place = _n_ + **2**;

else place = _n_ + **1**;

end;

else if Parm = 'mode_of_delivery' then do;

if Level1 = '(01) SVD' then place = _n_ - **8**;

else place = _n_ + **1**;

end;

else if Parm = 'b_weight_g_cat1' then do;

if Level1 = '(1) <3000' then place = _n_;

else if Level1 = '(2) 3000-3999' then place = _n_ - **3**;

else place = _n_ + **1**;

end;

else place = _n_;

**run**;

**proc** **sort** data=nulli_multi_rr_int out=nulli_multi_rr_int(drop=place); by place; **run**;

%***univariate***(data=parous, var=ancestry, ref=Caucasian, num=**1**);

%***univariate***(data=parous, var=age_group, ref=**20**-**24**, num=**2**);

**data** age_group_uni_rr;

set age_group_uni_rr;

if Level1 = '<20' then place2 = **1**;

else place2 = _n_ + **1**;

**run**;

%***univariate***(data=parous, var=plurality, ref=**0**, num=**3**);

%***univariate***(data=parous, var=primary_language, ref=English, num=**4**);

%***univariate***(data=parous, var=maternal_bmi_cat, ref=**18.5**-**24.9**, num=**5**);

**data** maternal_bmi_cat_uni_rr;

set maternal_bmi_cat_uni_rr;

if Level1 = '<18.5' then place2 = **1**;

else if Level1 not in ('>=40' 'Unknown') then place2 = _n_ + **1**;

else place2 = _n_;

**run**;

%***univariate***(data=parous, var=age_labourforce_q_da, ref=**1**, num=**6**);

%***univariate***(data=parous, var=material_resources_q_da, ref=**1**, num=**7**);

%***univariate***(data=parous, var=households_dwellings_q_da, ref=**1**, num=**8**);

%***univariate***(data=parous, var=immig_cat2, ref=(**1**) Not an immigrant, num=**9**);

%***univariate***(data=parous, var=immig_birth_datediff_cat, ref=(**5**) >**10** years, num=**10**);

%***univariate***(data=parous, var=rural_pccf, ref=N, num=**11**);

%***univariate***(data=parous, var=preg_smoking, ref=**0**, num=**12**);

%***univariate***(data=parous, var=preg_drug_exposure, ref=**0**, num=**13**);

%***univariate***(data=parous, var=conception_type, ref=Spontaneous, num=**14**);

%***univariate***(data=parous, var=tri1_prenatal_visit, ref=**1**, num=**15**);

%***univariate***(data=parous, var=preex_dm, ref=**0**, num=**16**);

%***univariate***(data=parous, var=gest_dm, ref=**0**, num=**17**);

%***univariate***(data=parous, var=preex_hyper, ref=**0**, num=**18**);

%***univariate***(data=parous, var=gest_hyper, ref=**0**, num=**19**);

%***univariate***(data=parous, var=previous_cd, ref=**0**, num=**20**);

%***univariate***(data=parous, var=placenta_previa, ref=**0**, num=**21**);

%***univariate***(data=parous, var=placenta_accspec, ref=**0**, num=**22**);

%***univariate***(data=parous, var=placental_abruption, ref=**0**, num=**23**);

%***univariate***(data=parous, var=induction, ref=**0**, num=**24**);

%***univariate***(data=parous, var=augmentation_ind, ref=**0**, num=**25**);

%***univariate***(data=parous, var=episiotomy, ref=None, num=**26**);

%***univariate***(data=parous, var=presentation_type, ref=Cephalic, num=**27**);

%***univariate***(data=parous, var=duration_stage2, ref=<**60** minutes, num=**28**);

**data** duration_stage2_uni_rr;

set duration_stage2_uni_rr;

if Level1 = '60-119 minutes' then place2 = **1**;

else if Level1 in ('120-179 minutes' '180-239 minutes') then place2 = _n_ + **1**;

else place2 = _n_;

**run**;

%***univariate***(data=parous, var=mode_of_delivery, ref=(**01**) SVD, num=**29**);

%***univariate***(data=parous, var=gestage_cat, ref=(**2**) Term, num=**30**);

%***univariate***(data=parous, var=b_weight_g_cat1, ref=(**2**) **3000**-**3999**, num=**31**);

%***univariate***(data=parous, var=b_headcirc_cm_cat1, ref=(**2**) **33**-**34**, num=**32**);

**data** parous_uni_rr;

retain Variable Level1;

length Level1 $ **30.**;

set ancestry_uni_rr age_group_uni_rr plurality_uni_rr primary_language_uni_rr maternal_bmi_cat_uni_rr age_labourforce_q_da_uni_rr

material_resources_q_da_uni_rr households_dwellings_q_da_uni_rr immig_cat2_uni_rr immig_birth_datediff_cat_uni_rr rural_pccf_uni_rr preg_smoking_uni_rr

preg_drug_exposure_uni_rr conception_type_uni_rr tri1_prenatal_visit_uni_rr preex_dm_uni_rr gest_dm_uni_rr preex_hyper_uni_rr gest_hyper_uni_rr

previous_cd_uni_rr placenta_previa_uni_rr placenta_accspec_uni_rr placental_abruption_uni_rr induction_uni_rr augmentation_ind_uni_rr episiotomy_uni_rr

presentation_type_uni_rr duration_stage2_uni_rr mode_of_delivery_uni_rr gestage_cat_uni_rr b_weight_g_cat1_uni_rr b_headcirc_cm_cat1_uni_rr;

rename est=uni_est lower=uni_lower upper=uni_upper pval=uni_pval;

**run**;

**proc** **sort** data=parous_uni_rr out=parous_uni_rr(drop=place1 place2); by place1 place2; **run**;

**data** parous_uni_rr_reduced;

retain Variable Level1;

length Level1 $ **30.**;

set ancestry_uni_rr age_group_uni_rr plurality_uni_rr primary_language_uni_rr maternal_bmi_cat_uni_rr material_resources_q_da_uni_rr immig_cat2_uni_rr

rural_pccf_uni_rr preg_smoking_uni_rr preg_drug_exposure_uni_rr conception_type_uni_rr tri1_prenatal_visit_uni_rr preex_dm_uni_rr gest_dm_uni_rr

preex_hyper_uni_rr gest_hyper_uni_rr previous_cd_uni_rr induction_uni_rr augmentation_ind_uni_rr presentation_type_uni_rr duration_stage2_uni_rr

mode_of_delivery_uni_rr b_weight_g_cat1_uni_rr;

rename est=uni_est lower=uni_lower upper=uni_upper pval=uni_pval;

**run**;

**proc** **sort** data=parous_uni_rr_reduced out=parous_uni_rr_reduced(drop=place1 place2); by place1 place2; **run**;

**proc** **genmod** data=parous;

class ikn ancestry(ref="Caucasian") age_group(ref="20-24") plurality(ref="0") primary_language(ref="English/French")

maternal_bmi_cat(ref="18.5-24.9") age_labourforce_q_da(ref="1") material_resources_q_da(ref="1") households_dwellings_q_da(ref="1") immig_cat2(ref="(1) Not an immigrant")

immig_birth_datediff_cat(ref="(5) >10 years") rural_pccf(ref="N") preg_smoking(ref="0") preg_drug_exposure(ref="0") conception_type(ref="Spontaneous")

tri1_prenatal_visit(ref="1") preex_dm(ref="0") gest_dm(ref="0") preex_hyper(ref="0") gest_hyper(ref="0") previous_cd(ref="0") placenta_previa(ref="0")

placenta_accspec(ref="0") placental_abruption(ref="0") induction(ref="0") augmentation_ind(ref="0") episiotomy(ref="None")

presentation_type(ref="Cephalic") duration_stage2(ref="<60 minutes") mode_of_delivery(ref="(01) SVD") gestage_cat(ref="(2) Term")

b_weight_g_cat1(ref="(2) 3000-3999") b_headcirc_cm_cat1(ref="(2) 33-34");

model born_dad_pph = ancestry age_group plurality primary_language maternal_bmi_cat age_labourforce_q_da material_resources_q_da households_dwellings_q_da immig_cat2

immig_birth_datediff_cat rural_pccf preg_smoking preg_drug_exposure conception_type tri1_prenatal_visit preex_dm gest_dm preex_hyper gest_hyper

previous_cd placenta_previa placenta_accspec placental_abruption induction augmentation_ind episiotomy presentation_type duration_stage2

mode_of_delivery gestage_cat b_weight_g_cat1 b_headcirc_cm_cat1 / dist=poisson link=log;

repeated subject=ikn;

ods output Genmod.GEEEmpPEst = parous_multi_rr;

**run**;

**data** parous_multi_rr;

retain Parm Level1 adj_est adj_lower adj_upper ProbZ;

set parous_multi_rr;

if _n_ = **1** or _n_ = **5** or _n_ = **11** or _n_ = **13** or _n_ = **16** or _n_ = **23** or _n_ = **29** or _n_ = **35** or _n_ = **41** or _n_ = **46** or _n_ = **52**

or _n_ = **55** or _n_ = **58** or _n_ = **61** or _n_ = **64** or _n_ = **67** or _n_ = **69** or _n_ = **71** or _n_ = **73** or _n_ = **75** or _n_ = **77** or _n_ = **79**

or _n_ = **81** or _n_ = **83** or _n_ = **85** or _n_ = **87** or _n_ = **91** or _n_ = **95** or _n_ = **101** or _n_ = **110** or _n_ = **113** or _n_ = **118**

or _n_ = **123** then delete;

adj_est = round(exp(Estimate), **0.0001**);

adj_lower = round(exp(LowerCL), **0.0001**);

adj_upper = round(exp(UpperCL), **0.0001**);

keep Parm Level1 adj_est adj_lower adj_upper ProbZ;

rename ProbZ = adj_pval;

**run**;

**data** parous_multi_rr;

set parous_multi_rr;

if Parm = 'age_group' then do;

if Level1 = '<20' then place = _n_ - **4**;

else place = _n_ + **1**;

end;

else if Parm = 'maternal_bmi_cat' then do;

if Level1 = '<18.5' then place = _n_ - **3**;

else if Level1 in ('>=40' 'Unknown') then place = _n_;

else place = _n_ + **1**;

end;

else if Parm = 'duration_stage2' then do;

if Level1 = '60-119 minutes' then place = _n_ - **2**;

else if Level1 in ('120-179 minutes' '180-239 minutes') then place = _n_ + **1**;

else place = _n_;

end;

else place = _n_;

**run**;

**proc** **sort** data=parous_multi_rr out=parous_multi_rr(drop=place); by place; **run**;

**data** tables.model_results_parous;

merge parous_uni_rr parous_multi_rr;

drop place: Parm;

**run**;

**proc** **genmod** data=parous;

class ikn ancestry(ref="Caucasian") age_group(ref="20-24") plurality(ref="0") primary_language(ref="English")

maternal_bmi_cat(ref="18.5-24.9") material_resources_q_da(ref="1") immig_cat2(ref="(1) Not an immigrant")

rural_pccf(ref="N") preg_smoking(ref="0") preg_drug_exposure(ref="0") conception_type(ref="Spontaneous") tri1_prenatal_visit(ref="1")

preex_dm(ref="0") gest_dm(ref="0") preex_hyper(ref="0") gest_hyper(ref="0") previous_cd(ref="0") induction(ref="0") augmentation_ind(ref="0")

presentation_type(ref="Cephalic") duration_stage2(ref="<60 minutes") mode_of_delivery(ref="(01) SVD") b_weight_g_cat1(ref="(2) 3000-3999");

model born_dad_pph = ancestry age_group plurality primary_language maternal_bmi_cat material_resources_q_da immig_cat2 rural_pccf

preg_smoking preg_drug_exposure conception_type tri1_prenatal_visit preex_dm gest_dm preex_hyper gest_hyper previous_cd induction augmentation_ind

presentation_type duration_stage2 mode_of_delivery b_weight_g_cat1 / dist=poisson link=log;

repeated subject=ikn;

ods output Genmod.GEEEmpPEst = parous_multi_rr_reduced;

**run**;

**data** parous_multi_rr_reduced;

retain Parm Level1 adj_est adj_lower adj_upper ProbZ;

set parous_multi_rr_reduced;

if Parm = 'Intercept' then delete;

adj_est = round(exp(Estimate), **0.0001**);

adj_lower = round(exp(LowerCL), **0.0001**);

adj_upper = round(exp(UpperCL), **0.0001**);

keep Parm Level1 adj_est adj_lower adj_upper ProbZ;

rename ProbZ = adj_pval;

**run**;

**data** parous_multi_rr_reduced;

set parous_multi_rr_reduced;

if Parm = 'ancestry' then do;

if Level1 = 'Caucasian' then place = _n_ - **1**;

else if Level1 = 'Other' then place = _n_ + **1**;

else place = _n_;

end;

else if Parm = 'age_group' then do;

if Level1 in ('<20' '20-24') then place = _n_ - **4**;

else place = _n_ + **2**;

end;

else if Parm = 'primary_language' then do;

if Level1 = 'English' then place = _n_ - **3**;

else place = _n_ + **1**;

end;

else if Parm = 'maternal_bmi_cat' then do;

if Level1 = '<18.5' then place = _n_ - **3**;

else if Level1 = '18.5-24.9' then place = _n_ - **5**;

else if Level1 in ('>=40' 'Unknown') then place = _n_ + **1**;

else place = _n_ + **2**;

end;

else if Parm = 'material_resources_q' then do;

if Level1 = '1' then place = _n_ - **5**;

else place = _n_ + **1**;

end;

else if Parm = 'immig_cat2' then do;

if Level1 = '(1) Not an immigrant' then place = _n_ - **4**;

else place = _n_ + **1**;

end;

else if Parm = 'rural_pccf' then do;

if Level1 = 'N' then place = _n_ - **2**;

else place = _n_ + **1**;

end;

else if Parm in ('preg_smoking' 'preg_drug_exposure') then do;

if Level1 = '0' then place = _n_ - **2**;

else place = _n_ + **1**;

end;

else if Parm = 'tri1_prenatal_visit' then do;

if Level1 = '1' then place = _n_ - **1**;

else if Level1 = '0' then place = _n_;

else place = _n_ + **1**;

end;

else if Parm = 'conception_type' then do;

if Level1 = 'Spontaneous' then place = _n_ - **2**;

else place = _n_ + **1**;

end;

else if Parm in ('preex_dm' 'gest_dm' 'preex_hyper' 'gest_hyper' 'induction' 'augmentation_ind') then do;

if Level1 = '0' then place = _n_ - **1**;

else place = _n_ + **1**;

end;

else if Parm = 'presentation_type' then do;

if Level1 = 'Cephalic' then place = _n_ - **2**;

else if Level1 = 'Breech' then place = _n_;

else place = _n_ + **1**;

end;

else if Parm = 'duration_stage2' then do;

if Level1 = '<60 minutes' then place = _n_ - **5**;

else if Level1 = '60-119 minutes' then place = _n_ - **1**;

else if Level1 in ('120-179 minutes' '180-239 minutes') then place = _n_ + **2**;

else place = _n_ + **1**;

end;

else if Parm = 'mode_of_delivery' then do;

if Level1 = '(01) SVD' then place = _n_ - **8**;

else place = _n_ + **1**;

end;

else if Parm = 'b_weight_g_cat1' then do;

if Level1 = '(1) <3000' then place = _n_;

else if Level1 = '(2) 3000-3999' then place = _n_ - **3**;

else place = _n_ + **1**;

end;

else place = _n_;

**run**;

**proc** **sort** data=parous_multi_rr_reduced out=parous_multi_rr_reduced(drop=place); by place; **run**;

**data** tables.model_results_parous_reduced;

merge parous_uni_rr_reduced parous_multi_rr_reduced;

drop Parm place:;

**run**;

**proc** **freq** data=parous; tables primary_language*born_dad_pph / out=parous_lang; **run**;

**data** parous_pph(rename=(count=pph)) parous_nopph(rename=(count=nopph));

set parous_lang;

if born_dad_pph = **1** then output parous_pph;

else output parous_nopph;

drop born_dad_pph percent;

**run**;

**data** parous_lang;

merge parous_pph parous_nopph;

by primary_language;

percent = pph***100**/(pph+nopph);

pph_pc = catx(' (', pph, cats(round(percent, **0.1**), '%)'));

drop nopph pph percent;

**run**;

**proc** **freq** data=parous; tables ancestry*primary_language*born_dad_pph / out=parous_crosstab; **run**;

**data** parous_pph(rename=(count=pph)) parous_nopph(rename=(count=nopph));

set parous_crosstab;

if born_dad_pph = **1** then output parous_pph;

else output parous_nopph;

drop born_dad_pph percent;

**run**;

**data** parous_crosstab;

merge parous_pph parous_nopph;

by ancestry primary_language;

percent = pph***100**/(pph+nopph);

pph_pc = catx(' (', pph, cats(round(percent, **0.1**), '%)'));

drop nopph pph percent;

**run**;

**proc** **genmod** data=parous;

class ikn ancestry(ref="Caucasian") age_group(ref="20-24") plurality(ref="0") primary_language(ref="English")

maternal_bmi_cat(ref="18.5-24.9") material_resources_q_da(ref="1") immig_cat2(ref="(1) Not an immigrant")

rural_pccf(ref="N") preg_smoking(ref="0") preg_drug_exposure(ref="0") conception_type(ref="Spontaneous") tri1_prenatal_visit(ref="1")

preex_dm(ref="0") gest_dm(ref="0") preex_hyper(ref="0") gest_hyper(ref="0") previous_cd(ref="0") induction(ref="0") augmentation_ind(ref="0")

presentation_type(ref="Cephalic") duration_stage2(ref="<60 minutes") mode_of_delivery(ref="(01) SVD") b_weight_g_cat1(ref="(2) 3000-3999");

model born_dad_pph = ancestry primary_language ancestry*primary_language age_group plurality maternal_bmi_cat material_resources_q_da

immig_cat2 rural_pccf preg_smoking preg_drug_exposure conception_type tri1_prenatal_visit preex_dm gest_dm preex_hyper gest_hyper

previous_cd induction augmentation_ind presentation_type duration_stage2 mode_of_delivery b_weight_g_cat1 / dist=poisson link=log;

repeated subject=ikn;

ods output Genmod.GEEEmpPEst = parous_multi_rr_int;

**run**;

**data** parous_multi_rr_int;

retain Parm Level1 Level2 adj_est adj_lower adj_upper ProbZ;

set parous_multi_rr_int;

if _n_ = **1** then delete;

*if Estimate = 0 and Stderr = 0 and LowerCL = 0 and UpperCL = 0 then delete;

adj_est = round(exp(Estimate), **0.0001**);

adj_lower = round(exp(LowerCL), **0.0001**);

adj_upper = round(exp(UpperCL), **0.0001**);

keep Parm Level1 Level2 adj_est adj_lower adj_upper ProbZ;

rename ProbZ = adj_pval;

**run**;

**data** parous_multi_rr_int;

set parous_multi_rr_int;

if Parm = 'ancestry' then do;

if Level1 = 'Caucasian' then place = _n_ - **1**;

else if Level1 = 'Other' then place = _n_ + **1**;

else place = _n_;

end;

else if Parm = 'primary_language' then do;

if Level1 = 'English' then place = _n_ - **3**;

else place = _n_ + **1**;

end;

else if Parm = 'ancestry*primary_lan' then do;

if Level1 in ('Asian' 'Black') then do;

if Level2 = 'English' then place = _n_ - **3**;

else place = _n_ + **1**;

end;

else if Level1 = 'Caucasian' then do;

if Level2 = 'English' then place = _n_ - **7**;

else place = _n_ - **3**;

end;

else do;

if Level2 = 'English' then place = _n_ + **1**;

else place = _n_ + **5**;

end;

end;

else if Parm = 'age_group' then do;

if Level1 = '<20' then place = _n_ - **4**;

else if Level1 = '20-24' then place = _n_ - **4**;

else place = _n_ + **2**;

end;

else if Parm = 'maternal_bmi_cat' then do;

if Level1 = '<18.5' then place = _n_ - **3**;

else if Level1 = '18.5-24.9' then place = _n_ - **5**;

else if Level1 in ('>=40' 'Unknown') then place = _n_ + **1**;

else place = _n_ + **2**;

end;

else if Parm = 'material_resources_q' then do;

if Level1 = '1' then place = _n_ - **5**;

else place = _n_ + **1**;

end;

else if Parm = 'immig_cat2' then do;

if Level1 = '(1) Not an immigrant' then place = _n_ - **4**;

else place = _n_ + **1**;

end;

else if Parm = 'rural_pccf' then do;

if Level1 = 'N' then place = _n_ - **2**;

else place = _n_ + **1**;

end;

else if Parm in ('preg_smoking' 'preg_drug_exposure') then do;

if Level1 = '0' then place = _n_ - **2**;

else place = _n_ + **1**;

end;

else if Parm = 'tri1_prenatal_visit' then do;

if Level1 = '1' then place = _n_ - **1**;

else if Level1 = '0' then place = _n_;

else place = _n_ + **1**;

end;

else if Parm = 'conception_type' then do;

if Level1 = 'Spontaneous' then place = _n_ - **2**;

else place = _n_ + **1**;

end;

else if Parm in ('preex_dm' 'gest_dm' 'preex_hyper' 'gest_hyper' 'previous_cd' 'induction' 'augmentation_ind') then do;

if Level1 = '0' then place = _n_ - **1**;

else place = _n_ + **1**;

end;

else if Parm = 'presentation_type' then do;

if Level1 = 'Cephalic' then place = _n_ - **2**;

else if Level1 = 'Breech' then place = _n_;

else place = _n_ + **1**;

end;

else if Parm = 'duration_stage2' then do;

if Level1 = '<60 minutes' then place = _n_ - **5**;

else if Level1 = '60-119 minutes' then place = _n_ - **1**;

else if Level1 in ('120-179 minutes' '180-239 minutes') then place = _n_ + **2**;

else place = _n_ + **1**;

end;

else if Parm = 'mode_of_delivery' then do;

if Level1 = '(01) SVD' then place = _n_ - **8**;

else place = _n_ + **1**;

end;

else if Parm = 'b_weight_g_cat1' then do;

if Level1 = '(1) <3000' then place = _n_;

else if Level1 = '(2) 3000-3999' then place = _n_ - **3**;

else place = _n_ + **1**;

end;

else place = _n_;

**run**;

**proc** **sort** data=parous_multi_rr_int out=parous_multi_rr_int(drop=place); by place; **run**;

/* Severe PPH outcome with reduced variable list: */

**%macro** uni_severe(data=, var=, ref=, num=);

proc genmod data=&data.;

class ikn &var.(ref="&ref") / param=ref;

model pph_severe = &var. / dist=poisson link=log;

repeated subject=ikn;

ods output Genmod.GEEEmpPEst = &var._uni_rr;

run;

data &var._uni_rr;

retain Variable Level1 est lower upper probz place1;

length Variable $ **24.**;

set &var._uni_rr;

Variable = "&var";

if Parm = 'Intercept' then delete;

est = round(exp(Estimate), **0.0001**);

lower = round(exp(LowerCL), **0.0001**);

upper = round(exp(UpperCL), **0.0001**);

place1 = &num.;

keep Variable Level1 est lower upper probz place1;

rename probz = pval;

run;

data &var._uni_rr;

set &var._uni_rr;

place2 = _n_;

run;

**%mend**;

%***uni_severe***(data=modeldata, var=ancestry, ref=Caucasian, num=**1**);

%***uni_severe***(data=modeldata, var=age_group, ref=**20**-**24**, num=**2**);

**data** age_group_uni_rr;

set age_group_uni_rr;

if Level1 = '<20' then place2 = **1**;

else place2 = _n_ + **1**;

**run**;

%***uni_severe***(data=modeldata, var=parity_bin, ref=**1**, num=**3**);

%***uni_severe***(data=modeldata, var=plurality, ref=**0**, num=**4**);

**data** modeldata;

set modeldata;

if primary_language in ('English' 'French') then primary_language2 = 'English/French';

else primary_language2 = primary_language;

**run**;

%***uni_severe***(data=modeldata, var=primary_language2, ref=English/French, num=**5**);

%***uni_severe***(data=modeldata, var=maternal_bmi_cat, ref=**18.5**-**24.9**, num=**6**);

**data** maternal_bmi_cat_uni_rr;

set maternal_bmi_cat_uni_rr;

if Level1 = '<18.5' then place2 = **1**;

else if Level1 not in ('>=40' 'Unknown') then place2 = _n_ + **1**;

else place2 = _n_;

**run**;

%***uni_severe***(data=modeldata, var=material_resources_q_da, ref=**1**, num=**7**);

%***uni_severe***(data=modeldata, var=immig_cat2, ref=(**1**) Not an immigrant, num=**8**);

%***uni_severe***(data=modeldata, var=rural_pccf, ref=N, num=**9**);

%***uni_severe***(data=modeldata, var=preg_smoking, ref=**0**, num=**10**);

%***uni_severe***(data=modeldata, var=preg_drug_exposure, ref=**0**, num=**11**);

%***uni_severe***(data=modeldata, var=conception_type, ref=Spontaneous, num=**12**);

%***uni_severe***(data=modeldata, var=tri1_prenatal_visit, ref=**1**, num=**13**);

%***uni_severe***(data=modeldata, var=preex_dm, ref=**0**, num=**14**);

%***uni_severe***(data=modeldata, var=gest_dm, ref=**0**, num=**15**);

%***uni_severe***(data=modeldata, var=preex_hyper, ref=**0**, num=**16**);

%***uni_severe***(data=modeldata, var=gest_hyper, ref=**0**, num=**17**);

%***uni_severe***(data=modeldata, var=previous_cd, ref=**0**, num=**18**);

%***uni_severe***(data=modeldata, var=induction, ref=**0**, num=**19**);

%***uni_severe***(data=modeldata, var=augmentation_ind, ref=**0**, num=**20**);

%***uni_severe***(data=modeldata, var=presentation_type, ref=Cephalic, num=**21**);

%***uni_severe***(data=modeldata, var=duration_stage2, ref=<**60** minutes, num=**22**);

**data** duration_stage2_uni_rr;

set duration_stage2_uni_rr;

if Level1 = '60-119 minutes' then place2 = **1**;

else if Level1 in ('120-179 minutes' '180-239 minutes') then place2 = _n_ + **1**;

else place2 = _n_;

**run**;

%***uni_severe***(data=modeldata, var=mode_of_delivery, ref=(**01**) SVD, num=**23**);

%***uni_severe***(data=modeldata, var=b_weight_g_cat1, ref=(**2**) **3000**-**3999**, num=**24**);

**data** uni_rr_severe;

set ancestry_uni_rr age_group_uni_rr parity_bin_uni_rr plurality_uni_rr primary_language2_uni_rr maternal_bmi_cat_uni_rr

material_resources_q_da_uni_rr immig_cat2_uni_rr rural_pccf_uni_rr preg_smoking_uni_rr preg_drug_exposure_uni_rr conception_type_uni_rr

tri1_prenatal_visit_uni_rr preex_dm_uni_rr gest_dm_uni_rr preex_hyper_uni_rr gest_hyper_uni_rr previous_cd_uni_rr induction_uni_rr

augmentation_ind_uni_rr presentation_type_uni_rr duration_stage2_uni_rr mode_of_delivery_uni_rr b_weight_g_cat1_uni_rr;

rename est=uni_est lower=uni_lower upper=uni_upper pval=uni_pval;

**run**;

**proc** **sort** data=uni_rr_severe out=uni_rr_severe(drop=place:); by place1 place2; **run**;

**proc** **genmod** data=modeldata;

class ikn ancestry(ref="Caucasian") age_group(ref="20-24") parity_bin(ref="1") plurality(ref="0") primary_language2(ref="English/French")

maternal_bmi_cat(ref="18.5-24.9") material_resources_q_da(ref="1") immig_cat2(ref="(1) Not an immigrant") rural_pccf(ref="N")

preg_smoking(ref="0") preg_drug_exposure(ref="0") conception_type(ref="Spontaneous") tri1_prenatal_visit(ref="1") preex_dm(ref="0")

gest_dm(ref="0") preex_hyper(ref="0") gest_hyper(ref="0") previous_cd(ref="0") induction(ref="0") augmentation_ind(ref="0")

presentation_type(ref="Cephalic") duration_stage2(ref="<60 minutes") mode_of_delivery(ref="(01) SVD") b_weight_g_cat1(ref="(2) 3000-3999");

model pph_severe = ancestry age_group parity_bin plurality primary_language2 maternal_bmi_cat material_resources_q_da immig_cat2 rural_pccf

preg_smoking preg_drug_exposure conception_type tri1_prenatal_visit preex_dm gest_dm preex_hyper gest_hyper previous_cd induction

augmentation_ind presentation_type duration_stage2 mode_of_delivery b_weight_g_cat1 / dist=poisson link=log;

repeated subject=ikn;

ods output Genmod.GEEEmpPEst = multi_rr_severe;

**run**;

**data** multi_rr_severe;

retain Parm Level1 adj_est adj_lower adj_upper ProbZ;

set multi_rr_severe;

if _n_ = **1** or _n_ = **5** or _n_ = **11** or _n_ = **14** or _n_ = **16** or _n_ = **19** or _n_ = **26** or _n_ = **32** or _n_ = **37** or _n_ = **40**

or _n_ = **43** or _n_ = **46** or _n_ = **49** or _n_ = **52** or _n_ = **54** or _n_ = **56** or _n_ = **58** or _n_ = **60** or _n_ = **62** or _n_ = **64**

or _n_ = **66** or _n_ = **70** or _n_ = **76** or _n_ = **85** or _n_ = **90** then delete;

adj_est = round(exp(Estimate), **0.0001**);

adj_lower = round(exp(LowerCL), **0.0001**);

adj_upper = round(exp(UpperCL), **0.0001**);

keep Parm Level1 adj_est adj_lower adj_upper ProbZ;

rename ProbZ = adj_pval;

**run**;

**data** multi_rr_severe;

set multi_rr_severe;

if Parm = 'age_group' then do;

if Level1 = '<20' then place = _n_ - **4**;

else place = _n_ + **1**;

end;

else if Parm = 'maternal_bmi_cat' then do;

if Level1 = '<18.5' then place = _n_ - **3**;

else if Level1 in ('>=40' 'Unknown') then place = _n_;

else place = _n_ + **1**;

end;

else if Parm = 'duration_stage2' then do;

if Level1 = '60-119 minutes' then place = _n_ - **2**;

else if Level1 in ('120-179 minutes' '180-239 minutes') then place = _n_ + **1**;

else place = _n_;

end;

else place = _n_;

**run**;

**proc** **sort** data=multi_rr_severe out=multi_rr_severe(drop=place:); by place; **run**;

**data** tables.model_results_severe;

merge uni_rr_severe multi_rr_severe;

drop place: Parm;

**run**;

/* Get overall Ns of outcomes: */

**data** modeldata;

set redpph.preg_outcomes;

if ancestry = 'Unknown/missing' then delete;

keep pregnancy_id_enc ancestry ikn born_dad_pph pph_severe;

**run**;

**data** modelvars;

set redpph.preg_cohort;

if parity_cat = '0' then parity_bin = **0**;

else if parity_cat = 'Unknown' then parity_bin = **9**;

else parity_bin = **1**;

if primary_language in ('English' 'French') then primary_language = 'English/French';

if smoking_atbirth = **1** or smoking_firstvisit = **1** then preg_smoking = **1**;

else if smoking_atbirth = **9** and smoking_firstvisit = **9** then preg_smoking = **9**;

else preg_smoking = **0**;

if preg_hyper not in ('N/A' 'None') then gest_hyper = **1**;

else gest_hyper = **0**;

if immig_birth_datediff_cat = '' then immig_birth_datediff_cat = '(6) Not an immigrant';

if duration_stage2 = 'Missing' then duration_stage2 = 'No second stage';

if substr(mode_of_delivery, **1**, **3**) in ('(09' '(10') then mode_of_delivery = '(09) US/PM CD';

keep pregnancy_id_enc age_group parity_bin plurality primary_language maternal_bmi_cat age_labourforce_q_da material_resources_q_da

households_dwellings_q_da immig_cat2 immig_birth_datediff_cat rural_pccf preg_smoking preg_drug_exposure conception_type

tri1_prenatal_visit preex_dm preex_hyper previous_cd gest_dm gest_hyper placenta_previa placenta_accspec placental_abruption

duration_stage2 mode_of_delivery gestage_cat induction augmentation_ind presentation_type episiotomy b_weight_g_cat1 b_headcirc_cm_cat1;

**run**;

**proc** **sql**;

create table modeldata as

select * from modeldata as a left join modelvars as b

on a.pregnancy_id_enc = b.pregnancy_id_enc;

**quit**;

**data** nulli parous;

set modeldata;

if parity_bin = **1** then output parous;

else if parity_bin = **0** then output nulli;

**run**;

%***bivartab***(

data = modeldata,

byvar = born_dad_pph,

numdq = miss_only,

catvar = ancestry age_group parity_bin plurality primary_language maternal_bmi_cat age_labourforce_q_da material_resources_q_da

households_dwellings_q_da immig_cat2 immig_birth_datediff_cat rural_pccf preg_smoking preg_drug_exposure conception_type

tri1_prenatal_visit preex_dm gest_dm preex_hyper gest_hyper previous_cd placenta_previa placenta_accspec placental_abruption

induction augmentation_ind episiotomy presentation_type duration_stage2 mode_of_delivery gestage_cat b_weight_g_cat1

b_headcirc_cm_cat1,

order = ancestry age_group parity_bin plurality primary_language maternal_bmi_cat age_labourforce_q_da material_resources_q_da

households_dwellings_q_da immig_cat2 immig_birth_datediff_cat rural_pccf preg_smoking preg_drug_exposure conception_type

tri1_prenatal_visit preex_dm gest_dm preex_hyper gest_hyper previous_cd placenta_previa placenta_accspec placental_abruption

induction augmentation_ind episiotomy presentation_type duration_stage2 mode_of_delivery gestage_cat b_weight_g_cat1

b_headcirc_cm_cat1,

total = F,

small = T,

stdiff = F,

trend = F,

colpct = F,

out = pph_freqs);

**data** pph_freqs;

set pph_freqs;

if VarValue = '' then delete;

keep VarLabel VarValue born_dad_pph_1;

**run**;

%***bivartab***(

data = nulli,

byvar = born_dad_pph,

numdq = miss_only,

catvar = ancestry age_group plurality primary_language maternal_bmi_cat age_labourforce_q_da material_resources_q_da

households_dwellings_q_da immig_cat2 immig_birth_datediff_cat rural_pccf preg_smoking preg_drug_exposure conception_type

tri1_prenatal_visit preex_dm gest_dm preex_hyper gest_hyper previous_cd placenta_previa placenta_accspec placental_abruption

induction augmentation_ind episiotomy presentation_type duration_stage2 mode_of_delivery gestage_cat b_weight_g_cat1

b_headcirc_cm_cat1,

order = ancestry age_group plurality primary_language maternal_bmi_cat age_labourforce_q_da material_resources_q_da

households_dwellings_q_da immig_cat2 immig_birth_datediff_cat rural_pccf preg_smoking preg_drug_exposure conception_type

tri1_prenatal_visit preex_dm gest_dm preex_hyper gest_hyper previous_cd placenta_previa placenta_accspec placental_abruption

induction augmentation_ind episiotomy presentation_type duration_stage2 mode_of_delivery gestage_cat b_weight_g_cat1

b_headcirc_cm_cat1,

total = F,

small = T,

stdiff = F,

trend = F,

colpct = F,

out = nulli_pph_freqs);

**data** nulli_pph_freqs;

set nulli_pph_freqs;

if VarValue = '' then delete;

keep VarLabel VarValue born_dad_pph_1;

**run**;

%***bivartab***(

data = parous,

byvar = born_dad_pph,

numdq = miss_only,

catvar = ancestry age_group plurality primary_language maternal_bmi_cat age_labourforce_q_da material_resources_q_da

households_dwellings_q_da immig_cat2 immig_birth_datediff_cat rural_pccf preg_smoking preg_drug_exposure conception_type

tri1_prenatal_visit preex_dm gest_dm preex_hyper gest_hyper previous_cd placenta_previa placenta_accspec placental_abruption

induction augmentation_ind episiotomy presentation_type duration_stage2 mode_of_delivery gestage_cat b_weight_g_cat1

b_headcirc_cm_cat1,

order = ancestry age_group plurality primary_language maternal_bmi_cat age_labourforce_q_da material_resources_q_da

households_dwellings_q_da immig_cat2 immig_birth_datediff_cat rural_pccf preg_smoking preg_drug_exposure conception_type

tri1_prenatal_visit preex_dm gest_dm preex_hyper gest_hyper previous_cd placenta_previa placenta_accspec placental_abruption

induction augmentation_ind episiotomy presentation_type duration_stage2 mode_of_delivery gestage_cat b_weight_g_cat1

b_headcirc_cm_cat1,

total = F,

small = T,

stdiff = F,

trend = F,

colpct = F,

out = parous_pph_freqs);

**data** parous_pph_freqs;

set parous_pph_freqs;

if VarValue = '' then delete;

keep VarLabel VarValue born_dad_pph_1;

**run**;

%***bivartab***(

data = modeldata,

byvar = pph_severe,

numdq = miss_only,

catvar = ancestry age_group parity_bin plurality primary_language maternal_bmi_cat material_resources_q_da immig_cat2 rural_pccf preg_smoking

preg_drug_exposure conception_type tri1_prenatal_visit preex_dm gest_dm preex_hyper gest_hyper previous_cd induction

augmentation_ind presentation_type duration_stage2 mode_of_delivery b_weight_g_cat1,

order = ancestry age_group parity_bin plurality primary_language maternal_bmi_cat material_resources_q_da immig_cat2 rural_pccf preg_smoking

preg_drug_exposure conception_type tri1_prenatal_visit preex_dm gest_dm preex_hyper gest_hyper previous_cd induction

augmentation_ind presentation_type duration_stage2 mode_of_delivery b_weight_g_cat1,

total = F,

small = T,

stdiff = F,

trend = F,

pdigits = **2**,

colpct = F,

out = severe_pph_freqs);

**data** severe_pph_freqs;

set severe_pph_freqs;

if VarValue = '' then delete;

keep VarLabel VarValue pph_severe_1;

**run**;

/*%macro languages();

%do i = 1 %to 12;

data code&i.;

set WORK.CIC_LANGUAGES_0000;

code = left(put(code&i., 8.));

keep continent WorldRegion Country language code;

run;

%end;

data redpph.asian_languages;

set code1-code12;

if code = '.' then delete;

if length(code) = 1 then code = cats('00', code);

else if length(code) = 2 then code = cats('0', code);

run;

proc datasets; delete code1-code12; run;

%mend;

%languages();*/

**proc** **sql**;

create table cohort as

select a.pregnancy_id_enc, a.ikn, a.b_bdate, a.landing_date, a.language_cat, a.nat_language, a.parity_cat, a.ancestry,

b.born_dad_pph, b.pph_severe,

c.continent, c.WorldRegion, c.Country, c.language

from redpph.preg_cohort as a left join redpph.preg_outcomes as b on a.pregnancy_id_enc = b.pregnancy_id_enc

left join redpph.asian_languages as c on a.nat_language = c.code;

**quit**;

**data** asian_languages;

set cohort;

if Continent = 'Asia';

**run**;

%***bivartab***(

data = asian_languages,

byvar = ancestry,

catvar = Language,

numdq = F,

total = F,

small = T,

trend = F,

stdiff = F,

colpct = T,

mdigits = **2**,

medigits = **0**,

pdigits = **2**,

label = T,

out = asian_language_tab,

binary = F);

**data** asian_language_tab;

set asian_language_tab;

if VarValue = '' then delete;

keep VarLabel VarValue Asian Black Caucasian Other Unknown_missing;

**run**;

**data** asian_cohort;

set cohort;

if ancestry = 'Asian';

**run**;

**data** southeast_asian;

length sea_langcat $ **10.**;

set asian_cohort;

if parity_cat = '0' then parous = **0**; else parous = **1**;

if nat_language in ('309' '8788' '306' '8785');

if nat_language in ('309' '8788') then sea_langcat = 'Tagalog';

else sea_langcat = 'Vietnamese';

**run**;

**proc** **freq** data=southeast_asian; tables sea_langcat / out=se_lang_freqs; **run**;

**proc** **sort** data=asian_cohort; by pregnancy_id_enc; **run**;

**data** asian_cohort;

length WorldRegion2 Country2 Language2 $ **20.**;

set asian_cohort;

if parity_cat = '0' then parous = **0**; else parous = **1**;

if landing_date = **.** then do;

WorldRegion2 = '1 Not an immigrant';

Country2 = '1 Not an immigrant';

Language2 = '1 Not an immigrant';

end;

else if landing_date ^= **.** and Continent = '' then do;

WorldRegion2 = '2 Non-Asian language';

Country2 = '2 Non-Asian language';

Language2 = '2 Non-Asian language';

end;

else do;

WorldRegion2 = WorldRegion;

Country2 = Country;

Language2 = Language;

end;

**run**;

**proc** **freq** data=asian_cohort;

tables WorldRegion2 / out=region_freqs;

tables Country2 / out=country_freqs;

**run**;

**proc** **sql**;

create table region_denom as

select WorldRegion2, count(*) as denom

from asian_cohort group by WorldRegion2;

create table region_pph as

select WorldRegion2, sum(born_dad_pph) as num_pph, sum(pph_severe) as num_sevpph

from asian_cohort group by WorldRegion2;

create table region_pph as

select * from region_pph as a inner join region_denom as b

on a.WorldRegion2 = b.WorldRegion2;

create table region_parity_denom as

select WorldRegion2, parous, count(*) as denom

from asian_cohort group by WorldRegion2, parous;

create table region_parity_pph as

select WorldRegion2, parous, sum(born_dad_pph) as num_pph, sum(pph_severe) as num_sevpph

from asian_cohort group by WorldRegion2, parous;

create table region_parity_pph as

select * from region_parity_pph as a inner join region_parity_denom as b

on a.WorldRegion2 = b.WorldRegion2 and a.parous = b.parous;

create table se_denom as

select sea_langcat, count(*) as denom

from southeast_asian group by sea_langcat;

create table se_pph as

select sea_langcat, sum(born_dad_pph) as num_pph, sum(pph_severe) as num_sevpph

from southeast_asian group by sea_langcat;

create table se_pph as

select * from se_pph as a inner join se_denom as b

on a.sea_langcat = b.sea_langcat;

create table se_parity_denom as

select sea_langcat, parous, count(*) as denom

from southeast_asian group by sea_langcat, parous;

create table se_parity_pph as

select sea_langcat, parous, sum(born_dad_pph) as num_pph, sum(pph_severe) as num_sevpph

from southeast_asian group by sea_langcat, parous;

create table se_parity_pph as

select * from se_parity_pph as a inner join se_parity_denom as b

on a.sea_langcat = b.sea_langcat and a.parous = b.parous;

**quit**;

%***cirates***(

data = region_pph,

num = num_pph,

denom = denom,

out = region_pph_rates,

per = **100**,

method = poisson);

%***cirates***(

data = region_pph,

num = num_sevpph,

denom = denom,

out = region_sevpph_rates,

per = **100**,

method = poisson);

%***cirates***(

data = region_parity_pph,

num = num_pph,

denom = denom,

out = region_parity_pph_rates,

per = **100**,

method = poisson);

%***cirates***(

data = region_parity_pph,

num = num_sevpph,

denom = denom,

out = region_parity_sevpph_rates,

per = **100**,

method = poisson);

%***cirates***(

data = se_pph,

num = num_pph,

denom = denom,

out = se_pph_rates,

per = **100**,

method = poisson);

%***cirates***(

data = se_pph,

num = num_sevpph,

denom = denom,

out = se_sevpph_rates,

per = **100**,

method = poisson);

%***cirates***(

data = se_parity_pph,

num = num_pph,

denom = denom,

out = se_parity_pph_rates,

per = **100**,

method = poisson);

%***cirates***(

data = se_parity_pph,

num = num_sevpph,

denom = denom,

out = se_parity_sevpph_rates,

per = **100**,

method = poisson);

**data** region_pph_rates;

set region_pph_rates;

pph_rate = catx(' (', round(CrudeRate, **0.01**), cats(round(lower, **0.01**), '-', round(upper, **0.01**), ')'));

keep WorldRegion2 pph_rate;

**run**;

**data** region_sevpph_rates;

set region_sevpph_rates;

sevpph_rate = catx(' (', round(CrudeRate, **0.01**), cats(round(lower, **0.01**), '-', round(upper, **0.01**), ')'));

keep WorldRegion2 sevpph_rate;

**run**;

**data** pph_parous(rename=(pph_rate=parous_pph_rate)) pph_nulli(rename=(pph_rate=nulli_pph_rate));

set region_parity_pph_rates;

pph_rate = catx(' (', round(CrudeRate, **0.01**), cats(round(lower, **0.01**), '-', round(upper, **0.01**), ')'));

if parous = **1** then output pph_parous;

else output pph_nulli;

keep WorldRegion2 pph_rate;

**run**;

**data** sevpph_parous(rename=(sevpph_rate=parous_sevpph_rate)) sevpph_nulli(rename=(sevpph_rate=nulli_sevpph_rate));

set region_parity_sevpph_rates;

sevpph_rate = catx(' (', round(CrudeRate, **0.01**), cats(round(lower, **0.01**), '-', round(upper, **0.01**), ')'));

if parous = **1** then output sevpph_parous;

else output sevpph_nulli;

keep WorldRegion2 sevpph_rate;

**run**;

**data** rates; merge region_pph_rates pph_nulli pph_parous region_sevpph_rates sevpph_nulli sevpph_parous; by WorldRegion2; **run**;

**data** se_pph_rates;

set se_pph_rates;

pph_rate = catx(' (', round(CrudeRate, **0.01**), cats(round(lower, **0.01**), '-', round(upper, **0.01**), ')'));

keep sea_langcat pph_rate;

**run**;

**data** se_sevpph_rates;

set se_sevpph_rates;

sevpph_rate = catx(' (', round(CrudeRate, **0.01**), cats(round(lower, **0.01**), '-', round(upper, **0.01**), ')'));

keep sea_langcat sevpph_rate;

**run**;

**data** pph_parous(rename=(pph_rate=parous_pph_rate)) pph_nulli(rename=(pph_rate=nulli_pph_rate));

set se_parity_pph_rates;

pph_rate = catx(' (', round(CrudeRate, **0.01**), cats(round(lower, **0.01**), '-', round(upper, **0.01**), ')'));

if parous = **1** then output pph_parous;

else output pph_nulli;

keep sea_langcat pph_rate;

**run**;

**data** sevpph_parous(rename=(sevpph_rate=parous_sevpph_rate)) sevpph_nulli(rename=(sevpph_rate=nulli_sevpph_rate));

set se_parity_sevpph_rates;

sevpph_rate = catx(' (', round(CrudeRate, **0.01**), cats(round(lower, **0.01**), '-', round(upper, **0.01**), ')'));

if parous = **1** then output sevpph_parous;

else output sevpph_nulli;

keep sea_langcat sevpph_rate;

**run**;

**data** rates; merge se_pph_rates pph_nulli pph_parous se_sevpph_rates sevpph_nulli sevpph_parous; by sea_langcat; **run**;

/* New baseline table: */

**data** asian_cohort;

length ancestry_asian $ **24.**;

set cohort;

if ancestry in ('Asian' 'Caucasian');

if ancestry = 'Caucasian' then ancestry_asian = 'White';

else if ancestry = 'Asian' then do;

if landing_date = **.** then ancestry_asian = 'Asian (not an immigrant)';

else do;

if Continent = '' then do;

if nat_language in ('001' '10206370' '8571') then ancestry_asian = 'Asian (English)';

else if nat_language in ('002' '10206371' '8572') then ancestry_asian = 'Asian (French)';

else ancestry_asian = 'Asian (other)';

end;

else do;

if WorldRegion = 'Central Asia' then ancestry_asian = 'Asian (Central Asia)';

else if WorldRegion = 'East Asia' then ancestry_asian = 'Asian (East Asia)';

else if WorldRegion = 'South Asia' then ancestry_asian = 'Asian (South Asia)';

else if WorldRegion = 'Southeast Asia' then ancestry_asian = 'Asian (Southeast Asia)';

else if WorldRegion = 'West Asia' then ancestry_asian = 'Asian (West Asia)';

end;

end;

end;

**run**;

/* Format baseline characteristics table: */

**data** preg_cohort;

set redpph.preg_cohort;

array exist[**15**] plurality smoking_firstvisit smoking_atbirth preg_drug_exposure tri1_prenatal_visit preex_dm gest_dm preex_hyper

previous_cd placenta_previa placenta_accspec placental_abruption induction augmentation_ind ob_trauma;

array new[**15**] $ plural smoking1 smoking2 drugexpose tri1visit dm1 dm2 hyper1 prevcd previa accspec abruption induct augment obtrauma;

do i = **1** to **15**;

if exist[i] = **1** then new[i] = '(1) Y'; else if exist[i] = **0** then new[i] = '(2) N';

end;

drop i;

**run**;

**proc** **sql**;

create table asian_cohort as

select a.pregnancy_id_enc, a.ancestry_asian, a.born_dad_pph, a.pph_severe, b.*

from asian_cohort as a inner join preg_cohort as b

on a.pregnancy_id_enc = b.pregnancy_id_enc;

**quit**;

%***bivartab***(

data = asian_cohort,

byvar = ancestry_asian,

numdq = miss_only,

catvar = age_group parity_cat plural primary_language maternal_bmi_cat age_labourforce_q_da material_resources_q_da

households_dwellings_q_da immig_cat2 immig_birth_datediff_cat rural_pccf smoking1 smoking2 drugexpose conception_type

tri1visit dm1 dm2 hyper1 preg_induced_hyper prevcd previa accspec abruption induct augment episiotomy presentation_type

labour_stage2_cat mode_of_delivery obtrauma gestage_cat b_weight_g_cat1 b_weight_g_cat2 b_weight_g_cat3 b_weight_g_cat4

b_headcirc_cm_cat1 b_headcirc_cm_cat2 b_headcirc_cm_cat3 b_headcirc_cm_cat4,

numvar = immig_birth_datediff prenatal_visits_combo lbr_time_sec_stg_total_minutes,

order = age_group parity_cat plural primary_language maternal_bmi_cat age_labourforce_q_da material_resources_q_da

households_dwellings_q_da immig_cat2 immig_birth_datediff immig_birth_datediff_cat rural_pccf smoking1 smoking2

drugexpose conception_type tri1visit prenatal_visits_combo dm1 dm2 hyper1 preg_induced_hyper prevcd previa accspec

abruption induct augment episiotomy presentation_type lbr_time_sec_stg_total_minutes labour_stage2_cat mode_of_delivery

obtrauma gestage_cat b_weight_g_cat1 b_weight_g_cat2 b_weight_g_cat3 b_weight_g_cat4 b_headcirc_cm_cat1 b_headcirc_cm_cat2

b_headcirc_cm_cat3 b_headcirc_cm_cat4,

total = T,

small = T,

stdiff = F,

trend = F,

out = table1_asian);

**data** table1_asian;

retain varlabel varvalue White Asian__not_an_immigrant_ Asian__English_ Asian__French_ Asian__other_ Asian__Central_Asia_ Asian__East_Asia_ Asian__South_Asia_ Asian__Southeast_Asia_ Asian__West_Asia_;

set table1_asian;

if VarValue = '' then delete;

keep varlabel varvalue White Asian__not_an_immigrant_ Asian__English_ Asian__French_ Asian__other_ Asian__Central_Asia_ Asian__East_Asia_ Asian__South_Asia_ Asian__Southeast_Asia_ Asian__West_Asia_;

**run**;

**data** table1_asian;

set table1_asian;

if _n_ = **51** or _n_ = **57** or _n_ = **78** or _n_ = **113** then delete;

**run**;

**data** table1_asian;

set table1_asian;

if varvalue = '<20 - n (%)' then place = _n_ - **5**;

else if varvalue in ('20-24 - n (%)' '25-29 - n (%)' '30-34 - n (%)' '35-39 - n (%)' '40+ - n (%)') then place = _n_ + **1**;

else if varvalue = '<18.5 - n (%)' then place = _n_ - **4**;

else if varvalue in ('18.5-24.9 - n (%)' '25-29.9 - n (%)' '30-34.9 - n (%)' '35-39.9 - n (%)') then place = _n_ + **1**;

else if varvalue = 'Breech - n (%)' then place = _n_ + **1**;

else if varvalue = 'Cephalic - n (%)' then place = _n_ - **1**;

else if varvalue = '<60 minutes - n (%)' then place = _n_ - **3**;

else if varvalue = '60-119 minutes - n (%)' then place = _n_ - **1**;

else if varvalue in ('120-179 minutes - n (%)' '180-239 minutes - n (%)') then place = _n_ + **2**;

else if varvalue = 'Spontaneous - n (%)' then place = _n_ - **1**;

else if varvalue = 'Assisted - n (%)' then place = _n_ + **1**;

else if varlabel = 'preg_induced_hyper' then place = _n_ + **5**;

else if varvalue = 'Preeclampsia - n (%)' then place = _n_ - **5**;

else if varvalue = 'HELLP - n (%)' then place = _n_ - **1**;

else if varvalue = 'Gestational - n (%)' then place = _n_ + **1**;

else place = _n_;

**run**;

**proc** **sort** data=table1_asian out=tables.table1_asian(drop=place); by place; **run**;

**data** asian_cohort;

set asian_cohort;

if parity_cat = '0' then parity_bin = **0**;

else if parity_cat = 'Unknown' then parity_bin = **9**;

else parity_bin = **1**;

if primary_language in ('English' 'French') then primary_language = 'English/French';

if smoking_atbirth = **1** or smoking_firstvisit = **1** then preg_smoking = **1**;

else if smoking_atbirth = **9** and smoking_firstvisit = **9** then preg_smoking = **9**;

else preg_smoking = **0**;

if preg_hyper not in ('N/A' 'None') then gest_hyper = **1**;

else gest_hyper = **0**;

if immig_birth_datediff_cat = '' then immig_birth_datediff_cat = '(6) Not an immigrant';

if duration_stage2 = 'Missing' then duration_stage2 = 'No second stage';

if substr(mode_of_delivery, **1**, **3**) in ('(09' '(10') then mode_of_delivery = '(09) US/PM CD';

**run**;

%***bivartab***(

data = asian_cohort,

byvar = born_dad_pph,

numdq = miss_only,

catvar = ancestry_asian age_group parity_bin plurality primary_language maternal_bmi_cat age_labourforce_q_da material_resources_q_da

households_dwellings_q_da immig_cat2 immig_birth_datediff_cat rural_pccf preg_smoking preg_drug_exposure conception_type

tri1_prenatal_visit preex_dm gest_dm preex_hyper gest_hyper previous_cd placenta_previa placenta_accspec placental_abruption induction

augmentation_ind episiotomy presentation_type duration_stage2 mode_of_delivery gestage_cat b_weight_g_cat1 b_headcirc_cm_cat1,

order = ancestry_asian age_group parity_bin plurality primary_language maternal_bmi_cat age_labourforce_q_da material_resources_q_da

households_dwellings_q_da immig_cat2 immig_birth_datediff_cat rural_pccf preg_smoking preg_drug_exposure conception_type

tri1_prenatal_visit preex_dm gest_dm preex_hyper gest_hyper previous_cd placenta_previa placenta_accspec placental_abruption induction

augmentation_ind episiotomy presentation_type duration_stage2 mode_of_delivery gestage_cat b_weight_g_cat1 b_headcirc_cm_cat1,

total = T,

small = T,

stdiff = F,

trend = F,

colpct = F,

out = outcome_freqs);

**data** outcome_freqs;

set outcome_freqs;

if VarValue in ('' 'Sample Size') then delete;

keep VarLabel VarValue born_dad_pph_1;

**run**;

**data** outcome_freqs;

set outcome_freqs;

if VarValue = 'White - n (%)' then place = _n_ - **8**;

else if VarValue = 'Asian (not an immigrant) - n (%)' then place = _n_ - **5**;

else if VarValue = 'Asian (other) - n (%)' then place = _n_ - **4**;

else if VarValue in ('Asian (Central Asia) - n (%)' 'Asian (East Asia) - n (%)') then place = _n_ + **4**;

else if VarValue in ('Asian (South Asia) - n (%)' 'Asian (Southeast Asia) - n (%)' 'Asian (West Asia) - n (%)') then place = _n_ + **3**;

else if VarValue = '<20 - n (%)' then place = _n_ - **5**;

else if VarValue in ('20-24 - n (%)' '25-29 - n (%)' '30-34 - n (%)' '35-39 - n (%)' '40+ - n (%)') then place = _n_ + **1**;

else if VarValue = '<18.5 - n (%)' then place = _n_ - **4**;

else if VarValue in ('18.5-24.9 - n (%)' '25-29.9 - n (%)' '30-34.9 - n (%)' '35-39.9 - n (%)') then place = _n_ + **1**;

else if VarValue = 'Spontaneous - n (%)' then place = _n_ - **1**;

else if VarValue = 'Assisted - n (%)' then place = _n_ + **1**;

else if VarValue = '60-119 minutes - n (%)' then place = _n_ - **1**;

else if VarValue = '<60 minutes - n (%)' then place = _n_ - **3**;

else if VarValue in ('120-179 minutes - n (%)' '180-239 minutes - n (%)') then place = _n_ + **2**;

else place = _n_;

/* Change plurality position: */

if _n_ = **20** then place = _n_ - **1**;

if _n_ = **19** then place = _n_ + **1**;

**run**;

**proc** **sort** data=outcome_freqs out=outcome_freqs(drop=place); by place; **run**;

**%macro** univariate(data=, var=, ref=, num=);

proc genmod data=&data.;

class ikn &var.(ref="&ref") / param=ref;

model born_dad_pph = &var. / dist=poisson link=log;

repeated subject=ikn;

ods output Genmod.GEEEmpPEst = &var._uni_rr;

run;

data &var._uni_rr;

retain Variable Level1 est lower upper probz place1;

length Variable $ **24.**;

set &var._uni_rr;

Variable = "&var";

if Parm = 'Intercept' then delete;

est = round(exp(Estimate), **0.0001**);

lower = round(exp(LowerCL), **0.0001**);

upper = round(exp(UpperCL), **0.0001**);

place1 = &num.;

keep Variable Level1 est lower upper probz place1;

rename probz = pval;

run;

data &var._uni_rr;

set &var._uni_rr;

place2 = _n_;

run;

**%mend**;

%***univariate***(data=asian_cohort, var=ancestry_asian, ref=White, num=**1**);

**data** ancestry_asian_uni_rr;

set ancestry_asian_uni_rr;

if Level1 = 'Asian (not an immigrant)' then place2 = **1**;

else if Level1 = 'Asian (English)' then place2 = **2**;

else if Level1 = 'Asian (other)' then place2 = **3**;

else if Level1 = 'Asian (Central Asia)' then place2 = **4**;

else if Level1 = 'Asian (East Asia)' then place2 = **5**;

else if Level1 = 'Asian (South Asia)' then place2 = **6**;

else if Level1 = 'Asian (Southeast Asia)' then place2 = **7**;

else if Level1 = 'Asian (West Asia)' then place2 = **8**;

**run**;

%***univariate***(data=asian_cohort, var=age_group, ref=**20**-**24**, num=**2**);

**data** age_group_uni_rr;

set age_group_uni_rr;

if Level1 = '<20' then place2 = **1**;

else place2 = _n_ + **1**;

**run**;

%***univariate***(data=asian_cohort, var=parity_bin, ref=**1**, num=**3**);

%***univariate***(data=asian_cohort, var=plurality, ref=**0**, num=**4**);

%***univariate***(data=asian_cohort, var=primary_language, ref=English/French, num=**5**);

%***univariate***(data=asian_cohort, var=maternal_bmi_cat, ref=**18.5**-**24.9**, num=**6**);

**data** maternal_bmi_cat_uni_rr;

set maternal_bmi_cat_uni_rr;

if Level1 = '<18.5' then place2 = **1**;

else if Level1 not in ('>=40' 'Unknown') then place2 = _n_ + **1**;

else place2 = _n_;

**run**;

%***univariate***(data=asian_cohort, var=age_labourforce_q_da, ref=**1**, num=**7**);

%***univariate***(data=asian_cohort, var=material_resources_q_da, ref=**1**, num=**8**);

%***univariate***(data=asian_cohort, var=households_dwellings_q_da, ref=**1**, num=**9**);

%***univariate***(data=asian_cohort, var=immig_cat2, ref=(**1**) Not an immigrant, num=**10**);

%***univariate***(data=asian_cohort, var=immig_birth_datediff_cat, ref=(**5**) >**10** years, num=**11**);

%***univariate***(data=asian_cohort, var=rural_pccf, ref=N, num=**12**);

%***univariate***(data=asian_cohort, var=preg_smoking, ref=**0**, num=**13**);

%***univariate***(data=asian_cohort, var=preg_drug_exposure, ref=**0**, num=**14**);

%***univariate***(data=asian_cohort, var=conception_type, ref=Spontaneous, num=**15**);

%***univariate***(data=asian_cohort, var=tri1_prenatal_visit, ref=**1**, num=**16**);

%***univariate***(data=asian_cohort, var=preex_dm, ref=**0**, num=**17**);

%***univariate***(data=asian_cohort, var=gest_dm, ref=**0**, num=**18**);

%***univariate***(data=asian_cohort, var=preex_hyper, ref=**0**, num=**19**);

%***univariate***(data=asian_cohort, var=gest_hyper, ref=**0**, num=**20**);

%***univariate***(data=asian_cohort, var=previous_cd, ref=**0**, num=**21**);

%***univariate***(data=asian_cohort, var=placenta_previa, ref=**0**, num=**22**);

%***univariate***(data=asian_cohort, var=placenta_accspec, ref=**0**, num=**23**);

%***univariate***(data=asian_cohort, var=placental_abruption, ref=**0**, num=**24**);

%***univariate***(data=asian_cohort, var=induction, ref=**0**, num=**25**);

%***univariate***(data=asian_cohort, var=augmentation_ind, ref=**0**, num=**26**);

%***univariate***(data=asian_cohort, var=episiotomy, ref=None, num=**27**);

%***univariate***(data=asian_cohort, var=presentation_type, ref=Cephalic, num=**28**);

%***univariate***(data=asian_cohort, var=duration_stage2, ref=<**60** minutes, num=**29**);

**data** duration_stage2_uni_rr;

set duration_stage2_uni_rr;

if Level1 = '60-119 minutes' then place2 = **1**;

else if Level1 in ('120-179 minutes' '180-239 minutes') then place2 = _n_ + **1**;

else place2 = _n_;

**run**;

%***univariate***(data=asian_cohort, var=mode_of_delivery, ref=(**01**) SVD, num=**30**);

%***univariate***(data=asian_cohort, var=gestage_cat, ref=(**2**) Term, num=**31**);

%***univariate***(data=asian_cohort, var=b_weight_g_cat1, ref=(**2**) **3000**-**3999**, num=**32**);

%***univariate***(data=asian_cohort, var=b_headcirc_cm_cat1, ref=(**2**) **33**-**34**, num=**33**);

**data** uni_rr;

retain Variable Level1;

length Level1 $ **30.**;

set ancestry_asian_uni_rr age_group_uni_rr parity_bin_uni_rr plurality_uni_rr primary_language_uni_rr maternal_bmi_cat_uni_rr age_labourforce_q_da_uni_rr

material_resources_q_da_uni_rr households_dwellings_q_da_uni_rr immig_cat2_uni_rr immig_birth_datediff_cat_uni_rr rural_pccf_uni_rr preg_smoking_uni_rr

preg_drug_exposure_uni_rr conception_type_uni_rr tri1_prenatal_visit_uni_rr preex_dm_uni_rr gest_dm_uni_rr preex_hyper_uni_rr gest_hyper_uni_rr

previous_cd_uni_rr placenta_previa_uni_rr placenta_accspec_uni_rr placental_abruption_uni_rr induction_uni_rr augmentation_ind_uni_rr episiotomy_uni_rr

presentation_type_uni_rr duration_stage2_uni_rr mode_of_delivery_uni_rr gestage_cat_uni_rr b_weight_g_cat1_uni_rr b_headcirc_cm_cat1_uni_rr;

rename est=uni_est lower=uni_lower upper=uni_upper pval=uni_pval;

**run**;

**proc** **sort** data=uni_rr out=uni_rr(drop=place:); by place1 place2; **run**;

**proc** **genmod** data=asian_cohort;

class ikn ancestry_asian(ref="White") age_group(ref="20-24") parity_bin(ref="1") plurality(ref="0") primary_language(ref="English/French")

maternal_bmi_cat(ref="18.5-24.9") age_labourforce_q_da(ref="1") material_resources_q_da(ref="1") households_dwellings_q_da(ref="1")

immig_cat2(ref="(1) Not an immigrant") immig_birth_datediff_cat(ref="(5) >10 years") rural_pccf(ref="N") preg_smoking(ref="0")

preg_drug_exposure(ref="0") conception_type(ref="Spontaneous") tri1_prenatal_visit(ref="1") preex_dm(ref="0") gest_dm(ref="0")

preex_hyper(ref="0") gest_hyper(ref="0") previous_cd(ref="0") placenta_previa(ref="0") placenta_accspec(ref="0")

placental_abruption(ref="0") induction(ref="0") augmentation_ind(ref="0") episiotomy(ref="None") presentation_type(ref="Cephalic")

duration_stage2(ref="<60 minutes") mode_of_delivery(ref="(01) SVD") gestage_cat(ref="(2) Term") b_weight_g_cat1(ref="(2) 3000-3999")

b_headcirc_cm_cat1(ref="(2) 33-34");

model born_dad_pph = ancestry_asian age_group parity_bin plurality primary_language maternal_bmi_cat age_labourforce_q_da material_resources_q_da

households_dwellings_q_da immig_cat2 immig_birth_datediff_cat rural_pccf preg_smoking preg_drug_exposure conception_type

tri1_prenatal_visit preex_dm gest_dm preex_hyper gest_hyper previous_cd placenta_previa placenta_accspec placental_abruption induction

augmentation_ind episiotomy presentation_type duration_stage2 mode_of_delivery gestage_cat b_weight_g_cat1 b_headcirc_cm_cat1

/ dist=poisson link=log;

repeated subject=ikn;

ods output Genmod.GEEEmpPEst = multi_rr;

**run**;

**data** multi_rr;

retain Parm Level1 adj_est adj_lower adj_upper ProbZ;

set multi_rr;

if _n_ = **1** then delete;

adj_est = round(exp(Estimate), **0.0001**);

adj_lower = round(exp(LowerCL), **0.0001**);

adj_upper = round(exp(UpperCL), **0.0001**);

keep Parm Level1 adj_est adj_lower adj_upper ProbZ;

rename ProbZ = adj_pval;

**run**;

**data** multi_rr2;

set multi_rr;

if Parm = 'ancestry_asian' then do;

if Level1 = 'White' then place = _n_ - **8**;

else if Level1 = 'Asian (not an immigrant)' then place = _n_ - **5**;

else if Level1 = 'Asian (English)' then place = _n_;

else if Level1 = 'Asian (other)' then place = _n_ - **4**;

else if Level1 in ('Asian (Central Asia)' 'Asian (East Asia)') then place = _n_ + **4**;

else if Level1 in ('Asian (South Asia)' 'Asian (Southeast Asia)' 'Asian (West Asia)') then place = _n_ + **3**;

end;

else if Parm = 'age_group' then do;

if Level1 in ('<20' '20-24') then place = _n_ - **4**;

else place = _n_ + **2**;

end;

else if Parm in ('parity_bin' 'tri1_prenatal_visit') then do;

if Level1 = '1' then place = _n_ - **1**;

else if Level1 = '9' then place = _n_ + **1**;

else place = _n_;

end;

else if Parm in ('preg_smoking' 'preg_drug_exposure' 'tri1_prenatal_visit') then do;

if Level1 = '0' then place = _n_ - **2**;

else place = _n_ + **1**;

end;

else if Parm = 'primary_language' then do;

if Level1 = 'English/French' then place = _n_ - **2**;

else place = _n_ + **1**;

end;

else if Parm = 'maternal_bmi_cat' then do;

if Level1 = '<18.5' then place = _n_ - **3**;

else if Level1 = '18.5-24.9' then place = _n_ - **5**;

else if Level1 in ('>=40' 'Unknown') then place = _n_ + **1**;

else place = _n_ + **2**;

end;

else if Parm in ('age_labourforce_q_da' 'material_resources_q' 'households_dwellings') then do;

if Level1 = '1' then place = _n_ - **5**;

else place = _n_ + **1**;

end;

else if Parm = 'immig_cat2' then do;

if Level1 = '(1) Not an immigrant' then place = _n_ - **4**;

else place = _n_ + **1**;

end;

else if Parm = 'immig_birth_datediff' then do;

if Level1 = '(5) >10 years' then place = _n_ - **1**;

else if Level1 = '(6) Not an immigrant' then place = _n_ + **1**;

else place = _n_;

end;

else if Parm = 'rural_pccf' then do;

if Level1 = 'N' then place = _n_ - **2**;

else place = _n_ + **1**;

end;

else if Parm = 'conception_type' then do;

if Level1 = 'Spontaneous' then place = _n_ - **2**;

else place = _n_ + **1**;

end;

else if Parm in ('preex_dm' 'gest_dm' 'preex_hyper' 'gest_hyper' 'previous_cd' 'placenta_previa' 'placenta_accspec' 'placental_abruption' 'induction' 'augmentation_ind') then do;

if Level1 = '0' then place = _n_ - **1**;

else place = _n_ + **1**;

end;

else if Parm = 'episiotomy' then do;

if Level1 = 'None' then place = _n_ - **1**;

else if Level1 = 'Unknown' then place = _n_ + **1**;

else place = _n_;

end;

else if Parm = 'presentation_type' then do;

if Level1 = 'Cephalic' then place = _n_ - **2**;

else if Level1 = 'Breech' then place = _n_;

else place = _n_ + **1**;

end;

else if Parm = 'duration_stage2' then do;

if Level1 = '60-119 minutes' then place = _n_ - ;

else if Level1 = '<60 minutes' then place = _n_ - **5**;

else if Level1 in ('120-179 minutes' '180-239 minutes') then place = _n_ + **2**;

else place = _n_ + **1**;

end;

else if Parm = 'mode_of_delivery' then do;

if Level1 = '(01) SVD' then place = _n_ - **8**;

else place = _n_ + **1**;

end;

else if Parm = 'gestage_cat' then do;

if Level1 = '(2) Term' then place = _n_ - **1**;

else if Level1 = '(3) Post-term' then place = _n_ + **1**;

else place = _n_;

end;

else if Parm = 'b_weight_g_cat1' then do;

if Level1 = '(2) 3000-3999' then place = _n_ - **3**;

else if Level1 = '(1) <3000' then place = _n_;

else place = _n_ + **1**;

end;

else if Parm = 'b_headcirc_cm_cat1' then do;

if Level1 = '(2) 33-34' then place = _n_ - **3**;

else if Level1 = '(1) <33' then place = _n_;

else place = _n_ + **1**;

end;

else place = _n_;

**run**;

**proc** **sort** data=multi_rr2 out=multi_rr2(drop=place); by place; **run**;

/* Severe PPH: */

%***bivartab***(

data = asian_cohort,

byvar = pph_severe,

numdq = miss_only,

catvar = ancestry_asian age_group parity_bin plurality primary_language maternal_bmi_cat material_resources_q_da immig_cat2 rural_pccf

preg_smoking preg_drug_exposure conception_type tri1_prenatal_visit preex_dm gest_dm preex_hyper gest_hyper previous_cd induction

augmentation_ind presentation_type duration_stage2 mode_of_delivery b_weight_g_cat1,

order = ancestry_asian age_group parity_bin plurality primary_language maternal_bmi_cat material_resources_q_da immig_cat2 rural_pccf

preg_smoking preg_drug_exposure conception_type tri1_prenatal_visit preex_dm gest_dm preex_hyper gest_hyper previous_cd induction

augmentation_ind presentation_type duration_stage2 mode_of_delivery b_weight_g_cat1,

total = T,

small = F,

stdiff = F,

trend = F,

colpct = F,

out = outcome_freqs);

**data** outcome_freqs;

set outcome_freqs;

if VarValue in ('' 'Sample Size') then delete;

keep VarLabel VarValue pph_severe_1;

**run**;

**data** outcome_freqs;

set outcome_freqs;

if VarValue = 'White - n (%)' then place = _n_ - **8**;

else if VarValue = 'Asian (not an immigrant) - n (%)' then place = _n_ - **5**;

else if VarValue = 'Asian (other) - n (%)' then place = _n_ - **4**;

else if VarValue in ('Asian (Central Asia) - n (%)' 'Asian (East Asia) - n (%)') then place = _n_ + **4**;

else if VarValue in ('Asian (South Asia) - n (%)' 'Asian (Southeast Asia) - n (%)' 'Asian (West Asia) - n (%)') then place = _n_ + **3**;

else if VarValue = '<20 - n (%)' then place = _n_ - **5**;

else if VarValue in ('20-24 - n (%)' '25-29 - n (%)' '30-34 - n (%)' '35-39 - n (%)' '40+ - n (%)') then place = _n_ + **1**;

else if VarValue = '<18.5 - n (%)' then place = _n_ - **4**;

else if VarValue in ('18.5-24.9 - n (%)' '25-29.9 - n (%)' '30-34.9 - n (%)' '35-39.9 - n (%)') then place = _n_ + **1**;

else if VarValue = 'Spontaneous - n (%)' then place = _n_ - **1**;

else if VarValue = 'Assisted - n (%)' then place = _n_ + **1**;

else if VarValue = '60-119 minutes - n (%)' then place = _n_ - **1**;

else if VarValue = '<60 minutes - n (%)' then place = _n_ - **3**;

else if VarValue in ('120-179 minutes - n (%)' '180-239 minutes - n (%)') then place = _n_ + **2**;

else place = _n_;

/* Change plurality position: */

if _n_ = **20** then place = _n_ - **1**;

if _n_ = **19** then place = _n_ + **1**;

**run**;

**proc** **sort** data=outcome_freqs out=outcome_freqs(drop=place); by place; **run**;

**%macro** univariate(data=, var=, ref=, num=);

proc genmod data=&data.;

class ikn &var.(ref="&ref") / param=ref;

model pph_severe = &var. / dist=poisson link=log;

repeated subject=ikn;

ods output Genmod.GEEEmpPEst = &var._uni_rr;

run;

data &var._uni_rr;

retain Variable Level1 est lower upper probz place1;

length Variable $ **24.**;

set &var._uni_rr;

Variable = "&var";

if Parm = 'Intercept' then delete;

est = round(exp(Estimate), **0.0001**);

lower = round(exp(LowerCL), **0.0001**);

upper = round(exp(UpperCL), **0.0001**);

place1 = &num.;

keep Variable Level1 est lower upper probz place1;

rename probz = pval;

run;

data &var._uni_rr;

set &var._uni_rr;

place2 = _n_;

run;

**%mend**;

%***univariate***(data=asian_cohort, var=ancestry_asian, ref=White, num=**1**);

**data** ancestry_asian_uni_rr;

set ancestry_asian_uni_rr;

if Level1 = 'Asian (not an immigrant)' then place2 = **1**;

else if Level1 = 'Asian (English)' then place2 = **2**;

else if Level1 = 'Asian (other)' then place2 = **3**;

else if Level1 = 'Asian (Central Asia)' then place2 = **4**;

else if Level1 = 'Asian (East Asia)' then place2 = **5**;

else if Level1 = 'Asian (South Asia)' then place2 = **6**;

else if Level1 = 'Asian (Southeast Asia)' then place2 = **7**;

else if Level1 = 'Asian (West Asia)' then place2 = **8**;

**run**;

%***univariate***(data=asian_cohort, var=age_group, ref=**20**-**24**, num=**2**);

**data** age_group_uni_rr;

set age_group_uni_rr;

if Level1 = '<20' then place2 = **1**;

else place2 = _n_ + **1**;

**run**;

%***univariate***(data=asian_cohort, var=parity_bin, ref=**1**, num=**3**);

%***univariate***(data=asian_cohort, var=plurality, ref=**0**, num=**4**);

%***univariate***(data=asian_cohort, var=primary_language, ref=English/French, num=**5**);

%***univariate***(data=asian_cohort, var=maternal_bmi_cat, ref=**18.5**-**24.9**, num=**6**);

**data** maternal_bmi_cat_uni_rr;

set maternal_bmi_cat_uni_rr;

if Level1 = '<18.5' then place2 = **1**;

else if Level1 not in ('>=40' 'Unknown') then place2 = _n_ + **1**;

else place2 = _n_;

**run**;

%***univariate***(data=asian_cohort, var=material_resources_q_da, ref=**1**, num=**7**);

%***univariate***(data=asian_cohort, var=immig_cat2, ref=(**1**) Not an immigrant, num=**8**);

%***univariate***(data=asian_cohort, var=rural_pccf, ref=N, num=**9**);

%***univariate***(data=asian_cohort, var=preg_smoking, ref=**0**, num=**10**);

%***univariate***(data=asian_cohort, var=preg_drug_exposure, ref=**0**, num=**11**);

%***univariate***(data=asian_cohort, var=conception_type, ref=Spontaneous, num=**12**);

%***univariate***(data=asian_cohort, var=tri1_prenatal_visit, ref=**1**, num=**13**);

%***univariate***(data=asian_cohort, var=preex_dm, ref=**0**, num=**14**);

%***univariate***(data=asian_cohort, var=gest_dm, ref=**0**, num=**15**);

%***univariate***(data=asian_cohort, var=preex_hyper, ref=**0**, num=**16**);

%***univariate***(data=asian_cohort, var=gest_hyper, ref=**0**, num=**17**);

%***univariate***(data=asian_cohort, var=previous_cd, ref=**0**, num=**18**);

%***univariate***(data=asian_cohort, var=induction, ref=**0**, num=**19**);

%***univariate***(data=asian_cohort, var=augmentation_ind, ref=**0**, num=**20**);

%***univariate***(data=asian_cohort, var=presentation_type, ref=Cephalic, num=**21**);

%***univariate***(data=asian_cohort, var=duration_stage2, ref=<**60** minutes, num=**22**);

**data** duration_stage2_uni_rr;

set duration_stage2_uni_rr;

if Level1 = '60-119 minutes' then place2 = **1**;

else if Level1 in ('120-179 minutes' '180-239 minutes') then place2 = _n_ + **1**;

else place2 = _n_;

**run**;

%***univariate***(data=asian_cohort, var=mode_of_delivery, ref=(**01**) SVD, num=**23**);

%***univariate***(data=asian_cohort, var=b_weight_g_cat1, ref=(**2**) **3000**-**3999**, num=**24**);

**data** uni_rr;

retain Variable Level1;

length Level1 $ **30.**;

set ancestry_asian_uni_rr age_group_uni_rr parity_bin_uni_rr plurality_uni_rr primary_language_uni_rr maternal_bmi_cat_uni_rr

material_resources_q_da_uni_rr immig_cat2_uni_rr rural_pccf_uni_rr preg_smoking_uni_rr

preg_drug_exposure_uni_rr conception_type_uni_rr tri1_prenatal_visit_uni_rr preex_dm_uni_rr gest_dm_uni_rr preex_hyper_uni_rr gest_hyper_uni_rr

previous_cd_uni_rr induction_uni_rr augmentation_ind_uni_rr

presentation_type_uni_rr duration_stage2_uni_rr mode_of_delivery_uni_rr b_weight_g_cat1_uni_rr;

rename est=uni_est lower=uni_lower upper=uni_upper pval=uni_pval;

**run**;

**proc** **sort** data=uni_rr out=uni_rr(drop=place:); by place1 place2; **run**;

**proc** **genmod** data=asian_cohort;

class ikn ancestry_asian(ref="White") age_group(ref="20-24") parity_bin(ref="1") plurality(ref="0") primary_language(ref="English/French")

maternal_bmi_cat(ref="18.5-24.9") material_resources_q_da(ref="1") immig_cat2(ref="(1) Not an immigrant") rural_pccf(ref="N")

preg_smoking(ref="0") preg_drug_exposure(ref="0") conception_type(ref="Spontaneous") tri1_prenatal_visit(ref="1") preex_dm(ref="0")

gest_dm(ref="0") preex_hyper(ref="0") gest_hyper(ref="0") previous_cd(ref="0") induction(ref="0") augmentation_ind(ref="0")

presentation_type(ref="Cephalic") duration_stage2(ref="<60 minutes") mode_of_delivery(ref="(01) SVD") b_weight_g_cat1(ref="(2) 3000-3999");

model pph_severe = ancestry_asian age_group parity_bin plurality primary_language maternal_bmi_cat material_resources_q_da immig_cat2 rural_pccf

preg_smoking preg_drug_exposure conception_type tri1_prenatal_visit preex_dm gest_dm preex_hyper gest_hyper previous_cd induction

augmentation_ind presentation_type duration_stage2 mode_of_delivery b_weight_g_cat1 / dist=poisson link=log;

repeated subject=ikn;

ods output Genmod.GEEEmpPEst = multi_rr;

**run**;

**data** multi_rr;

retain Parm Level1 adj_est adj_lower adj_upper ProbZ;

set multi_rr;

if _n_ = **1** then delete;

adj_est = round(exp(Estimate), **0.0001**);

adj_lower = round(exp(LowerCL), **0.0001**);

adj_upper = round(exp(UpperCL), **0.0001**);

keep Parm Level1 adj_est adj_lower adj_upper ProbZ;

rename ProbZ = adj_pval;

**run**;

**data** multi_rr2;

set multi_rr;

if Parm = 'ancestry_asian' then do;

if Level1 = 'White' then place = _n_ - **8**;

else if Level1 = 'Asian (not an immigrant)' then place = _n_ - **5**;

else if Level1 = 'Asian (English)' then place = _n_;

else if Level1 = 'Asian (other)' then place = _n_ - **4**;

else if Level1 in ('Asian (Central Asia)' 'Asian (East Asia)') then place = _n_ + **4**;

else if Level1 in ('Asian (South Asia)' 'Asian (Southeast Asia)' 'Asian (West Asia)') then place = _n_ + **3**;

end;

else if Parm = 'age_group' then do;

if Level1 in ('<20' '20-24') then place = _n_ - **4**;

else place = _n_ + **2**;

end;

else if Parm in ('parity_bin' 'tri1_prenatal_visit') then do;

if Level1 = '1' then place = _n_ - **1**;

else if Level1 = '9' then place = _n_ + **1**;

else place = _n_;

end;

else if Parm in ('preg_smoking' 'preg_drug_exposure') then do;

if Level1 = '0' then place = _n_ - **2**;

else place = _n_ + **1**;

end;

else if Parm = 'primary_language' then do;

if Level1 = 'English/French' then place = _n_ - **2**;

else place = _n_ + **1**;

end;

else if Parm = 'maternal_bmi_cat' then do;

if Level1 = '<18.5' then place = _n_ - **3**;

else if Level1 = '18.5-24.9' then place = _n_ - **5**;

else if Level1 in ('>=40' 'Unknown') then place = _n_ + **1**;

else place = _n_ + **2**;

end;

else if Parm = 'material_resources_q' then do;

if Level1 = '1' then place = _n_ - **5**;

else place = _n_ + **1**;

end;

else if Parm = 'immig_cat2' then do;

if Level1 = '(1) Not an immigrant' then place = _n_ - **4**;

else place = _n_ + **1**;

end;

else if Parm = 'rural_pccf' then do;

if Level1 = 'N' then place = _n_ - **2**;

else place = _n_ + **1**;

end;

else if Parm = 'conception_type' then do;

if Level1 = 'Spontaneous' then place = _n_ - **2**;

else place = _n_ + **1**;

end;

else if Parm in ('preex_dm' 'gest_dm' 'preex_hyper' 'gest_hyper' 'previous_cd' 'induction' 'augmentation_ind') then do;

if Level1 = '0' then place = _n_ - **1**;

else place = _n_ + **1**;

end;

else if Parm = 'presentation_type' then do;

if Level1 = 'Cephalic' then place = _n_ - **2**;

else if Level1 = 'Breech' then place = _n_;

else place = _n_ + **1**;

end;

else if Parm = 'duration_stage2' then do;

if Level1 = '60-119 minutes' then place = _n_ - **1**;

else if Level1 = '<60 minutes' then place = _n_ - **5**;

else if Level1 in ('120-179 minutes' '180-239 minutes') then place = _n_ + **2**;

else place = _n_ + **1**;

end;

else if Parm = 'mode_of_delivery' then do;

if Level1 = '(01) SVD' then place = _n_ - **8**;

else place = _n_ + **1**;

end;

else if Parm = 'b_weight_g_cat1' then do;

if Level1 = '(2) 3000-3999' then place = _n_ - **3**;

else if Level1 = '(1) <3000' then place = _n_;

else place = _n_ + **1**;

end;

else place = _n_;

**run**;

**proc** **sort** data=multi_rr2 out=multi_rr2(drop=place); by place; **run**;

/* Get data for models: */

**data** modeldata;

set redpph.preg_outcomes;

if ancestry = 'Unknown/missing' then delete;

keep pregnancy_id_enc ancestry ikn born_dad_pph pph_severe;

**run**;

**data** modelvars;

set redpph.preg_cohort;

if parity_cat = '0' then parity_bin = **0**;

else if parity_cat = 'Unknown' then parity_bin = **9**;

else parity_bin = **1**;

*if primary_language in ('English' 'French') then primary_language = 'English/French';

if smoking_atbirth = **1** or smoking_firstvisit = **1** then preg_smoking = **1**;

else if smoking_atbirth = **9** and smoking_firstvisit = **9** then preg_smoking = **9**;

else preg_smoking = **0**;

if preg_hyper not in ('N/A' 'None') then gest_hyper = **1**;

else gest_hyper = **0**;

if immig_birth_datediff_cat = '' then immig_birth_datediff_cat = '(6) Not an immigrant';

if duration_stage2 = 'Missing' then duration_stage2 = 'No second stage';

if substr(mode_of_delivery, **1**, **3**) in ('(09' '(10') then mode_of_delivery = '(09) US/PM CD';

keep pregnancy_id_enc age_group parity_bin plurality primary_language maternal_bmi_cat age_labourforce_q_da material_resources_q_da

households_dwellings_q_da immig_cat2 immig_birth_datediff_cat rural_pccf preg_smoking preg_drug_exposure conception_type landing_date

tri1_prenatal_visit preex_dm preex_hyper previous_cd gest_dm gest_hyper placenta_previa placenta_accspec placental_abruption b_bdate

duration_stage2 mode_of_delivery gestage_cat induction augmentation_ind presentation_type episiotomy b_weight_g_cat1 b_headcirc_cm_cat1;

**run**;

**proc** **sql**;

create table modeldata as

select * from modeldata as a left join modelvars as b

on a.pregnancy_id_enc = b.pregnancy_id_enc;

**quit**;

**data** cohort;

set modeldata;

if ancestry in ('Asian' 'Caucasian');

**run**;

**proc** **sql**;

create table totals as

select ancestry, immig_cat2, count(*) as total

from cohort

group by ancestry, immig_cat2;

create table pph_freqs as

select ancestry, immig_cat2, sum(born_dad_pph) as pph, sum(pph_severe) as severe_pph

from cohort

group by ancestry, immig_cat2;

create table pph_freqs as

select a.*, b.total, a.pph/b.total as prop_pph,

**1.9599***(sqrt((((calculated prop_pph)*(**1**-(calculated prop_pph)))/b.total))) as pph_cipm,

(calculated prop_pph) - (calculated pph_cipm) as prop_pph_lower,

(calculated prop_pph) + (calculated pph_cipm) as prop_pph_upper,

a.severe_pph/b.total as prop_sevpph,

**1.9599***(sqrt((((calculated prop_sevpph)*(**1**-(calculated prop_sevpph)))/b.total))) as sev_pph_cipm,

(calculated prop_sevpph) - (calculated sev_pph_cipm) as prop_sev_pph_lower,

(calculated prop_sevpph) + (calculated sev_pph_cipm) as prop_sev_pph_upper

from pph_freqs as a inner join totals as b

on a.ancestry = b.ancestry and a.immig_cat2 = b.immig_cat2;

**quit**;

**data** tables.pph_freqs_asian;

retain ancestry immig_cat2 total pph pc_pph pc_pph_lower pc_pph_upper severe_pph pc_sevpph pc_sevpph_lower pc_sevpph_upper;

set pph_freqs;

pc_pph = prop_pph***100**;

pc_pph_lower = prop_pph_lower***100**;

pc_pph_upper = prop_pph_upper***100**;

pc_sevpph = prop_sevpph***100**;

pc_sevpph_lower = prop_sev_pph_lower***100**;

pc_sevpph_upper = prop_sev_pph_upper***100**;

keep ancestry immig_cat2 total pph pc_pph pc_pph_lower pc_pph_upper severe_pph pc_sevpph pc_sevpph_lower pc_sevpph_upper;

**run**;

**proc** **sort** data=tables.pph_freqs_asian; by descending ancestry immig_cat2; **run**;

/* Pull birth dates and update ages: */

%***getdemo***(

data = cohort,

out = cohort,

getsex = F,

getdeath = F,

getdolc = F,

getbdate = T,

agedate = b_bdate);

**data** redpph.preg_cohort_asian;

length age_group_updated $5.;

set cohort;

if age < **20** then age_group_updated = '<20';

else if **20** <= age <= **24** then age_group_updated = '20-24';

else if **25** <= age <= **29** then age_group_updated = '25-29';

else if **30** <= age <= **34** then age_group_updated = '30-34';

else if **35** <= age <= **39** then age_group_updated = '35-39';

else age_group_updated = '40+';

if landing_date ^= **.** then do;

if landing_date >= b_bdate then duration_in_canada = **0**;

else duration_in_canada = (b_bdate - landing_date)/**365.25**;

end;

else if landing_date = **.** then duration_in_canada = (b_bdate - bdate)/**365.25**;

drop age_group;

rename age_group_updated = age_group;

**run**;

**proc** **sql**;

create table preg_cohort_asian as

select a.pregnancy_id_enc, a.nat_language, c.Continent, c.WorldRegion

from redpph.preg_cohort as a left join redpph.asian_languages as c on a.nat_language = c.code;

create table redpph.preg_cohort_asian as

select * from redpph.preg_cohort_asian as a left join preg_cohort_asian as b

on a.pregnancy_id_enc = b.pregnancy_id_enc;

**quit**;

**data** redpph.preg_cohort_asian;

length ancestry_asian $ **24.**;

set redpph.preg_cohort_asian;

if ancestry = 'Caucasian' then ancestry_asian = 'White';

else do;

if landing_date = **.** then ancestry_asian = 'Asian (not an immigrant)';

else do;

if Continent = '' then do;

if nat_language in ('001' '10206370' '8571') then ancestry_asian = 'Asian (English)';

else if nat_language in ('002' '10206371' '8572') then ancestry_asian = 'Asian (French)';

else ancestry_asian = 'Asian (other)';

end;

else do;

if WorldRegion = 'Central Asia' then ancestry_asian = 'Asian (Central Asia)';

else if WorldRegion = 'East Asia' then ancestry_asian = 'Asian (East Asia)';

else if WorldRegion = 'South Asia' then ancestry_asian = 'Asian (South Asia)';

else if WorldRegion = 'Southeast Asia' then ancestry_asian = 'Asian (Southeast Asia)';

else if WorldRegion = 'West Asia' then ancestry_asian = 'Asian (West Asia)';

end;

end;

end;

**run**;

**data** redpph.preg_cohort_asian;

length ancestry_asian3 $ **25.**;

set redpph.preg_cohort_asian;

if ancestry_asian = 'Asian (French)' then ancestry_asian3 = 'Asian (other)';

else ancestry_asian3 = ancestry_asian;

**run**;

**proc** **sql**;

create table redpph.preg_cohort_asian as

select a.*, /*b.smoking_firstvisit, b.smoking_atbirth, b.ob_trauma, b.parity_cat, b.preg_induced_hyper, b.labour_stage2_cat, b.b_weight_g_cat2,

b.b_weight_g_cat3, b.b_weight_g_cat4, b.b_headcirc_cm_cat2, b.b_headcirc_cm_cat3, b.b_headcirc_cm_cat4, b.immig_birth_datediff,

b.prenatal_visits_combo, b.lbr_time_sec_stg_total_minutes,*/ b.cs_stage

from redpph.preg_cohort_asian as a inner join redpph.preg_cohort as b

on a.pregnancy_id_enc = b.pregnancy_id_enc;

**quit**;

**data** redpph.preg_cohort_asian;

set redpph.preg_cohort_asian;

array exist[**15**] plurality smoking_firstvisit smoking_atbirth preg_drug_exposure tri1_prenatal_visit preex_dm gest_dm preex_hyper

previous_cd placenta_previa placenta_accspec placental_abruption induction augmentation_ind ob_trauma;

array new[**15**] plural smoking1 smoking2 drugexpose tri1visit dm1 dm2 hyper1 prevcd previa accspec abruption induct augment obtrauma;

do i = **1** to **15**;

if exist[i] = **1** then new[i] = **1**; else new[i] = **0**;

end;

drop i;

**run**;

**data** redpph.preg_cohort_asian;

length mat_bmi2 $ **13.**;

length ctype2 $ **15.**;

set redpph.preg_cohort_asian;

if maternal_bmi_cat = '<18.5' then mat_bmi2 = '(1) <18.5';

else if maternal_bmi_cat = '18.5-24.9' then mat_bmi2 = '(2) 18.5-24.9';

else if maternal_bmi_cat = '25-29.9' then mat_bmi2 = '(3) 25-29.9';

else if maternal_bmi_cat = '30-34.9' then mat_bmi2 = '(4) 30-34.9';

else if maternal_bmi_cat = '35-39.9' then mat_bmi2 = '(5) 35-39.9';

else if maternal_bmi_cat = '>=40' then mat_bmi2 = '(6) >=40';

else mat_bmi2 = '(7) Unknown';

if conception_type = 'Assisted' then ctype2 = '(2) Assisted';

else if conception_type = 'Spontaneous' then ctype2 = '(1) Spontaneous';

else if conception_type = 'Unknown' then ctype2 = '(3) Unknown';

if preg_induced_hyper = 'Preeclampsia' then pi_hyper = '(1) Preeclampsia';

else if preg_induced_hyper = 'Eclampsia' then pi_hyper = '(2) Eclampsia';

else if preg_induced_hyper = 'HELLP' then pi_hyper = '(3) HELLP';

else if preg_induced_hyper = 'Gestational' then pi_hyper = '(4) Gestational';

else if preg_induced_hyper = '' then pi_hyper = '(5) Missing';

else if preg_induced_hyper = 'None' then pi_hyper = '(6) None';

if episiotomy = 'None' then episiotomy = 'ZNone';

if presentation_type = 'Breech' then presentation_type = '2Breech';

else if presentation_type = 'Cephalic' then presentation_type = '1Cephalic';

if mode_of_delivery = '(09) US/PM CD' then do;

if cs_stage = 'Perimortem' then mode_of_delivery = '(10) PM CD';

else mode_of_delivery = '(09) US CD';

end;

else if mode_of_delivery = '(06) First stage CD' then mode_of_delivery = '(07) First stage CD';

else if mode_of_delivery = '(07) Second stage CD' then mode_of_delivery = '(08) Second stage CD';

else if mode_of_delivery = '(08) CD without labour' then mode_of_delivery = '(06) CD without labour';

**run**;

/* Manuscript Table 1: */

%***bivartab***(

data = redpph.preg_cohort_asian,

byvar = ancestry,

numdq = miss_only,

catvar = age_group parity_cat plural mat_bmi2 age_labourforce_q_da material_resources_q_da households_dwellings_q_da immig_cat2

immig_birth_datediff_cat rural_pccf smoking1 smoking2 drugexpose ctype2 tri1visit dm1 dm2 hyper1 pi_hyper prevcd previa

accspec abruption induct augment episiotomy presentation_type mode_of_delivery obtrauma gestage_cat b_weight_g_cat1 b_headcirc_cm_cat1,

numvar = duration_in_canada immig_birth_datediff prenatal_visits_combo,

order = age_group parity_cat plural mat_bmi2 age_labourforce_q_da material_resources_q_da households_dwellings_q_da immig_cat2

duration_in_canada immig_birth_datediff immig_birth_datediff_cat rural_pccf smoking1 smoking2 drugexpose ctype2 tri1visit

prenatal_visits_combo dm1 dm2 hyper1 pi_hyper prevcd previa accspec abruption induct augment episiotomy presentation_type

mode_of_delivery obtrauma gestage_cat b_weight_g_cat1 b_headcirc_cm_cat1,

total = T,

small = T,

stdiff = T,

trend = F,

binary = T,

out = table1_asian);

**data** table1_asian;

retain VarLabel VarValue Caucasian Asian;

set table1_asian;

if VarValue in ('' 'Missing Data (%)') then delete;

drop Variable;

keep VarLabel VarValue Caucasian Asian Std_Diff;

**run**;

**data** table1_asian;

set table1_asian;

if _n_ = **53** or _n_ = **56** or _n_ = **74** or _n_ = **84** then delete;

**run**;

%***bivartab***(

data = redpph.preg_cohort_asian,

byvar = ancestry_asian3,

numdq = miss_only,

catvar = age_group parity_cat plural mat_bmi2 age_labourforce_q_da material_resources_q_da households_dwellings_q_da immig_cat2

immig_birth_datediff_cat rural_pccf smoking1 smoking2 drugexpose ctype2 tri1visit dm1 dm2 hyper1 pi_hyper prevcd previa

accspec abruption induct augment episiotomy presentation_type mode_of_delivery obtrauma gestage_cat b_weight_g_cat1 b_headcirc_cm_cat1,

numvar = duration_in_canada immig_birth_datediff prenatal_visits_combo,

order = age_group parity_cat plural mat_bmi2 age_labourforce_q_da material_resources_q_da households_dwellings_q_da immig_cat2

duration_in_canada immig_birth_datediff immig_birth_datediff_cat rural_pccf smoking1 smoking2 drugexpose ctype2 tri1visit

prenatal_visits_combo dm1 dm2 hyper1 pi_hyper prevcd previa accspec abruption induct augment episiotomy presentation_type

mode_of_delivery obtrauma gestage_cat b_weight_g_cat1 b_headcirc_cm_cat1,

total = T,

small = T,

stdiff = F,

trend = F,

binary = T,

out = table1_asian3);

**data** table1_asian3;

retain VarLabel VarValue Asian__not_an_immigrant_ Asian__Central_Asia_ Asian__East_Asia_

Asian__South_Asia_ Asian__Southeast_Asia_ Asian__West_Asia_ Asian__English_ Asian__other_;

set table1_asian3;

if VarValue in ('' 'Missing Data (%)') then delete;

drop Variable;

keep VarLabel VarValue Asian__not_an_immigrant_ Asian__English_ Asian__other_ Asian__Central_Asia_

Asian__East_Asia_ Asian__South_Asia_ Asian__Southeast_Asia_ Asian__West_Asia_;

**run**;

**data** table1_asian3;

set table1_asian3;

if _n_ = **53** or _n_ = **56** or _n_ = **74** or _n_ = **84** then delete;

**run**;

**data** manuscript_table1; merge table1_asian table1_asian3; **run**;

/* Figure 2 crude rates of PPH and severe PPH: */

**data** redpph.preg_cohort_asian;

length ancestry_asian2 $ **25.**;

set redpph.preg_cohort_asian;

if ancestry_asian in ('Asian (English)' 'Asian (French)' 'Asian (other)') then ancestry_asian2 = 'Non-Asian language';

else ancestry_asian2 = ancestry_asian;

**run**;

**proc** **sql**;

create table pph_ns as

select ancestry, count(*) as total, sum(born_dad_pph) as num_pph, sum(pph_severe) as num_sev_pph

from redpph.preg_cohort_asian

group by ancestry;

create table pph_parity_ns as

select parity_bin, ancestry, count(*) as total, sum(born_dad_pph) as num_pph, sum(pph_severe) as num_sev_pph

from redpph.preg_cohort_asian

group by parity_bin, ancestry;

create table pph_lang_ns as

select ancestry_asian2, count(*) as total, sum(born_dad_pph) as num_pph, sum(pph_severe) as num_sev_pph

from redpph.preg_cohort_asian

group by ancestry_asian2;

create table pph_lang_parity_ns as

select parity_bin, ancestry_asian2, count(*) as total, sum(born_dad_pph) as num_pph, sum(pph_severe) as num_sev_pph

from redpph.preg_cohort_asian

group by parity_bin, ancestry_asian2;

**quit**;

**data** pph_parity_ns; set pph_parity_ns; if parity_bin = **9** then delete; **run**;

**data** pph_lang_ns; set pph_lang_ns; if ancestry_asian2 = 'White' then delete; **run**;

**data** pph_lang_parity_ns; set pph_lang_parity_ns; if parity_bin = **9** or ancestry_asian2 = 'White' then delete; **run**;

%***cirates***(data = pph_ns, num = num_pph, denom = total, out = pph_rates, per = **100**, method = poisson);

%***cirates***(data = pph_parity_ns, num = num_pph, denom = total, out = pph_parity_rates, per = **100**, method = poisson);

%***cirates***(data = pph_lang_ns, num = num_pph, denom = total, out = pph_lang_rates, per = **100**, method = poisson);

%***cirates***(data = pph_lang_parity_ns, num = num_pph, denom = total, out = pph_lang_parity_rates, per = **100**, method = poisson);

%***cirates***(data = pph_ns, num = num_sev_pph, denom = total, out = spph_rates, per = **100**, method = poisson);

%***cirates***(data = pph_parity_ns, num = num_sev_pph, denom = total, out = spph_parity_rates, per = **100**, method = poisson);

%***cirates***(data = pph_lang_ns, num = num_sev_pph, denom = total, out = spph_lang_rates, per = **100**, method = poisson);

%***cirates***(data = pph_lang_parity_ns, num = num_sev_pph, denom = total, out = spph_lang_parity_rates, per = **100**, method = poisson);

**data** pph_rates; set pph_rates; keep ancestry total CrudeRate lower upper; **run**;

**data** pph_nulli(rename=(CrudeRate=nulli_rate lower=nulli_lower upper=nulli_upper)) pph_parous(rename=(CrudeRate=parous_rate lower=parous_lower upper=parous_upper));

set pph_parity_rates;

if parity_bin = **0** then output pph_nulli;

else output pph_parous;

drop total num_pph num_sev_pph parity_bin;

**run**;

**data** pph_rates_tab1; merge pph_rates pph_nulli pph_parous; by ancestry; **run**;

**data** pph_lang_rates; set pph_lang_rates; keep ancestry_asian2 total CrudeRate lower upper; **run**;

**data** pph_lang_nulli(rename=(CrudeRate=nulli_rate lower=nulli_lower upper=nulli_upper))

pph_lang_parous(rename=(CrudeRate=parous_rate lower=parous_lower upper=parous_upper));

set pph_lang_parity_rates;

if parity_bin = **0** then output pph_lang_nulli;

else output pph_lang_parous;

drop total num_pph num_sev_pph parity_bin;

**run**;

**data** pph_rates_tab2; merge pph_lang_rates pph_lang_nulli pph_lang_parous; by ancestry_asian2; rename ancestry_asian2 = ancestry; **run**;

**data** spph_rates; set spph_rates; keep ancestry CrudeRate lower upper; rename CrudeRate = sevRate lower = sevlower upper = sevupper; **run**;

**data** spph_nulli(rename=(CrudeRate=nulli_sevrate lower=nulli_sevlower upper=nulli_sevupper))

spph_parous(rename=(CrudeRate=parous_sevrate lower=parous_sevlower upper=parous_sevupper));

set spph_parity_rates;

if parity_bin = **0** then output spph_nulli;

else output spph_parous;

drop total num_pph num_sev_pph parity_bin;

**run**;

**data** spph_rates_tab1; merge spph_rates spph_nulli spph_parous; by ancestry; **run**;

**data** pph_rates_tab1; merge pph_rates_tab1 spph_rates_tab1; by ancestry; **run**;

**proc** **sort** data=pph_rates_tab1; by descending ancestry; **run**;

**data** spph_lang_rates; set spph_lang_rates; keep ancestry_asian2 CrudeRate lower upper; rename CrudeRate = sevRate lower = sevlower upper=sevupper; **run**;

**data** spph_lang_nulli(rename=(CrudeRate=nulli_sevrate lower=nulli_sevlower upper=nulli_sevupper))

spph_lang_parous(rename=(CrudeRate=parous_sevrate lower=parous_sevlower upper=parous_sevupper));

set spph_lang_parity_rates;

if parity_bin = **0** then output spph_lang_nulli;

else output spph_lang_parous;

drop total num_pph num_sev_pph parity_bin;

**run**;

**data** spph_rates_tab2; merge spph_lang_rates spph_lang_nulli spph_lang_parous; by ancestry_asian2; rename ancestry_asian2 = ancestry; **run**;

**data** pph_rates_tab2; merge pph_rates_tab2 spph_rates_tab2; by ancestry; **run**;

**data** pph_rates_tab2;

set pph_rates_tab2;

if ancestry = 'Asian (not an immigrant)' then sort = **1**;

else if ancestry = 'Non-Asian language' then sort = **2**;

else if ancestry = 'Asian (Central Asia)' then sort = **3**;

else if ancestry = 'Asian (East Asia)' then sort = **4**;

else if ancestry = 'Asian (South Asia)' then sort = **5**;

else if ancestry = 'Asian (Southeast Asia)' then sort = **6**;

else sort = **7**;

**run**;

**proc** **sort** data=pph_rates_tab2 out=pph_rates_tab2(drop=sort); by sort; **run**;

**data** pph_rates_tab;

length ancestry $ **25.**;

set pph_rates_tab1 pph_rates_tab2;

**run**;

/* Additional table - crude rates of PPH and severe PPH by ancestry and immigrant status: */

**proc** **freq** data=redpph.preg_cohort_asian;

tables immig_cat2 ancestry;

**run**;

**data** redpph.preg_cohort_asian;

set redpph.preg_cohort_asian;

if immig_cat2 = '(1) Not an immigrant' then immig_ind = **0**; else immig_ind = **1**;

**run**;

**proc** **sql**;

create table immig_pph_freqs as

select ancestry, immig_ind, count(*) as total, sum(born_dad_pph) as num_pph, sum(pph_severe) as num_sev_pph

from redpph.preg_cohort_asian

group by ancestry, immig_ind;

create table immig_pph_freqs2 as

select ancestry, immig_cat2, count(*) as total, sum(born_dad_pph) as num_pph, sum(pph_severe) as num_sev_pph

from redpph.preg_cohort_asian

group by ancestry, immig_cat2;

**quit**;

**data** immig_pph_freqs;

retain ancestry immig_cat2;

length immig_cat2 $ **20.**;

set immig_pph_freqs;

immig_cat2 = '(1B) Immigrant';

if immig_ind = **1**;

drop immig_ind;

**run**;

**data** immig_pph_freqs; set immig_pph_freqs2 immig_pph_freqs; **run**;

**proc** **sort** data=immig_pph_freqs; by ancestry immig_cat2; **run**;

%***cirates***(data = immig_pph_freqs, num = num_pph, denom = total, out = pph_rates, per = **100**, method = poisson);

%***cirates***(data = immig_pph_freqs, num = num_sev_pph, denom = total, out = spph_rates, per = **100**, method = poisson)

/* Get predicted probabilities with interaction term between race variable and duration in Canada: */

**data** modeldata;

length asian_immig_cat $ **21.**;

set redpph.preg_cohort_asian;

if landing_date = **.** and b_bdate = bdate then delete;

else if age < **16** then delete;

if ancestry = 'Asian' then do;

if landing_date ^= **.** then asian_immig_cat = 'Asian (immigrant)';

else if landing_date = **.** then asian_immig_cat = 'Asian (non-immigrant)';

end;

else do;

if landing_date ^= **.** then asian_immig_cat = 'White (immigrant)';

else if landing_date = **.** then asian_immig_cat = 'White (non-immigrant)';

end;

keep born_dad_pph landing_date ancestry ikn pregnancy_id_enc bdate b_bdate asian_immig_cat ancestry_asian3 duration_in_canada;

**run**;

**proc** **genmod** data=modeldata;

class ikn ancestry_asian3(ref="White");

model born_dad_pph = ancestry_asian3 duration_in_canada ancestry_asian3*duration_in_canada / dist=poisson link=log type3;

repeated subject=ikn;

output predicted=predicted lower=lower upper=upper out=pred_probs;

**run**;

**data** redpph.intmod_predprobs;

set pred_probs;

keep ancestry_asian3 pregnancy_id_enc ikn duration_in_canada predicted lower upper;

**run**;

**proc** **genmod** data=modeldata;

class ikn asian_immig_cat(ref="White (non-immigrant)");

model born_dad_pph = asian_immig_cat duration_in_canada asian_immig_cat*duration_in_canada / dist=poisson link=log type3;

repeated subject=ikn;

output predicted=predicted lower=lower upper=upper out=pred_probs;

**run**;

**data** redpph.intmod_predprobs2;

set pred_probs;

keep asian_immig_cat pregnancy_id_enc ikn duration_in_canada predicted lower upper;

**run**;

**data** redpph.preg_cohort_asian;

set redpph.preg_cohort_asian;

if ancestry_asian3 = 'White' then asian_white = 'White';

else asian_white = 'Asian';

**run**;

/* Update model results: */

**%macro** univariate(data=, var=, ref=, num=);

proc genmod data=&data.;

class ikn &var.(ref="&ref") / param=ref;

model born_dad_pph = &var. / dist=poisson link=log;

repeated subject=ikn;

ods output Genmod.GEEEmpPEst = &var._uni_rr;

run;

data &var._uni_rr;

retain Variable Level1 est lower upper probz place1;

length Variable $ **24.**;

set &var._uni_rr;

Variable = "&var";

if Parm = 'Intercept' then delete;

est = round(exp(Estimate), **0.0001**);

lower = round(exp(LowerCL), **0.0001**);

upper = round(exp(UpperCL), **0.0001**);

place1 = &num.;

keep Variable Level1 est lower upper probz place1;

rename probz = pval;

run;

data &var._uni_rr;

set &var._uni_rr;

place2 = _n_;

run;

**%mend**;

%***univariate***(data=redpph.preg_cohort_asian, var=ancestry_asian3, ref=White, num=**1**);

**data** ancestry_asian3_uni_rr;

set ancestry_asian3_uni_rr;

if Level1 = 'Asian (not an immigrant)' then place2 = **1**;

else if Level1 = 'Asian (English)' then place2 = **2**;

else if Level1 = 'Asian (other)' then place2 = **3**;

else if Level1 = 'Asian (Central Asia)' then place2 = **4**;

else if Level1 = 'Asian (East Asia)' then place2 = **5**;

else if Level1 = 'Asian (South Asia)' then place2 = **6**;

else if Level1 = 'Asian (Southeast Asia)' then place2 = **7**;

else if Level1 = 'Asian (West Asia)' then place2 = **8**;

**run**;

**proc** **sort** data=ancestry_asian3_uni_rr; by place2; **run**;

%***univariate***(data=redpph.preg_cohort_asian, var=age_group, ref=**20**-**24**, num=**2**);

**data** age_group_uni_rr;

set age_group_uni_rr;

if Level1 = '<20' then place2 = **1**;

else place2 = _n_ + **1**;

**run**;

**proc** **sort** data=age_group_uni_rr; by place2; **run**;

**proc** **freq** data=redpph.preg_cohort_asian; tables ancestry_asian3*born_dad_pph age_group*born_dad_pph; **run**;

**proc** **genmod** data=redpph.preg_cohort_asian;

class ikn / param=ref;

model born_dad_pph = duration_in_canada / dist=poisson link=log;

repeated subject=ikn;

ods output Genmod.GEEEmpPEst = durinca_uni_rr;

**run**;

**data** durinca_uni_rr;

retain Parm est lower upper probz place1;

length Parm $ **24.**;

set durinca_uni_rr;

if Parm = 'Intercept' then delete;

est = round(exp(Estimate), **0.0001**);

lower = round(exp(LowerCL), **0.0001**);

upper = round(exp(UpperCL), **0.0001**);

keep Parm est lower upper probz place1;

rename probz = pval Parm = Variable;

**run**;

%***univariate***(data=redpph.preg_cohort_asian, var=asian_white, ref=White, num=**1**);

**proc** **freq** data=redpph.preg_cohort_asian; tables asian_white*born_dad_pph; **run**;

/* Multivariate: */

**data** redpph.preg_cohort_asian;

length mode_of_delivery2 $ **32.**;

set redpph.preg_cohort_asian;

if mode_of_delivery in ('(09) US CD' '(10) PM CD') then mode_of_delivery2 = '(09) US/PM CD';

else mode_of_delivery2 = mode_of_delivery;

**run**;

**proc** **genmod** data=redpph.preg_cohort_asian;

class ikn ancestry_asian3(ref="White") age_group(ref="20-24") parity_bin(ref="1") plurality(ref="0") maternal_bmi_cat(ref="18.5-24.9")

age_labourforce_q_da(ref="1") material_resources_q_da(ref="1") households_dwellings_q_da(ref="1") immig_cat2(ref="(1) Not an immigrant")

rural_pccf(ref="N") preg_smoking(ref="0") preg_drug_exposure(ref="0") conception_type(ref="Spontaneous") tri1_prenatal_visit(ref="1")

preex_dm(ref="0") gest_dm(ref="0") preex_hyper(ref="0") gest_hyper(ref="0") previous_cd(ref="0") placenta_previa(ref="0")

placenta_accspec(ref="0") placental_abruption(ref="0") induction(ref="0") augmentation_ind(ref="0") episiotomy(ref="ZNone")

presentation_type(ref="1Cephalic") duration_stage2(ref="<60 minutes") mode_of_delivery2(ref="(01) SVD") gestage_cat(ref="(2) Term")

b_weight_g_cat1(ref="(2) 3000-3999") b_headcirc_cm_cat1(ref="(2) 33-34");

model born_dad_pph = ancestry_asian3 duration_in_canada ancestry_asian3*duration_in_canada age_group parity_bin plurality maternal_bmi_cat

age_labourforce_q_da material_resources_q_da households_dwellings_q_da immig_cat2 rural_pccf preg_smoking preg_drug_exposure conception_type

tri1_prenatal_visit preex_dm gest_dm preex_hyper gest_hyper previous_cd placenta_previa placenta_accspec placental_abruption induction

augmentation_ind episiotomy presentation_type duration_stage2 mode_of_delivery2 gestage_cat b_weight_g_cat1 b_headcirc_cm_cat1

/ dist=poisson link=log;

repeated subject=ikn;

ods output Genmod.GEEEmpPEst = multi_rr;

**run**;

**data** multi_rr;

retain Parm Level1 adj_est adj_lower adj_upper ProbZ;

set multi_rr;

if Parm = 'Intercept' then delete;

adj_est = round(exp(Estimate), **0.0001**);

adj_lower = round(exp(LowerCL), **0.0001**);

adj_upper = round(exp(UpperCL), **0.0001**);

keep Parm Level1 adj_est adj_lower adj_upper ProbZ;

rename ProbZ = adj_pval;

**run**;

**data** multi_rr;

set multi_rr;

if Parm in ('ancestry_asian3' 'duration_*ancestry_a') then do;

if Level1 = 'White' then place = _n_ - **8**;

else if Level1 = 'Asian (not an immigrant)' then place = _n_ - **5**;

else if Level1 = 'Asian (English)' then place = _n_;

else if Level1 = 'Asian (other)' then place = _n_ - **4**;

else if Level1 in ('Asian (Central Asia)' 'Asian (East Asia)') then place = _n_ + **4**;

else if Level1 in ('Asian (South Asia)' 'Asian (Southeast Asia)' 'Asian (West Asia)') then place = _n_ + **3**;

end;

else if Parm = 'age_group' then do;

if Level1 in ('<20' '20-24') then place = _n_ - **4**;

else place = _n_ + **2**;

end;

else if Parm in ('parity_bin' 'tri1_prenatal_visit') then do;

if Level1 = '9' then place = _n_ + **1**;

else if Level1 = '1' then place = _n_ - **1**;

else place = _n_;

end;

else if Parm = 'maternal_bmi_cat' then do;

if Level1 = '<18.5' then place = _n_ - **3**;

else if Level1 = '18.5-24.9' then place = _n_ - **5**;

else if Level1 in ('>=40' 'Unknown') then place = _n_ + **1**;

else place = _n_ + **2**;

end;

else if Parm in ('age_labourforce_q_da' 'material_resources_q' 'households_dwellings') then do;

if Level1 = '1' then place = _n_ - **5**;

else place = _n_ + **1**;

end;

else if Parm = 'immig_cat2' then do;

if Level1 = '(1) Not an immigrant' then place = _n_ - **4**;

else place = _n_ + **1**;

end;

else if Parm = 'rural_pccf' then do;

if Level1 = 'N' then place = _n_ - **2**;

else place = _n_ + **1**;

end;

else if Parm in ('preg_smoking' 'preg_drug_exposure') then do;

if Level1 = '0' then place = _n_ - **2**;

else place = _n_ + **1**;

end;

else if Parm = 'conception_type' then do;

if Level1 = 'Spontaneous' then place = _n_ - **2**;

else place = _n_ + **1**;

end;

else if Parm in ('preex_dm' 'gest_dm' 'preex_hyper' 'gest_hyper' 'previous_cd' 'placenta_previa'

'placenta_accspec' 'placental_abruption' 'induction' 'augmentation_ind') then do;

if Level1 = '0' then place = _n_ - **1**;

else place = _n_ + **1**;

end;

else if Parm = 'episiotomy' then do;

if Level1 = 'ZNone' then place = _n_ - **1**;

else if Level1 = 'Unknown' then place = _n_ + **1**;

else place = _n_;

end;

else if Parm = 'presentation_type' then do;

if Level1 = '1Cephalic' then place = _n_ - **2**;

else if Level1 = '2Breech' then place = _n_;

else place = _n_ + **1**;

end;

else if Parm = 'duration_stage2' then do;

if Level1 = '<60 minutes' then place = _n_ - **5**;

else if Level1 = '60-119 minutes' then place = _n_ - **1**;

else if Level1 in ('120-179 minutes' '180-239 minutes') then place = _n_ + **2**;

else place = _n_ + **1**;

end;

else if Parm = 'mode_of_delivery2' then do;

if Level1 = '(01) SVD' then place = _n_ - **8**;

else place = _n_ + **1**;

end;

else if Parm = 'gestage_cat' then do;

if Level1 = '(2) Term' then place = _n_ - **1**;

else if Level1 = '(3) Post-term' then place = _n_ + **1**;

else place = _n_;

end;

else if Parm in ('b_weight_g_cat1' 'b_headcirc_cm_cat1') then do;

if substr(Level1, **1**, **2**) = '(2' then place = _n_ - **3**;

else if substr(Level1, **1**, **2**) = '(1' then place = _n_;

else place = _n_ + **1**;

end;

else place = _n_;

**run**;

**proc** **sort** data=multi_rr out=multi_rr/*(drop=place)*/; by place; **run**;

**proc** **genmod** data=redpph.preg_cohort_asian;

class ikn asian_white(ref="White") age_group(ref="20-24") parity_bin(ref="1") plurality(ref="0") maternal_bmi_cat(ref="18.5-24.9")

age_labourforce_q_da(ref="1") material_resources_q_da(ref="1") households_dwellings_q_da(ref="1") immig_cat2(ref="(1) Not an immigrant")

rural_pccf(ref="N") preg_smoking(ref="0") preg_drug_exposure(ref="0") conception_type(ref="Spontaneous") tri1_prenatal_visit(ref="1")

preex_dm(ref="0") gest_dm(ref="0") preex_hyper(ref="0") gest_hyper(ref="0") previous_cd(ref="0") placenta_previa(ref="0")

placenta_accspec(ref="0") placental_abruption(ref="0") induction(ref="0") augmentation_ind(ref="0") episiotomy(ref="ZNone")

presentation_type(ref="1Cephalic") duration_stage2(ref="<60 minutes") mode_of_delivery2(ref="(01) SVD") gestage_cat(ref="(2) Term")

b_weight_g_cat1(ref="(2) 3000-3999") b_headcirc_cm_cat1(ref="(2) 33-34");

model born_dad_pph = asian_white duration_in_canada asian_white*duration_in_canada age_group parity_bin plurality maternal_bmi_cat

age_labourforce_q_da material_resources_q_da households_dwellings_q_da immig_cat2 rural_pccf preg_smoking preg_drug_exposure conception_type

tri1_prenatal_visit preex_dm gest_dm preex_hyper gest_hyper previous_cd placenta_previa placenta_accspec placental_abruption induction

augmentation_ind episiotomy presentation_type duration_stage2 mode_of_delivery2 gestage_cat b_weight_g_cat1 b_headcirc_cm_cat1

/ dist=poisson link=log;

repeated subject=ikn;

ods output Genmod.GEEEmpPEst = multi_rr;

**run**;

**data** multi_rr;

retain Parm Level1 adj_est adj_lower adj_upper ProbZ;

set multi_rr;

if Parm = 'Intercept' then delete;

adj_est = round(exp(Estimate), **0.0001**);

adj_lower = round(exp(LowerCL), **0.0001**);

adj_upper = round(exp(UpperCL), **0.0001**);

keep Parm Level1 adj_est adj_lower adj_upper ProbZ;

rename ProbZ = adj_pval;

**run**;

**data** multi_rr;

set multi_rr;

if Parm in ('asian_white' 'duration_*asian_whit') then do;

if Level1 = 'White' then place = _n_ - **1**;

else place = _n_ + **1**;

end;

else if Parm = 'age_group' then do;

if Level1 in ('<20' '20-24') then place = _n_ - **4**;

else place = _n_ + **2**;

end;

else if Parm in ('parity_bin' 'tri1_prenatal_visit') then do;

if Level1 = '9' then place = _n_ + **1**;

else if Level1 = '1' then place = _n_ - **1**;

else place = _n_;

end;

else if Parm = 'maternal_bmi_cat' then do;

if Level1 = '<18.5' then place = _n_ - **3**;

else if Level1 = '18.5-24.9' then place = _n_ - **5**;

else if Level1 in ('>=40' 'Unknown') then place = _n_ + **1**;

else place = _n_ + **2**;

end;

else if Parm in ('age_labourforce_q_da' 'material_resources_q' 'households_dwellings') then do;

if Level1 = '1' then place = _n_ - **5**;

else place = _n_ + **1**;

end;

else if Parm = 'immig_cat2' then do;

if Level1 = '(1) Not an immigrant' then place = _n_ - **4**;

else place = _n_ + **1**;

end;

else if Parm = 'rural_pccf' then do;

if Level1 = 'N' then place = _n_ - **2**;

else place = _n_ + **1**;

end;

else if Parm in ('preg_smoking' 'preg_drug_exposure') then do;

if Level1 = '0' then place = _n_ - **2**;

else place = _n_ + **1**;

end;

else if Parm = 'conception_type' then do;

if Level1 = 'Spontaneous' then place = _n_ - **2**;

else place = _n_ + **1**;

end;

else if Parm in ('preex_dm' 'gest_dm' 'preex_hyper' 'gest_hyper' 'previous_cd' 'placenta_previa'

'placenta_accspec' 'placental_abruption' 'induction' 'augmentation_ind') then do;

if Level1 = '0' then place = _n_ - **1**;

else place = _n_ + **1**;

end;

else if Parm = 'episiotomy' then do;

if Level1 = 'ZNone' then place = _n_ - **1**;

else if Level1 = 'Unknown' then place = _n_ + **1**;

else place = _n_;

end;

else if Parm = 'presentation_type' then do;

if Level1 = '1Cephalic' then place = _n_ - **2**;

else if Level1 = '2Breech' then place = _n_;

else place = _n_ + **1**;

end;

else if Parm = 'duration_stage2' then do;

if Level1 = '<60 minutes' then place = _n_ - **5**;

else if Level1 = '60-119 minutes' then place = _n_ - **1**;

else if Level1 in ('120-179 minutes' '180-239 minutes') then place = _n_ + **2**;

else place = _n_ + **1**;

end;

else if Parm = 'mode_of_delivery2' then do;

if Level1 = '(01) SVD' then place = _n_ - **8**;

else place = _n_ + **1**;

end;

else if Parm = 'gestage_cat' then do;

if Level1 = '(2) Term' then place = _n_ - **1**;

else if Level1 = '(3) Post-term' then place = _n_ + **1**;

else place = _n_;

end;

else if Parm in ('b_weight_g_cat1' 'b_headcirc_cm_cat1') then do;

if substr(Level1, **1**, **2**) = '(2' then place = _n_ - **3**;

else if substr(Level1, **1**, **2**) = '(1' then place = _n_;

else place = _n_ + **1**;

end;

else place = _n_;

**run**;

**proc** **sort** data=multi_rr out=multi_rr/*(drop=place)*/; by place; **run**;

/* Supplemental Table 2 */

**proc** **sql**;

create table region_counts as

select ancestry_asian3, count(*) as total

from redpph.preg_cohort_asian

group by ancestry_asian3;

create table language_counts as

select ancestry_asian3, language, count(*) as count

from redpph.preg_cohort_asian

group by ancestry_asian3, language;

create table lang_by_region as

select * from region_counts as a inner join language_counts as b

on a.ancestry_asian3 = b.ancestry_asian3;

**quit**;

**data** lang_by_region;

retain ancestry_asian3 Language;

set lang_by_region;

if language = '' or ancestry_asian3 = "White" then delete;

percent = count***100**/total;

**run**;

/* Supplemental Table 4: */

**proc** **sql**;

create table cohort as

select a.pregnancy_id_enc, a.ikn, a.b_bdate, a.landing_date, a.language_cat, a.nat_language, a.parity_cat, a.ancestry,

b.born_dad_pph, b.pph_severe,

c.continent, c.WorldRegion, c.Country, c.language

from redpph.preg_cohort as a left join redpph.preg_outcomes as b on a.pregnancy_id_enc = b.pregnancy_id_enc

left join redpph.asian_languages as c on a.nat_language = c.code;

**quit**;

**data** cohort;

set cohort;

if WorldRegion = '' then delete;

**run**;

%***bivartab***(

data = cohort,

byvar = WorldRegion,

catvar = ancestry,

numdq = F,

total = F,

small = F,

trend = F,

stdiff = F,

colpct = T,

mdigits = **2**,

medigits = **0**,

pdigits = **2**,

label = T,

out = asian_wr_tab,

binary = F);

**data** asian_wr_tab; set asian_wr_tab; if VarValue in ('') then delete; drop Variable VarLabel P_value North_Asia; **run**;

/* Additional descriptive table - prenatal screenings: */

**proc** **sql**;

create table psos_records as

select a.pregnancy_id_enc from redpph.preg_cohort as a inner join born.psos as b

on a.pregnancy_id_enc = b.pregnancy_id_enc;

**quit**;

**proc** **sort** data=psos_records nodupkey; by pregnancy_id_enc; **run**;

**proc** **sort** data=redpph.preg_cohort; by pregnancy_id_enc; **run**;

**data** pn_screening;

length mat_bmi2 $ **13.**;

length ctype2 $ **15.**;

merge redpph.preg_cohort(in=a) psos_records(in=b);

by pregnancy_id_enc;

if a and b then pn_screen = **1**; else pn_screen = **0**;

array exist[**15**] plurality smoking_firstvisit smoking_atbirth preg_drug_exposure tri1_prenatal_visit preex_dm gest_dm preex_hyper

previous_cd placenta_previa placenta_accspec placental_abruption induction augmentation_ind ob_trauma;

array new[**15**] plural smoking1 smoking2 drugexpose tri1visit dm1 dm2 hyper1 prevcd previa accspec abruption induct augment obtrauma;

do i = **1** to **15**;

if exist[i] = **1** then new[i] = **1**; else new[i] = **0**;

end;

if maternal_bmi_cat = '<18.5' then mat_bmi2 = '(1) <18.5';

else if maternal_bmi_cat = '18.5-24.9' then mat_bmi2 = '(2) 18.5-24.9';

else if maternal_bmi_cat = '25-29.9' then mat_bmi2 = '(3) 25-29.9';

else if maternal_bmi_cat = '30-34.9' then mat_bmi2 = '(4) 30-34.9';

else if maternal_bmi_cat = '35-39.9' then mat_bmi2 = '(5) 35-39.9';

else if maternal_bmi_cat = '>=40' then mat_bmi2 = '(6) >=40';

else mat_bmi2 = '(7) Unknown';

if conception_type = 'Assisted' then ctype2 = '(2) Assisted';

else if conception_type = 'Spontaneous' then ctype2 = '(1) Spontaneous';

else if conception_type = 'Unknown' then ctype2 = '(3) Unknown';

if preg_induced_hyper = 'Preeclampsia' then pi_hyper = '(1) Preeclampsia';

else if preg_induced_hyper = 'Eclampsia' then pi_hyper = '(2) Eclampsia';

else if preg_induced_hyper = 'HELLP' then pi_hyper = '(3) HELLP';

else if preg_induced_hyper = 'Gestational' then pi_hyper = '(4) Gestational';

else if preg_induced_hyper = '' then pi_hyper = '(5) Missing';

else if preg_induced_hyper = 'None' then pi_hyper = '(6) None';

if episiotomy = 'None' then episiotomy = 'ZNone';

if presentation_type = 'Breech' then presentation_type = '2Breech';

else if presentation_type = 'Cephalic' then presentation_type = '1Cephalic';

if mode_of_delivery = '(09) US/PM CD' then do;

if cs_stage = 'Perimortem' then mode_of_delivery = '(10) PM CD';

else mode_of_delivery = '(09) US CD';

end;

else if mode_of_delivery = '(06) First stage CD' then mode_of_delivery = '(07) First stage CD';

else if mode_of_delivery = '(07) Second stage CD' then mode_of_delivery = '(08) Second stage CD';

else if mode_of_delivery = '(08) CD without labour' then mode_of_delivery = '(06) CD without labour';

drop i;

**run**;

%***bivartab***(

data = pn_screening,

byvar = pn_screen,

numdq = miss_only,

catvar = age_group parity_cat plural mat_bmi2 age_labourforce_q_da material_resources_q_da households_dwellings_q_da immig_cat2

immig_birth_datediff_cat rural_pccf smoking1 smoking2 drugexpose ctype2 tri1visit dm1 dm2 hyper1 pi_hyper prevcd previa

accspec abruption induct augment episiotomy presentation_type mode_of_delivery obtrauma gestage_cat b_weight_g_cat1 b_headcirc_cm_cat1,

numvar = immig_birth_datediff prenatal_visits_combo,

order = age_group parity_cat plural mat_bmi2 age_labourforce_q_da material_resources_q_da households_dwellings_q_da immig_cat2

immig_birth_datediff immig_birth_datediff_cat rural_pccf smoking1 smoking2 drugexpose ctype2 tri1visit

prenatal_visits_combo dm1 dm2 hyper1 pi_hyper prevcd previa accspec abruption induct augment episiotomy presentation_type

mode_of_delivery obtrauma gestage_cat b_weight_g_cat1 b_headcirc_cm_cat1,

total = F,

small = T,

stdiff = T,

trend = F,

binary = T,

out = table1_pn_screen);

**data** table1_pn_screen;

retain VarLabel VarValue pn_screen_1 pn_screen_0;

set table1_pn_screen;

if VarValue = '' then delete;

drop Variable;

keep VarLabel VarValue pn_screen_0 pn_screen_1 Std_Diff;

**run**;

**data** table1_pn_screen;

set table1_pn_screen;

if _n_ = **46** or _n_ = **47** or _n_ = **55** or _n_ = **65** or _n_ = **74** or _n_ = **84** then delete;

**run**;
