# Supplementary material for: Asian-White racial disparities in postpartum hemorrhage and severe postpartum hemorrhage in Ontario, Canada: A population-based cohort study
Source: PLoS One. 2026 Mar 12;21(3):e0344365. doi: 10.1371/journal.pone.0344365 (PMC12981453; doi:10.1371/journal.pone.0344365)
Supplement: S4 File — (DOCX) [file pone.0344365.s012.docx]

| G Project InitiationThis Section must be Completed Prior to Project Dataset(s) Creation | | | | | | | | | |
| --- | --- | --- | --- | --- | --- | --- | --- | --- | --- |
| **Project Title:** | Racial/ethnic disparities in post-partum hemorrhage in Canada | | | | | | | | |
| **Project TRIM number:** | 2023 0908 096 000 | | | | | | | | |
|  | Life | | | | | | | | |
| **Site:** | ICES McMaster | | | | | | | | |
| **Project Purpose and Objectives:** | *Insert Project Purpose and Objectives as listed in the approved ICES Project PIA* | | | | | | | | |
|  | **Purpose**  In a population-based cohort of individuals who gave birth to a liveborn or stillborn infant in Ontario between April 1, 2012 and March 31, 2021, we aim to evaluate the association between maternal race and post-partum hemorrhage (PPH) and assess the role of system, provider, and socio-economic factors on this association.  **Objectives:**  The primary objectives of this project are to:   1. Examine the variation in frequency and temporal trends in PPH and severe PPH by maternal race/ethnicity 2. Quantify the variation in rates of PPH and severe PPH by racial/ethnic group that is explained by differences in:    1. Hospital/provider-level factors (e.g., hospital setting [rural/urban], hospital tier of obstetric service [tier 0-tier 3], provider specialty [obstetrician, family physician, midwife]);    2. Socio-economic factors (e.g., residential instability, material deprivation, dependency).   The secondary objective of the study is to evaluate differences in severe PPH outcomes (prolonged maternal ICU admission or maternal death) by race/ethnicity. | | | | | | | | |
| **ICES Project PIA Initial Approval Date:** | *The ICES Employee or agent who is responsible for creating the Project Dataset(s) is responsible for ensuring there is an approved ICES Project PIA and verifying the date of approval prior to creating the Project Dataset(s)* | | | | | | | | |
|  | March 7, 2023 | | | | | | | | |
| **Principal Investigator (PI):** | *Consult with the RPM to confirm data access level for the PI, as needed* | | | | | | | | |
|  | *Name: Giulia Muraca* | | | *(If PI has access to data)*  Data Access Level:  Analytic environment: RAE  DSH | | | | | |
| **Check the applicable box if the PI is an ICES Student/Trainee** | ICES Student  ICES Fellow  ICES Post-Doctoral Trainee  Visiting Scholar | | | | | | | | |
| **Responsible ICES Scientist:** | *Name the Responsible ICES Scientist if the PI is not a Full Status ICES Scientist* | | | | | | | | |
|  |  | | | | | | | | |
| **Project Team Member(s) Responsible for Project Dataset Creation and/or Statistical Analysis and date joined (list all):** | *All person(s) (ICES Analyst, Appointed Analyst, Analytic Epidemiologist, PI, and/or Student) responsible for creating the Project Dataset(s) and/or statistical analysis on the Research Analytics Environment (RAE) and/or Data Safe Haven (DSH) and the date they joined the project must be recorded* | | | | | | | | |
|  | Giulia M Muraca  Rohan D’Souza | | | | | | 2023-01-01  2023-01-01 | | |
| **Project Team Member(s) who will request RAE and/or DSH folder access (list all):** | *List the project team member responsible for dataset creation who will request access for all members requiring RAE and/or DSH project folder access (e.g. analyst, methodologist, student, etc). Consult with the RPM regarding RAE/DSH environment access, as needed.* | | | | | | | | |
|  | *Name: Giulia Muraca*  *Name:*  *Name:* | RAE  DSH  RAE  DSH  RAE  DSH | | | | yyyy-mon-dd  yyyy-mon-dd  yyyy-mon-dd | | |  |
| **Other ICES Project Team Members and date joined (list all):** | *All other Research Project Team Members (e.g., Research Administrative Assistants, Research Assistants, Project Managers, Epidemiologists) and the date they joined the project must be recorded* | | | | | | | | |
|  | Parnian Hossein Pour | | | | | | 2024-MAY-03 | | |
| **Confirmation that DCP is consistent with Project Objectives:** | *The following individuals must confirm that the ICES Data provided for in this DCP is relevant (e.g., with respect to cohort, timeframe, and variables) and required to achieve the purpose(s) stated in the ICES Project PIA prior to initial Project Dataset creation: 1) PI; 2) Responsible ICES Scientist if the PI is not a Full Status ICES Scientist, or a second ICES Scientist or the Scientific Program Lead if the PI is creating both the DCP and the Project Dataset[s]; 3) ICES Research and Analysis Staff creating the DCP; and 4) ICES Analytic Staff (ICES Employee or agent responsible for creating the Project Dataset[s]). Documentation of this confirmation may be delegated either verbally or via e-mail.* | | | | | | | | |
|  | ***Principal Investigator*** | | | |  | | | ***2023-Jun-12*** | |
|  | ***Responsible ICES Scientist or Second ICES Scientist/Lead*** | | | |  | | 2023-Jul-17 | | |
|  | ***ICES Research and Analysis Staff Creating the DCP*** | | | |  | | yyyy-mon-dd | | |
|  | ***ICES Analytic Staff*** | | | |  | | yyyy-mon-dd | | |
|  |  | | | | | | | | |
| **Designated ICES Research and Analysis Staff accountable for Project Documentation:** | *The person named (ICES staff) is accountable for ensuring that the approved ICES Project PIA, ICES Project PIA Amendments, and DCP are saved on the T Drive, ensuring ICES Project PIA Amendments are submitted as required, ensuring DCP Amendments are documented, and sharing the final DCP with the PI/Responsible ICES Scientist at project completion* | | | | | | | | |
|  |  | | | | | | | | |
| **DCP Creation Date and Author:** | *Date DCP was finalized prior to Project Dataset(s) creation* | | *Name of person who created the DCP* | | | | | | |
|  | ***Date*** | | ***Name*** | | | | | | |
|  | 2023-June-12 | | Giulia Muraca | | | | | | |

| ICES DataThis Section must be Completed Prior to Project Dataset(s) Creation | |
| --- | --- |
| *The ICES Employee or agent who is responsible for creating the Project Dataset(s) must ensure that this list includes only data listed in the ICES Project PIA*  *Changes to this list after initial ICES Project PIA approval require an ICES Project PIA Amendment* | *Mandatory for all datasets that are available by individual year* |
| ***General Use Datasets – Health Services*** | ***Years (where applicable)*** |
| CIHI DAD | Apr 1 2012-May 12, 2022 |
| NACRS | Apr 1 2012-May 12, 2022 |
| OHIP | July 1, 2011-Mar 31, 2022 |
| See list |  |
| ***General Use Datasets – Care Providers*** |  |
| See list |  |
| See list |  |
| ***General Use Datasets – Population*** |  |
| RPDB | Apr 1 2012-Mar 31 2021 |
| See list |  |
| ***General Use Datasets – Coding/Geography*** |  |
| REF | 2012-2021 |
| PCCF | Apr 1 2012-Mar 31 2021 |
|  |  |
| ***General Use Datasets - Facilities*** |  |
| See list |  |
| ***General Use Datasets - Other*** |  |
| MOMBABY | Apr 1 2012-Mar 31 2021 |
| ODD | Apr 1 1991-Mar 31 2021 |
| ONMARG | 2011, 2016, (2021 if available) |
| HYPER | Apr 1 1991-Mar 31 2021 |
| ***Controlled Use Datasets*** |  |
| CIC | Inception to -Mar 31, 2021 |
| BORN | Apr 1 2012-Mar 31 2021 |
| ***Other Datasets (including PSD and PDC data)*** |  |
|  |  |
|  |  |

| Project Amendments and Reconciliation | | | |
| --- | --- | --- | --- |
| **ICES Project PIA Amendment History (add additional rows as needed):** | *Privacy approval date* | *Person who submitted amendment* | *Note that any changes to the list of ICES Data or Project Objectives require an ICES Project PIA Amendment* |
|  | ***Date*** | ***Name*** | ***Amendment*** |
|  | yyyy-mon-dd |  |  |
| **DCP Amendment History (add additional rows as needed):** | *Date DCP amended* | *Person who made the DCP amendment* | *Note that any DCP amendments involving changes to the list of ICES Data or Project Objectives require an ICES Project PIA Amendment* |
|  | ***Date*** | ***Name*** | ***Amendment*** |
|  | 2024-MAY-03 | Ana Gayowsky | Adding project team member |
| **Date Programs/DCP reconciled** | *The person(s) creating the dataset and/or analyzing the data are responsible for ensuring that the final DCP reflects the final program(s) when the project is completed* | | |
|  | yyyy-mon-dd | | |

| Project Cohort | | |
| --- | --- | --- |
| **Study Design** | Cohort study  Matched cohort study  Case-control study  Cross-sectional study  Other (specify): | |
| **Index Event / Inclusion Criteria**  *(please ensure index event / inclusion criteria are specified with data sources, variables, study period and values or codes)* | All people in Ontario with one or more singleton or multiple obstetrical deliveries of a liveborn or stillborn infant after 20 weeks gestational age with a delivery date between April 1, 2013 and March 31, 2021. (using records from BORN)  We will identify deliveries using the BORN datafile. The index event will be the delivery. | |
| **Estimated Size of Cohort** | 1,323,000 | |
| **Exclusions** *(in order)  (common exclusions are listed in grey italics for consideration)*  (Use Aggregate pregnancy dataset) | *Step* | Description |
|  | 1 | Missing pregnancy_id in BORN |
|  | 2 | Invalid IKN |
|  | 3 | Duplicate pregnancy_id |
|  | 4 | Non-Ontario residents at index (b_bdate) for each individual birth  (If in RPDB: substr(prcddablk,1,2) ne ‘35’ |
|  | 5 | Pregnancies ending in miscarriages <20 weeks gestation (140 days) and all induced abortions   - agg_pregnancy_outcome_ID in (1021035, 1021040, 1021050, 1021070) in BORN.AGG_PREGNANCY |
|  | 6 | Maternal date of death recorded as prior to b_bdate |
|  | 7 | More than one landing date in CIC |
| Additional Exclusions for Objective 2 (*in order)* | 1 |  |
|  | 2 |  |
|  | 3 |  |
|  | 11 |  |

| Project Time Frame Definitions | | |
| --- | --- | --- |
| Look-back Window  Observation Window  (in which to look for outcomes)  **Index Event Date**  Accrual Window  Max Follow-up Date | |  |
| **Accrual Start/End Dates** | April 1, 2013 to March 31, 2021 |  |
| **Max Follow-up Date** | March 31, 2022 |  |
| **When does observation window terminate?** | In CIHI DAD, NACRS, and OHIP: 365 days after delivery date so that everyone is followed from the delivery plus 12 months postpartum, to a maximum of March 31, 2022 |  |
| **Lookback Window(s)**  *(please ensure lookback windows are defined with start and end dates and in relation to the index event date)* | In CIC: 36 years from end of accrual window (starts June 1, 1985, ends March 31, 2021)  In derived disease cohorts (ODD, HYPER) (starts Apr 1^st^ 1991 to Mar 31, 2021)  In ONMARG: 10 years from end of accrual window (2011, 2016, ?2021 if available?)  In OHIP: 9 month look-back window (July 1, 2011). |  |

| Variable Definitions (add additional rows as needed) *A few key points to keep in mind:*   1. *Please ensure codes, data sources, diagnosis types and lookback periods (if applicable) are provided for all definitions listed below and that codes are provided in Excel format. If borrowing codes from another project, please list all the codes here* 2. *There are maximum number of digits that can be specified using ICES data (ICD 9 CA codes are up to maximum of 4 digits, ICD 10 CA codes are 6 digits, OHIP diagnosis codes are 3 digits)* | | |
| --- | --- | --- |
| **Main Exposure or Risk Factor** | **Maternal race**:   - Using BORN: Use variable ‘ancestry’ in PSOS as the primary source - To supplement any records that do not have ancestry information in PSOS, use variable ‘maternal_ancestry_id’ in AS   - 1006295, 1006300=Asian   - 1006350=White   - 1006330=Black   - 1006440=Other   - 1006290, 1006460= Unknown |  |
| **Primary Outcome Definition** | \| Primary outcome \| ICD‐10-CA, CCI codes, and other variables \| BORN variable(s) \| \| --- \| --- \| --- \| \| Postpartum hemorrhage (PPH) \| ICD-10-CA: 072.0 to 072.3 within 6 weeks of delivery \| BORN.AGG_PREG in ‘lbr_and_birth_complication_id ‘=1014540 \| \| Severe PPH (one or more of the following) \| \| \| \| \| Postpartum hemorrhage with blood transfusion \| ICD-10-CA: O72 + any of the following  CCI: 1LZ19  DAD data element: ‘BTREDBC’ = ‘1’  within 6 weeks of delivery \| PPH+transfusion:  ‘postpartum_complication_id’ in  BORN.PPM=3100563  OR ‘postpartum_complication_id’  BORN.MW_PREG_COC=3100563 \| \| Postpartum hemorrhage with hysterectomy \| ICD-10-CA: O72 + any of the following  CCI: 5MD60KE; 5MD60RC; 5MD60CB; 5MD60RD; 1RM87LAGX; (1RM89LA without 1PL74;1RS80; 1RS74) within 6 weeks of delivery \| Hysterectomy: ‘lbr_and_birth_complication_id’=1014475  OR  ‘postpartum_complication_id’=1020090 \| \| Postpartum hemorrhage with procedures to control bleeding \| ICD-10-CA: O72 +  DAD data element: ‘BTREDBC’ = 1 + any of the following  CCI: 5PC91LA, 1RM13, 1KT51; 5PC91HT  within 6 weeks of delivery \|  \| |  |
| **Secondary Outcome Definition(s)** | **Maternal length of stay in ICU**   - Identify those transferred to ICU within up to 6 weeks from the index delivery with:   - Using ‘Institution To’, ‘Discharge Disposition’, and ‘Institution To Type’ in CIHI DAD or ‘FTSPCU’ in (‘10’, ‘20’, ‘25’, ‘30’, ‘35’, ‘40’, ‘45’, ‘60’, ‘80’) - Calculate length of stay in ICU (hours) by date of discharge subtracted by date of admission using   - In CIHI DAD: ‘SCU Discharge Date/Time’ – ‘SCU Admit date/time’ or   - In CIHI DAD: ‘SCUHRS’ (Difference between the ICU Admit Date/Time and ICU Discharge Date/Time [hours])   **Prolonged maternal ICU stay**   - Admission > 24 hours up to 6 weeks from the index delivery - CIHI DAD data element: ‘FTSPCU’ in (‘10’,‘20’,‘25’,‘30’,‘35’,‘40’,‘45’,‘60’,‘80’)   + ‘SCUHRS’ (Difference between the ICU Admit Date/Time and ICU Discharge Date/Time [hours])  **Maternal death**   - Maternal death up to 12 months from the index delivery   - RPDB data element: ‘DTHDATE’   **Length of stay**   - Difference between the Admission Date and Discharge Date (days)   - CIHI DAD Data element ‘LOS’   - At delivery   - In the 6 weeks post delivery   - Total # of days   **Prolonged length of stay**   - Prolonged (≥7 days) hospital stay (Yes/No)   - CIHI DAD Data element ‘LOS’ ≥7 days   **ED visits in 6 weeks following delivery**   - Number of ED days   - For delivery   - In the 6 weeks post delivery   - Total # of days   **Total hospital and ED days 6 weeks following delivery**   - - For delivery   - In the 6 weeks post delivery   - Total # of days |  |
| **Baseline Characteristics**  *For details re: source variable and values, see ‘Data dictionary – Homebirth – Darling_2022-12-12’* | **Maternal Age**   - ‘b_bdate’ from RPDB into the following categories   - <20   - 20-24   - 25-34   - 35-39   - ≥40   - Missing   **Parity**   - ‘parity’ in BORN.AGG_Pregnancy - Categorize into   - Nulliparous (0) = 0   - Parous = >0     - 1 = 1     - 2-3 = 2 or 3     - 4+ = >3   **Plurality**   - ‘NUMBER_OF_FETUSES’ >1 in BORN.AGG_PREGNANCY; or - “Consult_Reason_id” = 1025340 in BORN.MW_Pregnancy_COC   **Primary Language**   - “primary_language_ID” in BORN.AGG_Pregnancy (1=English, 2=French, 3=Other) - CIC: Use “NAT_LANGUAGE” variable   **Pre-pregnancy body mass index (kg/m^2^)**   - ‘maternal_BMI’ in BORN.AGG_PREG in categories   - 18.5   - 18.5-24.9   - 25.0-29.9   - 30.0-34.9   - 35.0-39.9   - ≥40.0   - Missing/ unknown   **Material Deprivation Quintile**   - From ON-MARG   - Q1 (least deprived)   - Q2   - Q3   - Q4   - Q5 (most deprived)   - Missing/ unknown   **Geographic location**   - From PCCF   - Urban   - Rural   - Missing/unknown   **Tobacco use in pregnancy**   - ‘matsmokingatfirstprenvisit’ in BORN.AGG_Pregnancy   - No = 1017430   - Yes = 1017440, 1017450, 1017460, 1017470   - Unknown/missing = 1017475 or missing - Variable mat_smoking_at_adm_for_birth in BORN.AGG_Pregnancy   - No = 1017380   - Yes = 1017390, 1017400, 1017410, 1017420   - Unknow/missing = 1017425 or missing   **Drug and substance exposure in pregnancy**   - “ expos_drug_and_subst_ID” in BORN.AGG_pregnancy   - Yes = 1020460, 1020470, 1020480, 1020490, 1020500, 1020510, 1020520, 1020530   - No = 1020457   - Unknown/missing = 1020535 or missing   **Type of conception**   - ‘conception_type_ID’ in BORN.AGG_pregnancy   - Spontaneous = 1013160   - Assisted = 1013110, 1013120, 1013130, 1013140, 1013150, 1013170, 300006   - Unknown = 1013180 or missing   **First trimester prenatal visit**   - Variable “first_trimester_visit_flag” on BORN.AGG_pregnancy dataset   **Number of prenatal visits**   - For physician clients: sum of prenatal visits using OHIP service codes   - P002=High risk prenatal assessment   - P003=General assessment (major prenatal visit)   - P004=Minor prenatal assessment   - P005=Antenatal preventative health assessment - For midwifery clients: Sum of Variables from all records in BORN.MW_pregnancy_COC:   - Mw_prenatalnumofvisitshome   - Mw_prenatalnumofvisitshospital   - Mw_prenatalnumofvisitsclinic   **Gestational diabetes**   - “diabetes_and_pregnancy_ID” in BORN.AGG_pregnancy   - Yes=1013430, 1013440, 1013450, 1013460, 1013465, 1013470, 1013480, 1013490, 300001   - No=1013390 - ICD-10 Dx codes = (O24.8, P70.0) in CIHI DAD   **Pre existing diabetes**   - ‘DIAG_Last’ in ICES_ODD dataset - “diabetes_and_pregnancy_id” (in BORN.AGG_PREGNANCY)   - Type II in (1013510, 1013520, 1013530, 1013540, 1013545, 1013550, 1013560, 1013570)   - Type I in (1013500)   **Pregnancy induced hypertension (Preeclampsia/eclampsia/HELLP)**  Preeclampsia   - “Preg_hypertension_disorder_ID” (in BORN.AGG_PREGNANCY) = 1020840, 1020854, 1020850 - “All_ind_for_lbd_induct_ID” (in BORN.AGG_PREGNANCY) =1014330 - “All_ind_for_CS_id” (in BORN.AGG_PREGNANCY) =1023800 - ICD-10 Dx code = (O11-O15)   Eclampsia   - “Preg_hypertension_disorder_ID” (in BORN.AGG_PREGNANCY) = 1020800   HELLP syndrome   - “preg_hypertension_disorder_ID” = 1020820 - ICD-10 Dx code = (O14)   **Chronic (pre-existing hypertension)**   - ‘DIAGDATE_LAST’ in ICES HYPER dataset - “Preg_hypertension_disorder_ID” (in BORN.AGG_PREGNANCY) " =1020840 (existing hypertension with superimposed preeclampsia) - "antenatal_consult_reason_all_id" in (1025170) in BORN.AG   **Previous cesarean delivery**   - If “num_of_prev_cs_births” >0 in BORN.AGG_PREGNANCY then “prev_CD”=1   **Maternal duration of residence in Canada:**   - Date of delivery subtracted by date of landing in Canada using ‘LANDING_DATE’ in CIC – ‘birth-date’ in BORN.AGG_INFANT or BORN.MW_BIRTH_COC in BORN   **Maternal immigrant/refugee status**: CIC.   - Immigration status: Please classify women who immigrated to Canada from 1985 onward (inception of CIC database) as immigrants; else, classify as long-term residents (born in Canada or emigrated prior to 1985) - Immigration category: Use variable immigration_category and apply format $cic_immigcateg_ircc_5cat. Records in CIC_IRCC are categorized into 5 groups using this format (to be collapsed as immigrant, refugee, or permanent resident):   - Economic (economic class) immigrants   - Sponsored family (family class) immigrants   - Resettled Refugee & Protected Person in Canada   - Other immigrants   - Category not stated   **Placental abnormalities**   - Placenta previa   - ‘complication_id’ = 1020340 in BORN.AGG_PREGNANCY   - ICD-10 Dx code ‘O44’ in CIHI DAD - Placenta accreta spectrum   - ‘complication_id’ = 1020310, 1020320, 1020330 in BORN.AGG_PREGNANCY   - ICD-10 Dx code ‘O432’ in CIHI DAD - Placental abruption   - ‘complication_id’=1020300 in BORN.AGG_PREGNANCY   - ICD-10 Dx code ‘O45’ in CIHI DAD   **Fetal presentation**  in BORN.AGG_PREGNANCY and MW_pregnancy_COG datasets   - “presentation_type_id” IN:   - cephalic (1021190, 1021180, 3000088, 1021160, 1021150, 1021170)   - breech (1021130, 1021120, 1021110, 1021100,3000089, 1021140)   - transverse (1021200)   - unknown (1021205)   **Mode of delivery**   - “birth_type_id” in BORN.AGG_PREGNANCY   - 1012920=Vaginal delivery   - 1012910=Spontaneous vaginal delivery (SVD)   - 1012880=Operative vaginal delivery (OVD)   - 1012890=Induced or spontaneous labour cesarean delivery (CD)   - 1012900=No labour CD   - 1012925=unknown   **OVD type**   - “forceps_vacuum_id” (in BORN.AGG_PREGNANCY)   - 1013830=Forceps   - 1013850=Vacuum   - 1013860=Vacuum and forceps   - 1013865=Unknown   - 1013840=None   **Stage of cesarean delivery**   - “CS_stage_id” (in BORN.LBM)   - 1005730=First stage   - 1005750=Second stage   - 3000021=No labour   - 1048725=Unknown   **Type of cesarean delivery**   - “CS_type_ID” (in BORN.LBM)   - 1013360=Planned (as scheduled)   - 1013370=Planned (not as scheduled)   - 1048727=Planned   - 1013380=Unplanned   - 1012445=Unknown   **Duration of second stage (mins)**   - “lbr_time_sec_stg_total_minutes” (in BORN.AGG_PREGNANCY) - As a continuous variable in mean and SD and in categories as follows:   - <60   - 60-119   - 120-179   - 180-239   - ≥240   - No second stage of labour (“CS_stage_id” (in BORN.LBM)=3000021 and/or “CS_type_ID” (in BORN.LBM)= 1013360, 1013370, 1048727 or “CS_dilation_cm”<10 (in BORN.AGG_PREG)= 1013230, 1013240, 1013250, 1013260, 1013270, 1013280, 1013290, 1013300, 1013310, 1013320   **Gestational age**   - ‘GA_at_birth_days’ in BORN.AGG_INFANT - ‘GA_at_birth_weeks’ in BORN.AGG_INFANT   - <37 weeks=preterm   - 37-41 weeks=term   - >41 weeks=post-term   **Induction**   - CIHI DAD CCI code (5.AC.30) - BORN.AGG_PREG “labour_induction_method_id” in (1014610, 1014627, 1014620, 1048728, 1014625, 3000008, 3000119) - check “number_of_induction_attempts” and check “all_ind_for_lbr_induct_id”   **Augmentation**   - BORN.AGG_PREG "augmentation_id" (1012690, 1012715, 1012680, 1012700, 1012710)   **Episiotomy**   - AGG_PREG "episiotomy_type_id"   - 1013630 =Midline   - 1013620 =Mediolateral   - 1013650 =Unknown   - 1013640 =None - CIHI DAD CCI codes: 5.MD.53.KJ, 5.MD.53.KL, 5.MD.53.KN, 5.MD.54.KJ, 5.MD.54.KL, 5.MD.54.KN, 5.MD.54.NF, 5.MD.55.KN, 5.MD.55.KL, 5.MD.55.KJ, 5.MD.55.KR, 5.MD.50.GH, 5.PC.80.JN = ‘Unknown’   **Infant birth weight**   - “birth_weight_grams” in BORN.AGG_INFANT   - <3000   - 3000-3999   - 4000-4499   - ≥4500   - Missing (“birth_weight_is_unknown_flag”=yes in BORN.AGG_INFANT)   **Infant head circumference (*retired in 2018*)**   - “Head_circumference_at_birth_cm” in BORN.AGG_INFANT   - <33   - 33-34   - 35-36   - ≥37   - Missing   **Obstetric trauma**   - CIHI DAD ICD-10 codes:O70.2, O70.3, O71.3, O71.4, O71.5, O71.6, O71.7, O71.8, O71.9 during admdate <= b_bdate <= ddate - CIHI DAD CCI codes: 5.PC.80.JQ, 5.PC.80.JJ, 5.PC.80.JU, 5.PC.80.JR, 5.PC.80.JH, 5.PC.80.JK, 5.PC.80.JL during admdate <= b_bdate <= ddate - OHIP billing codes: P045, P046, P039, P036 during (b_bdate – 3) <= servdate <= (b_bdate + 3) - BORN variables:   - AGG_PREG “perineal_laceration_id” = 1019960, 1019970, 1019980   - MW_PREGNANCY_COC “intrapartum_cons_reas_id” = 1017902, 1017903, 1017904 |  |
| **Other Variables** |  |  |

**Table 1. Maternal demographic, obstetric and neonatal characteristics by maternal race/ethnicity, Ontario, 2012-2021**

|  | **Total** | **Black** | **White** | **Asian** | **Missing/Unknown** |
| --- | --- | --- | --- | --- | --- |
| **Characteristic** | **N=** | **N=** | **N=** | **N=** | **N=** |
|  | **n (%)** | **n (%)** | **n (%)** | **n (%)** | **n (%)** |
| **Maternal age (years)** |  |  |  |  |  |
| <20 |  |  |  |  |  |
| 20-24 |  |  |  |  |  |
| 25-34 |  |  |  |  |  |
| 35-39 |  |  |  |  |  |
| ≥40 |  |  |  |  |  |
| Missing/ unknown |  |  |  |  |  |
| **Parity** |  |  |  |  |  |
| Nulliparous |  |  |  |  |  |
| Parous |  |  |  |  |  |
| 1 |  |  |  |  |  |
| 2-3 |  |  |  |  |  |
| 4+ |  |  |  |  |  |
| Missing/ unknown |  |  |  |  |  |
| **Plurality** |  |  |  |  |  |
| Singleton |  |  |  |  |  |
| Multiple |  |  |  |  |  |
| **Primary Language** |  |  |  |  |  |
| French or English |  |  |  |  |  |
| Other |  |  |  |  |  |
| Missing/ unknown |  |  |  |  |  |
| **Pre-pregnancy body mass index (kg/m^2^)** |  |  |  |  |  |
| <18.5 |  |  |  |  |  |
| 18.5-24.9 |  |  |  |  |  |
| 25.0-29.9 |  |  |  |  |  |
| 30.0-34.9 |  |  |  |  |  |
| 35.0-39.9 |  |  |  |  |  |
| ≥40.0 |  |  |  |  |  |
| Missing/ unknown |  |  |  |  |  |
| **Material deprivation (ON-MARG quintile)** |  |  |  |  |  |
| Q1 (least deprived) |  |  |  |  |  |
| Q2 |  |  |  |  |  |
| Q3 |  |  |  |  |  |
| Q4 |  |  |  |  |  |
| Q5 (most deprived) |  |  |  |  |  |
| Missing/ unknown |  |  |  |  |  |
| **Maternal immigrant/**  **refugee status** |  |  |  |  |  |
| Not an immigrant |  |  |  |  |  |
| Economic |  |  |  |  |  |
| Family |  |  |  |  |  |
| Resettled Refugee & Protected Person |  |  |  |  |  |
| Other |  |  |  |  |  |
| Not stated |  |  |  |  |  |
| **Maternal duration or residence in Canada (years)** |  |  |  |  |  |
| (mean, SD, median, IQR) |  |  |  |  |  |
| <1 |  |  |  |  |  |
| 1-5 |  |  |  |  |  |
| 6-10 |  |  |  |  |  |
| >10 |  |  |  |  |  |
| **Geographic location** |  |  |  |  |  |
| Urban |  |  |  |  |  |
| Rural |  |  |  |  |  |
| Missing/ unknown |  |  |  |  |  |
| **Tobacco use in pregnancy** |  |  |  |  |  |
| Yes |  |  |  |  |  |
| No |  |  |  |  |  |
| Missing/ unknown |  |  |  |  |  |
| **Drug and substance exposure in pregnancy** |  |  |  |  |  |
| Yes |  |  |  |  |  |
| No |  |  |  |  |  |
| Missing/ unknown |  |  |  |  |  |
| **Type of conception** |  |  |  |  |  |
| Spontaneous |  |  |  |  |  |
| Assisted |  |  |  |  |  |
| Missing/ unknown |  |  |  |  |  |
| **First trimester prenatal visit** |  |  |  |  |  |
| Yes |  |  |  |  |  |
| No |  |  |  |  |  |
| Missing/ unknown |  |  |  |  |  |
| **Number of prenatal visits* (mean, SD)** |  |  |  |  |  |
| Missing/ unknown |  |  |  |  |  |
| **Pre-existing diabetes** |  |  |  |  |  |
| Yes |  |  |  |  |  |
| No |  |  |  |  |  |
| **Gestational diabetes** |  |  |  |  |  |
| Yes |  |  |  |  |  |
| No |  |  |  |  |  |
| **Pre-existing hypertension** |  |  |  |  |  |
| Yes |  |  |  |  |  |
| No |  |  |  |  |  |
| **Pregnancy induced hypertension** |  |  |  |  |  |
| Preeclampsia |  |  |  |  |  |
| Eclampsia |  |  |  |  |  |
| HELLP Syndrome |  |  |  |  |  |
| **Previous cesarean delivery** |  |  |  |  |  |
| Yes |  |  |  |  |  |
| No |  |  |  |  |  |
| Missing/ unknown |  |  |  |  |  |
| **Placental previa** |  |  |  |  |  |
| Yes |  |  |  |  |  |
| No |  |  |  |  |  |
| **Placenta accreta spectrum** |  |  |  |  |  |
| Yes |  |  |  |  |  |
| No |  |  |  |  |  |
| **Placental abruption** |  |  |  |  |  |
| Yes |  |  |  |  |  |
| No |  |  |  |  |  |
| **Induction** |  |  |  |  |  |
| Yes |  |  |  |  |  |
| No |  |  |  |  |  |
| **Augmentation** |  |  |  |  |  |
| Yes |  |  |  |  |  |
| No |  |  |  |  |  |
| **Episiotomy** |  |  |  |  |  |
| Mediolateral |  |  |  |  |  |
| Midline |  |  |  |  |  |
| Unknown |  |  |  |  |  |
| None |  |  |  |  |  |
| **Fetal presentation** |  |  |  |  |  |
| Cephalic |  |  |  |  |  |
| Breech |  |  |  |  |  |
| Transverse |  |  |  |  |  |
| Missing/ unknown |  |  |  |  |  |
| **Duration of second stage of labour (mins)** |  |  |  |  |  |
| (mean, SD, median, IQR) |  |  |  |  |  |
| <60 |  |  |  |  |  |
| 60-119 |  |  |  |  |  |
| 120-179 |  |  |  |  |  |
| 180-239 |  |  |  |  |  |
| ≥240 |  |  |  |  |  |
| No second stage |  |  |  |  |  |
| **Mode of delivery** |  |  |  |  |  |
| Vaginal delivery |  |  |  |  |  |
| Spontaneous vaginal delivery (SVD) |  |  |  |  |  |
| Forceps delivery |  |  |  |  |  |
| Vacuum delivery |  |  |  |  |  |
| Forceps + vacuum delivery |  |  |  |  |  |
| Cesarean delivery (CD) |  |  |  |  |  |
| First stage CD |  |  |  |  |  |
| Second stage CD |  |  |  |  |  |
| CD without labour |  |  |  |  |  |
| Missing/unknown |  |  |  |  |  |
| **Gestational age (weeks)** |  |  |  |  |  |
| Preterm (<37) |  |  |  |  |  |
| Term (37-41) |  |  |  |  |  |
| Post term (≥42) |  |  |  |  |  |
| **Infant birth weight (g)** |  |  |  |  |  |
| <3000 |  |  |  |  |  |
| 3000-3999 |  |  |  |  |  |
| 4000-4499 |  |  |  |  |  |
| ≥4500 |  |  |  |  |  |
| Missing |  |  |  |  |  |
| **Infant head circumference at birth (cm)** |  |  |  |  |  |
| <33 |  |  |  |  |  |
| 33-34 |  |  |  |  |  |
| 35-36 |  |  |  |  |  |
| ≥37 |  |  |  |  |  |
| Missing |  |  |  |  |  |

**Table 2. Risk ratios and 95% CI for PPH, Ontario, 2012-2021**

| **Characteristic** | **Unadjusted (univariate) RR (95% CI)** | **p-value** | **Adjusted (multivariate)**  **RR (95% CI)** | **p-value** |
| --- | --- | --- | --- | --- |
| **Maternal Race** |  |  |  |  |
| Black |  |  |  |  |
| White | 1.0 (REF) |  | 1.0 (REF) |  |
| Asian |  |  |  |  |
| **Maternal age (years)** |  |  |  |  |
| <20 |  |  |  |  |
| 20-24 | 1.0 (REF) |  | 1.0 (REF) |  |
| 25-34 |  |  |  |  |
| 35-39 |  |  |  |  |
| ≥40 |  |  |  |  |
| Missing/ unknown |  |  |  |  |
| **Parity** |  |  |  |  |
| Nulliparous |  |  |  |  |
| Parous | 1.0 (REF) |  | 1.0 (REF) |  |
| **Plurality** |  |  |  |  |
| Singleton | 1.0 (REF) |  | 1.0 (REF) |  |
| Multiple |  |  |  |  |
| **Primary Language** |  |  |  |  |
| French or English | 1.0 (REF) |  | 1.0 (REF) |  |
| Other |  |  |  |  |
| Missing/ unknown |  |  |  |  |
| **Pre-pregnancy body mass index (kg/m^2^)** |  |  |  |  |
| <18.5 |  |  |  |  |
| 18.5-24.9 | 1.0 (REF) |  | 1.0 (REF) |  |
| 25.0-29.9 |  |  |  |  |
| 30.0-34.9 |  |  |  |  |
| 35.0-39.9 |  |  |  |  |
| ≥40.0 |  |  |  |  |
| Missing/ unknown |  |  |  |  |
| **Material deprivation (ON-Marg quintile)** |  |  |  |  |
| Q1 (least deprived) | 1.0 (REF) |  | 1.0 (REF) |  |
| Q2 |  |  |  |  |
| Q3 |  |  |  |  |
| Q4 |  |  |  |  |
| Q5 (most deprived) |  |  |  |  |
| Missing/ unknown |  |  |  |  |
| **Maternal immigrant/refugee status** |  |  |  |  |
| Not an immigrant | 1.0 (REF) |  | 1.0 (REF) |  |
| Economic |  |  |  |  |
| Family |  |  |  |  |
| Resettled Refugee & Protected Person |  |  |  |  |
| Other |  |  |  |  |
| Not stated |  |  |  |  |
| **Maternal duration or residence in Canada (years)** |  |  |  |  |
| <1 |  |  |  |  |
| 1-5 |  |  |  |  |
| 6-10 |  |  |  |  |
| >10 | 1.0 (REF) |  | 1.0 (REF) |  |
| **Geographic location** |  |  |  |  |
| Urban | 1.0 (REF) |  | 1.0 (REF) |  |
| Rural |  |  |  |  |
| Missing/ unknown |  |  |  |  |
| **Tobacco use in pregnancy** |  |  |  |  |
| Yes |  |  |  |  |
| No | 1.0 (REF) |  | 1.0 (REF) |  |
| Missing/ unknown |  |  |  |  |
| **Drug and substance exposure in pregnancy** |  |  |  |  |
| Yes |  |  |  |  |
| No | 1.0 (REF) |  | 1.0 (REF) |  |
| Missing/ unknown |  |  |  |  |
| **Type of conception** |  |  |  |  |
| Spontaneous | 1.0 (REF) |  | 1.0 (REF) |  |
| Assisted |  |  |  |  |
| Missing/ unknown |  |  |  |  |
| **First trimester prenatal visit** |  |  |  |  |
| Yes | 1.0 (REF) |  | 1.0 (REF) |  |
| No |  |  |  |  |
| Missing/ unknown |  |  |  |  |
| **Pre-existing diabetes** |  |  |  |  |
| Yes |  |  |  |  |
| No | 1.0 (REF) |  | 1.0 (REF) |  |
| **Pre-existing hypertension** |  |  |  |  |
| Yes |  |  |  |  |
| No | 1.0 (REF) |  | 1.0 (REF) |  |
| **Previous cesarean delivery** |  |  |  |  |
| Yes |  |  |  |  |
| No | 1.0 (REF) |  | 1.0 (REF) |  |
| Missing/ unknown |  |  |  |  |
| **Gestational diabetes** |  |  |  |  |
| Yes |  |  |  |  |
| No | 1.0 (REF) |  | 1.0 (REF) |  |
| Missing/ unknown |  |  |  |  |
| **Pregnancy induced hypertension (Preeclampsia, eclampsia, HELLP)** |  |  |  |  |
| Yes |  |  |  |  |
| No | 1.0 (REF) |  | 1.0 (REF) |  |
| Placenta previa |  |  |  |  |
| Yes |  |  |  |  |
| No | 1.0 (REF) |  | 1.0 (REF) |  |
| Placenta accreta spectrum |  |  |  |  |
| Yes |  |  |  |  |
| No | 1.0 (REF) |  | 1.0 (REF) |  |
| Placental abruption |  |  |  |  |
| Yes |  |  |  |  |
| No | 1.0 (REF) |  | 1.0 (REF) |  |
| **Duration of second stage of labour (min)** |  |  |  |  |
| <60 | 1.0 (REF) |  | 1.0 (REF) |  |
| 60-119 |  |  |  |  |
| 120-179 |  |  |  |  |
| 180-239 |  |  |  |  |
| ≥240 |  |  |  |  |
| **Mode of delivery** |  |  |  |  |
| Spontaneous vaginal delivery | 1.0 (REF) |  | 1.0 (REF) |  |
| Forceps delivery |  |  |  |  |
| Vacuum delivery |  |  |  |  |
| Forceps + vacuum delivery |  |  |  |  |
| First stage CD |  |  |  |  |
| Second stage CD |  |  |  |  |
| No labour CD |  |  |  |  |
| Missing/ unknown CD |  |  |  |  |
| **Gestational age (weeks)** |  |  |  |  |
| Preterm (<37) |  |  |  |  |
| Term (37-41) | 1.0 (REF) |  | 1.0 (REF) |  |
| Post term (≥42) |  |  |  |  |
| **Induction** |  |  |  |  |
| Yes |  |  |  |  |
| No | 1.0 (REF) |  | 1.0 (REF) |  |
| **Augumentation** |  |  |  |  |
| Yes |  |  |  |  |
| No | 1.0 (REF) |  | 1.0 (REF) |  |
| **Fetal presentation** |  |  |  |  |
| Cephalic | 1.0 (REF) |  | 1.0 (REF) |  |
| Breech |  |  |  |  |
| Tranverse |  |  |  |  |
| Missing/ unknown |  |  |  |  |
| **Episiotomy** |  |  |  |  |
| Mediolateral |  |  |  |  |
| Midline |  |  |  |  |
| Unknown |  |  |  |  |
| None | 1.0 (REF) |  | 1.0 (REF) |  |
| **Infant birth weight (g)** |  |  |  |  |
| <3000 |  |  |  |  |
| 3000-3999 | 1.0 (REF) |  | 1.0 (REF) |  |
| 4000-4499 |  |  |  |  |
| ≥4500 |  |  |  |  |
| Missing |  |  |  |  |
| **Infant head circumference at birth (cm)** |  |  |  |  |
| <33 |  |  |  |  |
| 33-34 | 1.0 (REF) |  | 1.0 (REF) |  |
| 35-36 |  |  |  |  |
| ≥37 |  |  |  |  |
| Missing |  |  |  |  |

| **Statistical Model(s)** | | |
| --- | --- | --- |
| **Type of model** | Modified Poisson regression (with robust error variance)  -use GEE with exchangeable correlation structure to account for more than one pregnancy to an individual within the study window (use maternal person ID variable to identify individuals) | |
| **Primary independent variable** | Maternal Race | |
| **Dependent variable** | PPH, severe PPH | |
| **Covariates** | Maternal Age  Parity  Plurality  Pre-pregnancy Body Mass Index (kg/m^2^)  Material Deprivation Quintile (ON MARG)  Type of conception  Mode of delivery  First trimester prenatal visit  Number of prenatal visits  Diabetes (gestational and chronic)  Hypertension (gestational and chronic)  Previous cesarean delivery | |
| **Sensitivity Analyses** |  | |
| **Type of model** |  | |
| **Primary independent variable** |  | |
| **Dependent variable** |  | |
| **Covariates** |  | |
|  | |  |

| Quality Assurance Activities | | | |
| --- | --- | --- | --- |
| **RAE/DSH Directory of SAS Programs** |  | | |
| **RAE/DSH Directory of Final Dataset(s)** | *The* *final analytic dataset for each cohort includes all the data required to create the baseline tables and run all the models. It should include all covariates for all models such as patient risk factors, hospital characteristics, physician characteristics, exposure measures (continuous, categorical) and outcomes. It should include covariates that were considered but didn’t make the final cut. This would permit an analyst to easily re-run the models in the future.* | | |
|  |  | | |
| **RAE/DSH README file available:** Yes No | | | |
| **Date results of quality assurance tools for final dataset shared with project team (where applicable):** | | |  |
|  | | **%assign** | yyyy-mon-dd |
|  | | **%evolution** | yyyy-mon-dd |
|  | | **%dinexplore** | yyyy-mon-dd |
|  | | **%track / %exclude** | yyyy-mon-dd |
|  | | **%codebook** | yyyy-mon-dd |
| **Additional comments:** | |  | |
